# Clinical practice guidelines of the European Association for Endoscopic Surgery (EAES) on bariatric surgery: update 2020 endorsed by IFSO-EC, EASO and ESPCOP

**DOI:** 10.1007/s00464-020-07555-y

**Published:** 2020-04-23

**Authors:** Nicola Di Lorenzo, Stavros A. Antoniou, Rachel L. Batterham, Luca Busetto, Daniela Godoroja, Angelo Iossa, Francesco M. Carrano, Ferdinando Agresta, Isaias Alarçon, Carmil Azran, Nicole Bouvy, Carmen Balaguè Ponz, Maura Buza, Catalin Copaescu, Maurizio De Luca, Dror Dicker, Angelo Di Vincenzo, Daniel M. Felsenreich, Nader K. Francis, Martin Fried, Berta Gonzalo Prats, David Goitein, Jason C. G. Halford, Jitka Herlesova, Marina Kalogridaki, Hans Ket, Salvador Morales-Conde, Giacomo Piatto, Gerhard Prager, Suzanne Pruijssers, Andrea Pucci, Shlomi Rayman, Eugenia Romano, Sergi Sanchez-Cordero, Ramon Vilallonga, Gianfranco Silecchia

**Affiliations:** 1 Department of Surgical Sciences, University of Rome “Tor Vergata”, Rome, Italy; 2 Department of Surgery, European University of Cyprus, Nicosia, Cyprus; 3 Department of Surgery, Mediterranean Hospital of Cyprus, Limassol, Cyprus; 4 Centre for Obesity Research, University College London, London, UK; 5 Biomedical Research Centre, National Institute of Health Research, London, UK; 6 Internal Medicine 3, Department of Medicine, DIMED, Center for the Study and the Integrated Treatment of Obesity, University Hospital of Padua, Padua, Italy; 7 Department of Anesthesiology, Ponderas Academic Hospital Regina Maria, Bucharest, Romania; 8 Department of Medical-Surgical Sciences and Biotechnologies, Faculty of Pharmacy and Medicine, “La Sapienza” University of Rome-Polo Pontino, Bariatric Centre of Excellence IFSO-EC, Via F. Faggiana 1668, 04100 Latina, Italy; 9 Department of Endocrine and Metabolic Surgery, University of Insubria, Ospedale di Circolo and Fondazione Macchi, ASST Sette Laghi, Varese, Italy; 10 Department of General Surgery, ULSS5 del Veneto, Adria, Italy; 11 Unit of Innovation in Minimally Invasive Surgery, Department of General and Digestive Surgery, University Hospital “Virgen del Rocío”, 41010 Sevilla, Spain; 12 Herzliya Medical Center, Herzliya, Israel; 13 Department of Surgery, Maastricht University Medical Centre, Maastricht, The Netherlands; 14 Hospital Sant Pau, UAB, Barcelona, Spain; 15 Department of General Surgery, Ponderas Academic Hospital Regina Maria, Bucharest, Romania; 16 Division of General Surgery, Castelfranco and Montebelluna Hospitals, Treviso, Italy; 17 Department of Internal Medicine D, Hasharon Hospital, Rabin Medical Center, Sackler School of Medicine, Tel Aviv University, Tel Aviv, Israel; 18 Division of General Surgery, Department of Surgery, Vienna Medical University, Vienna, Austria; 19 Department of General Surgery, Yeovil District Hospital NHS Foundation Trust, Yeovil, UK; 20 Center for Treatment of Obesity and Metabolic Disorders, OB Klinika, Prague, Czech Republic; 21 Sackler School of Medicine, Tel Aviv University, Tel Aviv, Israel; 22 Department of Surgery C, Chaim Sheba Medical Center, Ramat Gan, Israel; 23 Department of Psychological Sciences, Institute of Psychology, Health and Society, University of Liverpool, Liverpool, UK; 24 Emergency Department, General Hospital of Attica “KAT”, Athens, Greece; 25 VU Amsterdam, Amsterdam, Netherlands; 26 General Surgery Department, Consorci Sanitari de L’Anoia, Barcelona, Spain; 27 Endocrine, Metabolic and Bariatric Unit, General Surgery Department, Vall D’Hebron University Hospital, Center of Excellence for the EAC-BC, Universitat Autònoma de Barcelona, Barcelona, Spain

**Keywords:** Bariatric surgery, Obesity, Guidelines, EAES, GRADE, AGREE II

## Abstract

**Background:**

Surgery for obesity and metabolic diseases has been evolved in the light of new scientific evidence, long-term outcomes and accumulated experience. EAES has sponsored an update of previous guidelines on bariatric surgery.

**Methods:**

A multidisciplinary group of bariatric surgeons, obesity physicians, nutritional experts, psychologists, anesthetists and a patient representative comprised the guideline development panel. Development and reporting conformed to GRADE guidelines and AGREE II standards.

**Results:**

Systematic review of databases, record selection, data extraction and synthesis, evidence appraisal and evidence-to-decision frameworks were developed for 42 key questions in the domains Indication; Preoperative work-up; Perioperative management; Non-bypass, bypass and one-anastomosis procedures; Revisional surgery; Postoperative care; and Investigational procedures. A total of 36 recommendations and position statements were formed through a modified Delphi procedure.

**Conclusion:**

This document summarizes the latest evidence on bariatric surgery through state-of-the art guideline development, aiming to facilitate evidence-based clinical decisions.

**Electronic supplementary material:**

The online version of this article (10.1007/s00464-020-07555-y) contains supplementary material, which is available to authorized users.

## Preamble

It has been 14 years since EAES has launched the 2004 guidelines on obesity surgery. A lot has changed in the field since then. “Historical” techniques developed by the pioneers of bariatric surgery were virtually abandoned (e.g. vertical gastroplasty). Plenty of innovations were added to the armamentarium of healthcare professionals for the operative and perioperative management of bariatric patients. Sleeve gastrectomy, although considered experimental in 2004, has become the most common bariatric procedure. Most recent techniques, such as gastric plication, one-anastomosis bypass and endoluminal procedures are gaining increasing attention. Reiterative (redo) surgery has gained the interest of several bariatric surgeons, although clear indications and even a common definition are lacking.

Importantly, laparoscopic surgery is now considered the gold standard approach for bariatric surgery. Under consideration of the above, these guidelines focus exclusively on minimally invasive bariatric surgery and common surgical techniques. Techniques which are now considered obsolete, although properly addressed in the previous guidelines, are not included in this update. Furthermore, the following topics are not addressed herein: modified laparoscopy (natural orifice transluminal, single-incision and robotic surgery), intragastric balloons, the impact of emerging technologies (3-D, fluorescence, hybrid) and pure metabolic surgery (without the obesity parameter).

Former standards of clinical guidance development, although of quality at the time, were replaced by the most evidence-based development and reporting standards summarized by the GRADE methodology and AGREE II guidelines. The support of the EAES Guideline Subcommittee is hereto commended.

The complex treatment of obesity and its comorbidities requires multidisciplinary integration. To this end, we invited the participation of European organizations involved in the research and management of obesity. The European Chapter of the International Federation for the Surgery of Obesity and Metabolic Disorders (IFSO-EC), the European Association for the Study of Obesity (EASO) and the European Society for the Peri-operative Care of the Obese Patient (ESPCOP) joined their forces with EAES to produce high quality work. Their representatives carried a wide variety of competencies (psychologists, obesity physicians, nutritional experts, anesthetists, laparoscopic surgeons) and comprised a concerted multidisciplinary panel. This is the first guideline with active involvement of a patient representative from the EASO patient task force.

Upon completion of the guideline manuscript, 2 prominent experts in bariatric surgery from outside Europe were invited to appraise the work against AGREE II criteria and provided their assessment with the AGREE II tool.

Finally, we are obligated to deeply thank all those who have contributed to this project, which we hope will contribute to the quality of healthcare in bariatric patients in Europe.

Nicola Di Lorenzo & Gianfranco Silecchia

Content Coordinators

Obesity is a multifactorial disease caused by a combination of genetic, environment and metabolic factors [1]. From a public health perspective, obesity is a major risk factor for a range of chronic diseases including type 2 diabetes, cardiovascular diseases and cancer [2].

### History

The first guidelines endorsing surgery for the management of morbid obesity were published in 1991 by the US National Institutes of Health (NIH) [3]. After this first regulatory act, several international guidelines and consensus projects recommended bariatric surgery as an effective treatment of weight loss and obesity-related comorbidities.

After the introduction of laparoscopic bariatric surgery, EAES and the Society of American Gastrointestinal and Endoscopic Surgeons (SAGES) launched clinical practice guidelines in 2004 and 2008, respectively [4, 5]. The “Interdisciplinary European Guidelines” on surgery for severe obesity were published in 2008, and updated in 2014 through an expert-based consensus process [6, 7].

### Rationale and objective

The growing burden of obesity in both industrialized and non-industrialized countries [2], the recognition of obesity as a disease in 2013 by the American Medical Association and other regulatory bodies [8], the ever-increasing research evidence in the field and the lack of recent clinical guidance, mandate an urgent need to incorporate latest evidence into clinical practice guidelines. EAES recognized this need and decided to sponsor the present update, which ultimately aims to inform healthcare in bariatric patients.

### Where do these guidelines apply?

These guidelines apply to adult (age > 18) patients with body mass index (BMI) > 35 kg/m^2^ who are considered fit for surgery and with no contraindications to laparoscopic surgery, unless otherwise indicated. They do not apply to the pediatric population. Healthcare systems, infrastructures, human and financial resources across European countries were considered upon developing these guidelines. Therefore, they are primarily intended to be applicable in European countries, although some recommendations might be applicable to other regions with modifications.

### Who are the target users?

The present guidelines may be used by healthcare professionals, including bariatric surgeons, laparoscopic surgeons, obesity physicians, anesthetists, general practitioners, nutritional experts, psychologists, obstetrics and gynecologists, anesthetic and intensive care unit staff; and may be used as a reference to policymakers, such as European and national authorities, healthcare administrators and health insurance providers, under consideration that the external validity may vary across countries, regions and healthcare institutions.

### How long are these guidelines valid for?

In view of current and ongoing research in the field, these guidelines are valid from publication up to December 2024. Target users are instructed to monitor upcoming research (research published from November 2018 onwards) which might provide evidence further supporting or even negating recommendations provided herein. For further information, see Disclaimer.

### Update and monitoring

The content coordinators will monitor the literature and will recommend an update of these guidelines in 2023, unless developments in the field and emerging evidence will suggest an earlier or later update.

A web-based survey of EAES, IFSO-EC, EASO and ESPCOP is planned to be launched in October 2021 to assess guidelines use among healthcare professionals and collect feedback on implementation barriers.

## Material and methods

Please see Supplementary file 1 for a detailed report of the methodology.

This guideline was developed in accordance with the GRADE methodology and complied with AGREE II guideline development and reporting standards [9, 10]. Institutional review board approval and written consent were not required. The systematic review and synthesis of evidence conformed to PRISMA and MOOSE reporting standards, as appropriate [11, 12].

### Guideline development group

The steering group consisted of bariatric surgeons, members of the EAES Consensus & Guideline Subcommittee and a GRADE methodologist [13]. The panel was comprised of bariatric surgeons, obesity physicians, nutritional experts, psychologists, anesthetists and a patient representative (“Appendix”).

### Topics

PICO (Patient, Intervention, Comparator, Outcomes) questions were organized into seven domains:

1. Indication for surgery.
2. Preoperative work-up.
3. Perioperative management.
4. Bariatric procedures.
5. Revisional surgery.
6. Postoperative care.
7. Investigational procedures.

A full list of PICO questions can be found in Supplementary file 2.

### Systematic review

The literature search was confined from 2005 onwards to capture the most pertinent evidence under consideration of advances in surgical techniques, operative equipment and accumulated surgical experience, and to serve as an update of previous EAES guidelines [4]. The last search was run in November 2018. The search summary and the search syntaxes are provided in Supplementary files 3 and 4. PRISMA flow charts of record selection are provided in Supplementary file 5.

We considered meta-analyses of randomized controlled trials (RCTs), meta-analyses of cohort studies, or individual RCTs and cohort studies addressing similar PICO frameworks to those of the predefined questions. Overarching inclusion criteria across PICO questions were adult patients (age > 18 years) with body mass index > 35 kg/m^2^ (unless otherwise indicated) and laparoscopic surgery (in relevant topic domains). Studies addressing bariatric procedures were considered for recommendation only if they provided data on weight loss with a follow-up of at least 5 years. Animal studies, studies on pediatric patients and on robotic or open surgery were discarded. A total of 65 systematic reviews were performed.

### Evidence synthesis

In the presence of a recent meta-analysis in the context of interest, summary effect measures and interval estimates, and risk of bias parameters were considered for assessment of the quality of evidence as per GRADE methodology [9, 14]. If no recent meta-analysis was available, we searched for relevant RCTs and/or cohort studies and we extracted summary data [15, 16]. We performed pairwise meta-analyses using a fixed or random-effects model, as appropriate. For adjustable gastric banding, we performed proportion meta-analysis to summarize the incidence of related complications and reoperations. Forest plots and funnel plots (where available) can be provided by the authors upon reasonable request.

We generated evidence tables, summarizing judgments on study design, risk of bias, inconsistency, indirectness, imprecision and the overall quality of evidence on each outcome of interest [17, 18].

### Evidence-to-decision framework

Predefined parameters were taken into account to formulate recommendations. More specifically, importance of the problem, desirable/undesirable effects and their balance, the certainty (quality) of evidence, patient values and preferences, acceptability to key stakeholders, cost of implementation and feasibility of incorporating the intervention into practice were considered through research evidence, where available, or through panel consensus [19]. Under consideration of these parameters, the panel provided for each PICO question:

- A strong recommendation for the intervention or the comparator,
- A conditional recommendation for the intervention or the comparator, or
- No recommendation (conditional recommendation for either the intervention or the comparator) [19].

If no recommendation could be formulated on a PICO question, the panel had the option to draft a position statement. Position statements reflect the opinion of the panel, are not necessarily based upon research evidence and should not be considered formal, evidence-based recommendations.

We used the GRADEpro GDT software (enterprise version) for generation of evidence tables, development of recommendations and Delphi process. [20].

### Delphi process

The recommendation drafts, along with background evidence and judgements on the above parameters, were subjected to a web-based Delphi process involving all panel members, using PanelVoice 2.0 add-on to GRADEpro. Three Delphi rounds took place overall.

### Survey

Members of participating societies were surveyed to investigate the applicability of recommendations to their practice. Further, attendees of the 27^th^ International Congress of EAES were invited to participate in an on-site survey using a smartphone application in a dedicated session. Results of the online survey are provided in Supplementary file 6.

### Appraisal

The full guideline in its final version was reviewed by 2 prominent obesity surgeons and was appraised using the AGREE II tool. Their appraisal can be found in Supplementary file 7.

## Results

**Fig. 1:**
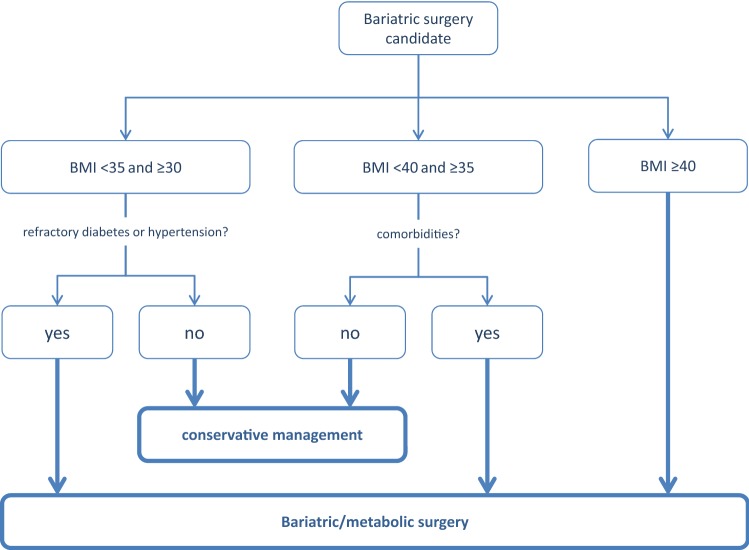
Evidence-based decision tree on the decision for bariatric surgery or conservative management. *BMI* body mass index. BMI values are kg/m^2^. Thick arrows and frames, and bold fonts indicate strong recommendation

**Fig. 2:**
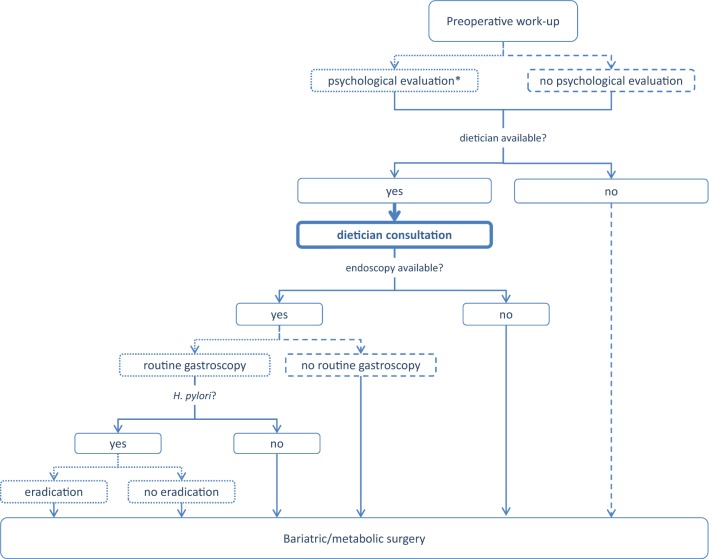
Evidence-based decision tree for preoperative work-up. *Psychological evaluation should be performed when psychological disorders are suspected. Binge eating and depression might not be a contraindication for bariatric/metabolic surgery. Thick arrows and frames, and bold fonts indicate strong recommendation. Dotted arrows and frames indicate conditional recommendation for the intervention. Dashed arrows and frames indicate conditional recommendation against the intervention

**Fig. 3:**
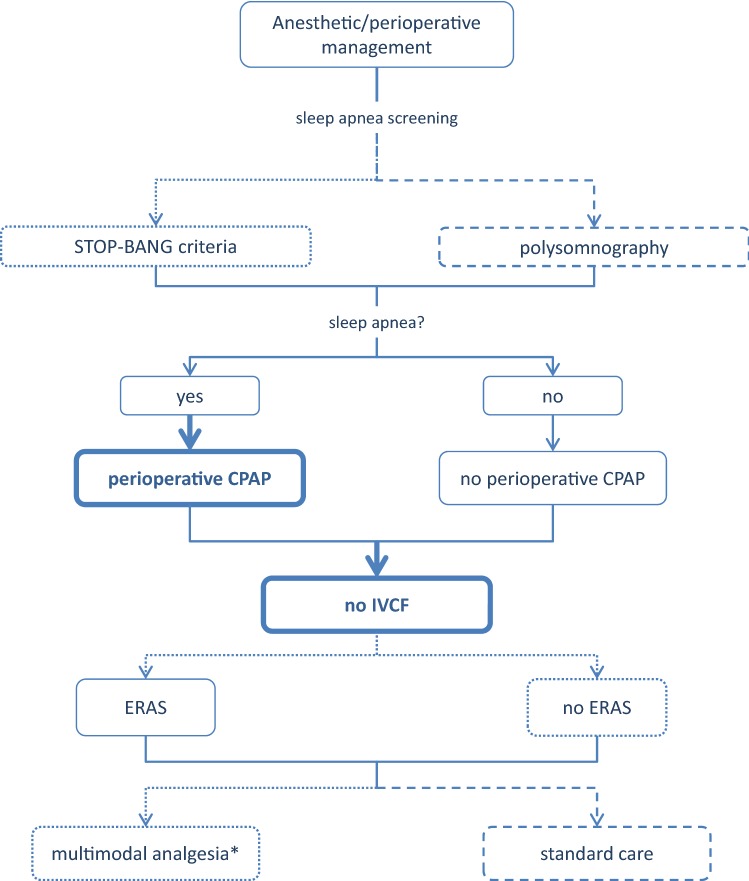
Evidence-based decision tree for anesthetic and perioperative management. *CPAP* continuous positive airway pressure, *IVCF* inferior vena cava filter, *ERAS* enhanced recovery after surgery. *with minimal use of opioids. Thick arrows and frames, and bold fonts indicate strong recommendation. Dotted arrows and frames indicate conditional recommendation for the intervention. Dashed arrows and frames indicate conditional recommendation against the intervention

**Fig. 4:**
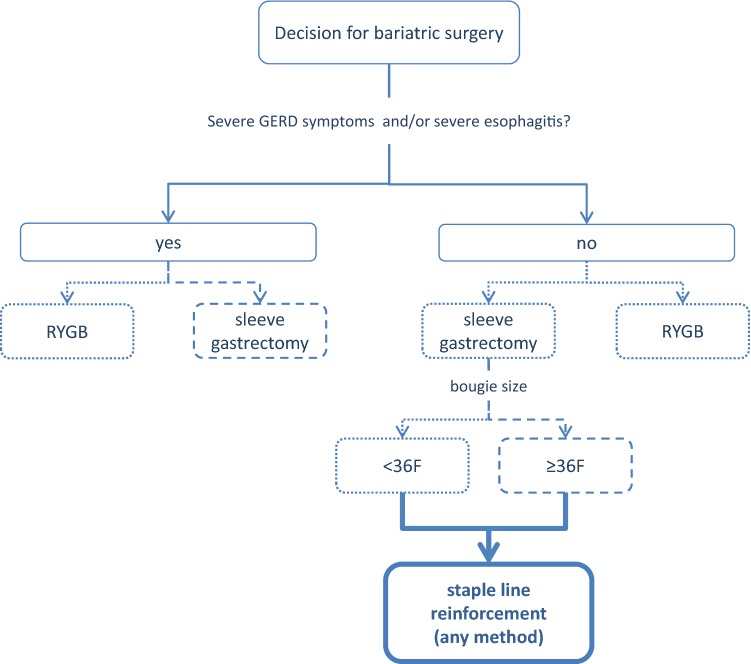
Evidence-based decision tree for the selection of operative approach. *BPD/DS* biliopancreatic diversion with duodenal switch, *AGB* adjustable gastric banding, *GERD* gastroesophageal reflux disease, *RYGB* Roux-en-Y gastric bypass. Thick arrows and frames, and bold fonts indicate strong recommendation. Dotted arrows and frames indicate conditional recommendation for the intervention. Dashed arrows and frames indicate conditional recommendation against the intervention

**Fig. 5:**
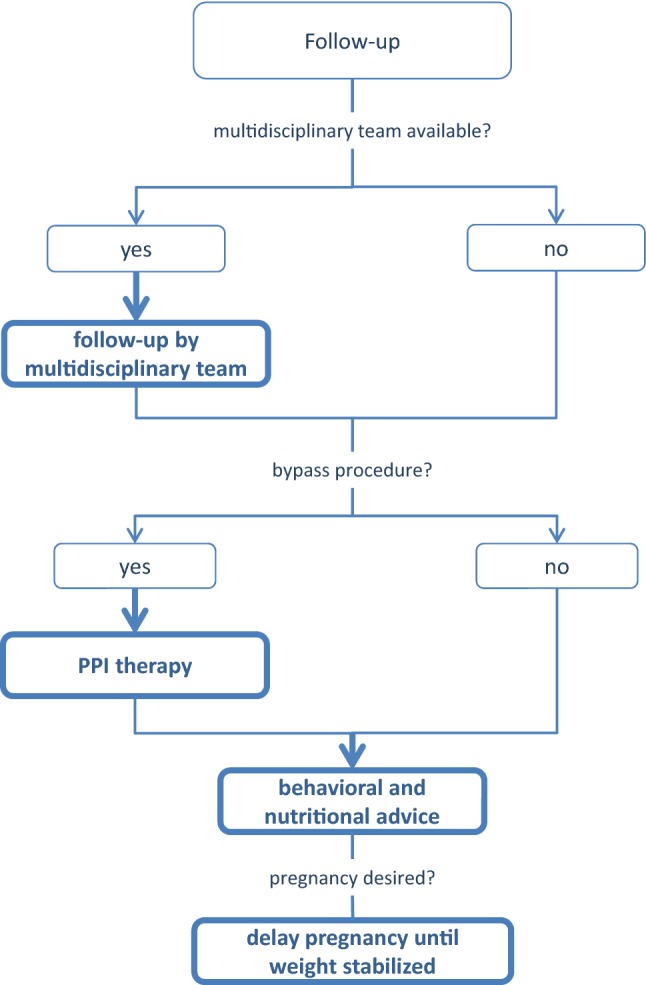
Evidence-based decision tree for postoperative follow-up. *PPI* proton-pump inhibitor. Thick arrows and frames, and bold fonts indicate strong recommendation

A summary list of recommendations can be found in Table 1. The decision trees depicted in Figs. 1, 2, 3, 4, and 5 schematically summarize the recommendations.

**Table 1:** Summary of recommendations

Indication for bariatric surgery	Laparoscopic bariatric surgery should be considered for patients with BMI ≥ 40 kg/m^2^ and for patients with BMI ≥ 35–40 kg/m^2^ with associated comorbidities that are expected to improve with weight loss	Strong
	Laparoscopic bariatric/metabolic surgery should be considered for patients with ≥ BMI 30–35 kg/m^2^ and type 2 diabetes and/or arterial hypertension with poor control despite optimal medical therapy	Strong
Preoperative work-up	No recommendation can be made for either routine *H. pylori* eradication or no eradication prior to bariatric surgery on the basis of available evidence	Conditional for either intervention or comparator
	Preoperative dietitian consultation should be considered for patients undergoing bariatric surgery	Strong
	Esophagogastroscopy can be considered as a routine diagnostic test prior to bariatric surgery	Conditional
	Psychological evaluation can be considered before bariatric surgery A previous diagnosis of binge eating or depression may not be considered as an absolute contraindication to surgery	Conditional
Perioperative management	Screening for obstructive sleep apnea using the STOP-BANG criteria can be considered prior to bariatric surgery	Conditional
	Perioperative CPAP should be considered in patients with severe obstructive sleep apnea syndrome who are undergoing bariatric surgery	Strong
	No recommendation can be made on the dose and duration of pharmacological thromboprophylaxis in patients after bariatric surgery	Conditional for either intervention or comparator
	Inferior vena cava filter is not recommended for thromboprophylaxis in patients undergoing bariatric surgery	Strong
	No recommendation for either an ERAS protocol or standard care can be made on the basis of available evidence	Conditional for either intervention or comparator
	Perioperative multimodal analgesia with minimal opioid usage may be considered in patients undergoing bariatric surgery	Conditional
Non-bypass procedures	Adjustable gastric banding surgeries are associated with a high rate of reoperations for complications or conversion to another bariatric procedure for insufficient weight loss in the long term	Position statement
	Sleeve gastrectomy may be preferred over adjustable gastric banding for weight loss and control/resolution of metabolic comorbidities	Conditional
	Sleeve gastrectomy may offer improved short-term weight loss and resolution of type 2 diabetes compared to gastric plication. No significant differences are observed at mid-term. Long-term comparative data on weight loss and metabolic effects are, however, lacking	Position statement
	There is insufficient evidence to recommend routine stapler line reinforcement^a^ to reduce the leak rate	Position statement
	Staple line reinforcement^a^ in sleeve gastrectomy should be considered to reduce the risk of perioperative complications^b^	Strong
	A bougie size < 36F compared to a bougie sized ≥ 36F may be recommended for calibration in sleeve gastrectomy as it is associated with greater weight loss in the mid-term	Conditional
	More extensive antral resection (2–3 cm from the pylorus versus > 5 cm antral preservation) potentially offers greater weight loss in the short term without a significant increase in post-operative complications. Long term data are, however, lacking	Position statement
Bypass procedures	RYGB should be preferred over adjustable gastric banding	Strong
	RYGB results in greater weight loss and control/remission of insulin resistance and type 2 diabetes compared to gastric plication	Position statement
	RYGB offers similar mid-term weight loss and control/remission of metabolic comorbidities compared to sleeve gastrectomy. Long-term comparative data are, however, lacking	Position statement
	RYGB can be preferred over sleeve gastrectomy in patients with severe gastroesophageal reflux disease and/or severe esophagitis	Conditional
	No recommendation for either BPD/DS or sleeve gastrectomy can be made on the basis of available comparative evidence	Conditional for either intervention or comparator
	With regard to mid-term weight loss there is no difference between BPD/DS and RYGB. BPD/DS is superior to RYGB for control/remission of type 2 diabetes. Long-term comparative data are, however, lacking	Position statement
One anastomosis procedures	OAGB may offer greater short-term weight loss compared to RYGB, gastric plication, adjustable gastric banding and sleeve gastrectomy. Long-term comparative data are, however, lacking. The effect on nutritional deficiencies remains controversial	Position statement
	No recommendation on SADI-S compared with OAGB, BPD/DS, RYGB or sleeve gastrectomy can be made on the basis of available evidence	Conditional for either intervention or comparator
Revisional surgery	No evidence-based criteria for indication to revisional bariatric/metabolic surgery are available to date The panel advises that the clinical decision to proceed to revisional bariatric/metabolic surgery be based on a complete multidisciplinary assessment of the patient, as recommended for the primary procedure	Position statement
Postoperative care	Scheduled multidisciplinary post-operative follow-up should be provided to every patient undergoing bariatric/metabolic surgery	Strong
	Treatment with ursodeoxycholic acid could be considered during the weight loss phase to prevent gallstones formation	Conditional
	Micro and/or macronutrients supplementation is recommended after bariatric surgery according to the type of the procedure and to the deficiencies documented during the follow-up	Strong
	PPI therapy should be given to patients undergoing bypass procedures for the prevention of marginal ulcers	Strong
	Postoperative nutritional and behavioral advice should be provided to patients undergoing bariatric surgery	Strong
	Pregnancy following bariatric surgery should be delayed during the weight loss phase	Strong
Investigational procedures	For duodenal-jejunal bypass sleeves, aspiration devices, gastric electrical stimulation, vagal blockade and duodenal mucosal resurfacing, the quality of evidence was too low to provide any recommendations	Position statement
	Endoluminal suturing procedures may have a role in the treatment of patients with obesity with BMI < 40 kg/m^2^	Position statement

Position statements do not constitute recommendations. *BMI* body mass index, *CPAP* continuous positive airway pressure, *ERAS* Enhanced recovery after surgery, *BPD/DS* biliopancreatic diversion with duodenal switch, *OAGB* one anastomosis gastric bypass, *SADIS* single-anastomosis duodeno-ileal switch, *PPI* proton-pump inhibitor

^a^Buttress, glues, suturing, clips

^b^Overall mortality, bleeding

## Topic 1: indication for bariatric surgery

### Bariatric surgery versus medical management for morbid obesity

**Table:** 

Laparoscopic bariatric surgery should be considered for patients with BMI ≥ 40 kg/m^2^ and for patients with BMI ≥ 35–40 kg/m^2^ with associated comorbidities that are expected to improve with weight loss *Strong recommendation* Laparoscopic bariatric/metabolic surgery should be considered for patients with ≥ BMI 30–35 kg/m^2^ and type 2 diabetes and/or arterial hypertension with poor control despite optimal medical therapy *Strong recommendation*	

#### Justification

Fifteen RCTs were identified comparing weight loss after bariatric surgery or medical therapy (5 reporting on RYGB, 3 on LAGB and the rest on mixed patient populations undergoing sleeve gastrectomy, BPD/DS, sleeve gastrectomy and/or banding) [21–35]. Random-effects meta-analysis was performed due to conceptual heterogeneity in operative interventions and non-operative management. A RCT performed in a mixed population (RYGB, sleeve gastrectomy, banding) reported a weighted mean difference (WMD) of 53% (95% confidence interval, CI, 42% to 63%) excess weight loss (EWL) compared to non-operative management [34]. Similarly, meta-analysis of 4 RCTs suggested a WMD of post-intervention weight of − 19 kg (95% CI − 27 to − 12) in favor of bariatric surgery, associated with moderate and low certainty of the evidence, respectively. These data lend support to the results of the Swedish Obese Subjects study, a large cohort study comparing bariatric surgery versus medical management in the very long term [36].

There were small or non-important differences for several metabolic surrogates. However, there was very strong association between bariatric surgery and type II diabetes (T2DM) resolution (odds ratio, OR, 29, 95% CI 13 to 67) and moderate reduction of systolic blood pressure. These effects were observed even in RCTs enrolling patients with BMI 30–35 kg/m^2^.

Non-operative management was associated with lower odds of complications (OR 2.44, 95% CI 1.47 to 4.06), although authors typically did not distinguish between minor and major complications, and certainty of the evidence was very low (Supplementary Table S1). There was insufficient evidence to support cost-effectiveness of operative management, however, the panel anticipated significant savings in terms of pharmacological management of comorbidities and other medical and social interventions.

## Topic 2: preoperative work-up

### Preoperative *H. pylori* eradication versus standard care in patients undergoing bariatric surgery

**Table:** 

No recommendation can be made for either routine *H. pylori* eradication or no eradication prior to bariatric surgery on the basis of available evidence. *Conditional recommendation for either the intervention or the comparator*	

#### Justification

There was no direct comparative observational evidence on the effect of *H. pylori* eradication in bariatric patients. One meta-analysis of 4 observational studies comparing *H. pylori*-positive- versus *H. pylori*-negative status was identified [37]. The odds for marginal ulcer (OR 0.51, 95% CI 0.03 to 8.35) and postoperative complications after bariatric surgery (OR 2.86, 95% CI 0.26 to 31.27) was similar for *H. pylori*-positive- versus *H. pylori*-negative patients, albeit interval estimates were extremely wide and uncertainty of the evidence high. Similarly, there was no firm evidence on postoperative bleeding (OR 0.90, 95% CI 0.23 to 3.52) or leakage (OR 1.62, 95% CI 0.17 to 15.62). Another meta-analysis, yielding seven studies with 255,435 patients, found similar results [38]. Multivariable analysis of a registry cohort found *H. pylori* status to be the most important independent predictor of marginal ulceration in patients undergoing RYGB, but it had little impact on the outcome of other bariatric operations [39]. Indirectness of the evidence and imprecision of effect estimates were major parameters to judge the quality of evidence, which was very low across outcomes (Supplementary Table S2). This is reflected in a conditional recommendation for either routine eradication or alternative practice.

### Preoperative diet consultation versus standard care in patients undergoing bariatric surgery

**Table:** 

Preoperative dietitian consultation should be considered for patients undergoing bariatric surgery *Strong recommendation*	

#### Justification

A meta-analysis reporting 3 RCTs was found on this topic [40]. Analyses were re-performed due to error in the primary meta-analysis (calculation of WMD instead of standardized MD, SMD). The overall quality of evidence was very low for weight loss and low for postoperative complications due to risk of bias across RCTs, inconsistency (conceptual and statistical heterogeneity due to variety of preoperative interventions for weight loss, and heterogeneity in the duration of follow-up) and indirectness (follow-up duration for weight loss insufficient for generalizability of findings). Postoperative weight loss was more pronounced in the preoperative diet consultation group (SMD 0.4, 95% CI 0.03 to 0.78 higher). No difference in the odds of postoperative complications was found (risk ratio, RR, 0.80, 95% CI 0.22 to 2.86), although interval estimates were wide. Confidence in the evidence was generally low (Supplementary Table S3), however the panel favored a strong recommendation after consulting with the patient representative who expressed a strong preference for a holistic approach of the bariatric patient with continuous preoperative and postoperative consultation. The panel considered this practice feasible, requiring moderate human and financial resources, and being acceptable to stakeholders. There was no evidence of any risk for the intervention according to the panel’s judgement.

### Preoperative endoscopy versus no endoscopy in patients undergoing bariatric surgery?

**Table:** 

Esophagogastroscopy can be considered as routine diagnostic test prior to bariatric surgery *Conditional recommendation*	

#### Justification

Two systematic reviews were available on this topic [7, 8, 41, 42] Proportion meta-analyses encompassing 23 observational studies and 6845 patients suggested a summary change in surgical management after esophagogastroscopy in 7.8% (95% CI 6.1 to 9.5%). Changes of surgical management included: hiatal hernia repairs, delays in surgery due to gastritis or peptic ulcer disease, major changes in the planned procedure and additional endoscopic dissection for suspicious lesions. Regarding a change in medical management, proportion meta-analysis of 20 observational studies reporting on 5140 patients found a management change in 27.5% (95% CI 20.2 to 34.8%) after esophagogastroscopy. Changes of medical management included primarily H. pylori eradication and initiation of proton-pump inhibitors or histamine blockers for gastritis or reflux [41]. The second meta-analysis demonstrated similar findings [42].

In view of the very low certainty owing primarily to risk of bias, inconsistency, publication bias, and questionable value in certain circumstances, hence indirectness (Supplementary Table S4), the panel provided a conditional recommendation for routine esophagogastroscopy, recognizing that selective endoscopy in patients with upper abdominal symptoms might be more appropriate.

### Assessment of preoperative psychological conditions versus no assessment in patients undergoing bariatric surgery

**Table:** 

Psychological evaluation can be considered before bariatric surgery A previous diagnosis of binge eating or depression may not be considered as an absolute contraindication to surgery *Conditional recommendation*	

#### Justification

In a meta-analysis of 26 observational studies, the prevalence of mental health disorders was higher among bariatric surgery candidates compared to the general population [43]. Furthermore, preoperative depression did not seem to be associated with postoperative weight loss, whereas there was conflicting evidence on binge eating. Due to the inconsistency of evidence, the variable availability of resources, and the uncertainty of the acceptability of the intervention to stakeholders, the panel provided a conditional recommendation for psychological evaluation before bariatric surgery. However, the treating physician should be alert to identify discrete signs of psychological disorders and refer those patients for further evaluation.

Similarly, due to the uncertainty of evidence and in view of the large beneficial effects of bariatric surgery on postoperative depression (Supplementary Table S5), the panel provided a conditional recommendation for bariatric surgery in the presence of a previous diagnosis of binge eating or depression. It should be recognized, however, that different interventions may have various effects on patients with different psychological backgrounds. Current data do not allow subgroup analyses to account for the above. Previous evidence suggests that most mental disorders (mood, anxiety, bipolar disorder, eating disorders etc.) might be considered as a contraindication for bariatric surgery if the conditions are severe and undertreated [44].

## Topic 3: perioperative management

### Screening versus no screening for obstructive sleep apnea in patients prior to bariatric surgery

**Table:** 

Screening for obstructive sleep apnea using the STOP-BANG criteria can be considered prior to bariatric surgery *Conditional recommendation*	

#### Justification

No observational evidence directly addressing the question was found. Meta-analysis of observational studies suggested that patients with obstructive sleep apnea or related disorders were more likely to sustain atrial fibrillation (OR 1.51, 95% CI 1.36 to 1.69) or hypoxemia (WMD − 3.8%, 95% CI − 5.4% to − 2.2%) [45–59]. The latter outcome might not be clinically important, whereas the summary certainty in the evidence was very low due to risk of bias (non-controlled confounders in cohort studies), imprecision, statistical and conceptual heterogeneity (differences in definition of sleep apnea and method of diagnosis) (Supplementary Table 6). Nevertheless, screening using the STOP-BANG criteria seemed to be predictive of postoperative complications in several observational studies [3, 4, 8, 9, 47, 48, 52, 53]. There was no evidence to support cost-effectiveness of diagnosis using STOP-BANG against polysomnography, however, the panel anticipated cost savings by identifying and offering intensive care to patients at risk. The panel provided a conditional recommendation for using the criteria for sleep apnea screening in candidates for bariatric surgery. There was agreement that, in case of clinical suspicion of sleep apnea, formal screening be performed.

### Perioperative continuous positive airway pressure (CPAP) versus no CPAP in patients with severe sleep apnea syndrome

**Table:** 

Perioperative CPAP should be considered in patients with severe obstructive sleep apnea syndrome who are undergoing bariatric surgery *Strong recommendation*	

Meta-analysis of observational studies suggested higher odds of postoperative pneumonia (OR 0.24, 95% CI 0.07 to 0.82), a trend towards lower odds of reintubation (OR 0.28, 95% CI 0.07 to 1.04), and shorter hospital stay (WMD − 1.6 days, 95% CI − 1.83 to − 1.28) albeit relevant evidence was of low certainty due to imprecision and the observational study design (Supplementary Table S7) [51, 52, 60, 61]. The panel provided a strong recommendation in spite of the low certainty of the evidence, due to the severity of these complications in the bariatric patient population and the low likelihood of harm associated with the intervention.

### High-dose versus standard-dose pharmacological antithrombotic prophylaxis after surgery

**Table:** 

No recommendation can be made on the dose and duration of pharmacological thromboprophylaxis in patients after bariatric surgery *Conditional recommendation for either the intervention or the comparator*	

#### Justification

Two RCTs were identified reporting on high versus standard dose low-molecular weight heparin after bariatric surgery [62, 63], whereas there was no evidence on the duration of prophylaxis. Meta-analysis suggested no significant difference with regard to deep venous thrombosis or bleeding, however, relevant evidence was moderate or low, due to substantial imprecision (Supplementary Table S8). The panel provided, therefore, conditional recommendation for either high dose or standard dose prophylaxis.

### Inferior vena cava filter versus standard care for prevention of thromboembolic events after bariatric surgery

**Table:** 

Inferior vena cava filter is not recommended for thromboprophylaxis in patients undergoing bariatric surgery *Strong recommendation*	

Meta-analysis of six observational studies suggested higher risk of deep venous thrombosis (OR 2.81, 95% CI 1.33 to 5.97), similar risk of pulmonary embolism (RR 1.02, 95% CI 0.31 to 3.37) and a trend towards higher risk of mortality with inferior vena cava filters (RR 3.27, 95% CI 0.78 to 13.64), albeit with wide interval estimates (Supplementary Table S9) [64]. Due to the importance of these outcomes and despite the low certainty of the evidence overall, this difference in effect estimates prompted the panel to provide a strong recommendation against the use of filters outside a research protocol.

### Enhanced recovery after surgery (ERAS) protocol versus standard care for bariatric surgery

**Table:** 

No recommendation for either an ERAS protocol or standard care can be made on the basis of available evidence *Conditional recommendation for either the intervention or the comparator*	

#### Justification

Two meta-analyses of 11 observational and randomized studies addressed the comparative effect of ERAS versus standard care in bariatric surgery [1, 2, 65, 66] No differences were found in major (OR 0.94, 95% CI 0.58 to 1.51) and minor postoperative complications (OR 0.88, 95% CI 0.55 to 1.41), and mortality (RR 0.4, 95% CI 0.1 to 2.2). Hospital stay was shorter for ERAS with a mean difference of 2.4 days (95% CI − 3.9 to − 0.9). The certainty of the evidence was very low primarily due to the observational study design, within-study risk of bias and wide interval estimates. Statistical inconsistency was also evident, probably reflecting conceptual heterogeneity of different ERAS protocols (Supplementary Table S10). In view of these findings, the panel did not favor either practice and invites further research.

### Multimodal analgesia with minimal use of opioids versus standard analgesia in bariatric surgery

**Table:** 

Perioperative multimodal analgesia with minimal opioid usage may be considered in patients undergoing bariatric surgery *Conditional recommendation*	

#### Justification

Four observational studies and seven RCTs were identified reporting on multimodal postoperative analgesia in laparoscopic bariatric surgery [3–13, 67–76] Random-effects meta-analyses to account for conceptual heterogeneity in analgesia protocols were performed. Most outcomes were addressed by few studies, hence effect estimates were not precise. Multimodal analgesia was associated with lower visual analog scale (VAS) scores and shorter stay in a post-anesthesia care unit (PACU). Of note, the odds for postoperative nausea and vomiting were lower with multimodal analgesia (OR 0.40, 95% CI 0.25 to 0.64). There was a marginal benefit of multimodal analgesia with regard to postoperative pneumonia (RD − 0.02, 95% CI − 0.05 to 0.00). There was high certainty of evidence for the latter findings and very low certainty for other critical outcomes (Supplementary Table S11), which prompted the panel to provide a conventional recommendation for multimodal analgesia with minimal use of opioids. This practice may be particularly considered in patients at increased risk for opioid sensitivity and obstructive sleep apnea.

## Topic 4: operative procedures

### Non-bypass procedures

## Adjustable gastric banding

**Table:** 

Position Statement Adjustable gastric banding surgeries are associated with a high rate of reoperations for complications or conversion to another bariatric procedure for insufficient weight loss in the long term	

### Justification

Proportion meta-analysis of randomized and observational studies found a pooled incidence of 20% (95% CI 13% to 26%, df = 10, *I*^2^ = 89%) for complications requiring surgical intervention, 2% (95% CI 1% to 3%, df = 7, *I*^2^ = 0%) for band erosion, 7% (95% CI 4% to 10%, df = 9, *I*^2^ = 72%) for band removal, 4% (95% CI 3% to 5%, df = 6, *I*^2^ = 0) for port revision, and 19% (95% CI 12 to 26%, df = 8, *I*^2^ = 85%) for overall complications [77–89].

Four studies with a follow-up of at least 5 years were identified. Port-related and band-related complications were documented for 18%, 23%, 27% and 43% of patients [78, 80, 85, 89]. Re-interventions for insufficient weight loss were reported by 3 studies at follow-up > 5 years, and documented for 6%, 12% and 18% of patients [78, 80, 85]. Port revisions occurred most commonly in the first year following surgery; reversals and conversions were more common during years 2 through 5. In view of this cumulative evidence, the panel provided a position statement on adjustable gastric banding.

### Sleeve gastrectomy versus adjustable gastric banding

**Table:** 

Sleeve gastrectomy may be preferred over adjustable gastric banding for weight loss and control/resolution of metabolic comorbidities *Conditional recommendation*	

#### Justification

Two network meta-analyses were available reporting on weight loss and diabetes remission [90, 91]. Sleeve gastrectomy was associated with a weighted mean difference of 25% EWL (95% CI 6.4% to 41%) and 57% higher odds of diabetes remission (OR 0.43, 95% CI 0.19 to 0.98). The network for weight loss was sparse with one direct and multiple indirect sources of evidence, which is the main reason for downgrading the certainty of evidence to low (Supplementary Table S12). Under consideration of the duration of follow-up (mean, 3 years, hence indirectness) and the high incidence of band-related complications, the panel provided a conditional recommendation for sleeve gastrectomy.

### Sleeve gastrectomy versus gastric plication

**Table:** 

Position statement Sleeve gastrectomy may offer improved short-term weight loss and resolution of type 2 diabetes compared to gastric plication. No significant differences are observed at mid-term. Long-term comparative data on weight-loss and metabolic effects are, however, lacking	

#### Justification

Analysis of long-term (≥ 5 years) and very long-term (≥ 10 years) evidence suggested an EWL between 42 and 55% with a mean weight regain between 9 and 31% for sleeve gastrectomy [9, 10, 92, 93]. Comparative long-term evidence was sparse, therefore, the panel provided a position statement under consideration of short- and mid-term outcomes. Meta-analysis of summary outcomes suggests a WMD of 31% (95% CI 10 to 72%) in favor of sleeve gastrectomy, but higher comparative odds for postoperative complications (OR 2.86, 95% CI 1.47 to 5.88) for the latter. Certainty of the evidence across outcomes was very low, primarily due to observational study design, inconsistency and imprecision (Supplementary Table S13).

### Technical considerations on sleeve gastrectomy: Staple line reinforcement

**Table:** 

Position statement There is insufficient evidence to recommend routine stapler line reinforcement* to reduce the leak rate *Buttress, glues, suturing, clips,	

**Table:** 

Recommendation Staple line reinforcement* in sleeve gastrectomy should be considered to reduce the risk of perioperative complications** *Strong recommendation* *Buttress, glues, suturing, clips, **Overall mortality, bleeding	

#### Justification

A meta-analysis of RCTs and a proportion meta-analysis addressed the topic of staple line reinforcement [94, 95]. Staple line reinforcement was associated with a 30% lower risk for complications overall (RR 0.7, 95% CI 0.5 to 0.9) and this finding was associated with high certainty. There was a trend towards lower risks of bleeding (RR 0.56, 95% CI 0.25 to 1.27) and leak (0.60, 95% CI 0.27 to 1.50) without reaching significance, however, effect estimates were imprecise and the certainty of evidence for these outcomes downgraded (Supplementary Table S14). Under consideration of the composite parameters, the feasibility, the cost and acceptability to stakeholders, the panel unanimously supported a strong recommendation. However, it should be noted that available evidence regards buttress material, glues, suturing and clips and external validity of these findings applies only to these interventions. Furthermore, evidence on the effect of buttressing material on leak was scarce and this is reflected in the panel’s position statement.

According to the Fifth International Consensus Summit on Sleeve Gastrectomy, 43% of surgeons preferred buttressing material for suture line reinforcement, 29% preferred oversewing and the remaining 28% did not use suture line reinforcement [96].

### Technical considerations on sleeve gastrectomy: Bougie size

**Table:** 

A bougie size < 36F compared to a bougie sized ≥ 36F may be recommended for calibration in sleeve gastrectomy as it is associated with greater weight loss in the mid-term *Conditional recommendation*	

#### Justification

A meta-analysis of observational studies comparing sleeve gastrectomy with bougie > 36F or < 36F was identified [97]. The use of bougie of smaller caliber was associated with more pronounced weight loss (SMD 0.23, 95% CI 0.14 to 0.33) and this finding was associated with moderate certainty. There was no difference in the odds for leak (OR 0.67, 95% CI 0.67 to 1.24), overall complications (OR 1.00, 95% CI 0.73 to 1.37) or gastroesophageal reflux (OR 0.77, 97% CI 0.37 to 1.59), albeit the certainty of the evidence was very low (Supplementary Table S15). The panel provided a conditional recommendation for the use of bougie sized < 36F.

The Fifth International Consensus Summit survey found that bariatric surgeons tend to use a larger bougie than previously recorded, the median size being 36F, most probably to avoid strictures and leak associated with stricture [96]. One of the widest differences between the consensus summit report of 2011 and 2014 is that more experts believe that smaller bougies are associated with stricture and leaks, hence the tendency to use bougies of larger diameter (from 65% in 2011 to 79% in 2014, *P* = 0.006) [96].

### Technical considerations on sleeve gastrectomy: antral resection

**Table:** 

Position Statement More extensive antral resection (2–3 cm from the pylorus versus > 5 cm antral preservation) potentially offers greater weight loss in the short term without a significant increase in post-operative complications. Long term data are, however, lacking	

#### Justification

A meta-analysis of 6 randomized and 2 observational studies addressed this topic [98]. Weight loss was more pronounced with antral resection (SMD 0.95, 95% CI 0.32 to 1.58), with no differences in staple line leak (RR 1.87, 95% CI 0.46 to 7.61), bleeding (RR 1.27, 95% CI 0.40 to 4.01) or gastroesophageal reflux (0.69, 95% CI 0.26 to 1.82). Nevertheless, certainty in the evidence was very low across outcomes due to the observational study design, risk of bias and imprecision (Supplementary Table S16). The panel decided that there was insufficient evidence to form a recommendation and a position statement was provided instead.

In the Fourth International Consensus Summit survey, bariatric surgeons reported that they resect the antrum at 4–5 cm (32%), 3–4 cm (27%), or 5–6 cm (22%) proximal to the pylorus [99].

## Bypass procedures

### Roux-en-Y gastric bypass (RYGB) versus adjustable gastric banding

**Table:** 

RYGB should be preferred over adjustable gastric banding *Strong recommendation*	

#### Justification

Two network meta-analyses including outcomes of pairwise comparisons addressed weight loss and diabetes remission after RYGB and adjustable gastric banding [90, 91]. The WMD of EWL was 22% (95% CI 6.5% to 34%) in favor of RYGB, which was associated with high certainty evidence. There was no difference in diabetes remission (RR 1.96, 95% CI 0.47 to 8.33), although certainty of the evidence was low (Supplementary Table S17). Nevertheless, mixed (direct and indirect) effect estimates were in favor of RYGB (RR 2.65, 95% CI 1.16 to 6.07) [91]. There was no summary evidence of perioperative complications, however, the panel unanimously supported a strong recommendation for RYGB over adjustable gastric banding, as it was judged that benefits outweigh potential risks.

### RYGB versus gastric plication

**Table:** 

Position Statement RYGB results in greater weight loss and control/remission of insulin resistance and type 2 diabetes compared to gastric plication	

#### Justification

Aggregate data were available for the outcome diabetes remission [91]. A network meta-analysis found RYGB to be associated with higher odds for remission compared to gastric plication (RR 4.00, 95% CI 1.40 to 11.11), albeit certainty was low due to imprecision and risk of bias (Supplementary Table S18). Mixed effect estimates were more precise (RR 2.86, 95% CI 1.17 to 6.98), however, still wide. The panel considered this evidence to be insufficient to form a recommendation and, in view of the scarcity of long-term data, provided a position statement instead. The statement on weight loss is based on indirect and empirical evidence suggesting a durable effect of weight loss compared to gastric plication.

### RYGB versus sleeve gastrectomy

**Table:** 

Position Statement RYGB offers similar mid-term weight loss and control/remission of metabolic comorbidities compared to sleeve gastrectomy. Long-term comparative data are, however, lacking RYGB can be preferred over sleeve gastrectomy in patients with severe gastroesophageal reflux disease and/or severe esophagitis *Conditional recommendation*	

#### Justification

A meta-analysis of observational studies, two meta-analyses of RCTs and two network meta-analyses addressed the comparative outcomes of RYGB and sleeve gastrectomy [90, 91, 100–102]. There was no significant difference in EWL (WMD − 4%, 95% CI − 14% to 8%) or diabetes remission (RR 0.89, 95% CI 0.73 to 1.06), findings supported by moderate certainty of evidence. There was marginal difference in major operative morbidity (OR 2.04, 95% CI 1.00 to 4.16), no differences in minor perioperative complications (OR 1.43, 95% CI 0.60 to 3.23), and long-term minor (OR 0.64, 95% CI 0.28 to 1.47) or major complications (OR 0.64, 95% CI 0.21 to 1.96), although the latter outcomes were associated with low or very low certainty of evidence. Remission of dyslipidemia and hypertension were in favor of sleeve gastrectomy, but certainty of the evidence was very low due to observational study design, risk of bias, inconsistency and indirectness (Supplementary Table S19).

Two RCTs addressed gastroesophageal reflux after RYGB and sleeve gastrectomy [103, 104]. Remission of pre-existing gastroesophageal reflux (absolute difference − 0.36, 95% CI − 0.57 to − 0.15) and de novo gastroesophageal reflux was more often seen after sleeve gastrectomy (absolute difference − 0.31%, 95% CI − 0.08% to − 0.54%) (moderate and low certainty of evidence). Under consideration of the low certainty of evidence in important outcomes and the lack of long-term (> 5 years) data, the panel provided a position statement on the comparative effect in the general bariatric population and a conditional recommendation for patients with reflux disease.

### Biliopancreatic diversion with duodenal switch (BPD/DS) versus sleeve gastrectomy

**Table:** 

No recommendation for either BPD/DS or sleeve gastrectomy can be made on the basis of available comparative evidence *Conditional recommendation for either the intervention or the comparator*	

#### Justification

Evidence on BPD/DS was very scarce, probably due to limited diffusion of this technique in bariatric surgeons [105]. Only 2 cohort studies were identified, which addressed morbidity and mortality [106, 107]. Effect estimates was summarized with meta-analysis, however, the certainty of evidence was very low due to imprecision (Supplementary Table S20). As such, the panel did not provide a recommendation for BPD/DS or sleeve gastrectomy.

### BPD/DS versus RYGB

**Table:** 

Position Statement With regard to mid-term weight loss there is no difference between BPD/DS and RYGB. BPD/DS is superior to RYGB for control/remission of type 2 diabetes. Long-term comparative data are, however, lacking	

#### Justification

Four RCTs were identified and outcomes were meta-analyzed [27, 108–110]. Despite low risk of bias across trials, effect estimates were imprecise and indirectness significant, because no long-term data were available. EWL was similar (WMD 14%, 95% CI − 12.21 to 42.15, very low certainty), whereas long-term morbidity (OR 3.38, 95% CI 1.14 to 10.05, low certainty) and diabetes remission (OR 8.06, 95% CI 1.35 to 48.14) were in favor of RYGB (Supplementary Table S21).

A matched cohort study on 73,702 patients from the Bariatric Outcomes Longitudinal Database reported BPD/DS to be associated with the greatest adjusted change in BMI compared to RYGB and sleeve gastrectomy. The study also suggested that BPD/DS was superior in terms of diabetes remission [111].

Due to conflicting evidence and generally low certainty across outcomes, no recommendation was provided by the panel.

## One anastomosis procedures

### One anastomosis gastric bypass (OAGB)

**Table:** 

Position Statement OAGB may offer greater short-term weight loss compared to RYGB, gastric plication, adjustable gastric banding and sleeve gastrectomy. Long-term comparative data are, however, lacking. The effect on nutritional deficiencies remains controversial	

#### Justification

OAGB is an emerging bariatric procedure which gains increasing interest among bariatric surgeons. According to the First IFSO Consensus Statement, the panel unanimously supported that OAGB is an acceptable mainstream surgical option and 96% considered that it may no longer be regarded as new or experimental procedure [112].

As a recently developed procedure, relevant evidence was limited. Certainty of the evidence was moderate across most outcomes for the comparison OAGB versus RYGB with only 2 RCTs, which were meta-analyzed, and a network meta-analysis addressing the comparison [91, 113, 114] .Quality of life and resolution of comorbidities was similar. OAGB was associated with marginally reduced odds for in-hospital morbidity (OR 0.38, 95% CI 0.13 to 1.14) and late complications (0.76, 95% CI 0.33 to 1.77) at the expense of less pronounced EWL (WMD 13%, 95% CI 2% to 29%; very low certainty) (Supplementary Table S22).

A meta-analysis of four observational studies compared AGB with one anastomosis gastric bypass (OAGB) [115]. OAGB was associated with lower postoperative BMI (WMD − 7 kg/m^2^, 95% CI − 9 to − 4) and smaller waist circumference (WMD − 14 cm, 95% CI − 27 to − 1), whereas there was no difference in diabetes remission (RR 1.48, 95% CI 0.98 to 2.25) at a mean follow-up of 1 year (Supplementary Table S23).

Two meta-analyses addressed the comparison OAGB versus sleeve gastrectomy [116, 117]. As they combine randomized and observational data, we meta-analyzed RCTs only to increase certainty in the evidence, when possible [114, 118]. EWL was more pronounced with sleeve gastrectomy (WMD 20%, 95% CI 20 to 23) and this finding was supported with high certainty in the evidence. The analysis favored sleeve gastrectomy in terms of diabetes and dyslipidemia remission and there was a trend towards lower odds for morbidity for OAGB (OR 0.67, 95% CI 0.28 to 1.61) (Supplementary Table S24). One RCT compared OAGB versus gastric plication, which provided very limited evidence (Supplementary Table S25) [119].

Under consideration of the lack of long-term data, the panel provided a position statement and no recommendation.

### Single anastomosis duodeno-ileal bypass with sleeve gastrectomy (SADI-S)

**Table:** 

No recommendation on SADI-S compared with OAGB, BPD/DS, RYGB or sleeve gastrectomy can be made on the basis of available evidence *Conditional recommendation for either the intervention or the comparator*	

#### Justification

SADI-S represents a simplified modification of the BPD/DS. There was limited evidence across comparisons.

One observational study investigated the comparative effect of SADI-S and RYGB, providing very low certainty evidence (Supplementary Table S26) [120]. Similarly, one observational study addressed SADI-S versus BPD/DS (Supplementary Table S27) and one addressed SADI-S versus sleeve gastrectomy (Supplementary Table S28) [121, 122].

## Topic 5: revisional surgery

**Table:** 

Position Statement No evidence-based criteria for indication to revisional bariatric/metabolic surgery are available to date The panel advises that the clinical decision to proceed to revisional bariatric/metabolic surgery be based on a complete multidisciplinary assessment of the patient, as recommended for the primary procedure	

### Terminology

The increasing use of bariatric/metabolic surgery was accompanied by a parallel increase of the number of patients who received an additional bariatric procedure after the index one [123]. In 2014, the American Society for Metabolic & Bariatric Surgery performed a systematic review on re-operative bariatric surgery and proposed a nomenclature for dividing the secondary procedures based on the technical aspects (Supplementary Table S29) [124].

Reoperations after bariatric surgery may be primarily performed for two reasons: (a) to solve or fix complications or side effects linked to the primary procedures; (b) to improve the results in patients with insufficient weight loss, continued or poorly controlled comorbid disease, or weight regain. We suggest the use of the term *revisional bariatric/metabolic surgery* only for re-operative procedures performed for the second group of indications. Revisional surgery can correct or convert the primary procedure. The surgical procedures targeting the first category of indications are re-interventions indicated by the patient’s medical condition, performed electively or in emergency, and they can include conversions, corrective or reversal procedures.

Severe obesity is a chronic disease that requires lifetime treatment. While bariatric/metabolic surgery is usually an effective and durable therapy, there will be patients who respond well to the initial therapy and others with only partial response, as in many other chronic diseases requiring medical or surgical therapy. There will be also a subset of patients having recurrent or persistent disease. These patients may require escalation of therapy or a new treatment modality [124]. We, therefore, suggest that the term “failure” or “failed” in respect to metabolic/bariatric procedures be abandoned. The term “non-responders” should be adopted because it is more consistent with the frame of obesity as chronic disease.

### Clinical indications for revisional surgery

We define as *revisional bariatric/metabolic surgery* any re-operative bariatric procedure performed to improve the results in patients with insufficient weight loss, continued comorbid disease, or weight regain. However, an established consensus on which levels of insufficient weight loss or weight regain should be considered as indicators for the need of a revisional procedure is still lacking.

Bonouvrie et al. recently performed a systematic review illustrating the lack of standard definitions of non-responders after bariatric surgery [125]. This is partly due to heterogeneity among studies which precludes evidence synthesis for revisional surgery. There is an urgent need to introduce standard definitions to be used in future research and clinical practice. Current definitions remain arbitrary, due to the lack of solid evidence in this field. A set of diagnostic criteria is proposed in Supplementary Table S30, taking into consideration current indications for bariatric/metabolic surgery and the evidence on the positive effects of a 10% weight loss [126].

### Work-up in case of revisional surgery

Current evidence suggests that revisional surgery may confer an improvement of obesity and obesity-related comorbidities in patients without optimal results after an index procedure. Escalation of therapy in patients with poor response should be considered rational in the long-term management of a chronic disease such as obesity [124]. On the other hand, revisional bariatric surgery confers a higher risk of perioperative complications than primary bariatric surgery [127]. The individual risk/benefit analysis for revisional surgery is, therefore, even more complex than for index procedures. We suggest that the clinical decision to proceed to revisional bariatric surgery be based on a complete multidisciplinary assessment, as recommended for the primary procedure, including endoscopic and radiological studies, with detailed information about the index procedure and proper evaluation of nutritional and behavioral status.

## Topic 6: postoperative care

### Scheduled multidisciplinary post-operative follow-up versus standard care

**Table:** 

Scheduled multidisciplinary post-operative follow-up should be provided to every patient undergoing bariatric/metabolic surgery *Strong recommendation*	

#### Justification

A meta-analysis of summary data from five RCTs reporting patients undergoing a variety of bariatric procedures and assessing the impact of scheduled multidisciplinary post-operative follow-up versus standard care reported more pronounced weight loss in the treatment group [128]. Scheduled multidisciplinary post-operative follow-up resulted in slightly greater EWL (WMD 1.6%, 95% CI 0.82% to 2.38%) compared to the control group (Supplementary Table S31). Despite this marginal effect and the low certainty in the evidence because of risk of bias and inconsistency, the panel provided a strong recommendation after consulting with the patient representative, who expressed a strong preference for close continuous preoperative and postoperative consultation. The panel considered this practice feasible, requiring moderate human and financial resources, and being acceptable to stakeholders. There was no evidence of any risk for the intervention according to the panel’s judgement.

### Treatment with ursodeoxycholic acid during the weight loss phase following bariatric surgery

**Table:** 

Treatment with ursodeoxycholic acid could be considered during the weight loss phase to prevent gallstones formation *Conditional recommendation*	

#### Justification

A meta-analysis of 6 RCTs and two observational studies found that ursodeoxycholic acid treatment versus no treatment was associated with lower odds of gallstone formation (OR 0.20, 95% CI 0.13 to 0.33) [129]. Due to mixed randomized and observational study design, substantial risk of bias and publication bias, certainty in the evidence was very low (Supplementary Table S32). Under consideration of the low-risk profile and the low cost of the intervention, the panel provided a conditional recommendation, recognizing, however, that further research is warranted.

### Supplementation of micro and/or micronutrients after bariatric surgery

**Table:** 

Micro and/or macronutrients supplementation is recommended after bariatric surgery according to the type of the procedure and to the deficiencies documented during the follow-up *Strong recommendation*	

#### Justification

Studies reporting micro and/or macronutrients supplementation post-surgery are limited. One meta-analysis of 5 RCTs and 7 observational studies was identified, which evaluated the effect of vitamin D supplementation on preventing Vitamin D deficiency [130]. Vitamin D deficiency was more common in the no supplementation group (OR 3.82, 95% CI 1.70 to 8.57) (Supplementary Table S33). Despite the sparse evidence, the panel decided to provide a strong recommendation, as it considered that the anticipated benefits outweigh the potential risks of such practice.

### Proton-pump inhibitor (PPI) therapy after bariatric surgery for the prevention of marginal ulcers

**Table:** 

PPI therapy should be given to patients undergoing bypass procedures for the prevention of marginal ulcers *Strong recommendation*	

#### Justification

A meta-analysis of three cohort studies that compared the comparative effect of PPIs on marginal ulcers suggested beneficial effect of PPI treatment (OR 0.50, 95% CI 0.29 to 0.90, moderate certainty) (Supplementary Table S34) [131]. Under consideration of the risk/benefit ratio, the low cost and acceptability of the intervention, the panel provided a strong recommendation.

### Postoperative nutritional and behavioral advice versus standard care

**Table:** 

Postoperative nutritional and behavioral advice should be provided to patients undergoing bariatric surgery *Strong recommendation*	

#### Justification

Evidence supporting the need for postoperative nutritional and behavioral counseling is supported by a meta-analysis of 6 RCTs [132]. At 12-moth follow-up, the WMD of EWL was 11% (95% CI 3% to 19%) in favor of the intervention. Certainty of the evidence was, however, very low, due to risk of bias, inconsistency and indirectness because of the variety of interventions (Supplementary Table S35). Under consideration of patient preferences and the anticipated feasibility and moderate resources, the panel supported a strong recommendation.

### Delaying pregnancy following bariatric surgery until after the weight loss phase versus no delay on fetal complications

**Table:** 

Pregnancy following bariatric surgery should be delayed during the weight loss phase *Strong recommendation*	

#### Justification

Six observational studies reporting fetal outcomes following pregnancy after bariatric surgery were identified and a meta-analysis comparing early pregnancy versus delayed pregnancy was undertaken [133–138]. Delaying pregnancy was associated with a trend towards lower odds for admission in the neonatal intensive care unit (OR 0.73, 95% 0.45 to 1.18). There were no further substantial findings, however, certainty was very low across outcomes (Supplementary Table S36). Despite this sparsity of evidence, the panel considered prudent to provide a strong recommendation for delaying pregnancy during the weight loss phase to avoid fetal complications that may not have been captured by current studies.

## Topic 7: investigational procedures

**Table:** 

Position statement For duodenal-jejunal bypass sleeves, aspiration devices, gastric electrical stimulation, vagal blockade and duodenal mucosal resurfacing, the quality of evidence was too low to provide any recommendations	

**Table:** 

Endoluminal suturing procedures may have a role in the treatment of obese patients with BMI below 40 kg/m^2^	

### Justification

Evidence was limited for emerging bariatric interventions and long-term data were not available. A meta-analysis of four RCTs that compared EndoBarrier® with non-surgical management was identified [139]. EWL was 36.9% (95% CI 29% to 45%) at 1-year follow-up (moderate certainty, Supplementary Table S37). EndoBarrier® was reported to be associated with 15.7% severe adverse events [139].

One RCT has compared AspireAssist® with lifestyle interventions and 1-year follow-up [140]. The WMD of EWL was 22% (95% CI 14% to 30%), HbA1c improvement was minimal (WMD 0.14%, 95% CI 0% to 0.28%), whereas evidence on morbidity was associated with low certainty (Supplementary Table S38). No severe complications were reported.

Three cohort studies reported on the abiliti® device and 1-year follow-up [141–143]. EWL ranged from 28.7 to 49.3%. Few self-limiting adverse events were reported and 2% severe adverse events. Certainty of the evidence was, however, very low.

Two RCTs assessing the effect of vBloc® reported 17% and 24% EWL at 1-year follow-up [144, 145]. Two-year follow-up suggested sustained weight loss [146, 147]. The technical difficulty of the procedure and a high rate of severe adverse events are significant drawbacks to this intervention.

One cohort study reported on duodenal mucosal resurfacing, relevant evidence being very low [148]. Two RCTs and 1 cohort study investigated the effect of the Pose® procedure and reported EWL between 16–45% [149–151]. The procedure safety profile seems acceptable, however certainty in the evidence was very low.

Five observational studies have addressed the use of OverStitch™ [152–156]. The procedure may be considered safe, well tolerated and effective with a mean EWL of 50% at 1 year. Evidence suggests durability of plications and progressive weight loss up to 2 years.

## Comments

### Survey results

The guideline development group aimed to investigate whether recommendations and position statements are applicable and can be transferred to individuals’ clinical practice. Indeed, one of the AGREE II domains is focused on the applicability of the guideline in the practice of target users. The rationale was therefore both to assess the clinical merit of the recommendations (as an aspect of external validity) and to inform the topics of future updates.

A total of 220 professionals involved in the management of patients with obesity responded to the 38 survey questions. The majority of survey participants were surgeons. Specifically, bariatric surgeons accounted for 61% of the total and 62% of participants worked in high volume bariatric centers (defined as > 50 bariatric procedures per year).

The majority of recommendations were considered applicable to participants’ practice. Among those where applicability was judged as low, was the recommendation on routine *H. pylori* eradication. This may be due to the fact that preoperative esophagogastroscopy is not considered standard preoperative study in many centers. Neutral recommendations were considered, as expected, to be not applicable by a substantial proportion of participants.

### Implications for future actions

The present guidelines summarize pertinent evidence in the field of bariatric surgery. Despite the advances in the field, we have identified several gaps, particularly in the long-term reporting of outcomes [90, 91]. Furthermore, we have identified only two network meta-analyses, which reported several outcomes of interest. Considering the variety of treatment options, network meta-analysis is the optimal method to summarize evidence across interventions in the same meta-analytical model and is undoubtedly a prosperous field of research.

Follow-up reports of emerging procedures, such as OAGB, and further, large-scales RCTs on investigational procedures, with robust methodology are eagerly awaited. As the incidence of obesity increases in societies with a high prevalence of psychological disorders, further investigation on the indications for bariatric surgery is warranted. Furthermore, obesity is an emerging problem in developing countries and, as such, healthcare authorities are called to promote health equity by ensuring access to healthcare for underprivileged and vulnerable populations.

## Conclusions

Evidence from clinical research suggests that bariatric surgery is highly effective in the management of obesity. This document summarizes the latest evidence on bariatric surgery. It was developed in compliance with state-of-the art methodological principles to reliably appraise evidence, hereby facilitating evidence-based clinical decisions and informing authoritative actions of policymakers and other stakeholders.

### Electronic supplementary material

Below is the link to the electronic supplementary material.

Supplementary file1 (DOCX 126 kb)

Supplementary file2 (DOCX 22 kb)

Supplementary file3 (DOCX 37 kb)

Supplementary file4 (DOCX 112 kb)

Supplementary file5 (DOCX 1792 kb)

Supplementary file6 (DOCX 733 kb)

Supplementary file7 (DOCX 125 kb)

Supplementary file8 (PDF 149 kb)

Supplementary file9 (PDF 113 kb)

Supplementary file10 (PDF 107 kb)

Supplementary file11 (PDF 107 kb)

Supplementary file12 (PDF 57 kb)

Supplementary file13 (PDF 121 kb)

Supplementary file14 (PDF 102 kb)

Supplementary file15 (PDF 67 kb)

Supplementary file16 (PDF 116 kb)

Supplementary file17 (PDF 120 kb)

Supplementary file18 (PDF 141 kb)

Supplementary file19 (PDF 61 kb)

Supplementary file20 (PDF 141 kb)

Supplementary file21 (PDF 115 kb)

Supplementary file22 (PDF 112 kb)

Supplementary file23 (PDF 71 kb)

Supplementary file24 (PDF 67 kb)

Supplementary file25 (PDF 63 kb)

Supplementary file26 (PDF 34 kb)

Supplementary file27 (PDF 68 kb)

Supplementary file28 (PDF 135 kb)

Supplementary file29 (PDF 101 kb)

Supplementary file30 (PDF 90 kb)

Supplementary file31 (PDF 69 kb)

Supplementary file32 (PDF 73 kb)

Supplementary file33 (PDF 70 kb)

Supplementary file34 (PDF 108 kb)

Supplementary file35 (PDF 135 kb)

Supplementary file36 (DOCX 46 kb)

Supplementary file37 (DOCX 88 kb)

Supplementary file38 (PDF 103 kb)

Supplementary file39 (PDF 63 kb)

Supplementary file40 (PDF 104 kb)

Supplementary file41 (PDF 102 kb)

Supplementary file42 (PDF 111 kb)

Supplementary file43 (PDF 103 kb)

Supplementary file44 (PDF 70 kb)

Supplementary file45 (PDF 71 kb)

Supplementary file46 (PDF 63 kb)

Supplementary file47 (PDF 70 kb)

Supplementary file48 (PDF 104 kb)

Supplementary file49 (PDF 107 kb)

Supplementary file50 (PDF 106 kb)
