# Supplementary material for: Clinical practice guidelines of the European Association for Endoscopic Surgery (EAES) on bariatric surgery: update 2020 endorsed by IFSO-EC, EASO and ESPCOP
Source: Surg Endosc. 2020 Apr 23;34(6):2332–58. doi: 10.1007/s00464-020-07555-y (PMC7214495; doi:10.1007/s00464-020-07555-y)
Supplement: Supplementary file 1 — Supplementary file1 (DOCX 126 kb) [file 464_2020_7555_MOESM1_ESM.docx]

**Supplementary file 1**

**DETAILED GUIDELINE DEVELOPMENT METHODOLOGY**

This guideline was developed according to the GRADE methodology and complied with AGREE II reporting and development standards.^1,2^ The systematic review and evidence synthesis conformed to PRISMA or MOOSE reporting standards, as appropriate.^3,4^

*Guideline development group*

A face-to-face kick-off meeting took place in London on June 1^st^, 2018. The steering group consisted of bariatric surgeons, members of the EAES Consensus & Guideline Subcommittee and a GRADE methodologist.^5^ The latter provided methodological guidance throughout the project. A panel of experts was invited on the basis of their research activity in the field of bariatric surgery. The following scientific societies were represented by panel members: EAES, IFSO-European Chapter, EASO and ESPCOP.

The panel consisted of bariatric surgeons, obesity physicians, nutritional experts, psychologists, anesthetists and a patient representative. Panel members nominated junior members who performed article screening and selection, data extraction, data synthesis, assessment of the quality of evidence and interpretation of findings, under supervision or in liaison with the former and under guidance from the methodologist. Junior members participated in 4 hourly web-based interactive seminars on guideline development methodology and 2 hourly web-based demonstrations of the Rayyan and the GRADEpro GDT platforms using a digital audio-visual platform (StartMeeting**^®^**). The steering group, the panel and junior members comprised the guideline development group (GDG).

*Topic selection*

A draft of PICO questions was developed by the steering group and was refined through email communication with panel members. PICO questions were organized in 7 domains:

1. Indication to surgery
2. Preoperative work-up
3. Perioperative management
4. Bariatric procedures
5. Revisional surgery
6. Postoperative care
7. Investigational procedures

*Systematic review*

A clinical information specialist developed topic-specific search strategies to identify relevant articles. The literature search was confined from 2005 onwards to capture the most pertinent evidence under consideration of advances in surgical techniques, operative equipment and accumulated surgical experience, and to serve as an update of previous EAES guidelines.^6^ The databases of the National Library of Medicine (Medline) and Exerpta Medica (EMBASE) were queried. Further relevant articles which members of the GDG were aware of were also considered. Reference lists of systematic reviews were screened to search for further potentially relevant studies. No language restrictions or publication types were applied. The last search was run in November 2018. Search syntaxes and PRISMA flow charts of record selection are provided as supplementary files.

We considered meta-analyses of randomized controlled trials (RCTs), meta-analyses of cohort studies, or individual RCTs and cohort studies within similar PICO frameworks to those of the predefined questions. An initial structured search aimed to identify meta-analyses. If a relevant meta-analysis published from 2016 onwards was identified, it was further screened against predefined PICO criteria. In the absence of a recent meta-analysis on a subject, a structured search was designed to identify RCTs and/or cohort studies.

Overarching inclusion criteria across PICO questions were adult patients (age>18 years) with body mass index >35kg/m^2^ (unless otherwise defined) and laparoscopic surgery (in relevant topic domains). Animal studies, studies on pediatric patients and studies on robotic or open surgery were discarded.

A total of 65 systematic reviews were performed and the de-duplicated search results were uploaded on the electronic platform Rayyan, which facilitates record screening and article selection.^7^ Two reviewers independently screened records for inclusion and any disagreements were resolved by discussion. The senior panel member acted as adjudicator, if needed.

*Evidence synthesis*

In the presence of a recent meta-analysis, summary effect measures and interval estimates, and risk of bias parameters were considered to assess the quality of evidence as per GRADE methodology.^1,8^ If no recent meta-analysis was available, we searched for relevant RCTs and/or cohort studies, we extracted raw data in ad hoc datasheets and we assessed the risk of bias using the Cochrane Tool for RCTs and the ROBINS I tool for cohort studies.^9,10^ In the presence of both RCTs and cohort studieson a PICO question, we assessed the quality of evidence separetely for the summary measures of RCTs and of cohort studies and we further considered the highest-level quality. Data of interest were extracted from the abstract, text, tables, figures and/or supplementary material. Only published data were considered. No attempt was made to contact study authors to request missing data.

We performed pairwise meta-analysis using the Mantel-Haenszel method and a fixed-effect model. In the presence of conceptual and/or statistical heterogeneity we performed random-effects meta-analysis according to DerSimonian and Laird.^11^ We calculated odds ratios and their 95% confidence intervals for dichotomous variables. In case of one or more studies reporting zero events in both arms, we calculated the risk difference and respective 95% interval estimates instead. For continuous variables, we calculated the weighted mean difference and 95% confidence intervals. If different scales were used across studies, we calculated the standardized mean difference instead. Publication bias was assessed by means of inspecting funnel plots, if 10 or more studies were available for analysis.^12^ Inconsistency was quantified by calculating the t^2^, the χ^2^ and respective p-value. Statistical heterogeneity was measured by calculaton of the I^2^-value. I^2^-values between 0-40% suggested not important, 30%-60% moderate, 50%-90% substantial and 75%-100% considerable heterogeneity.^12^ Forest plots and funnel plots (where available) can be provided by the authors upon reasonable request. We generated evidence tables, summarizing judgements on study design, risk of bias, inconsistency, indirectness, imprecision and the overall quality of evidence on each outcome of interest.^13,14^

For adjustable gastric banding, we performed proportion meta-analysis of related complications and reoperations after systematic review of RCTs and observational studies using a random-effects model to account for statistical (if any) and conceptual heterogeneity, in view of different study designs, procedural steps and methods/duration of follow-up. Results are presented as summary proportions and 95% confidence intervals. Individual outcomes of studies reporting follow-up of at least 5 years are presented separately.

A second face-to-face meeting took place in Rome, on June 26^th^ 2019, to discuss evidence summaries and plan the next steps.

*Evidence-to-decision framework*

Predefined parameters were taken into account to formulate recommendations. More specifically, importance of the problem, desirable/undesirable effects and their balance, the certainty (quality) of evidence, patient values and preferences, acceptability to key stakeholders, cost of implementation and feasibility of incorporating the intervention into practice were assessed.^15^ Under consideration of these parameters, the GDG provided:

- - a strong recommendation for the intervention or the comparator
  - a conditional recommendation for the intervention or the comparator, or
  - no recommendation (conditional recommendation for either the intervention or the comparator)^15^

If no recommendation could be formulated on a PICO question, the GDG had the option to draft a position statement. Position statements reflect the opinion of the GDG, are not necessarily based on available research evidence and should not be considered formal recommendations.

We used the GRADEpro GDT software for generation of evidence tables and the development of recommendations.^16^

*Delphi process*

The recommendation drafts, along with background evidence and judgements on the above parameters, were subjected to a web-based Delphi process. In the scenario of unanimous agreement among panel members, the recommendation was approved. In case of disagreement, comments from panel members were taken into account, recommendations were re-formulated and they were subjected to a second Delphi round. Three Delphi rounds took place overall. A third face-to-face meeting took place in Rome, on May 11^th^ 2019, to discuss the evidence-to-decision frameworks.

*Survey*

Members of participating societies were surveyed to investigate the applicability of recommendation on their practice. More specifically, each recommendation and practice statement were followed by the question: ‘Does this recommendation/practice statement apply to your practice?’ with 3 possible responses (Yes/No/I don’t know). The survey instrument was developed using a web-based platform (SurveyMonkey**^®^**). Individuals were sent email invitations and a reminder email to participate in the survey though a dedicated web link. The survey was also distributed via the EAES social media channels (Facebook Inc., Twitter Inc.) Further, attendees of the 27^th^ International Congress of EAES were invited to participate in an on-site survey using a smartphone application (networkapp**^®^**), in a dedicated session.

*Appraisal*

The full guideline was reviewed by 3 prominent obesity surgeons from Europe and USA and was appraised using the AGREE II tool.

**REFERENCES**

1. Schünemann H, Brożek J, Guyatt G, Oxman A. GRADE handbook for grading quality of evidence and strength of recommendations. The GRADE Working Group.

2. Brouwers MC, Kho ME, Browman GP, et al. AGREE II: Advancing guideline development, reporting and evaluation in health care. *J Clin Epidemiol*. 2010;63(12):1308-1311. doi:10.1016/j.jclinepi.2010.07.001

3. Liberati A, Altman DG, Tetzlaff J, et al. The PRISMA statement for reporting systematic reviews and meta-analyses of studies that evaluate health care interventions: explanation and elaboration. *J Clin Epidemiol*. 2009. doi:10.1016/j.jclinepi.2009.06.006

4. Stroup DF, Berlin JA, Morton SC, Olkin I, Williamson GD RD. MOOSE Guidelines for Meta-Analyses and Systematic Reviews of Observational Studies. *Jama*. 2000.

5. Norris SL, Meerpohl JJ, Akl EA, Schünemann HJ, Chen Y, Whittington C. The skills and experience of GRADE methodologists can be assessed with a simple tool. *J Clin Epidemiol*. 2016. doi:10.1016/j.jclinepi.2016.07.001

6. Sauerland S, Angrisani L, Belachew M, et al. Obesity surgery: Evidence-based guidelines of the European Association for Endoscopic Surgery (E.A.E.S.). *Surg Endosc Other Interv Tech*. 2005. doi:10.1007/s00464-004-9194-1

7. Ouzzani M, Hammady H, Fedorowicz Z, Elmagarmid A. Rayyan-a web and mobile app for systematic reviews. *Syst Rev*. 2016. doi:10.1186/s13643-016-0384-4

8. Akl EA, Lang D, Jaeschke R, et al. Grading quality of evidence and strength of recommendations in clinical practice guidelines Part 1 of 3 . An overview of the GRADE approach and grading quality of evidence about interventions. 2009;(November 2008):669-677. doi:10.1111/j.1398-9995.2009.01973.x

9. Higgins JPT, Altman DG, Gøtzsche PC, et al. The Cochrane Collaboration’s tool for assessing risk of bias in randomised trials. *BMJ*. 2011. doi:10.1136/bmj.d5928

10. Sterne J, Hernán M, Reeves B, et al. Risk Of Bias In Non-randomized Studies of Interventions ( ROBINS-I ): detailed guidance. *Bmj*. 2016.

11. DerSimonian R, Laird N. Meta-analysis in clinical trials. *Control Clin Trials*. 1986. doi:10.1016/0197-2456(86)90046-2

12. Higgins J, Green S. Cochrane Handbook for Systematic Reviews of Interventions Version 5.1.0 [updated March 2011]. *Cochrane Collab*. 2011.

13. Schünemann HJ, Brozek J, Guyatt GH, Oxman AD. 4. Summarizing the evidence. GRADE Handbook. https://gdt.gradepro.org/app/handbook/handbook.html. Published 2013.

14. Schünemann HJ, Brozek J, Guyatt GH, Oxman AD. 5. Quality of evidence. GRADE Handbook. https://gdt.gradepro.org/app/handbook/handbook.html. Published 2013.

15. Schünemann HJ, Brozek J, Guyatt GH, Oxman AD. 6. Going from evidence to recommendations. GRADE Handbook. https://gdt.gradepro.org/app/handbook/handbook.html. Published 2013.

16. GRADE Working Group. GRADEpro guideline development tool [software]. *McMaster Univ*. 2015.
