# Supplementary material for: Clinical practice guidelines of the European Association for Endoscopic Surgery (EAES) on bariatric surgery: update 2020 endorsed by IFSO-EC, EASO and ESPCOP
Source: Surg Endosc. 2020 Apr 23;34(6):2332–58. doi: 10.1007/s00464-020-07555-y (PMC7214495; doi:10.1007/s00464-020-07555-y)
Supplement: Supplementary file 2 — Supplementary file2 (DOCX 22 kb) [file 464_2020_7555_MOESM2_ESM.docx]

**Supplementary file 2**

**PICO QUESTIONS**

**Topic 1 Indication to surgery**

- Should bariatric surgery vs. non surgical management be used for weight loss?

**Topic 2 Preoperative workup**

- Should preoperative *H. pylori* eradication vs. standard care be used for prevention of foregut symptoms in patients undergoing bariatric surgery?
- Should preoperative diet consultation vs. standard care be used for weight loss in patients undergoing bariatric surgery?
- Should assessment of pre-operative psychological conditions vs. no assesment be used in bariatric surgery prior to operation?
- Should preoperative esophagogastroscopy vs. no endoscopy be used for preoperative evaluation in patients undergoing bariatric surgery?

**Topic 3 Perioperative management**

- Should perioperative obstructive sleep apnea screening vs. no screening be used for prevention of respiratory complications after bariatric surgery?
- Should perioperative continuous positive airway pressure (CPAP) vs. no CPAP be used for prevention of respiratory complications after bariatric surgery?
- Should high dose pharmacological antithrombotic prophylaxis vs. standard dose antithrombotic prophylaxis be used for prevention of of thromboembolism in patients undergoing bariatric surgery?
- Should inferior vena cava filter vs. low molecular weight heparine and intermittent pneumatic compression be used for prevention of of thromboembolism in patients undergoing bariatric surgery?
- Should an Enhanced Recovery After Surgery protocol vs. standard care be used for patients undergoing bariatric surgery?
- Should multimodal analgesia with minimal use of opioids vs. standard analgesia be used for pain control in patients after bariatric surgery?

**Topic 4. Bariatric procedures**

***One anastomosis procedures***

- Should one-anastomosis gastric bypass (OAGB) vs. gastric plication be used for weight loss?
- Should OAGB vs. sleeve gastrectomy be used for weight loss?
- Should OAGB vs. Roux-en-Y gastric bypass (RYGB) be used for weight loss?
- Should OAGB vs. adjustable gastric banding (AGB) be used for weight loss?
- Should single-anastomosis duodeno-ileal switch (SADI-S) vs. sleeve gastrectomy be used for weight loss?
- Should SADI-S vs. biliopancreatic diversion with duodenal switch (BPD/DS) be used for weight loss?
- Should SADI-S vs. RYGB be used for weight loss?

***BPD***

- Should BPD/DS vs. RYGB be used for weight loss?
- Should BPD/DS vs. sleeve gastrectomy be used for weight loss?

***RYGB***

- Should RYGB vs. gastric plication be used for weight loss in obese patients?
- Should RYGB vs. AGB be used for weight loss in obese patients?
- Should RYGB vs. sleeve gastrectomy be used for weight loss in obese patients?

***Sleeve gastrectomy***

- Should sleeve gastrectomy vs. LAGB be used for weight loss?
- Should sleeve gastrectomy vs. gastric plication be used for weight loss?
- Should sleeve gastrectomy with antral resection (2-3 cm) vs. antral preservation (>5 cm) be used for weight loss?
- Should sleeve gastrectomy calibrated on bougie size ≤36 Fr vs. >36 Fr be used for weight loss?
- Should staple line reinforcement vs. no staple line reinforcement be used in patients undergoing sleeve gastrectomy?

**Topic 5 Revisional surgery**

- Should revisional surgery vs. no revisional surgery be used for failed bariatric surgery?

**Topic 6 Postoperative care**

- Should scheduled post-operative follow up vs. no post-operative follow up be used in patients undergoing bariatric surgery?
- Should treatment with ursodeoxycolic acid vs. no treatment with ursodeoxycolic acid be used for prevention of gallstone disease after bariatri surgery?
- Should nutritional (micro and/or macronutrients) supplementation vs. no supplementation be used in in patients undergoing bariatric surgery?
- Should proton pump inhibitor (PPI) treatment vs. no PPI treatment be used in patients undergoing bariatric surgery?
- Should postoperative diet vs. no postoperative diet be used in patients undergoing bariatric surgery?
- Should delayed pregnancy vs. early pregnancy be used for prevention of fetal complications?

**Topic 7 Investigational procedures**

- Should EndoBarrier® vs. non-surgical management be used for weight loss?
- Should AspireAssist® vs. non-surgical management be used for weight loss?
- Should abiliti® vs. non-surgical management be used for weight loss?
- Should vBloc® vs. non-surgical management be used for weight loss?
- Should Pose® procedure vs. non-surgical management be used for weight loss?
- Should OverStitch™ vs. non-surgical management be used for weight loss?
- Should Duodenal Mucosal Resurfacing vs. non-surgical management be used for weight loss?
