# Supplementary material for: Clinical practice guidelines of the European Association for Endoscopic Surgery (EAES) on bariatric surgery: update 2020 endorsed by IFSO-EC, EASO and ESPCOP
Source: Surg Endosc. 2020 Apr 23;34(6):2332–58. doi: 10.1007/s00464-020-07555-y (PMC7214495; doi:10.1007/s00464-020-07555-y)
Supplement: Supplementary file 3 — Supplementary file3 (DOCX 37 kb) [file 464_2020_7555_MOESM3_ESM.docx]

EAES guideline searches summary

Contents

[Number of studies found per Topic (3,699) 3](#_Toc23855513)

[Number of PICOs and Questions per Topic (93) 3](#_Toc23855514)

[Topic 1: Indication for bariatric/metabolic surgery 3](#_Toc23855515)

[PICO 3](#_Toc23855516)

[PubMed (5 November 2018) 3](#_Toc23855517)

[Embase.com (5 November 2018) 3](#_Toc23855518)

[Number of studies (110) 3](#_Toc23855519)

[Topic 2: Pre-operative workup and assessment of co-morbidities by multidisciplinary team 3](#_Toc23855520)

[PICOs 3](#_Toc23855521)

[PubMed (30 October 2018 + 20 February 2019) 4](#_Toc23855522)

[Embase.com (30 October + 10 December 2018 + 20 February 2019) 4](#_Toc23855523)

[Wiley/Cochrane Library CENTRAL (30 October 2018) 4](#_Toc23855524)

[Number of studies found per question (531) 4](#_Toc23855525)

[Topic 3: Perioperative management 4](#_Toc23855526)

[PICOs 4](#_Toc23855527)

[PubMed (9 November 2018 + 11 February 2019) 5](#_Toc23855528)

[Embase.com (9 November 2018 + 11 February 2019) 5](#_Toc23855529)

[Number of studies found per question (566) 5](#_Toc23855530)

[Topic 4: Primary bariatric surgery 6](#_Toc23855531)

[PICOs 6](#_Toc23855532)

[Categories of bariatric surgery 6](#_Toc23855533)

[PubMed (21 November 2018) 7](#_Toc23855534)

[Embase.com (22 November 2018) 7](#_Toc23855535)

[Number of studies found per question (1104) 7](#_Toc23855536)

[Topic 5: Revisional surgery 7](#_Toc23855537)

[Reorganized PICOs 7](#_Toc23855538)

[PubMed (14 November 2018) 9](#_Toc23855539)

[Embase.com (16 November 2018) 10](#_Toc23855540)

[Number of studies found per question (307) 10](#_Toc23855541)

[Topic 6: Postoperative Care 10](#_Toc23855542)

[PICOs 10](#_Toc23855543)

[PubMed (23 November 2018 + 11 February + 20 February 2019) 11](#_Toc23855544)

[Embase.com (23 November 2018 + 11 February + 20 February 2019) 11](#_Toc23855545)

[Number of studies found per question (756) 11](#_Toc23855546)

[Topic 7: Investigational procedures 11](#_Toc23855547)

[PICOs 11](#_Toc23855548)

[Embase.com (23 November 2018) 12](#_Toc23855549)

[Number of studies found per question (325) 12](#_Toc23855550)

# Number of studies found per Topic (3,699)

| T1 | **110** |
| --- | --- |
| T2 | **531** |
| T3 | **566** |
| T4 | **1,104** |
| T5 | **307** |
| T6 | **756** |
| T7 | **325** |
| Total | **3,699** |

# Number of PICOs and Questions per Topic (93)

| Topic | **# PICOs** | **# Questions** |
| --- | --- | --- |
| T1 | **1** | **1** |
| T2 | **5** | **4** |
| T3 | **10** | **7** |
| T4 | **17** | **7** |
| T5 | **36** | **10** |
| T6 | **9** | **6** |
| T7 | **15** | **17** |
| **Total** | **93** | **52** |

# Topic 1: Indication for bariatric/metabolic surgery

## PICO

1. Should [bariatric surgery] vs [medical therapy] be used for [weight loss] in [obese patients]?

## PubMed (5 November 2018)

- 39 SRs, 34 RCTs, 65 cohort studies

## Embase.com (5 November 2018)

- 89 SRs, 17 RCTs, 119 cohort studies

### Number of studies (110)

| T1 | **110** |
| --- | --- |

# Topic 2: Pre-operative workup and assessment of co-morbidities by multidisciplinary team

## PICOs

1. Should [preoperative diet consultation] vs [standard care] be used for [weight loss] in [patients undergoing bariatric surgery]?
2. Should [preoperative long-term diet consultation] vs [short term] be used for [weight loss] in [patients undergoing bariatric surgery]?
3. Should [smoking cessation] vs [no smoking cessation] be used for [prevention of pulmonary complications] in [patients undergoing bariatric surgery]?
4. Should [preoperative H. pylori be tested] vs [not tested] be used for [prevention of foregut symptoms] in [patients undergoing bariatric surgery]?
5. Should [preoperative psychological consultation] vs [no preoperative psychological consultation] be used in [patients undergoing bariatric surgery]?

## PubMed (30 October 2018 + 20 February 2019)

- T2Q1 Preoperative diet (12 SRs, 42 RCTs, 70 cohort studies) (30 October 2018)
- T2Q3 Smoking cessation (6) (30 October 2018)
- T2Q4 H. Pylori (66) (30 October 2018)
- T2Q6 Preoperative psychologicalconsult (4 SRs, 13 RCTs, 31 cohort studies) (20 February 2019)

## Embase.com (30 October + 10 December 2018 + 20 February 2019)

- T2Q1 Preoperative diet (21 SRs, 18 RCTs, 45 cohort studies) (30 October 2018)
- T2Q3 (12 SRs, 2 RCTs, 13 cohort studies) (10 December 2018)
- T2Q4 H. pylori (32 SRs, 6 RCTs, 60 cohort studies) (30 October 2018)
- T2Q6 Preoperative psychologicalconsult (60 SRs, 12 RCTs, 94 cohort studies) (20 February 2019)

## Wiley/Cochrane Library CENTRAL (30 October 2018)

- T2Q1 Diet (20) (30 October 2018)
- T2Q3 Smoking cessation (2) (30 October 2018)
- T2Q4 H. pylori (4) (30 October 2018)

### Number of studies found per question (531)

| T2Q1&2 | **194** |
| --- | --- |
| T2Q3 | **31** |
| T2Q4 | **86** |
| T2Q6 | **220** |
| **Total** | **531** |

# Topic 3: Perioperative management

## PICOs

1. Should [pre or peri-operative CPAP] vs [standard care] be used for [prevention of respiratory complications] in [patients with OSA [obstructive sleep apnea] undergoing bariatric surgery]?
2. Should [(multimodal) analgesia with or without (minimal use of) opioids] vs [standard analgesia] be used for [pain control] in [patients undergoing bariatric surgery]?
3. Should [antibiotic prophylaxis] vs [no prophylaxis] be used for [prevention of infectious complications] in [patients undergoing bariatric surgery]?
4. Should [postoperative intermittent pneumatic compression (IPC) and pharmacological prophylaxis] vs [pharmacological prophylaxis alone] be used for [prevention of thromboembolism] in [patients undergoing bariatric surgery]?
5. Should [low molecular weight heparin (LMWH)] vs [low dose unfractionated heparin (LDUH)] be used for [prevention of thromboembolism] in [patients undergoing bariatric surgery]?
6. Should [high dose low molecular weight heparin (LMWH)] vs [standard dose unfractionated heparin (LDUH)] be used for [prevention of thromboembolism] in [patients undergoing bariatric surgery]?
7. Should [Administration of low molecular weight heparin (LMWH) beyond hospital stay] vs [in-hospital administration] be used for [prevention of thromboembolism] in [patients undergoing bariatric surgery]?
8. Should [inferior vena cava filter (IVCF)] vs [intermittent pneumatic compression] be used for [prevention of thromboembolism] in [high risk patients undergoing bariatric surgery]?
9. Should [ERAS protocol] vs [standard care] be used in [patients undergoing bariatric surgery]?

## PubMed (9 November 2018 + 11 February 2019)

- T3Q1: OSA + CPAP (7 SRs, 11 RCTs, 15 cohort studies) (9 November 2018)
- T3Q2: multimodal analgesia (6 SRs, 19 RCTs, 4 cohort studies) (9 November 2018)
- T3Q3: antibiotic prophylaxis (5 SRs, 6 RCTs, 9 cohort studies) (9 November 2018)
- T3Q4: intermittent pneumatic compression (IPC) (8 all study types) (9 November 2018)
- T3Q5+Q6+Q7: low molecular weight heparin (LMWH) (10 SRs, 8 RCTs, 13 cohort studies) (9 November 2018)
- T3 Q8+Q9: inferior vena cava filter (IVCF) (5 SRs, 3 RCTs, 11 cohort studies) (9 November 2018)
- T3Q10: ERAS (18 SRs, 39 RCTs, 38 cohort studies) (11 February 2019)

## Embase.com (9 November 2018 + 11 February 2019)

- T3Q1 OSA + CPAP (61 SRs, 15 RCTs, 71 cohort studies) (9 November 2018)
- T3Q2 multimodal analgesia (10 SRs, 11 RCTs, 7 cohort studies) (9 November 2018)
- T3Q3 antibiotic prophylaxis (57 SRs, 27 RCTs, 52 cohort studies) (9 November 2018)
- T3Q4 intermittent pneumatic compression (IPC) (18 all study types) (9 November 2018)
- T3Q5+Q6+Q7 low molecular weight heparin (LMWH) (97 SRs, 27 RCTs, 107 cohort studies) (9 November 2018)
- T3Q8+Q9 inferior vena cava filter (IVCF) (24 SRs, 5 RCTs, 18 cohort studies) (9 November 2018)
- T3Q10: ERAS (15 SRs, 15 RCTs, 18 cohort studies) (11 February 2019)

### Number of studies found per question (566)

| T3Q1 | **101** |
| --- | --- |
| T3Q2 | **86** |
| T3Q3 | **94** |
| T3Q4 | **17** |
| T3Q5-7 | **206** |
| T3Q8-9 | **38** |
| T3Q10 | **24** |
| **Total** | **566** |

# Topic 4: Primary bariatric surgery

## PICOs

1. Should [BPD/DS] vs [RYGB] be used for [weight loss] in [obese patients]?
2. Should [BPD/DS] vs [sleeve gastrectomy] be used for [weight loss] in [obese patients]?
3. Should [BPD/DS] vs [SADI-S] be used for [weight loss] in [obese patients]?
4. Should [1-stage BPD/DS] vs [2-stage BPD/DS] be used for [weight loss] in [obese patients]?
5. Should [RYGB] vs [LAGB] be used for [weight loss] in [obese patients]?
6. Should [RYGB] vs [sleeve gastrectomy] be used for [weight loss] in [obese patients]?
7. Should [RYGB] vs [gastric plication] be used for [weight loss] in [obese patients]?
8. Should [RYGB] vs [MGBP/OAGB] be used for [weight loss] in [obese patients]?
9. Should [LAGB] vs [sleeve gastrectomy] be used for [weight loss] in [obese patients]?
10. Should [LAGB] vs [gastric plication] be used for [weight loss] in [obese patients]?
11. Should [LAGB] vs [MGBP/OAGB] be used for [weight loss] in [obese patients]?
12. Should [sleeve gastrectomy] vs [gastric plication] be used for [weight loss] in [obese patients]?
13. Should [MGBP/OAGB] vs [sleeve gastrectomy] be used for [weight loss] in [obese patients]?
14. Should [sleeve gastrectomy with resection <6cm from the pylorus] vs [>6cm from the pylorus] be used for [weight loss] in [obese patients]? 🡪 Combine Q14-16
15. Should [sleeve gastrectomy with staple line reinforcement] vs [no reinforcement] be used for [weight loss] in [obese patients]?
16. Should [sleeve gastrectomy calibrated on bougie size <40Fr] vs [>40Fr] be used for [weight loss] in [obese patients]?
17. Should [gastric plication] vs [MGBP/OAGB] be used for [weight loss] in [obese patients]?

### Categories of bariatric surgery

I used eight categories (a-h) of bariatric surgery (see below) and compared each category with all others, starting with smaller subjects. I deduplicated every category with previous searches.

|  | 1. **Plica­tion** | 1. **SADIS or DS** | 1. **BPD** | 1. **MGB** | 1. **Ban­ding** | 1. **Sleeve** | 1. **Bypass** |
| --- | --- | --- | --- | --- | --- | --- | --- |
| 1. **Plica­tion** | *---* | **a** | **a** | **a** | **a** | **a** | **a** |
| 1. **SADIS or DS** | *---* | *---* | **b** | **b** | **b** | **b** | **b** |
| 1. **BPD** | *---* | *---* | *---* | **c** | **c** | **c** | **c** |
| 1. **MGB** | *---* | *---* | *---* | *---* | **d** | **d** | **d** |
| 1. **Banding** | *---* | *---* | *---* | *---* | *---* | **e** | **e** |
| 1. **Sleeve** | *---* | *---* | *---* | *---* | *---* | *---* | **f** |
| 1. **Bypass** | *---* | *---* | *---* | *---* | *---* | *---* | *---* |

## PubMed (21 November 2018)

- T4Qa: Gastric plication (21 SRs, 20 RCTs, 30 cohort studies)
- T4Qb: SADIS or DS (27 SRs, 25 RCTs, 56 cohort studies)
- T4Qc: BPD (43 SRs, 27 RCTs, 58 cohort studies)
- T4Qd: MGB (37 SRs, 32 RCTs, 93 cohort studies)
- T4Qe: Banding (133 SRs, 152 RCTs, 246 cohort studies)
- T4Qf: Sleeve (150 SRs, 162 RCTs, 284 cohort studies)
- T4Q14: Sleeve + resection, stapling, bougie (43 SRs, 58 RCTs, 86 cohort studies)

## Embase.com (22 November 2018)

- T4Qa: Gastric plication (18 SRs, 10 RCTs, 29 cohort studies)
- T4Qb: SADIS or DS (37 SRs, 6 RCTs, 55 cohort studies)
- T4Qc: BPD (42 SRs, 6 RCTs, 28 cohort studies)
- T4Qd: MGB (30 SRs, 22 RCTs, 77 cohort studies)
- T4Qe: Banding (146 SRs, 51 RCTs, 180 cohort studies)
- T4Qf: Sleeve (153 SRs, 78 RCTs, 226 cohort studies)
- T4Q14: Sleeve + resection, stapling, bougie (47 SRs, 42 RCTs, 94 cohort studies)

### Number of studies found per question (1104)

| T4Qa | **80** |
| --- | --- |
| T4Qb | **78** |
| T4Qc | **80** |
| T4Qd | **64** |
| T4Qe | **295** |
| T4Qf | **479** |
| T4Q14-16 | **28** |
| **Total** | **1104** |

# Topic 5: Revisional surgery

## Reorganized PICOs

- 1. **Resleeve**

1. Should [re-sleeve] vs [RYGB] be used for [weight loss] in [patients who regained weight after sleeve gastrectomy]?
2. Should [re-sleeve] vs [BPD/DS or SADI-S] be used for [weight loss] in [patients who regained weight after sleeve gastrectomy]?
3. Should [re-sleeve] vs [gastric banding] be used for [weight loss] in [patients who regained weight after sleeve gastrectomy]?
4. Should [re-sleeve gastrectomy] vs [limb length modification] be used for [weight loss] in [patients who regained weight after BPD/DS]?
   1. **RYGB**
5. Should [RYGB] vs [BPD/DS/SADI-S] be used for [weight loss] in [patients who regained weight after sleeve gastrectomy]?
6. Should [RYGB] vs [gastric banding] be used for [weight loss] in [patients who regained weight after sleeve gastrectomy]?
7. Should [RYGB] vs [BPD/DS or SADI-S] be used for [weight loss] in [patients who regained weight after failed gastric plication]?
8. Should [RYGB] vs [re-plication] be used for [weight loss] in [patients who regained weight after failed gastric plication]?
9. Should [RYGB] vs [BPD/DS or SADI-S] be used for [weight loss] in [patients who regained weight after failed gastric banding]?
10. Should [RYGB] vs [re-banding] be used for [weight loss] in [patients who regained weight after failed gastric banding]?
11. Should [RYGB] vs [MGBP/OAGB] be used for [weight loss] in [patients who regained weight after sleeve gastrectomy]?
    1. **BPD/DS or SADI-S**
12. Should [BPD/DS/SADI-S] vs [gastric banding] be used for [weight loss] in [patients who regained weight after sleeve gastrectomy]?
13. Should [BPD/DS or SADI-S] vs [re-plication] be used for [weight loss] in [patients who regained weight after failed gastric plication]?
14. Should [BPD/DS or SADI-S] vs [re-banding] be used for [weight loss] in [patients who regained weight after failed gastric banding]?
    1. **Sleeve gastrectomy**
15. Should [sleeve gastrectomy] vs [BPD/DS or SADI-S] be used for [weight loss] in [patients who regained weight after failed gastric plication]?
16. Should [sleeve gastrectomy] vs [RYGB] be used for [weight loss] in [patients who regained weight after failed gastric banding]?
17. Should [1-stage sleeve gastrectomy] vs [2-stages sleeve gastrectomy] be used for [weight loss] in [patients who regained weight after failed gastric banding]?
18. Should [sleeve gastrectomy] vs [RYGB] be used for [weight loss] in [patients who regained weight after failed gastric plication]?
19. Should [sleeve gastrectomy] vs [re-plication] be used for [weight loss] in [patients who regained weight after failed gastric plication]?
    1. **Limb lengthening**
20. Should [limb lengthening] vs [gastric bypass banding] be used for [weight loss] in [patients who regained weight after RYGB]?
21. Should [limb lengthening] vs [pouch resizing] be used for [weight loss] in [patients who regained weight after RYGB]?
22. Should [limb lengthening] vs [BPD/DS or SADI-S] be used for [weight loss] in [patients who regained weight after RYGB]?
23. Should [lengthening the biliopancreatic limb] vs [gastric bypass banding] be used for [weight loss] in [patients who regained weight after OAGB]?
24. Should [limb lengthening] vs [pouch resizing] be used for [weight loss] in [patients who regained weight after OAGB]?
25. Should [limb lengthening] vs [BPD/DS or SADI-S] be used for [weight loss] in [patients who regained weight after RYGB]?
    1. **Gastric bypass banding**
26. Should [gastric bypass banding] vs [BPD/DS or SADI-S] be used for [weight loss] in [patients who regained weight after RYGB]?
27. Should [gastric bypass banding] vs [pouch resizing] be used for [weight loss] in [patients who regained weight after RYGB]?
28. Should [gastric bypass banding] vs [pouch resizing] be used for [weight loss] in [patients who regained weight after RYGB]?
29. Should [gastric bypass banding] vs [BPD/DS or SADI-S] be used for [weight loss] in [patients who regained weight after RYGB]?
    1. **Pouch resizing**
30. Should [pouch resizing] vs [BPD/DS or SADI-S] be used for [weight loss] in [patients who regained weight after RYGB]?
31. Should [pouch resizing] vs [BPD/DS or SADI-S] be used for [weight loss] in [patients who regained weight after RYGB]?
    1. **Alimentary limb**
32. Should [extending the alimentary limb] vs [lengthening the biliopancreatic limb] be used for [weight loss] in [patients who regained weight after RYGB]?
    1. **Watchful waiting**
33. Should [watchful waiting] vs [operative treatment] be used for [functional/morphological obstruction] in [patients who had revisional bariatric surgery]?
    1. **Hiatal hernia repair**
34. Should [hiatal hernia repair] vs [RYGB] be used for [reflux control] in [patients with GERD after sleeve gastrectomy]?
35. Should [hiatal hernia repair] vs [PPI treatment] be used for [reflux control] in [patients with GERD after sleeve gastrectomy]?
36. Should [hiatal hernia repair] vs [redo RYGB] be used for [reflux control] in [patients with GERD after OAGB]?

## PubMed (14 November 2018)

- T5Qa: Resleeve (30 all study types)
- T5Qb: RYGB (37 SRs, 18 RCTs, 72 cohort studies)
- T5Qc: BPD/DS or SADI-S (28 SRs, 14 RCTs, 67 cohort studies)
- T5Qd: Sleeve gastrectomy (20 SRs, 11 RCTs, 63 cohort studies)
- T5Qe: Limb lengthening (9 all study types)
- T5Qf: Gastric bypass banding (49 SRs, 24 RCTs, 117 cohort studies)
- T5Qg: Pouch resizing (33 SRs, 25 RCTs, 130 cohort studies)
- T5Qh: Alimentary limb (12 SRs, 23 RCTs, 50 cohort studies)
- T5Qi: Watchful waiting (3 all study types)
- T5Qj: Hiatal hernia repair (48 all study types)

## Embase.com (16 November 2018)

- T5Qa: Resleeve (12 SRs, 4 RCTs, 63 cohort studies)
- T5Qb: RYGB (56 SRs, 14 RCTs, 93 cohort studies)
- T5Qc: BPD/DS or SADI-S (44 SRs, 6 RCTs, 109 cohort studies)
- T5Qd: Sleeve gastrectomy (17 SRs, 4 RCTs, 54 cohort studies)
- T5Qe: Limb lengthening (10 all study types)
- T5Qf: Gastric bypass banding (30 SRs, 3 RCTs, 54 cohort studies)
- T5Qg: Pouch resizing (6 SRs, 6 RCTs, 27 cohort studies)
- T5Qh: Alimentary limb (32 SRs, 17 RCTs, 92 cohort studies)
- T5Qi: Watchful waiting (2 all study types)
- T5Qj: Hiatal hernia repair (48 SRs, 10 RCTs, 84 cohort studies)

### Number of studies found per question (307)

| T5Qa | **36** |
| --- | --- |
| T5Qb | **65** |
| T5Qc | **43** |
| T5Qd | **9** |
| T5Qe | **11** |
| T5Qf | **7** |
| T5Qg | **24** |
| T5Qh | **31** |
| T5Qi | **5** |
| T5Qj | **76** |
| **Total** | **307** |

# Topic 6: Postoperative Care

## PICOs

1. Should [micronutrient and/or macronutrient (protein) supplementation] vs [no supplementation] be used for [prevention of complications] in [patients post bariatric surgery]?
2. Should [ursodeoxycholic acid] vs [no supplementation] be used for [prevention of gallstone disease] in [patients post bariatric surgery]?
3. Should [pregnancy be delayed] vs [no delay] be used for [prevention of fetal complications] in [patients post bariatric surgery]?
4. Should [pregnancy be delayed until expected weight loss has been achieved] vs [earlier] be used for [prevention of complications] in [patients post bariatric surgery]?
5. Should [PPI therapy] vs [no PPI therapy] be used in [the post-operative period after bariatric surgery]?
6. Should [low dose PPI] vs [high dose PPI] be used in [the post-operative period after bariatric surgery]?
7. Should [long-term PPI therapy] vs [short-term PPI therapy] be used in [the post-operative period after bariatric surgery]?
8. Should [intensive postoperative control schedule] vs [non intensive postoperative control schedule] be used for [weight loss] in [patients submitted to bariatric surgery]?
9. Should [postoperative sequential diet regimen] vs [no sequential postoperative diet regimen] be used for [postoperative food tolerance] in [patients submitted to bariatric surgery]?

## PubMed (23 November 2018 + 11 February + 20 February 2019)

- T6Q1: Nutrients (36 SRs, 60 RCTs, 81 cohort studies) (23 November 2018)
- T6Q2: Ursodeoxycholic acid (18 all study types) (23 November 2018)
- T6Q3-4: Pregnancy (40 SRs, 14 RCTs, 43 cohort studies) (23 November 2018)
- T6Q5: PPI (29 SRs, 39 RCTs, 45 cohort studies) (11 February 2019)
- T6Q6: Postoperative control schedule (12 SRs, 62 RCTs, 59 cohort studies) (20 February 2019)
- T6Q7: Sequential diet regimen (30 SRs, 73 RCTs, 105 cohort studies) (20 February 2019)

## Embase.com (23 November 2018 + 11 February + 20 February 2019)

- T6Q1: Nutrients (67 SRs, 33 RCTs, 84 cohort studies) (23 November 2018)
- T6Q2: Ursodeoxycholic acid (39 SRs, 11 RCTs, 26 cohort studies) (23 November 2018)
- T6Q3-4: Pregnancy (58 SRs, 4 RCTs, 76 cohort studies) (23 November 2018)
- T6Q5: PPI (75 SRs, 33 RCTs, 127 cohort studies) (11 February 2019)
- T6Q6: Postoperative control schedule (33 SRs, 86 RCTs, 104 cohort studies) (20 February 2019)
- T6Q7: Sequential diet regimen (45 SRs, 47 RCTs, 57 cohort studies) (20 February 2019)

### Number of studies found per question (756)

| T6Q1 | **97** |
| --- | --- |
| T6Q2 | **53** |
| T6Q3 | **6** |
| T6Q5 | **86** |
| T6Q6 | **252** |
| T6Q7 | **262** |
| **Total** | **756** |

# Topic 7: Investigational procedures

## PICOs

|  | Duodenal-jejunal bypass liner/sleeve | “duodenal-jejunal bypass liner” OR “duodenal-jejunal bypass sleeve” OR endobarrier |
| --- | --- | --- |
|  |  | valentx |
|  | Transoral Endoscopic Vertical Gastroplasty (TOGA) | transoral endoscopic vertical gastroplast* OR toga system* |
|  | Primary obesity surgery endoluminal (POSE) | primary obesity surgery endoluminal* OR incisionless operating platform* |
|  | Full Sense Bariatric device | full sense bariatric device* |
|  | Transpyloric Shuttle (TPS) | transpyloric shuttle* OR trans pyloric shuttle* |
|  | Endoscopic sleeve gastroplasty | endoscopic sleeve gastroplast* OR overstitch |
|  | Transoral anterior-to-posterior greater curvature plication | “transoral anterior-to-posterior greater curvature plication” OR endomina |
|  | Duodenal mucosal resurfacing procedure (DMR) | duodenal mucosal resurfacing OR (balloon catheter*) AND ablati*) |
|  | Self-Assembling Magnets for endoscopy to create a dual-path enteral bypass | self-assembling magnet* OR selfassembling magnet* |
|  | Articular Circular Endoscopic stapling (ACE) | articular circular endoscopic stapl* |
|  | Endoscopic Aspiration Therapy (EAT) | endoscopic aspiration therap* OR aspireassist OR aspire assist OR aspiration therapy system* |
|  | Pacing of the stomach vagal nerve/ reversible intra-abdominal stomach vagal nerve blockade: VBloc therapy | (pacing AND vagal nerve*) OR vagal nerve block* OR vbloc* OR maestro rechargeable system* |
|  | Gastric electrical stimulation (GES) | gastric electrical stimulat* OR exilis |
|  |  | abiliti system* |
|  |  | enterra system* |
|  | Selective vagal nerve stimulation | selective vagal nerve stimulat* OR transcend implantable gastric stimulat* |
|  | Gastric contractility modulation | gastric contractility modulat* OR tantalus |

## Embase.com (23 November 2018)

### Number of studies found per question (325)

| T7Q1 | **67** |
| --- | --- |
| T7Q2 | **6** |
| T7Q3 | **6** |
| T7Q4 | **34** |
| T7Q6 | **4** |
| T7Q7 | **31** |
| T7Q8 | **7** |
| T7Q9 | **21** |
| T7Q10 | **16** |
| T7Q11 | **5** |
| T7Q12 | **13** |
| T7Q13 | **37** |
| T7Q14 | **22** |
| T7Q15 | **9** |
| T7Q16 | **10** |
| T7Q17 | **1** |
| T7Q18 | **36** |
| **Total** | **325** |
