# Supplementary material for: Clinical practice guidelines of the European Association for Endoscopic Surgery (EAES) on bariatric surgery: update 2020 endorsed by IFSO-EC, EASO and ESPCOP
Source: Surg Endosc. 2020 Apr 23;34(6):2332–58. doi: 10.1007/s00464-020-07555-y (PMC7214495; doi:10.1007/s00464-020-07555-y)
Supplement: Supplementary file 4 — Supplementary file4 (DOCX 112 kb) [file 464_2020_7555_MOESM4_ESM.docx]

EAES guideline search strategies

Responsibility and execution of search strategies by Johannes C.F. Ket, medical information specialist, Medical Library, Vrije Universiteit Amsterdam, The Netherlands, [h.ket@vu.nl](mailto:h.ket@vu.nl)

Contents

[Topic 1: Indication for bariatric/metabolic surgery 5](#_Toc23853062)

[PubMed (5 November 2018) 5](#_Toc23853063)

[T1: 39 SRs, 34 RCTs, 65 cohort studies 5](#_Toc23853064)

[Embase.com (5 November 2018) 6](#_Toc23853065)

[T1: 89 SRs, 17 RCTs, 119 cohort studies 6](#_Toc23853066)

[Topic 2: Pre-operative workup and assessment of co-morbidities by multidisciplinary team 7](#_Toc23853067)

[PubMed (30 October 2018 + 20 February 2019) 7](#_Toc23853068)

[T2Q1: Preoperative diet (12 SRs, 42 RCTs, 70 cohort studies) (30 October 2018) 7](#_Toc23853069)

[T2Q3: Smoking cessation (6) (30 October 2018) 8](#_Toc23853070)

[T2Q4: H. Pylori (66) (30 October 2018) 8](#_Toc23853071)

[T2Q6: Preoperative psychological consult (4 SRs, 13 RCTs, 31 cohort studies) (20 February 2019) 9](#_Toc23853072)

[Embase.com (30 October + 10 December 2018 + 20 February 2019) 10](#_Toc23853073)

[T2Q1: Preoperative diet (21 SRs, 18 RCTs, 45 cohort studies) (30 October 2018) 10](#_Toc23853074)

[T2Q3: Smoking cessation (12 SRs, 2 RCTs, 13 cohort studies) (10 December 2018) 11](#_Toc23853075)

[T2Q4: H. pylori (32 SRs, 6 RCTs, 60 cohort studies) (30 October 2018) 12](#_Toc23853076)

[T2Q6: Preoperative psychological consult (60 SRs, 12 RCTs, 94 cohort studies) (20 February 2019) 13](#_Toc23853077)

[Wiley/Cochrane Library-CENTRAL (30 October 2018) 14](#_Toc23853078)

[T2Q1: Diet (20) 14](#_Toc23853079)

[T2Q3: Smoking cessation (2) 14](#_Toc23853080)

[T2Q4: H. pylori (4) 14](#_Toc23853081)

[Topic 3: Perioperative management 15](#_Toc23853082)

[PubMed (9 November 2018 + 11 February 2019) 15](#_Toc23853083)

[T3Q1: OSA + CPAP (7 SRs, 11 RCTs, 15 cohort studies) (9 November 2018) 15](#_Toc23853084)

[T3Q2: Multimodal analgesia (6 SRs, 19 RCTs, 4 cohort studies) (9 November 2018) 16](#_Toc23853085)

[T3Q3: Antibiotic prophylaxis (5 SRs, 6 RCTs, 9 cohort studies) (9 November 2018) 17](#_Toc23853086)

[T3Q4: Intermittent pneumatic compression (IPC) (8 all study types) 18](#_Toc23853087)

[T3Q5+Q6+Q7: Low molecular weight heparin (LMWH) (10 SRs, 8 RCTs, 13 cohort studies) 18](#_Toc23853088)

[T3 Q8+Q9: Inferior vena cava filter (IVCF) (5 SRs, 3 RCTs, 11 cohort studies) 19](#_Toc23853089)

[T3Q10: ERAS (18 SRs, 39 RCTs, 38 cohort studies) (11 February 2019) 20](#_Toc23853090)

[Embase.com (9 November 2018 + 11 February 2019) 21](#_Toc23853091)

[T3Q1: OSA + CPAP (61 SRs, 15 RCTs, 71 cohort studies) 21](#_Toc23853092)

[T3Q2: Multimodal analgesia (10 SRs, 11 RCTs, 7 cohort studies) 22](#_Toc23853093)

[T3Q3: Antibiotic prophylaxis (57 SRs, 27 RCTs, 52 cohort studies) 23](#_Toc23853094)

[T3Q4: Intermittent pneumatic compression (IPC) (18 all study types) 24](#_Toc23853095)

[T3Q5+Q6+Q7: Low molecular weight heparin (LMWH) (97 SRs, 27 RCTs, 107 cohort studies) 25](#_Toc23853096)

[T3Q8+Q9: Inferior vena cava filter (IVCF) (24 SRs, 5 RCTs, 18 cohort studies) 25](#_Toc23853097)

[T3Q10: ERAS (15 SRs, 15 RCTs, 18 cohort studies) (11 February 2019) 26](#_Toc23853098)

[Topic 4: Primary bariatric surgery 27](#_Toc23853099)

[PubMed (21 November 2018) 27](#_Toc23853100)

[T4Qa: Gastric plication (21 SRs, 20 RCTs, 30 cohort studies) 27](#_Toc23853101)

[T4Qb: SADIS or DS (27 SRs, 25 RCTs, 56 cohort studies) 28](#_Toc23853102)

[T4Qc: BPD (43 SRs, 27 RCTs, 58 cohort studies) 29](#_Toc23853103)

[T4Qd: MGB (37 SRs, 32 RCTs, 93 cohort studies) 30](#_Toc23853104)

[T4Qe: Banding (133 SRs, 152 RCTs, 246 cohort studies) 30](#_Toc23853105)

[T4Qf: Sleeve (150 SRs, 162 RCTs, 284 cohort studies) 31](#_Toc23853106)

[T4Q14: Sleeve + resection, stapling, bougie (43 SRs, 58 RCTs, 86 cohort studies) 32](#_Toc23853107)

[Embase.com (22 November 2018) 33](#_Toc23853108)

[T4Qa: Gastric plication (18 SRs, 10 RCTs, 29 cohort studies) 33](#_Toc23853109)

[T4Qb: SADIS or DS (37 SRs, 6 RCTs, 55 cohort studies) 34](#_Toc23853110)

[T4Qc: BPD (42 SRs, 6 RCTs, 28 cohort studies) 34](#_Toc23853111)

[T4Qd: MGB (30 SRs, 22 RCTs, 77 cohort studies) 35](#_Toc23853112)

[T4Qe: Banding (146 SRs, 51 RCTs, 180 cohort studies) 36](#_Toc23853113)

[T4Qf: Sleeve (153 SRs, 78 RCTs, 226 cohort studies) 37](#_Toc23853114)

[T4Q14: Sleeve + resection, stapling, bougie (47 SRs, 42 RCTs, 94 cohort studies) 37](#_Toc23853115)

[Topic 5: Revisional surgery 38](#_Toc23853116)

[PubMed (14 November 2018) 38](#_Toc23853117)

[T5Qa: Resleeve (30 all study types) 38](#_Toc23853118)

[T5Qb: RYGB (37 SRs, 18 RCTs, 72 cohort studies) 38](#_Toc23853119)

[T5Qc: BPD/DS or SADI-S (28 SRs, 14 RCTs, 67 cohort studies) 39](#_Toc23853120)

[T5Qd: Sleeve gastrectomy (20 SRs, 11 RCTs, 63 cohort studies) 40](#_Toc23853121)

[T5Qe: Limb lengthening (9 all study types) 40](#_Toc23853122)

[T5Qf: Gastric bypass banding (49 SRs, 24 RCTs, 117 cohort studies) 41](#_Toc23853123)

[T5Qg: Pouch resizing (33 SRs, 25 RCTs, 130 cohort studies) 42](#_Toc23853124)

[T5Qh: Alimentary limb (12 SRs, 23 RCTs, 50 cohort studies) 43](#_Toc23853125)

[T5Qi: Watchful waiting (3 all study types) 44](#_Toc23853126)

[T5Qj: Hiatal hernia repair (48 all study types) 44](#_Toc23853127)

[Embase.com (16 November 2018) 45](#_Toc23853128)

[T5Qa: Resleeve (12 SRs, 4 RCTs, 63 cohort studies) 45](#_Toc23853129)

[T5Qb: RYGB (56 SRs, 14 RCTs, 93 cohort studies) 46](#_Toc23853130)

[T5Qc: BPD/DS or SADI-S (44 SRs, 6 RCTs, 109 cohort studies) 46](#_Toc23853131)

[T5Qd: Sleeve gastrectomy (17 SRs, 4 RCTs, 54 cohort studies) 47](#_Toc23853132)

[T5Qe: Limb lengthening (10 all study types) 47](#_Toc23853133)

[T5Qf: Gastric bypass banding (30 SRs, 3 RCTs, 54 cohort studies) 48](#_Toc23853134)

[T5Qg: Pouch resizing (6 SRs, 6 RCTs, 27 cohort studies) 49](#_Toc23853135)

[T5Qh: Alimentary limb (32 SRs, 17 RCTs, 92 cohort studies) 50](#_Toc23853136)

[T5Qi: Watchful waiting (2 all study types) 50](#_Toc23853137)

[T5Qj: Hiatal hernia repair (48 SRs, 10 RCTs, 84 cohort studies) 51](#_Toc23853138)

[Topic 6: Postoperative Care 52](#_Toc23853139)

[PubMed (23 November 2018 + 11 February + 20 February 2019) 52](#_Toc23853140)

[T6Q1: Nutrients (36 SRs, 60 RCTs, 81 cohort studies) 52](#_Toc23853141)

[T6Q2: Ursodeoxycholic acid (18 all study types) 53](#_Toc23853142)

[T6Q3-4: Pregnancy (40 SRs, 14 RCTs, 43 cohort studies) 54](#_Toc23853143)

[T6Q5: PPI (29 SRs, 39 RCTs, 45 cohort studies) (11 February 2019) 55](#_Toc23853144)

[T6Q6: Postoperative control schedule (12 SRs, 62 RCTs, 59 cohort studies) (20 February 2019) 57](#_Toc23853145)

[T6Q7: Sequential diet regimen (30 SRs, 73 RCTs, 105 cohort studies) (20 February 2019) 58](#_Toc23853146)

[Embase.com (23 November 2018 + 11 February + 20 February 2019) 59](#_Toc23853147)

[T6Q1: Nutrients (67 SRs, 33 RCTs, 84 cohort studies) 59](#_Toc23853148)

[T6Q2: Ursodeoxycholic acid (39 SRs, 11 RCTs, 26 cohort studies) 60](#_Toc23853149)

[T6Q3-4: Pregnancy (58 SRs, 4 RCTs, 76 cohort studies) 60](#_Toc23853150)

[T6Q5: PPI (75 SRs, 33 RCTs, 127 cohort studies) (11 February 2019) 61](#_Toc23853151)

[T6Q6: Postoperative control schedule (33 SRs, 86 RCTs, 104 cohort studies) (20 February 2019) 62](#_Toc23853152)

[T6Q7: Sequential diet regimen (45 SRs, 47 RCTs, 57 cohort studies) (20 February 2019) 63](#_Toc23853153)

[Topic 7: Investigational procedures 64](#_Toc23853154)

[Embase.com (23 November 2018) 64](#_Toc23853155)

[T7: All questions and all results 64](#_Toc23853156)

# Topic 1: Indication for bariatric/metabolic surgery

## PubMed (5 November 2018)

### T1: 39 SRs, 34 RCTs, 65 cohort studies

| **Search** | **Query** | **Items found** |
| --- | --- | --- |
| [**#32**](https://www.ncbi.nlm.nih.gov/pubmed) | ((#26 AND #31) NOT (#28 OR #29)) | [**65**](https://www.ncbi.nlm.nih.gov/pubmed/?cmd=HistorySearch&querykey=32) |
| [**#31**](https://www.ncbi.nlm.nih.gov/pubmed) | "Epidemiologic Studies"[Mesh] OR "Prognosis"[Mesh] OR cohort[tiab] OR (case[tiab] AND (control[tiab] OR controll*[tiab] OR comparison[tiab] OR referent[tiab])) OR risk[tiab] OR causation[tiab] OR causal[tiab] OR "odds ratio"[tiab] OR etiol*[tiab] OR aetiol*[tiab] OR "natural history"[tiab] OR predict*[tiab] OR outcome[tiab] OR course[tiab] OR retrospect*[tiab] OR “follow up”[tiab] OR followup[tiab] OR prognos*[tiab] | [**6588338**](https://www.ncbi.nlm.nih.gov/pubmed/?cmd=HistorySearch&querykey=31) |
| [**#30**](https://www.ncbi.nlm.nih.gov/pubmed) | (#26 AND #29) NOT #28 | [**34**](https://www.ncbi.nlm.nih.gov/pubmed/?cmd=HistorySearch&querykey=30) |
| [**#29**](https://www.ncbi.nlm.nih.gov/pubmed) | "Randomized Controlled Trial"[Publication Type] OR "Controlled Clinical Trial"[Publication Type] OR random*[tiab] OR placebo[tiab] OR "Drug Therapy"[Subheading] OR trial[tiab] OR groups[tiab] | [**4562875**](https://www.ncbi.nlm.nih.gov/pubmed/?cmd=HistorySearch&querykey=29) |
| [**#28**](https://www.ncbi.nlm.nih.gov/pubmed) | (#26 AND #27) | [**39**](https://www.ncbi.nlm.nih.gov/pubmed/?cmd=HistorySearch&querykey=28) |
| [**#27**](https://www.ncbi.nlm.nih.gov/pubmed) | (review[tiab] OR "Review"[Publication Type] OR "Meta-Analysis as Topic"[Mesh] OR meta-analysis[tiab] OR "Meta-Analysis"[Publication Type]) NOT ("Letter"[Publication Type] OR "Editorial"[Publication Type] OR "Comment"[Publication Type]) | [**2988435**](https://www.ncbi.nlm.nih.gov/pubmed/?cmd=HistorySearch&querykey=27) |
| [**#26**](https://www.ncbi.nlm.nih.gov/pubmed) | #25 NOT (("Adolescent"[Mesh] OR "Child"[Mesh] OR "Infant"[Mesh] OR adolescen*[tiab] OR child*[tiab] OR schoolchild*[tiab] OR infant*[tiab] OR girl*[tiab] OR boy*[tiab] OR teen[tiab] OR teens[tiab] OR teenager*[tiab] OR youth*[tiab] OR pediatr*[tiab] OR paediatr*[tiab] OR puber*[tiab]) NOT ("Adult"[Mesh] OR adult*[tiab] OR man[tiab] OR men[tiab] OR woman[tiab] OR women[tiab])) | [**161**](https://www.ncbi.nlm.nih.gov/pubmed/?cmd=HistorySearch&querykey=26) |
| [**#25**](https://www.ncbi.nlm.nih.gov/pubmed) | #24 NOT ("Animals"[Mesh] NOT "Humans"[Mesh]) AND ("2004"[Date - Entrez] : "3000"[Date - Entrez]) | [**165**](https://www.ncbi.nlm.nih.gov/pubmed/?cmd=HistorySearch&querykey=25) |
| [**#24**](https://www.ncbi.nlm.nih.gov/pubmed) | (#22 AND #23) | [**188**](https://www.ncbi.nlm.nih.gov/pubmed/?cmd=HistorySearch&querykey=24) |
| [**#23**](https://www.ncbi.nlm.nih.gov/pubmed) | "Contraindications"[Majr] OR "Patient Selection"[Majr] OR "Risk Assessment"[Majr] OR indicati*[ti] OR contraindicati*[ti] OR selecti*[ti] OR recruit*[ti] OR criteri*[ti] OR indicati*[ot] OR contraindicati*[ot] OR selecti*[ot] OR recruit*[ot] OR criteri*[ot] | [**374252**](https://www.ncbi.nlm.nih.gov/pubmed/?cmd=HistorySearch&querykey=23) |
| [**#22**](https://www.ncbi.nlm.nih.gov/pubmed) | (("Bariatric Surgery"[Mesh] OR "Obesity/surgery"[Mesh:NoExp] OR "Obesity, Abdominal/surgery"[Mesh] OR "Obesity, Metabolically Benign/surgery"[Mesh] OR "Obesity, Morbid/surgery"[Mesh] OR "Anastomosis, Roux-en-Y"[Mesh] OR "Biliopancreatic Diversion"[Mesh] OR "Overweight/surgery"[Mesh] OR "Gastrectomy"[Mesh] OR bariatric surg*[tiab] OR bariatric operati*[tiab] OR bariatric procedure*[tiab] OR bilio pancreatic bypass*[tiab] OR bilio pancreatic diversion*[tiab] OR biliopancreatic bypass*[tiab] OR biliopancreatic diversion*[tiab] OR duodenal switch*[tiab] OR gastric band*[tiab] OR stomach band*[tiab] OR gastric bypass*[tiab] OR gastric partiti*[tiab] OR gastric plicati*[tiab] OR gastroplast*[tiab] OR mgbp*[tiab] OR oagb*[tiab] OR obese surg*[tiab] OR weight loss surg*[tiab] OR obesity surg*[tiab] OR metabolic surg*[tiab] OR roux en y*[tiab] OR “roux y”[tiab] OR “roux n y”[tiab] OR “roux in y”[tiab] OR rya[tiab] OR rygb*[tiab] OR sadis[tiab] OR “sadi s”[tiab] OR sagb*[tiab] OR sleeve gastrectom*[tiab] OR gastric sleeve*[tiab] OR gastrectom*[tiab] OR ((bariatric[ti] OR obes*[ti] OR weight loss[ti] OR metabolic[ti]) AND (surg*[ti] OR operati*[ti] OR procedure*[ti])) OR ((biliopancreatic[ti] OR “bilio pancreatic”[ti]) AND (diversion*[ti] OR bypass*[ti])) OR (duodenal[ti] AND switch*[ti]) OR ((stomach[ti] OR gastric[ti]) AND (band*[ti] OR bypass*[ti] OR partiti*[ti] OR plicati*[ti] OR sleeve*[ti])) OR (sleeve[ti] AND gastrectom*[ti]) OR (roux[ti] AND y[ti])) AND ("Laparoscopy"[Mesh:NoExp] OR "Minimally Invasive Surgical Procedures"[Mesh:NoExp] OR laparoscop*[tiab] OR minimally invasive[tiab] OR minimal invasive[tiab] OR minimal access[tiab] OR minimal surg*[tiab] OR (minimal*[ti] AND invasive[ti]) OR (minimal[ti] AND (access[ti] OR surg*[ti])))) OR (lagb*[tiab] OR lrygb*[tiab] OR lap band*[tiab] OR lrygb*[tiab]) | [**13041**](https://www.ncbi.nlm.nih.gov/pubmed/?cmd=HistorySearch&querykey=22) |

## Embase.com (5 November 2018)

### T1: 89 SRs, 17 RCTs, 119 cohort studies

| **No.** | **Query** | **Results** |
| --- | --- | --- |
| **#12** | #6 AND #11 NOT (#8 OR #9) | **119** |
| **#11** | 'epidemiology'/de OR (((cohort OR case) NEAR/3 (control OR controll* OR comparison OR referent)):ti,ab,kw) OR risk:ti,ab,kw OR causation:ti,ab,kw OR causal:ti,ab,kw OR 'odds ratio':ti,ab,kw OR etiol*:ti,ab,kw OR aetiol*:ti,ab,kw OR 'natural history':ti,ab,kw OR outcome:ti,ab,kw OR course:ti,ab,kw OR retrospect*:ti,ab,kw OR 'follow up':ti,ab,kw OR followup:ti,ab,kw OR predict*:ti,ab,kw OR prognos*:ti,ab,kw | **7303820** |
| **#10** | #6 AND #9 NOT #8 | **17** |
| **#9** | 'clinical study':ti,ab,kw AND 'trial':ti,ab,kw OR 'clinical trial'/exp OR random*:ti,ab,kw | **2189445** |
| **#8** | #6 AND #7 | **89** |
| **#7** | 'meta-analysis':ti,ab,kw OR 'meta analysis'/exp OR 'review'/exp OR review:ti,ab,kw | **3427657** |
| **#6** | #5 NOT (('adolescent'/exp OR 'child'/exp OR adolescent*:ti,ab OR child*:ti,ab OR schoolchild*:ti,ab OR infant*:ti,ab OR girl*:ti,ab OR boy*:ti,ab OR teen:ti,ab OR teens:ti,ab OR teenager*:ti,ab OR youth*:ti,ab OR pediatr*:ti,ab OR paediatr*:ti,ab OR puber*:ti,ab) NOT ('adult'/exp OR 'aged'/exp OR 'middle aged'/exp OR adult*:ti,ab OR man:ti,ab OR men:ti,ab OR woman:ti,ab OR women:ti,ab)) | **305** |
| **#5** | #4 NOT ('conference abstract'/it OR 'conference paper'/it OR 'letter'/it OR 'note'/it) | **310** |
| **#4** | #3 NOT ([animals]/lim NOT [humans]/lim) AND [1-1-2004]/sd | **461** |
| **#3** | #1 AND #2 | **545** |
| **#2** | 'contraindication'/exp/mj OR 'treatment indication'/exp/mj OR 'patient selection'/exp/mj OR 'risk assessment'/exp/mj OR indicati*:ti,kw OR contraindicati*:ti,kw OR selecti*:ti,kw OR recruit*:ti,kw OR criteri*:ti,kw | **477704** |
| **#1** | ('obesity'/exp/mj AND 'surgery'/lnk OR 'bariatric surgery'/exp/mj OR 'roux y anastomosis'/exp/mj OR 'gastric bypass surgery'/exp/mj OR ((('bilio pancreatic' OR biliopancreatic) NEAR/3 (diversion* OR bypass*)):ti,kw) OR ((duodenal NEAR/3 switch*):ti,kw) OR (((stomach OR gastric) NEAR/3 (band* OR bypass* OR partiti* OR plicati* OR sleeve*)):ti,kw) OR ((sleeve NEAR/3 gastrectom*):ti,kw) OR ((roux NEAR/2 y):ti,kw) OR gastroplast*:ti,kw OR mgbp*:ti,kw OR oagb*:ti,kw OR rya:ti,kw OR rygb*:ti,kw OR sadis:ti,kw OR 'sadi s':ti,kw OR (((bariatric OR obes* OR 'weight loss' OR metabolic) NEAR/3 (surg* OR operati* OR procedure*)):ti,kw) OR sagb*:ti,kw) AND ('laparoscopy'/de OR 'laparoendoscopic single site surgery'/exp OR 'laparoscopic surgery'/exp OR 'minimally invasive procedure'/exp OR 'minimally invasive surgery'/exp OR laparoscop*:ti,ab,kw OR ((minimal* NEAR/3 invasive):ti,ab,kw) OR access:ti,ab,kw OR surg*:ti,ab,kw) OR 'laparoscopic sleeve gastrectomy'/exp OR lagb*:ti,ab,kw OR 'lap band*':ti,ab,kw OR lrygb*:ti,ab,kw | **40167** |

# Topic 2: Pre-operative workup and assessment of co-morbidities by multidisciplinary team

## PubMed (30 October 2018 + 20 February 2019)

### T2Q1: Preoperative diet (12 SRs, 42 RCTs, 70 cohort studies) (30 October 2018)

| **Search** | **Query** | **Items found** |
| --- | --- | --- |
| [**#29**](https://www.ncbi.nlm.nih.gov/pubmed/advanced) | ((#23 AND #28) NOT (#26 OR #25)) | [**70**](https://www.ncbi.nlm.nih.gov/pubmed/?cmd=HistorySearch&querykey=29) |
| [**#28**](https://www.ncbi.nlm.nih.gov/pubmed/advanced) | ("Epidemiologic Studies"[Mesh] OR "Prognosis"[Mesh] OR cohort[tiab] OR (case[tiab] AND (control[tiab] OR controll*[tiab] OR comparison[tiab] OR referent[tiab])) OR risk[tiab] OR causation[tiab] OR causal[tiab] OR "odds ratio"[tiab] OR etiol*[tiab] OR aetiol*[tiab] OR "natural history"[tiab] OR predict*[tiab] OR outcome[tiab] OR course[tiab] OR retrospect*[tiab] OR “follow up”[tiab] OR followup[tiab] OR prognos*[tiab]) | [**6582812**](https://www.ncbi.nlm.nih.gov/pubmed/?cmd=HistorySearch&querykey=28) |
| [**#27**](https://www.ncbi.nlm.nih.gov/pubmed/advanced) | ((#23 AND #26) NOT #25) | [**42**](https://www.ncbi.nlm.nih.gov/pubmed/?cmd=HistorySearch&querykey=27) |
| [**#26**](https://www.ncbi.nlm.nih.gov/pubmed/advanced) | ("Randomized Controlled Trial"[Publication Type] OR "Controlled Clinical Trial"[Publication Type] OR random*[tiab] OR placebo[tiab] OR "Drug Therapy"[Subheading] OR trial[tiab] OR groups[tiab]) | [**4559743**](https://www.ncbi.nlm.nih.gov/pubmed/?cmd=HistorySearch&querykey=26) |
| [**#25**](https://www.ncbi.nlm.nih.gov/pubmed/advanced) | (#23 AND #24) | [**12**](https://www.ncbi.nlm.nih.gov/pubmed/?cmd=HistorySearch&querykey=25) |
| [**#23**](https://www.ncbi.nlm.nih.gov/pubmed/advanced) | (#22 NOT (("Adolescent"[Mesh] OR "Child"[Mesh] OR "Infant"[Mesh] OR adolescen*[tiab] OR child*[tiab] OR schoolchild*[tiab] OR infant*[tiab] OR girl*[tiab] OR boy*[tiab] OR teen[tiab] OR teens[tiab] OR teenager*[tiab] OR youth*[tiab] OR pediatr*[tiab] OR paediatr*[tiab] OR puber*[tiab]) NOT ("Adult"[Mesh] OR adult*[tiab] OR man[tiab] OR men[tiab] OR woman[tiab] OR women[tiab]))) | [**136**](https://www.ncbi.nlm.nih.gov/pubmed/?cmd=HistorySearch&querykey=23) |
| [**#24**](https://www.ncbi.nlm.nih.gov/pubmed/advanced) | (review[tiab] OR "Review"[Publication Type] OR "Meta-Analysis as Topic"[Mesh] OR meta-analysis[tiab] OR "Meta-Analysis"[Publication Type]) NOT ("Letter"[Publication Type] OR "Editorial"[Publication Type] OR "Comment"[Publication Type])) | [**2986282**](https://www.ncbi.nlm.nih.gov/pubmed/?cmd=HistorySearch&querykey=24) |
| [**#22**](https://www.ncbi.nlm.nih.gov/pubmed/advanced) | (#6 AND #21 NOT ("Animals"[Mesh] NOT "Humans"[Mesh]) AND ("2004"[Date - Entrez] : "3000"[Date - Entrez])) | [**140**](https://www.ncbi.nlm.nih.gov/pubmed/?cmd=HistorySearch&querykey=22) |
| [**#21**](https://www.ncbi.nlm.nih.gov/pubmed/advanced) | (("Diet Therapy"[Mesh] OR "diet therapy"[Subheading] OR "Diet, Reducing"[Mesh] OR diet[tiab] OR diets[tiab] OR dietary[tiab]) AND ("Preoperative Period"[Mesh] OR "Preoperative Care"[Mesh] OR pre operat*[tiab] OR preoperat*[tiab] OR before surg*[tiab])) | [**2385**](https://www.ncbi.nlm.nih.gov/pubmed/?cmd=HistorySearch&querykey=21) |
| [**#6**](https://www.ncbi.nlm.nih.gov/pubmed/advanced) | ((#3 AND #4) OR #5) | [**13022**](https://www.ncbi.nlm.nih.gov/pubmed/?cmd=HistorySearch&querykey=6) |
| [**#5**](https://www.ncbi.nlm.nih.gov/pubmed/advanced) | lagb*[tiab] OR lrygb*[tiab] OR lap band*[tiab] OR lrygb*[tiab] | [**2048**](https://www.ncbi.nlm.nih.gov/pubmed/?cmd=HistorySearch&querykey=5) |
| [**#4**](https://www.ncbi.nlm.nih.gov/pubmed/advanced) | "Laparoscopy"[Mesh:NoExp] OR "Minimally Invasive Surgical Procedures"[Mesh:NoExp] OR laparoscop*[tiab] OR minimally invasive[tiab] OR minimal invasive[tiab] OR minimal access[tiab] OR minimal surg*[tiab] OR (minimal*[ti] AND invasive[ti]) OR (minimal[ti] AND (access[ti] OR surg*[ti])) | [**183110**](https://www.ncbi.nlm.nih.gov/pubmed/?cmd=HistorySearch&querykey=4) |
| [**#3**](https://www.ncbi.nlm.nih.gov/pubmed/advanced) | "Bariatric Surgery"[Mesh] OR "Obesity/surgery"[Mesh:NoExp] OR "Obesity, Abdominal/surgery"[Mesh] OR "Obesity, Metabolically Benign/surgery"[Mesh] OR "Obesity, Morbid/surgery"[Mesh] OR "Anastomosis, Roux-en-Y"[Mesh] OR "Biliopancreatic Diversion"[Mesh] OR "Overweight/surgery"[Mesh] OR "Gastrectomy"[Mesh] OR bariatric surg*[tiab] OR bariatric operati*[tiab] OR bariatric procedure*[tiab] OR bilio pancreatic bypass*[tiab] OR bilio pancreatic diversion*[tiab] OR biliopancreatic bypass*[tiab] OR biliopancreatic diversion*[tiab] OR duodenal switch*[tiab] OR gastric band*[tiab] OR stomach band*[tiab] OR gastric bypass*[tiab] OR gastric partiti*[tiab] OR gastric plicati*[tiab] OR gastroplast*[tiab] OR mgbp*[tiab] OR oagb*[tiab] OR obese surg*[tiab] OR weight loss surg*[tiab] OR obesity surg*[tiab] OR metabolic surg*[tiab] OR roux en y*[tiab] OR “roux y”[tiab] OR “roux n y”[tiab] OR “roux in y”[tiab] OR rya[tiab] OR rygb*[tiab] OR sadis[tiab] OR “sadi s”[tiab] OR sagb*[tiab] OR sleeve gastrectom*[tiab] OR gastric sleeve*[tiab] OR gastrectom*[tiab] OR ((bariatric[ti] OR obes*[ti] OR weight loss[ti] OR metabolic[ti]) AND (surg*[ti] OR operati*[ti] OR procedure*[ti])) OR ((biliopancreatic[ti] OR “bilio pancreatic”[ti]) AND (diversion*[ti] OR bypass*[ti])) OR (duodenal[ti] AND switch*[ti]) OR ((stomach[ti] OR gastric[ti]) AND (band*[ti] OR bypass*[ti] OR partiti*[ti] OR plicati*[ti] OR sleeve*[ti])) OR (sleeve[ti] AND gastrectom*[ti]) OR (roux[ti] AND y[ti]) | [**76980**](https://www.ncbi.nlm.nih.gov/pubmed/?cmd=HistorySearch&querykey=3) |

### T2Q3: Smoking cessation (6) (30 October 2018)

| **Search** | **Query** | **Items found** |
| --- | --- | --- |
| [**#37**](https://www.ncbi.nlm.nih.gov/pubmed/advanced) | ((#6 AND #32) NOT ("Animals"[Mesh] NOT "Humans"[Mesh]) AND ("2004"[Date - Entrez] : "3000"[Date - Entrez])) | [**6**](https://www.ncbi.nlm.nih.gov/pubmed/?cmd=HistorySearch&querykey=37) |
| [**#32**](https://www.ncbi.nlm.nih.gov/pubmed/advanced) | "Smoking Cessation"[Mesh] OR smoking cessati*[tiab] OR stopping smoking*[tiab] OR giving up smoking*[tiab] OR quitting smoking*[tiab] | [**36185**](https://www.ncbi.nlm.nih.gov/pubmed/?cmd=HistorySearch&querykey=32) |
| [**#6**](https://www.ncbi.nlm.nih.gov/pubmed/advanced) | ((#3 AND #4) OR #5) | [**13022**](https://www.ncbi.nlm.nih.gov/pubmed/?cmd=HistorySearch&querykey=6) |
| [**#5**](https://www.ncbi.nlm.nih.gov/pubmed/advanced) | lagb*[tiab] OR lrygb*[tiab] OR lap band*[tiab] OR lrygb*[tiab] | [**2048**](https://www.ncbi.nlm.nih.gov/pubmed/?cmd=HistorySearch&querykey=5) |
| [**#4**](https://www.ncbi.nlm.nih.gov/pubmed/advanced) | "Laparoscopy"[Mesh:NoExp] OR "Minimally Invasive Surgical Procedures"[Mesh:NoExp] OR laparoscop*[tiab] OR minimally invasive[tiab] OR minimal invasive[tiab] OR minimal access[tiab] OR minimal surg*[tiab] OR (minimal*[ti] AND invasive[ti]) OR (minimal[ti] AND (access[ti] OR surg*[ti])) | [**183110**](https://www.ncbi.nlm.nih.gov/pubmed/?cmd=HistorySearch&querykey=4) |
| [**#3**](https://www.ncbi.nlm.nih.gov/pubmed/advanced) | "Bariatric Surgery"[Mesh] OR "Obesity/surgery"[Mesh:NoExp] OR "Obesity, Abdominal/surgery"[Mesh] OR "Obesity, Metabolically Benign/surgery"[Mesh] OR "Obesity, Morbid/surgery"[Mesh] OR "Anastomosis, Roux-en-Y"[Mesh] OR "Biliopancreatic Diversion"[Mesh] OR "Overweight/surgery"[Mesh] OR "Gastrectomy"[Mesh] OR bariatric surg*[tiab] OR bariatric operati*[tiab] OR bariatric procedure*[tiab] OR bilio pancreatic bypass*[tiab] OR bilio pancreatic diversion*[tiab] OR biliopancreatic bypass*[tiab] OR biliopancreatic diversion*[tiab] OR duodenal switch*[tiab] OR gastric band*[tiab] OR stomach band*[tiab] OR gastric bypass*[tiab] OR gastric partiti*[tiab] OR gastric plicati*[tiab] OR gastroplast*[tiab] OR mgbp*[tiab] OR oagb*[tiab] OR obese surg*[tiab] OR weight loss surg*[tiab] OR obesity surg*[tiab] OR metabolic surg*[tiab] OR roux en y*[tiab] OR “roux y”[tiab] OR “roux n y”[tiab] OR “roux in y”[tiab] OR rya[tiab] OR rygb*[tiab] OR sadis[tiab] OR “sadi s”[tiab] OR sagb*[tiab] OR sleeve gastrectom*[tiab] OR gastric sleeve*[tiab] OR gastrectom*[tiab] OR ((bariatric[ti] OR obes*[ti] OR weight loss[ti] OR metabolic[ti]) AND (surg*[ti] OR operati*[ti] OR procedure*[ti])) OR ((biliopancreatic[ti] OR “bilio pancreatic”[ti]) AND (diversion*[ti] OR bypass*[ti])) OR (duodenal[ti] AND switch*[ti]) OR ((stomach[ti] OR gastric[ti]) AND (band*[ti] OR bypass*[ti] OR partiti*[ti] OR plicati*[ti] OR sleeve*[ti])) OR (sleeve[ti] AND gastrectom*[ti]) OR (roux[ti] AND y[ti]) | [**76980**](https://www.ncbi.nlm.nih.gov/pubmed/?cmd=HistorySearch&querykey=3) |

### T2Q4: H. Pylori (66) (30 October 2018)

| **Search** | **Query** | **Items found** |
| --- | --- | --- |
| [**#41**](https://www.ncbi.nlm.nih.gov/pubmed/advanced) | (#40 AND (#24 OR #26 OR #28)) | [**66**](https://www.ncbi.nlm.nih.gov/pubmed/?cmd=HistorySearch&querykey=41) |
| [**#40**](https://www.ncbi.nlm.nih.gov/pubmed/advanced) | (#39 NOT (("Adolescent"[Mesh] OR "Child"[Mesh] OR "Infant"[Mesh] OR adolescen*[tiab] OR child*[tiab] OR schoolchild*[tiab] OR infant*[tiab] OR girl*[tiab] OR boy*[tiab] OR teen[tiab] OR teens[tiab] OR teenager*[tiab] OR youth*[tiab] OR pediatr*[tiab] OR paediatr*[tiab] OR puber*[tiab]) NOT ("Adult"[Mesh] OR adult*[tiab] OR man[tiab] OR men[tiab] OR woman[tiab] OR women[tiab])))) | [**78**](https://www.ncbi.nlm.nih.gov/pubmed/?cmd=HistorySearch&querykey=40) |
| [**#39**](https://www.ncbi.nlm.nih.gov/pubmed/advanced) | ((#6 AND #36) NOT ("Animals"[Mesh] NOT "Humans"[Mesh]) AND ("2004"[Date - Entrez] : "3000"[Date - Entrez])) | [**78**](https://www.ncbi.nlm.nih.gov/pubmed/?cmd=HistorySearch&querykey=39) |
| [**#36**](https://www.ncbi.nlm.nih.gov/pubmed/advanced) | ("Helicobacter pylori"[Mesh] OR helicobacter pylori*[tiab] OR h pylori*[tiab]) | [**42640**](https://www.ncbi.nlm.nih.gov/pubmed/?cmd=HistorySearch&querykey=36) |
| [**#28**](https://www.ncbi.nlm.nih.gov/pubmed/advanced) | ("Epidemiologic Studies"[Mesh] OR "Prognosis"[Mesh] OR cohort[tiab] OR (case[tiab] AND (control[tiab] OR controll*[tiab] OR comparison[tiab] OR referent[tiab])) OR risk[tiab] OR causation[tiab] OR causal[tiab] OR "odds ratio"[tiab] OR etiol*[tiab] OR aetiol*[tiab] OR "natural history"[tiab] OR predict*[tiab] OR outcome[tiab] OR course[tiab] OR retrospect*[tiab] OR “follow up”[tiab] OR followup[tiab] OR prognos*[tiab]) | [**6582812**](https://www.ncbi.nlm.nih.gov/pubmed/?cmd=HistorySearch&querykey=28) |
| [**#26**](https://www.ncbi.nlm.nih.gov/pubmed/advanced) | ("Randomized Controlled Trial"[Publication Type] OR "Controlled Clinical Trial"[Publication Type] OR random*[tiab] OR placebo[tiab] OR "Drug Therapy"[Subheading] OR trial[tiab] OR groups[tiab]) | [**4559743**](https://www.ncbi.nlm.nih.gov/pubmed/?cmd=HistorySearch&querykey=26) |
| [**#24**](https://www.ncbi.nlm.nih.gov/pubmed/advanced) | (review[tiab] OR "Review"[Publication Type] OR "Meta-Analysis as Topic"[Mesh] OR meta-analysis[tiab] OR "Meta-Analysis"[Publication Type]) NOT ("Letter"[Publication Type] OR "Editorial"[Publication Type] OR "Comment"[Publication Type])) | [**2986282**](https://www.ncbi.nlm.nih.gov/pubmed/?cmd=HistorySearch&querykey=24) |
| [**#23**](https://www.ncbi.nlm.nih.gov/pubmed/advanced) | (#22 NOT (("Adolescent"[Mesh] OR "Child"[Mesh] OR "Infant"[Mesh] OR adolescen*[tiab] OR child*[tiab] OR schoolchild*[tiab] OR infant*[tiab] OR girl*[tiab] OR boy*[tiab] OR teen[tiab] OR teens[tiab] OR teenager*[tiab] OR youth*[tiab] OR pediatr*[tiab] OR paediatr*[tiab] OR puber*[tiab]) NOT ("Adult"[Mesh] OR adult*[tiab] OR man[tiab] OR men[tiab] OR woman[tiab] OR women[tiab]))) | [**136**](https://www.ncbi.nlm.nih.gov/pubmed/?cmd=HistorySearch&querykey=23) |
| [**#6**](https://www.ncbi.nlm.nih.gov/pubmed/advanced) | ((#3 AND #4) OR #5) | [**13022**](https://www.ncbi.nlm.nih.gov/pubmed/?cmd=HistorySearch&querykey=6) |
| [**#5**](https://www.ncbi.nlm.nih.gov/pubmed/advanced) | lagb*[tiab] OR lrygb*[tiab] OR lap band*[tiab] OR lrygb*[tiab] | [**2048**](https://www.ncbi.nlm.nih.gov/pubmed/?cmd=HistorySearch&querykey=5) |
| [**#4**](https://www.ncbi.nlm.nih.gov/pubmed/advanced) | "Laparoscopy"[Mesh:NoExp] OR "Minimally Invasive Surgical Procedures"[Mesh:NoExp] OR laparoscop*[tiab] OR minimally invasive[tiab] OR minimal invasive[tiab] OR minimal access[tiab] OR minimal surg*[tiab] OR (minimal*[ti] AND invasive[ti]) OR (minimal[ti] AND (access[ti] OR surg*[ti])) | [**183110**](https://www.ncbi.nlm.nih.gov/pubmed/?cmd=HistorySearch&querykey=4) |
| [**#3**](https://www.ncbi.nlm.nih.gov/pubmed/advanced) | "Bariatric Surgery"[Mesh] OR "Obesity/surgery"[Mesh:NoExp] OR "Obesity, Abdominal/surgery"[Mesh] OR "Obesity, Metabolically Benign/surgery"[Mesh] OR "Obesity, Morbid/surgery"[Mesh] OR "Anastomosis, Roux-en-Y"[Mesh] OR "Biliopancreatic Diversion"[Mesh] OR "Overweight/surgery"[Mesh] OR "Gastrectomy"[Mesh] OR bariatric surg*[tiab] OR bariatric operati*[tiab] OR bariatric procedure*[tiab] OR bilio pancreatic bypass*[tiab] OR bilio pancreatic diversion*[tiab] OR biliopancreatic bypass*[tiab] OR biliopancreatic diversion*[tiab] OR duodenal switch*[tiab] OR gastric band*[tiab] OR stomach band*[tiab] OR gastric bypass*[tiab] OR gastric partiti*[tiab] OR gastric plicati*[tiab] OR gastroplast*[tiab] OR mgbp*[tiab] OR oagb*[tiab] OR obese surg*[tiab] OR weight loss surg*[tiab] OR obesity surg*[tiab] OR metabolic surg*[tiab] OR roux en y*[tiab] OR “roux y”[tiab] OR “roux n y”[tiab] OR “roux in y”[tiab] OR rya[tiab] OR rygb*[tiab] OR sadis[tiab] OR “sadi s”[tiab] OR sagb*[tiab] OR sleeve gastrectom*[tiab] OR gastric sleeve*[tiab] OR gastrectom*[tiab] OR ((bariatric[ti] OR obes*[ti] OR weight loss[ti] OR metabolic[ti]) AND (surg*[ti] OR operati*[ti] OR procedure*[ti])) OR ((biliopancreatic[ti] OR “bilio pancreatic”[ti]) AND (diversion*[ti] OR bypass*[ti])) OR (duodenal[ti] AND switch*[ti]) OR ((stomach[ti] OR gastric[ti]) AND (band*[ti] OR bypass*[ti] OR partiti*[ti] OR plicati*[ti] OR sleeve*[ti])) OR (sleeve[ti] AND gastrectom*[ti]) OR (roux[ti] AND y[ti]) | [**76980**](https://www.ncbi.nlm.nih.gov/pubmed/?cmd=HistorySearch&querykey=3) |

### T2Q6: Preoperative psychological consult (4 SRs, 13 RCTs, 31 cohort studies) (20 February 2019)

| **Search** | **Query** | **Items found** |
| --- | --- | --- |
| [**#12**](https://www.ncbi.nlm.nih.gov/pubmed) | ((#6 AND #11) NOT (#10 OR #8)) | [**31**](https://www.ncbi.nlm.nih.gov/pubmed/?cmd=HistorySearch&querykey=12) |
| [**#11**](https://www.ncbi.nlm.nih.gov/pubmed) | ("Epidemiologic Studies"[Mesh] OR "Prognosis"[Mesh] OR cohort[tiab] OR (case[tiab] AND (control[tiab] OR controll*[tiab] OR comparison[tiab] OR referent[tiab])) OR risk[tiab] OR causation[tiab] OR causal[tiab] OR "odds ratio"[tiab] OR etiol*[tiab] OR aetiol*[tiab] OR "natural history"[tiab] OR predict*[tiab] OR outcome[tiab] OR course[tiab] OR retrospect*[tiab] OR “follow up”[tiab] OR followup[tiab] OR prognos*[tiab]) | [**6719914**](https://www.ncbi.nlm.nih.gov/pubmed/?cmd=HistorySearch&querykey=11) |
| [**#10**](https://www.ncbi.nlm.nih.gov/pubmed) | ((#6 AND #9) NOT #8) | [**13**](https://www.ncbi.nlm.nih.gov/pubmed/?cmd=HistorySearch&querykey=10) |
| [**#9**](https://www.ncbi.nlm.nih.gov/pubmed) | ("Randomized Controlled Trial"[Publication Type] OR "Controlled Clinical Trial"[Publication Type] OR random*[tiab] OR placebo[tiab] OR "Drug Therapy"[Subheading] OR trial[tiab] OR groups[tiab]) | [**4637478**](https://www.ncbi.nlm.nih.gov/pubmed/?cmd=HistorySearch&querykey=9) |
| [**#8**](https://www.ncbi.nlm.nih.gov/pubmed) | (#6 AND #7) | [**4**](https://www.ncbi.nlm.nih.gov/pubmed/?cmd=HistorySearch&querykey=8) |
| [**#7**](https://www.ncbi.nlm.nih.gov/pubmed) | ((review[tiab] OR "Review"[Publication Type] OR "Meta-Analysis as Topic"[Mesh] OR meta-analysis[tiab] OR "Meta-Analysis"[Publication Type]) NOT ("Letter"[Publication Type] OR "Editorial"[Publication Type] OR "Comment"[Publication Type])) | [**3039634**](https://www.ncbi.nlm.nih.gov/pubmed/?cmd=HistorySearch&querykey=7) |
| [**#6**](https://www.ncbi.nlm.nih.gov/pubmed) | (#5 NOT (("Adolescent"[Mesh] OR "Child"[Mesh] OR "Infant"[Mesh] OR adolescen*[tiab] OR child*[tiab] OR schoolchild*[tiab] OR infant*[tiab] OR girl*[tiab] OR boy*[tiab] OR teen[tiab] OR teens[tiab] OR teenager*[tiab] OR youth*[tiab] OR pediatr*[tiab] OR paediatr*[tiab] OR puber*[tiab]) NOT ("Adult"[Mesh] OR adult*[tiab] OR man[tiab] OR men[tiab] OR woman[tiab] OR women[tiab]))) | [**49**](https://www.ncbi.nlm.nih.gov/pubmed/?cmd=HistorySearch&querykey=6) |
| [**#5**](https://www.ncbi.nlm.nih.gov/pubmed) | (#4 NOT ("Animals"[Mesh] NOT "Humans"[Mesh]) AND ("2004"[Date - Entrez] : "3000"[Date - Entrez])) | [**51**](https://www.ncbi.nlm.nih.gov/pubmed/?cmd=HistorySearch&querykey=5) |
| [**#4**](https://www.ncbi.nlm.nih.gov/pubmed) | (#1 AND #2 AND #3) | [**59**](https://www.ncbi.nlm.nih.gov/pubmed/?cmd=HistorySearch&querykey=4) |
| [**#3**](https://www.ncbi.nlm.nih.gov/pubmed) | ("Preoperative Period"[Mesh] OR "Preoperative Care"[Mesh] OR pre operat*[tiab] OR preoperat*[tiab] OR before surg*[tiab]) | [**351666**](https://www.ncbi.nlm.nih.gov/pubmed/?cmd=HistorySearch&querykey=3) |
| [**#2**](https://www.ncbi.nlm.nih.gov/pubmed) | "Psychological Techniques"[Mesh] OR "Psychological Tests"[Mesh] OR (("Counseling"[Mesh] OR consult*[tiab] OR counsel*[tiab] OR test[tiab] OR tests[tiab] OR testing[tiab] OR technique*[tiab] OR observati*[tiab]) AND ("psychology" [Subheading] OR psycholog*[tiab] OR psychometr*[tiab])) | [**604563**](https://www.ncbi.nlm.nih.gov/pubmed/?cmd=HistorySearch&querykey=2) |
| [**#1**](https://www.ncbi.nlm.nih.gov/pubmed) | (("Bariatric Surgery"[Mesh] OR "Obesity/surgery"[Mesh:NoExp] OR "Obesity, Abdominal/surgery"[Mesh] OR "Obesity, Metabolically Benign/surgery"[Mesh] OR "Obesity, Morbid/surgery"[Mesh] OR "Anastomosis, Roux-en-Y"[Mesh] OR "Biliopancreatic Diversion"[Mesh] OR "Overweight/surgery"[Mesh] OR "Gastrectomy"[Mesh] OR bariatric surg*[tiab] OR bariatric operati*[tiab] OR bariatric procedure*[tiab] OR bilio pancreatic bypass*[tiab] OR bilio pancreatic diversion*[tiab] OR biliopancreatic bypass*[tiab] OR biliopancreatic diversion*[tiab] OR duodenal switch*[tiab] OR gastric band*[tiab] OR stomach band*[tiab] OR gastric bypass*[tiab] OR gastric partiti*[tiab] OR gastric plicati*[tiab] OR gastroplast*[tiab] OR mgbp*[tiab] OR oagb*[tiab] OR obese surg*[tiab] OR weight loss surg*[tiab] OR obesity surg*[tiab] OR metabolic surg*[tiab] OR roux en y*[tiab] OR “roux y”[tiab] OR “roux n y”[tiab] OR “roux in y”[tiab] OR rya[tiab] OR rygb*[tiab] OR sadis[tiab] OR “sadi s”[tiab] OR sagb*[tiab] OR sleeve gastrectom*[tiab] OR gastric sleeve*[tiab] OR gastrectom*[tiab] OR ((bariatric[ti] OR obes*[ti] OR weight loss[ti] OR metabolic[ti]) AND (surg*[ti] OR operati*[ti] OR procedure*[ti])) OR ((biliopancreatic[ti] OR “bilio pancreatic”[ti]) AND (diversion*[ti] OR bypass*[ti])) OR (duodenal[ti] AND switch*[ti]) OR ((stomach[ti] OR gastric[ti]) AND (band*[ti] OR bypass*[ti] OR partiti*[ti] OR plicati*[ti] OR sleeve*[ti])) OR (sleeve[ti] AND gastrectom*[ti]) OR (roux[ti] AND y[ti])) AND ("Laparoscopy"[Mesh:NoExp] OR "Minimally Invasive Surgical Procedures"[Mesh:NoExp] OR laparoscop*[tiab] OR minimally invasive[tiab] OR minimal invasive[tiab] OR minimal access[tiab] OR minimal surg*[tiab] OR (minimal*[ti] AND invasive[ti]) OR (minimal[ti] AND (access[ti] OR surg*[ti])))) OR (lagb*[tiab] OR lrygb*[tiab] OR lap band*[tiab] OR lrygb*[tiab]) | [**13441**](https://www.ncbi.nlm.nih.gov/pubmed/?cmd=HistorySearch&querykey=1) |

## Embase.com (30 October + 10 December 2018 + 20 February 2019)

### T2Q1: Preoperative diet (21 SRs, 18 RCTs, 45 cohort studies) (30 October 2018)

| **No.** | **Query** | **Results** |
| --- | --- | --- |
| **#15** | #10 AND #14 NOT (#12 OR #11) | **45** |
| **#14** | 'epidemiology'/de OR (((cohort OR case) NEAR/3 (control OR controll* OR comparison OR referent)):ti,ab,kw) OR risk:ti,ab,kw OR causation:ti,ab,kw OR causal:ti,ab,kw OR 'odds ratio':ti,ab,kw OR etiol*:ti,ab,kw OR aetiol*:ti,ab,kw OR 'natural history':ti,ab,kw OR outcome:ti,ab,kw OR course:ti,ab,kw OR retrospect*:ti,ab,kw OR 'follow up':ti,ab,kw OR followup:ti,ab,kw OR predict*:ti,ab,kw OR prognos*:ti,ab,kw | **7283403** |
| **#13** | #10 AND #12 NOT #11 | **18** |
| **#12** | 'clinical':ti,ab,kw AND 'trial':ti,ab,kw OR 'clinical trial'/exp OR random*:ti,ab,kw | **2279278** |
| **#11** | #7 AND #10 | **21** |
| **#10** | #4 AND #9 | **117** |
| **#9** | ('diet therapy'/exp/mj OR diet:ti,kw OR diets:ti,kw OR dietary:ti,kw) AND ('preoperative period'/exp OR 'pre operat*':ti,kw OR preoperat*:ti,kw OR 'before surg*':ti,kw) | **1230** |
| **#8** | #6 AND #7 | **140** |
| **#7** | 'meta-analysis':ti,ab,kw OR 'meta analysis'/exp OR 'review'/exp OR review:ti,ab,kw | **3421796** |
| **#6** | #4 AND #5 | **679** |
| **#5** | ('diet therapy'/exp OR diet:ti,ab,kw OR diets:ti,ab,kw OR dietary:ti,ab,kw) AND ('preoperative period'/exp OR 'pre operat*':ti,ab,kw OR preoperat*:ti,ab,kw OR 'before surg*':ti,ab,kw) | **9081** |
| **#4** | #3 NOT (('adolescent'/exp OR 'child'/exp OR adolescent*:ti,ab OR child*:ti,ab OR schoolchild*:ti,ab OR infant*:ti,ab OR girl*:ti,ab OR boy*:ti,ab OR teen:ti,ab OR teens:ti,ab OR teenager*:ti,ab OR youth*:ti,ab OR pediatr*:ti,ab OR paediatr*:ti,ab OR puber*:ti,ab) NOT ('adult'/exp OR 'aged'/exp OR 'middle aged'/exp OR adult*:ti,ab OR man:ti,ab OR men:ti,ab OR woman:ti,ab OR women:ti,ab)) | **19626** |
| **#3** | #2 NOT ('conference abstract'/it OR 'conference paper'/it OR 'letter'/it OR 'note'/it) | **20161** |
| **#2** | #1 NOT ([animals]/lim NOT [humans]/lim) AND [1-1-2004]/sd | **35675** |
| **#1** | ('obesity'/exp/mj AND 'surgery'/lnk OR 'bariatric surgery'/exp/mj OR 'roux y anastomosis'/exp/mj OR 'gastric bypass surgery'/exp/mj OR ((('bilio pancreatic' OR biliopancreatic) NEAR/3 (diversion* OR bypass*)):ti,kw) OR ((duodenal NEAR/3 switch*):ti,kw) OR (((stomach OR gastric) NEAR/3 (band* OR bypass* OR partiti* OR plicati* OR sleeve*)):ti,kw) OR ((sleeve NEAR/3 gastrectom*):ti,kw) OR ((roux NEAR/2 y):ti,kw) OR gastroplast*:ti,kw OR mgbp*:ti,kw OR oagb*:ti,kw OR rya:ti,kw OR rygb*:ti,kw OR sadis:ti,kw OR 'sadi s':ti,kw OR (((bariatric OR obes* OR 'weight loss' OR metabolic) NEAR/3 (surg* OR operati* OR procedure*)):ti,kw) OR sagb*:ti,kw) AND ('laparoscopy'/de OR 'laparoendoscopic single site surgery'/exp OR 'laparoscopic surgery'/exp OR 'minimally invasive procedure'/exp OR 'minimally invasive surgery'/exp OR laparoscop*:ti,ab,kw OR ((minimal* NEAR/3 invasive):ti,ab,kw) OR access:ti,ab,kw OR surg*:ti,ab,kw) OR 'laparoscopic sleeve gastrectomy'/exp OR lagb*:ti,ab,kw OR 'lap band*':ti,ab,kw OR lrygb*:ti,ab,kw | **40079** |

### T2Q3: Smoking cessation (12 SRs, 2 RCTs, 13 cohort studies) (10 December 2018)

| **No.** | **Query** | **Results** |
| --- | --- | --- |
| **#12** | #6 AND #11 NOT (#8 OR #10) | **13** |
| **#11** | 'epidemiology'/de OR (((cohort OR case) NEAR/3 (control OR controll* OR comparison OR referent)):ti,ab,kw) OR risk:ti,ab,kw OR causation:ti,ab,kw OR causal:ti,ab,kw OR 'odds ratio':ti,ab,kw OR etiol*:ti,ab,kw OR aetiol*:ti,ab,kw OR 'natural history':ti,ab,kw OR outcome:ti,ab,kw OR course:ti,ab,kw OR retrospect*:ti,ab,kw OR 'follow up':ti,ab,kw OR followup:ti,ab,kw OR predict*:ti,ab,kw OR prognos*:ti,ab,kw | **7360269** |
| **#10** | #6 AND #9 NOT #8 | **2** |
| **#9** | 'clinical':ti,ab,kw AND 'trial':ti,ab,kw OR 'clinical trial'/exp OR random*:ti,ab,kw | **2299001** |
| **#8** | #6 AND #7 | **12** |
| **#7** | 'meta-analysis':ti,ab,kw OR 'meta analysis'/exp OR 'review'/exp OR review:ti,ab,kw | **3451178** |
| **#6** | #5 NOT (('adolescent'/exp OR 'child'/exp OR adolescent*:ti,ab OR child*:ti,ab OR schoolchild*:ti,ab OR infant*:ti,ab OR girl*:ti,ab OR boy*:ti,ab OR teen:ti,ab OR teens:ti,ab OR teenager*:ti,ab OR youth*:ti,ab OR pediatr*:ti,ab OR paediatr*:ti,ab OR puber*:ti,ab) NOT ('adult'/exp OR 'aged'/exp OR 'middle aged'/exp OR adult*:ti,ab OR man:ti,ab OR men:ti,ab OR woman:ti,ab OR women:ti,ab)) | **35** |
| **#5** | #4 NOT ('conference abstract'/it OR 'conference paper'/it OR 'letter'/it OR 'note'/it) | **35** |
| **#4** | #3 NOT ([animals]/lim NOT [humans]/lim) AND [1-1-2004]/sd | **63** |
| **#3** | #1 AND #2 | **64** |
| **#2** | 'smoking cessation'/exp OR (((smoking OR tobacco OR nicotin*) NEAR/3 (cessati* OR stop* OR 'giving up' OR quitting OR dehabituati*)):ti,ab,kw) | **62926** |
| **#1** | ('obesity'/exp/mj AND 'surgery'/lnk OR 'bariatric surgery'/exp/mj OR 'roux y anastomosis'/exp/mj OR 'gastric bypass surgery'/exp/mj OR ((('bilio pancreatic' OR biliopancreatic) NEAR/3 (diversion* OR bypass*)):ti,kw) OR ((duodenal NEAR/3 switch*):ti,kw) OR (((stomach OR gastric) NEAR/3 (band* OR bypass* OR partiti* OR plicati* OR sleeve*)):ti,kw) OR ((sleeve NEAR/3 gastrectom*):ti,kw) OR ((roux NEAR/2 y):ti,kw) OR gastroplast*:ti,kw OR mgbp*:ti,kw OR oagb*:ti,kw OR rya:ti,kw OR rygb*:ti,kw OR sadis:ti,kw OR 'sadi s':ti,kw OR (((bariatric OR obes* OR 'weight loss' OR metabolic) NEAR/3 (surg* OR operati* OR procedure*)):ti,kw) OR sagb*:ti,kw) AND ('laparoscopy'/de OR 'laparoendoscopic single site surgery'/exp OR 'laparoscopic surgery'/exp OR 'minimally invasive procedure'/exp OR 'minimally invasive surgery'/exp OR laparoscop*:ti,ab,kw OR ((minimal* NEAR/3 invasive):ti,ab,kw) OR access:ti,ab,kw OR surg*:ti,ab,kw) OR 'laparoscopic sleeve gastrectomy'/exp OR lagb*:ti,ab,kw OR 'lap band*':ti,ab,kw OR lrygb*:ti,ab,kw | **40862** |

### T2Q4: H. pylori (32 SRs, 6 RCTs, 60 cohort studies) (30 October 2018)

| **No.** | **Query** | **Results** |
| --- | --- | --- |
| **#12** | #9 AND #7 NOT (#10 OR #11) | **60** |
| **#11** | #9 AND #6 NOT #10 | **6** |
| **#10** | #5 AND #9 | **32** |
| **#9** | #4 AND #8 | **145** |
| **#8** | 'helicobacter pylori'/exp OR 'helicobacter pylori*':ti,ab,kw OR 'h pylori*':ti,ab,kw | **64601** |
| **#7** | 'epidemiology'/de OR (((cohort OR case) NEAR/3 (control OR controll* OR comparison OR referent)):ti,ab,kw) OR risk:ti,ab,kw OR causation:ti,ab,kw OR causal:ti,ab,kw OR 'odds ratio':ti,ab,kw OR etiol*:ti,ab,kw OR aetiol*:ti,ab,kw OR 'natural history':ti,ab,kw OR outcome:ti,ab,kw OR course:ti,ab,kw OR retrospect*:ti,ab,kw OR 'follow up':ti,ab,kw OR followup:ti,ab,kw OR predict*:ti,ab,kw OR prognos*:ti,ab,kw | **7283403** |
| **#6** | 'clinical':ti,ab,kw AND 'trial':ti,ab,kw OR 'clinical trial'/exp OR random*:ti,ab,kw | **2279278** |
| **#5** | 'meta-analysis':ti,ab,kw OR 'meta analysis'/exp OR 'review'/exp OR review:ti,ab,kw | **3421796** |
| **#4** | #3 NOT (('adolescent'/exp OR 'child'/exp OR adolescent*:ti,ab OR child*:ti,ab OR schoolchild*:ti,ab OR infant*:ti,ab OR girl*:ti,ab OR boy*:ti,ab OR teen:ti,ab OR teens:ti,ab OR teenager*:ti,ab OR youth*:ti,ab OR pediatr*:ti,ab OR paediatr*:ti,ab OR puber*:ti,ab) NOT ('adult'/exp OR 'aged'/exp OR 'middle aged'/exp OR adult*:ti,ab OR man:ti,ab OR men:ti,ab OR woman:ti,ab OR women:ti,ab)) | **19626** |
| **#3** | #2 NOT ('conference abstract'/it OR 'conference paper'/it OR 'letter'/it OR 'note'/it) | **20161** |
| **#2** | #1 NOT ([animals]/lim NOT [humans]/lim) AND [1-1-2004]/sd | **35675** |
| **#1** | ('obesity'/exp/mj AND 'surgery'/lnk OR 'bariatric surgery'/exp/mj OR 'roux y anastomosis'/exp/mj OR 'gastric bypass surgery'/exp/mj OR ((('bilio pancreatic' OR biliopancreatic) NEAR/3 (diversion* OR bypass*)):ti,kw) OR ((duodenal NEAR/3 switch*):ti,kw) OR (((stomach OR gastric) NEAR/3 (band* OR bypass* OR partiti* OR plicati* OR sleeve*)):ti,kw) OR ((sleeve NEAR/3 gastrectom*):ti,kw) OR ((roux NEAR/2 y):ti,kw) OR gastroplast*:ti,kw OR mgbp*:ti,kw OR oagb*:ti,kw OR rya:ti,kw OR rygb*:ti,kw OR sadis:ti,kw OR 'sadi s':ti,kw OR (((bariatric OR obes* OR 'weight loss' OR metabolic) NEAR/3 (surg* OR operati* OR procedure*)):ti,kw) OR sagb*:ti,kw) AND ('laparoscopy'/de OR 'laparoendoscopic single site surgery'/exp OR 'laparoscopic surgery'/exp OR 'minimally invasive procedure'/exp OR 'minimally invasive surgery'/exp OR laparoscop*:ti,ab,kw OR ((minimal* NEAR/3 invasive):ti,ab,kw) OR access:ti,ab,kw OR surg*:ti,ab,kw) OR 'laparoscopic sleeve gastrectomy'/exp OR lagb*:ti,ab,kw OR 'lap band*':ti,ab,kw OR lrygb*:ti,ab,kw | **40079** |

### T2Q6: Preoperative psychological consult (60 SRs, 12 RCTs, 94 cohort studies) (20 February 2019)

| **No.** | **Query** | **Results** |
| --- | --- | --- |
| **#13** | #7 AND #12 NOT (#11 OR #9) | **94** |
| **#12** | 'epidemiology'/de OR (((cohort OR case) NEAR/3 (control OR controll* OR comparison OR referent)):ti,ab,kw) OR risk:ti,ab,kw OR causation:ti,ab,kw OR causal:ti,ab,kw OR 'odds ratio':ti,ab,kw OR etiol*:ti,ab,kw OR aetiol*:ti,ab,kw OR 'natural history':ti,ab,kw OR outcome:ti,ab,kw OR course:ti,ab,kw OR retrospect*:ti,ab,kw OR 'follow up':ti,ab,kw OR followup:ti,ab,kw OR predict*:ti,ab,kw OR prognos*:ti,ab,kw | **7463105** |
| **#11** | #7 AND #10 NOT #9 | **12** |
| **#10** | 'clinical':ti,ab,kw AND 'trial':ti,ab,kw OR 'clinical trial'/exp OR random*:ti,ab,kw | **2328797** |
| **#9** | #7 AND #8 | **60** |
| **#8** | 'meta-analysis':ti,ab,kw OR 'meta analysis'/exp OR 'review'/exp OR review:ti,ab,kw | **3489113** |
| **#7** | #6 NOT (('adolescent'/exp OR 'child'/exp OR adolescent*:ti,ab OR child*:ti,ab OR schoolchild*:ti,ab OR infant*:ti,ab OR girl*:ti,ab OR boy*:ti,ab OR teen:ti,ab OR teens:ti,ab OR teenager*:ti,ab OR youth*:ti,ab OR pediatr*:ti,ab OR paediatr*:ti,ab OR puber*:ti,ab) NOT ('adult'/exp OR 'aged'/exp OR 'middle aged'/exp OR adult*:ti,ab OR man:ti,ab OR men:ti,ab OR woman:ti,ab OR women:ti,ab)) | **222** |
| **#6** | #5 NOT ('conference abstract'/it OR 'conference paper'/it OR 'letter'/it OR 'note'/it) | **233** |
| **#5** | #4 NOT ([animals]/lim NOT [humans]/lim) AND [1-1-2004]/sd | **359** |
| **#4** | #1 AND #2 AND #3 | **404** |
| **#3** | 'preoperative period'/exp OR 'pre operat*':ti,ab,kw OR preoperat*:ti,ab,kw OR 'before surg*':ti,ab,kw | **590278** |
| **#2** | 'psychological aspect'/exp OR 'psychologic test'/exp OR 'psychometry'/exp OR (('counseling'/exp OR consult*:ti,ab,kw OR counsel*:ti,ab,kw OR test:ti,ab,kw OR tests:ti,ab,kw OR testing:ti,ab,kw OR technique*:ti,ab,kw OR observati*:ti,ab,kw) AND ('psychology'/exp OR psycholog*:ti,ab,kw OR psychometr*:ti,ab,kw)) | **805329** |
| **#1** | ('obesity'/exp/mj AND 'surgery'/lnk OR 'bariatric surgery'/exp/mj OR 'roux y anastomosis'/exp/mj OR 'gastric bypass surgery'/exp/mj OR ((('bilio pancreatic' OR biliopancreatic) NEAR/3 (diversion* OR bypass*)):ti,kw) OR ((duodenal NEAR/3 switch*):ti,kw) OR (((stomach OR gastric) NEAR/3 (band* OR bypass* OR partiti* OR plicati* OR sleeve*)):ti,kw) OR ((sleeve NEAR/3 gastrectom*):ti,kw) OR ((roux NEAR/2 y):ti,kw) OR gastroplast*:ti,kw OR mgbp*:ti,kw OR oagb*:ti,kw OR rya:ti,kw OR rygb*:ti,kw OR sadis:ti,kw OR 'sadi s':ti,kw OR (((bariatric OR obes* OR 'weight loss' OR metabolic) NEAR/3 (surg* OR operati* OR procedure*)):ti,kw) OR sagb*:ti,kw) AND ('laparoscopy'/de OR 'laparoendoscopic single site surgery'/exp OR 'laparoscopic surgery'/exp OR 'minimally invasive procedure'/exp OR 'minimally invasive surgery'/exp OR laparoscop*:ti,ab,kw OR ((minimal* NEAR/3 invasive):ti,ab,kw) OR access:ti,ab,kw OR surg*:ti,ab,kw) OR 'laparoscopic sleeve gastrectomy'/exp OR lagb*:ti,ab,kw OR 'lap band*':ti,ab,kw OR lrygb*:ti,ab,kw | **41440** |

## Wiley/Cochrane Library-CENTRAL (30 October 2018)

### T2Q1: Diet (20)

| **ID** | **Search** | **Hits** |
| --- | --- | --- |
| **#1** | (((“bilio pancreatic” OR biliopancreatic) NEAR/3 (diversion* OR bypass*)) OR (duodenal NEAR/3 switch*) OR ((stomach OR gastric) NEAR/3 (band* OR bypass* OR partiti* OR plicati* OR sleeve*)) OR (sleeve NEAR/3 gastrectom*) OR (roux NEAR/2 y) OR gastroplast* OR mgbp* OR oagb* OR rya OR rygb* OR sadis OR “sadi s” OR ((bariatric OR obes* OR “weight loss” OR metabolic) NEAR/3 (surg* OR operati* OR procedure*)) OR sagb*):ti,ab,kw | **3636** |
| **#2** | (laparoscop* OR (minimal* NEAR/3 (invasive OR access OR surg*))):ti,ab,kw | **17676** |
| **#3** | #1 AND #2 | **1205** |
| **#4** | (lagb* OR lrygb* OR “lap band*” OR lrygb*):ti,ab,kw | **238** |
| **#5** | #3 OR #4 with Cochrane Library publication date from Jan 2004 to present | **1175** |
| **#6** | ((diet OR diets OR dietary) NEAR/3 (“pre operat*” OR preoperat* OR “before surg*”)):ti,ab,kw | **70** |
| **#7** | #5 AND #6 | **20** |

### T2Q3: Smoking cessation (2)

| **ID** | **Search** | **Hits** |
| --- | --- | --- |
| **#1** | (((“bilio pancreatic” OR biliopancreatic) NEAR/3 (diversion* OR bypass*)) OR (duodenal NEAR/3 switch*) OR ((stomach OR gastric) NEAR/3 (band* OR bypass* OR partiti* OR plicati* OR sleeve*)) OR (sleeve NEAR/3 gastrectom*) OR (roux NEAR/2 y) OR gastroplast* OR mgbp* OR oagb* OR rya OR rygb* OR sadis OR “sadi s” OR ((bariatric OR obes* OR “weight loss” OR metabolic) NEAR/3 (surg* OR operati* OR procedure*)) OR sagb*):ti,ab,kw | **3636** |
| **#2** | (laparoscop* OR (minimal* NEAR/3 (invasive OR access OR surg*))):ti,ab,kw | **17676** |
| **#3** | #1 AND #2 | **1205** |
| **#4** | (lagb* OR lrygb* OR “lap band*” OR lrygb*):ti,ab,kw | **238** |
| **#5** | #3 OR #4 with Cochrane Library publication date from Jan 2004 to present | **1175** |
| **#6** | ((smoking OR tobacco OR nicotin*) NEAR/3 (cessati* OR stop* OR “giving up” OR quitting OR dehabituati*)):ti,ab,kw | **8413** |
| **#7** | #5 AND #6 | **2** |

### T2Q4: H. pylori (4)

| **ID** | **Search** | **Hits** |
| --- | --- | --- |
| **#1** | (((“bilio pancreatic” OR biliopancreatic) NEAR/3 (diversion* OR bypass*)) OR (duodenal NEAR/3 switch*) OR ((stomach OR gastric) NEAR/3 (band* OR bypass* OR partiti* OR plicati* OR sleeve*)) OR (sleeve NEAR/3 gastrectom*) OR (roux NEAR/2 y) OR gastroplast* OR mgbp* OR oagb* OR rya OR rygb* OR sadis OR “sadi s” OR ((bariatric OR obes* OR “weight loss” OR metabolic) NEAR/3 (surg* OR operati* OR procedure*)) OR sagb*):ti,ab,kw | **3636** |
| **#2** | (laparoscop* OR (minimal* NEAR/3 (invasive OR access OR surg*))):ti,ab,kw | **17676** |
| **#3** | #1 AND #2 | **1205** |
| **#4** | (lagb* OR lrygb* OR “lap band*” OR lrygb*):ti,ab,kw | **238** |
| **#5** | #3 OR #4 with Cochrane Library publication date from Jan 2004 to present | **1175** |
| **#6** | (“helicobacter pylori*” OR “h pylori*”):ti,ab,kw | **4486** |
| **#7** | #5 AND #6 | **4** |

# Topic 3: Perioperative management

## PubMed (9 November 2018 + 11 February 2019)

### T3Q1: OSA + CPAP (7 SRs, 11 RCTs, 15 cohort studies) (9 November 2018)

| **Search** | **Query** | **Items found** |
| --- | --- | --- |
| [**#35**](https://www.ncbi.nlm.nih.gov/pubmed) | ((#29 AND #34) NOT (#32 OR #31)) | [**15**](https://www.ncbi.nlm.nih.gov/pubmed/?cmd=HistorySearch&querykey=35) |
| [**#34**](https://www.ncbi.nlm.nih.gov/pubmed) | "Epidemiologic Studies"[Mesh] OR "Prognosis"[Mesh] OR cohort[tiab] OR (case[tiab] AND (control[tiab] OR controll*[tiab] OR comparison[tiab] OR referent[tiab])) OR risk[tiab] OR causation[tiab] OR causal[tiab] OR "odds ratio"[tiab] OR etiol*[tiab] OR aetiol*[tiab] OR "natural history"[tiab] OR predict*[tiab] OR outcome[tiab] OR course[tiab] OR retrospect*[tiab] OR “follow up”[tiab] OR followup[tiab] OR prognos*[tiab] | [**6595486**](https://www.ncbi.nlm.nih.gov/pubmed/?cmd=HistorySearch&querykey=34) |
| [**#33**](https://www.ncbi.nlm.nih.gov/pubmed) | ((#29 AND #32) NOT #31) | [**11**](https://www.ncbi.nlm.nih.gov/pubmed/?cmd=HistorySearch&querykey=33) |
| [**#32**](https://www.ncbi.nlm.nih.gov/pubmed) | "Randomized Controlled Trial"[Publication Type] OR "Controlled Clinical Trial"[Publication Type] OR random*[tiab] OR placebo[tiab] OR "Drug Therapy"[Subheading] OR trial[tiab] OR groups[tiab] | [**4567282**](https://www.ncbi.nlm.nih.gov/pubmed/?cmd=HistorySearch&querykey=32) |
| [**#31**](https://www.ncbi.nlm.nih.gov/pubmed) | #29 AND #30 | [**7**](https://www.ncbi.nlm.nih.gov/pubmed/?cmd=HistorySearch&querykey=31) |
| [**#30**](https://www.ncbi.nlm.nih.gov/pubmed) | (review[tiab] OR "Review"[Publication Type] OR "Meta-Analysis as Topic"[Mesh] OR meta-analysis[tiab] OR "Meta-Analysis"[Publication Type]) NOT ("Letter"[Publication Type] OR "Editorial"[Publication Type] OR "Comment"[Publication Type]) | [**2991355**](https://www.ncbi.nlm.nih.gov/pubmed/?cmd=HistorySearch&querykey=30) |
| [**#29**](https://www.ncbi.nlm.nih.gov/pubmed) | #28 NOT (("Adolescent"[Mesh] OR "Child"[Mesh] OR "Infant"[Mesh] OR adolescen*[tiab] OR child*[tiab] OR schoolchild*[tiab] OR infant*[tiab] OR girl*[tiab] OR boy*[tiab] OR teen[tiab] OR teens[tiab] OR teenager*[tiab] OR youth*[tiab] OR pediatr*[tiab] OR paediatr*[tiab] OR puber*[tiab]) NOT ("Adult"[Mesh] OR adult*[tiab] OR man[tiab] OR men[tiab] OR woman[tiab] OR women[tiab])) | [**35**](https://www.ncbi.nlm.nih.gov/pubmed/?cmd=HistorySearch&querykey=29) |
| [**#28**](https://www.ncbi.nlm.nih.gov/pubmed) | (#27 NOT ("Animals"[Mesh] NOT "Humans"[Mesh]) AND ("2004"[Date - Entrez] : "3000"[Date - Entrez])) | [**35**](https://www.ncbi.nlm.nih.gov/pubmed/?cmd=HistorySearch&querykey=28) |
| [**#27**](https://www.ncbi.nlm.nih.gov/pubmed) | (#25 AND #26) | [**37**](https://www.ncbi.nlm.nih.gov/pubmed/?cmd=HistorySearch&querykey=27) |
| [**#26**](https://www.ncbi.nlm.nih.gov/pubmed) | (("Sleep Apnea, Obstructive"[Mesh] OR sleep apnea*[tiab] OR nocturnal apnea*[tiab] OR sleep hypopnea*[tiab] OR sleep apnoea*[tiab] OR nocturnal apnoea*[tiab] OR sleep hypopnoea*[tiab] OR sleep-disordered breathing[tiab] OR osahs[tiab] OR osas[tiab]) AND ("Continuous Positive Airway Pressure"[Mesh] OR continuous positive airway pressure*[tiab] OR cpap*[tiab] OR ncpap*[tiab] OR airway pressure release ventilati*[tiab] OR aprv*[tiab])) | [**7309**](https://www.ncbi.nlm.nih.gov/pubmed/?cmd=HistorySearch&querykey=26) |
| [**#25**](https://www.ncbi.nlm.nih.gov/pubmed) | (("Bariatric Surgery"[Mesh] OR "Obesity/surgery"[Mesh:NoExp] OR "Obesity, Abdominal/surgery"[Mesh] OR "Obesity, Metabolically Benign/surgery"[Mesh] OR "Obesity, Morbid/surgery"[Mesh] OR "Anastomosis, Roux-en-Y"[Mesh] OR "Biliopancreatic Diversion"[Mesh] OR "Overweight/surgery"[Mesh] OR "Gastrectomy"[Mesh] OR bariatric surg*[tiab] OR bariatric operati*[tiab] OR bariatric procedure*[tiab] OR bilio pancreatic bypass*[tiab] OR bilio pancreatic diversion*[tiab] OR biliopancreatic bypass*[tiab] OR biliopancreatic diversion*[tiab] OR duodenal switch*[tiab] OR gastric band*[tiab] OR stomach band*[tiab] OR gastric bypass*[tiab] OR gastric partiti*[tiab] OR gastric plicati*[tiab] OR gastroplast*[tiab] OR mgbp*[tiab] OR oagb*[tiab] OR obese surg*[tiab] OR weight loss surg*[tiab] OR obesity surg*[tiab] OR metabolic surg*[tiab] OR roux en y*[tiab] OR “roux y”[tiab] OR “roux n y”[tiab] OR “roux in y”[tiab] OR rya[tiab] OR rygb*[tiab] OR sadis[tiab] OR “sadi s”[tiab] OR sagb*[tiab] OR sleeve gastrectom*[tiab] OR gastric sleeve*[tiab] OR gastrectom*[tiab] OR ((bariatric[ti] OR obes*[ti] OR weight loss[ti] OR metabolic[ti]) AND (surg*[ti] OR operati*[ti] OR procedure*[ti])) OR ((biliopancreatic[ti] OR “bilio pancreatic”[ti]) AND (diversion*[ti] OR bypass*[ti])) OR (duodenal[ti] AND switch*[ti]) OR ((stomach[ti] OR gastric[ti]) AND (band*[ti] OR bypass*[ti] OR partiti*[ti] OR plicati*[ti] OR sleeve*[ti])) OR (sleeve[ti] AND gastrectom*[ti]) OR (roux[ti] AND y[ti])) AND ("Laparoscopy"[Mesh:NoExp] OR "Minimally Invasive Surgical Procedures"[Mesh:NoExp] OR laparoscop*[tiab] OR minimally invasive[tiab] OR minimal invasive[tiab] OR minimal access[tiab] OR minimal surg*[tiab] OR (minimal*[ti] AND invasive[ti]) OR (minimal[ti] AND (access[ti] OR surg*[ti])))) OR (lagb*[tiab] OR lrygb*[tiab] OR lap band*[tiab] OR lrygb*[tiab]) | [**13065**](https://www.ncbi.nlm.nih.gov/pubmed/?cmd=HistorySearch&querykey=25) |

### T3Q2: Multimodal analgesia (6 SRs, 19 RCTs, 4 cohort studies) (9 November 2018)

| **Search** | **Query** | **Items found** |
| --- | --- | --- |
| [**#41**](https://www.ncbi.nlm.nih.gov/pubmed) | ((#38 AND #34) NOT (#39 OR #40)) | [**4**](https://www.ncbi.nlm.nih.gov/pubmed/?cmd=HistorySearch&querykey=41) |
| [**#40**](https://www.ncbi.nlm.nih.gov/pubmed) | ((#38 AND #32) NOT #39) | [**19**](https://www.ncbi.nlm.nih.gov/pubmed/?cmd=HistorySearch&querykey=40) |
| [**#39**](https://www.ncbi.nlm.nih.gov/pubmed) | (#38 AND #30) | [**6**](https://www.ncbi.nlm.nih.gov/pubmed/?cmd=HistorySearch&querykey=39) |
| [**#38**](https://www.ncbi.nlm.nih.gov/pubmed) | (#37 NOT (("Adolescent"[Mesh] OR "Child"[Mesh] OR "Infant"[Mesh] OR adolescen*[tiab] OR child*[tiab] OR schoolchild*[tiab] OR infant*[tiab] OR girl*[tiab] OR boy*[tiab] OR teen[tiab] OR teens[tiab] OR teenager*[tiab] OR youth*[tiab] OR pediatr*[tiab] OR paediatr*[tiab] OR puber*[tiab]) NOT ("Adult"[Mesh] OR adult*[tiab] OR man[tiab] OR men[tiab] OR woman[tiab] OR women[tiab]))) | [**30**](https://www.ncbi.nlm.nih.gov/pubmed/?cmd=HistorySearch&querykey=38) |
| [**#37**](https://www.ncbi.nlm.nih.gov/pubmed) | (#36 NOT ("Animals"[Mesh] NOT "Humans"[Mesh]) AND ("2004"[Date - Entrez] : "3000"[Date - Entrez])) | [**30**](https://www.ncbi.nlm.nih.gov/pubmed/?cmd=HistorySearch&querykey=37) |
| [**#36**](https://www.ncbi.nlm.nih.gov/pubmed) | (#25 AND #35) | [**30**](https://www.ncbi.nlm.nih.gov/pubmed/?cmd=HistorySearch&querykey=36) |
| [**#35**](https://www.ncbi.nlm.nih.gov/pubmed) | (multimodal*[tiab] AND ("Analgesia"[Mesh] OR "Analgesics"[Mesh] OR "Analgesics"[Pharmacological Action] OR "Anesthesia"[Mesh] OR "Anesthetics"[Mesh] OR "Anesthetics"[Pharmacological Action] OR analgesi*[tiab] OR anestheti*[tiab] OR anaestheti*[tiab] OR nerve block*[tiab])) | [**3188**](https://www.ncbi.nlm.nih.gov/pubmed/?cmd=HistorySearch&querykey=35) |
| [**#34**](https://www.ncbi.nlm.nih.gov/pubmed) | "Epidemiologic Studies"[Mesh] OR "Prognosis"[Mesh] OR cohort[tiab] OR (case[tiab] AND (control[tiab] OR controll*[tiab] OR comparison[tiab] OR referent[tiab])) OR risk[tiab] OR causation[tiab] OR causal[tiab] OR "odds ratio"[tiab] OR etiol*[tiab] OR aetiol*[tiab] OR "natural history"[tiab] OR predict*[tiab] OR outcome[tiab] OR course[tiab] OR retrospect*[tiab] OR “follow up”[tiab] OR followup[tiab] OR prognos*[tiab] | [**6595486**](https://www.ncbi.nlm.nih.gov/pubmed/?cmd=HistorySearch&querykey=34) |
| [**#32**](https://www.ncbi.nlm.nih.gov/pubmed) | "Randomized Controlled Trial"[Publication Type] OR "Controlled Clinical Trial"[Publication Type] OR random*[tiab] OR placebo[tiab] OR "Drug Therapy"[Subheading] OR trial[tiab] OR groups[tiab] | [**4567282**](https://www.ncbi.nlm.nih.gov/pubmed/?cmd=HistorySearch&querykey=32) |
| [**#30**](https://www.ncbi.nlm.nih.gov/pubmed) | (review[tiab] OR "Review"[Publication Type] OR "Meta-Analysis as Topic"[Mesh] OR meta-analysis[tiab] OR "Meta-Analysis"[Publication Type]) NOT ("Letter"[Publication Type] OR "Editorial"[Publication Type] OR "Comment"[Publication Type]) | [**2991355**](https://www.ncbi.nlm.nih.gov/pubmed/?cmd=HistorySearch&querykey=30) |
| [**#25**](https://www.ncbi.nlm.nih.gov/pubmed) | (("Bariatric Surgery"[Mesh] OR "Obesity/surgery"[Mesh:NoExp] OR "Obesity, Abdominal/surgery"[Mesh] OR "Obesity, Metabolically Benign/surgery"[Mesh] OR "Obesity, Morbid/surgery"[Mesh] OR "Anastomosis, Roux-en-Y"[Mesh] OR "Biliopancreatic Diversion"[Mesh] OR "Overweight/surgery"[Mesh] OR "Gastrectomy"[Mesh] OR bariatric surg*[tiab] OR bariatric operati*[tiab] OR bariatric procedure*[tiab] OR bilio pancreatic bypass*[tiab] OR bilio pancreatic diversion*[tiab] OR biliopancreatic bypass*[tiab] OR biliopancreatic diversion*[tiab] OR duodenal switch*[tiab] OR gastric band*[tiab] OR stomach band*[tiab] OR gastric bypass*[tiab] OR gastric partiti*[tiab] OR gastric plicati*[tiab] OR gastroplast*[tiab] OR mgbp*[tiab] OR oagb*[tiab] OR obese surg*[tiab] OR weight loss surg*[tiab] OR obesity surg*[tiab] OR metabolic surg*[tiab] OR roux en y*[tiab] OR “roux y”[tiab] OR “roux n y”[tiab] OR “roux in y”[tiab] OR rya[tiab] OR rygb*[tiab] OR sadis[tiab] OR “sadi s”[tiab] OR sagb*[tiab] OR sleeve gastrectom*[tiab] OR gastric sleeve*[tiab] OR gastrectom*[tiab] OR ((bariatric[ti] OR obes*[ti] OR weight loss[ti] OR metabolic[ti]) AND (surg*[ti] OR operati*[ti] OR procedure*[ti])) OR ((biliopancreatic[ti] OR “bilio pancreatic”[ti]) AND (diversion*[ti] OR bypass*[ti])) OR (duodenal[ti] AND switch*[ti]) OR ((stomach[ti] OR gastric[ti]) AND (band*[ti] OR bypass*[ti] OR partiti*[ti] OR plicati*[ti] OR sleeve*[ti])) OR (sleeve[ti] AND gastrectom*[ti]) OR (roux[ti] AND y[ti])) AND ("Laparoscopy"[Mesh:NoExp] OR "Minimally Invasive Surgical Procedures"[Mesh:NoExp] OR laparoscop*[tiab] OR minimally invasive[tiab] OR minimal invasive[tiab] OR minimal access[tiab] OR minimal surg*[tiab] OR (minimal*[ti] AND invasive[ti]) OR (minimal[ti] AND (access[ti] OR surg*[ti])))) OR (lagb*[tiab] OR lrygb*[tiab] OR lap band*[tiab] OR lrygb*[tiab]) | [**13065**](https://www.ncbi.nlm.nih.gov/pubmed/?cmd=HistorySearch&querykey=25) |

### T3Q3: Antibiotic prophylaxis (5 SRs, 6 RCTs, 9 cohort studies) (9 November 2018)

| **Search** | **Query** | **Items found** |
| --- | --- | --- |
| [**#48**](https://www.ncbi.nlm.nih.gov/pubmed) | ((#45 AND #34) NOT (#46 OR #47)) | [**9**](https://www.ncbi.nlm.nih.gov/pubmed/?cmd=HistorySearch&querykey=48) |
| [**#47**](https://www.ncbi.nlm.nih.gov/pubmed) | ((#45 AND #32) NOT #46) | [**6**](https://www.ncbi.nlm.nih.gov/pubmed/?cmd=HistorySearch&querykey=47) |
| [**#46**](https://www.ncbi.nlm.nih.gov/pubmed) | (#45 AND #30) | [**5**](https://www.ncbi.nlm.nih.gov/pubmed/?cmd=HistorySearch&querykey=46) |
| [**#45**](https://www.ncbi.nlm.nih.gov/pubmed) | #44 NOT (("Adolescent"[Mesh] OR "Child"[Mesh] OR "Infant"[Mesh] OR adolescen*[tiab] OR child*[tiab] OR schoolchild*[tiab] OR infant*[tiab] OR girl*[tiab] OR boy*[tiab] OR teen[tiab] OR teens[tiab] OR teenager*[tiab] OR youth*[tiab] OR pediatr*[tiab] OR paediatr*[tiab] OR puber*[tiab]) NOT ("Adult"[Mesh] OR adult*[tiab] OR man[tiab] OR men[tiab] OR woman[tiab] OR women[tiab])) | [**22**](https://www.ncbi.nlm.nih.gov/pubmed/?cmd=HistorySearch&querykey=45) |
| [**#44**](https://www.ncbi.nlm.nih.gov/pubmed) | #43 NOT ("Animals"[Mesh] NOT "Humans"[Mesh]) AND ("2004"[Date - Entrez] : "3000"[Date - Entrez]) | [**23**](https://www.ncbi.nlm.nih.gov/pubmed/?cmd=HistorySearch&querykey=44) |
| [**#43**](https://www.ncbi.nlm.nih.gov/pubmed) | (#25 AND #42) | [**29**](https://www.ncbi.nlm.nih.gov/pubmed/?cmd=HistorySearch&querykey=43) |
| [**#42**](https://www.ncbi.nlm.nih.gov/pubmed) | (("Antimicrobial Stewardship"[Mesh] OR "Antibiotic Prophylaxis"[Mesh]) OR (("prevention and control"[Subheading] OR "Primary Prevention"[Mesh] OR preventi*[tiab] OR prophyla*[tiab] OR premedicati*[tiab] OR prescrib*[tiab] OR stewardship*[tiab]) AND ("Anti-Bacterial Agents"[Mesh] OR "Anti-Bacterial Agents" [Pharmacological Action] OR "Penicillins"[Mesh] OR antibacterial*[tiab] OR anti bacterial*[tiab] OR antibiotic*[tiab] OR antimicrobi*[tiab] OR anti microbi*[tiab]))) | [**115423**](https://www.ncbi.nlm.nih.gov/pubmed/?cmd=HistorySearch&querykey=42) |
| [**#34**](https://www.ncbi.nlm.nih.gov/pubmed) | "Epidemiologic Studies"[Mesh] OR "Prognosis"[Mesh] OR cohort[tiab] OR (case[tiab] AND (control[tiab] OR controll*[tiab] OR comparison[tiab] OR referent[tiab])) OR risk[tiab] OR causation[tiab] OR causal[tiab] OR "odds ratio"[tiab] OR etiol*[tiab] OR aetiol*[tiab] OR "natural history"[tiab] OR predict*[tiab] OR outcome[tiab] OR course[tiab] OR retrospect*[tiab] OR “follow up”[tiab] OR followup[tiab] OR prognos*[tiab] | [**6595486**](https://www.ncbi.nlm.nih.gov/pubmed/?cmd=HistorySearch&querykey=34) |
| [**#32**](https://www.ncbi.nlm.nih.gov/pubmed) | "Randomized Controlled Trial"[Publication Type] OR "Controlled Clinical Trial"[Publication Type] OR random*[tiab] OR placebo[tiab] OR "Drug Therapy"[Subheading] OR trial[tiab] OR groups[tiab] | [**4567282**](https://www.ncbi.nlm.nih.gov/pubmed/?cmd=HistorySearch&querykey=32) |
| [**#30**](https://www.ncbi.nlm.nih.gov/pubmed) | (review[tiab] OR "Review"[Publication Type] OR "Meta-Analysis as Topic"[Mesh] OR meta-analysis[tiab] OR "Meta-Analysis"[Publication Type]) NOT ("Letter"[Publication Type] OR "Editorial"[Publication Type] OR "Comment"[Publication Type]) | [**2991355**](https://www.ncbi.nlm.nih.gov/pubmed/?cmd=HistorySearch&querykey=30) |
| [**#25**](https://www.ncbi.nlm.nih.gov/pubmed) | (("Bariatric Surgery"[Mesh] OR "Obesity/surgery"[Mesh:NoExp] OR "Obesity, Abdominal/surgery"[Mesh] OR "Obesity, Metabolically Benign/surgery"[Mesh] OR "Obesity, Morbid/surgery"[Mesh] OR "Anastomosis, Roux-en-Y"[Mesh] OR "Biliopancreatic Diversion"[Mesh] OR "Overweight/surgery"[Mesh] OR "Gastrectomy"[Mesh] OR bariatric surg*[tiab] OR bariatric operati*[tiab] OR bariatric procedure*[tiab] OR bilio pancreatic bypass*[tiab] OR bilio pancreatic diversion*[tiab] OR biliopancreatic bypass*[tiab] OR biliopancreatic diversion*[tiab] OR duodenal switch*[tiab] OR gastric band*[tiab] OR stomach band*[tiab] OR gastric bypass*[tiab] OR gastric partiti*[tiab] OR gastric plicati*[tiab] OR gastroplast*[tiab] OR mgbp*[tiab] OR oagb*[tiab] OR obese surg*[tiab] OR weight loss surg*[tiab] OR obesity surg*[tiab] OR metabolic surg*[tiab] OR roux en y*[tiab] OR “roux y”[tiab] OR “roux n y”[tiab] OR “roux in y”[tiab] OR rya[tiab] OR rygb*[tiab] OR sadis[tiab] OR “sadi s”[tiab] OR sagb*[tiab] OR sleeve gastrectom*[tiab] OR gastric sleeve*[tiab] OR gastrectom*[tiab] OR ((bariatric[ti] OR obes*[ti] OR weight loss[ti] OR metabolic[ti]) AND (surg*[ti] OR operati*[ti] OR procedure*[ti])) OR ((biliopancreatic[ti] OR “bilio pancreatic”[ti]) AND (diversion*[ti] OR bypass*[ti])) OR (duodenal[ti] AND switch*[ti]) OR ((stomach[ti] OR gastric[ti]) AND (band*[ti] OR bypass*[ti] OR partiti*[ti] OR plicati*[ti] OR sleeve*[ti])) OR (sleeve[ti] AND gastrectom*[ti]) OR (roux[ti] AND y[ti])) AND ("Laparoscopy"[Mesh:NoExp] OR "Minimally Invasive Surgical Procedures"[Mesh:NoExp] OR laparoscop*[tiab] OR minimally invasive[tiab] OR minimal invasive[tiab] OR minimal access[tiab] OR minimal surg*[tiab] OR (minimal*[ti] AND invasive[ti]) OR (minimal[ti] AND (access[ti] OR surg*[ti])))) OR (lagb*[tiab] OR lrygb*[tiab] OR lap band*[tiab] OR lrygb*[tiab]) | [**13065**](https://www.ncbi.nlm.nih.gov/pubmed/?cmd=HistorySearch&querykey=25) |

### T3Q4: Intermittent pneumatic compression (IPC) (8 all study types)

| **Search** | **Query** | **Items found** |
| --- | --- | --- |
| [**#50**](https://www.ncbi.nlm.nih.gov/pubmed/advanced) | (#25 AND #49) | [**8**](https://www.ncbi.nlm.nih.gov/pubmed/?cmd=HistorySearch&querykey=50) |
| [**#49**](https://www.ncbi.nlm.nih.gov/pubmed/advanced) | "Intermittent Pneumatic Compression Devices"[Mesh] OR intermittent pneumatic compression*[tiab] OR pneumatic intermittent impulse*[tiab] OR pneumatic compression stocking*[tiab] OR pneumatic hose*[tiab] OR pneumatic compression hose*[tiab] | [**1107**](https://www.ncbi.nlm.nih.gov/pubmed/?cmd=HistorySearch&querykey=49) |
| [**#25**](https://www.ncbi.nlm.nih.gov/pubmed/advanced) | (("Bariatric Surgery"[Mesh] OR "Obesity/surgery"[Mesh:NoExp] OR "Obesity, Abdominal/surgery"[Mesh] OR "Obesity, Metabolically Benign/surgery"[Mesh] OR "Obesity, Morbid/surgery"[Mesh] OR "Anastomosis, Roux-en-Y"[Mesh] OR "Biliopancreatic Diversion"[Mesh] OR "Overweight/surgery"[Mesh] OR "Gastrectomy"[Mesh] OR bariatric surg*[tiab] OR bariatric operati*[tiab] OR bariatric procedure*[tiab] OR bilio pancreatic bypass*[tiab] OR bilio pancreatic diversion*[tiab] OR biliopancreatic bypass*[tiab] OR biliopancreatic diversion*[tiab] OR duodenal switch*[tiab] OR gastric band*[tiab] OR stomach band*[tiab] OR gastric bypass*[tiab] OR gastric partiti*[tiab] OR gastric plicati*[tiab] OR gastroplast*[tiab] OR mgbp*[tiab] OR oagb*[tiab] OR obese surg*[tiab] OR weight loss surg*[tiab] OR obesity surg*[tiab] OR metabolic surg*[tiab] OR roux en y*[tiab] OR “roux y”[tiab] OR “roux n y”[tiab] OR “roux in y”[tiab] OR rya[tiab] OR rygb*[tiab] OR sadis[tiab] OR “sadi s”[tiab] OR sagb*[tiab] OR sleeve gastrectom*[tiab] OR gastric sleeve*[tiab] OR gastrectom*[tiab] OR ((bariatric[ti] OR obes*[ti] OR weight loss[ti] OR metabolic[ti]) AND (surg*[ti] OR operati*[ti] OR procedure*[ti])) OR ((biliopancreatic[ti] OR “bilio pancreatic”[ti]) AND (diversion*[ti] OR bypass*[ti])) OR (duodenal[ti] AND switch*[ti]) OR ((stomach[ti] OR gastric[ti]) AND (band*[ti] OR bypass*[ti] OR partiti*[ti] OR plicati*[ti] OR sleeve*[ti])) OR (sleeve[ti] AND gastrectom*[ti]) OR (roux[ti] AND y[ti])) AND ("Laparoscopy"[Mesh:NoExp] OR "Minimally Invasive Surgical Procedures"[Mesh:NoExp] OR laparoscop*[tiab] OR minimally invasive[tiab] OR minimal invasive[tiab] OR minimal access[tiab] OR minimal surg*[tiab] OR (minimal*[ti] AND invasive[ti]) OR (minimal[ti] AND (access[ti] OR surg*[ti])))) OR (lagb*[tiab] OR lrygb*[tiab] OR lap band*[tiab] OR lrygb*[tiab]) | [**13065**](https://www.ncbi.nlm.nih.gov/pubmed/?cmd=HistorySearch&querykey=25) |

### T3Q5+Q6+Q7: Low molecular weight heparin (LMWH) (10 SRs, 8 RCTs, 13 cohort studies)

| **Search** | **Query** | **Items found** |
| --- | --- | --- |
| [**#57**](https://www.ncbi.nlm.nih.gov/pubmed) | ((#54 AND #34) NOT (#55 OR #56)) | [**13**](https://www.ncbi.nlm.nih.gov/pubmed/?cmd=HistorySearch&querykey=57) |
| [**#56**](https://www.ncbi.nlm.nih.gov/pubmed) | ((#54 AND #32) NOT #55) | [**8**](https://www.ncbi.nlm.nih.gov/pubmed/?cmd=HistorySearch&querykey=56) |
| [**#55**](https://www.ncbi.nlm.nih.gov/pubmed) | (#54 AND #30) | [**10**](https://www.ncbi.nlm.nih.gov/pubmed/?cmd=HistorySearch&querykey=55) |
| [**#54**](https://www.ncbi.nlm.nih.gov/pubmed) | (#53 NOT (("Adolescent"[Mesh] OR "Child"[Mesh] OR "Infant"[Mesh] OR adolescen*[tiab] OR child*[tiab] OR schoolchild*[tiab] OR infant*[tiab] OR girl*[tiab] OR boy*[tiab] OR teen[tiab] OR teens[tiab] OR teenager*[tiab] OR youth*[tiab] OR pediatr*[tiab] OR paediatr*[tiab] OR puber*[tiab]) NOT ("Adult"[Mesh] OR adult*[tiab] OR man[tiab] OR men[tiab] OR woman[tiab] OR women[tiab]))) | [**34**](https://www.ncbi.nlm.nih.gov/pubmed/?cmd=HistorySearch&querykey=54) |
| [**#53**](https://www.ncbi.nlm.nih.gov/pubmed) | (#52 NOT ("Animals"[Mesh] NOT "Humans"[Mesh]) AND ("2004"[Date - Entrez] : "3000"[Date - Entrez])) | [**35**](https://www.ncbi.nlm.nih.gov/pubmed/?cmd=HistorySearch&querykey=53) |
| [**#52**](https://www.ncbi.nlm.nih.gov/pubmed) | #25 AND #51 | [**36**](https://www.ncbi.nlm.nih.gov/pubmed/?cmd=HistorySearch&querykey=52) |
| [**#51**](https://www.ncbi.nlm.nih.gov/pubmed) | "Heparin, Low-Molecular-Weight"[Mesh] OR lmwh* OR low molecular weight heparin* OR tedelparin*[tiab] OR dalteparin*[tiab] OR fragmin*[tiab] OR enoxaparin*[tiab] OR lovenox[tiab] OR clexan*[tiab] OR nadroparin*[tiab] OR fraxiparin*[tiab] | [**18117**](https://www.ncbi.nlm.nih.gov/pubmed/?cmd=HistorySearch&querykey=51) |
| [**#34**](https://www.ncbi.nlm.nih.gov/pubmed) | "Epidemiologic Studies"[Mesh] OR "Prognosis"[Mesh] OR cohort[tiab] OR (case[tiab] AND (control[tiab] OR controll*[tiab] OR comparison[tiab] OR referent[tiab])) OR risk[tiab] OR causation[tiab] OR causal[tiab] OR "odds ratio"[tiab] OR etiol*[tiab] OR aetiol*[tiab] OR "natural history"[tiab] OR predict*[tiab] OR outcome[tiab] OR course[tiab] OR retrospect*[tiab] OR “follow up”[tiab] OR followup[tiab] OR prognos*[tiab] | [**6595486**](https://www.ncbi.nlm.nih.gov/pubmed/?cmd=HistorySearch&querykey=34) |
| [**#32**](https://www.ncbi.nlm.nih.gov/pubmed) | "Randomized Controlled Trial"[Publication Type] OR "Controlled Clinical Trial"[Publication Type] OR random*[tiab] OR placebo[tiab] OR "Drug Therapy"[Subheading] OR trial[tiab] OR groups[tiab] | [**4567282**](https://www.ncbi.nlm.nih.gov/pubmed/?cmd=HistorySearch&querykey=32) |
| [**#30**](https://www.ncbi.nlm.nih.gov/pubmed) | (review[tiab] OR "Review"[Publication Type] OR "Meta-Analysis as Topic"[Mesh] OR meta-analysis[tiab] OR "Meta-Analysis"[Publication Type]) NOT ("Letter"[Publication Type] OR "Editorial"[Publication Type] OR "Comment"[Publication Type]) | [**2991355**](https://www.ncbi.nlm.nih.gov/pubmed/?cmd=HistorySearch&querykey=30) |
| [**#25**](https://www.ncbi.nlm.nih.gov/pubmed) | (("Bariatric Surgery"[Mesh] OR "Obesity/surgery"[Mesh:NoExp] OR "Obesity, Abdominal/surgery"[Mesh] OR "Obesity, Metabolically Benign/surgery"[Mesh] OR "Obesity, Morbid/surgery"[Mesh] OR "Anastomosis, Roux-en-Y"[Mesh] OR "Biliopancreatic Diversion"[Mesh] OR "Overweight/surgery"[Mesh] OR "Gastrectomy"[Mesh] OR bariatric surg*[tiab] OR bariatric operati*[tiab] OR bariatric procedure*[tiab] OR bilio pancreatic bypass*[tiab] OR bilio pancreatic diversion*[tiab] OR biliopancreatic bypass*[tiab] OR biliopancreatic diversion*[tiab] OR duodenal switch*[tiab] OR gastric band*[tiab] OR stomach band*[tiab] OR gastric bypass*[tiab] OR gastric partiti*[tiab] OR gastric plicati*[tiab] OR gastroplast*[tiab] OR mgbp*[tiab] OR oagb*[tiab] OR obese surg*[tiab] OR weight loss surg*[tiab] OR obesity surg*[tiab] OR metabolic surg*[tiab] OR roux en y*[tiab] OR “roux y”[tiab] OR “roux n y”[tiab] OR “roux in y”[tiab] OR rya[tiab] OR rygb*[tiab] OR sadis[tiab] OR “sadi s”[tiab] OR sagb*[tiab] OR sleeve gastrectom*[tiab] OR gastric sleeve*[tiab] OR gastrectom*[tiab] OR ((bariatric[ti] OR obes*[ti] OR weight loss[ti] OR metabolic[ti]) AND (surg*[ti] OR operati*[ti] OR procedure*[ti])) OR ((biliopancreatic[ti] OR “bilio pancreatic”[ti]) AND (diversion*[ti] OR bypass*[ti])) OR (duodenal[ti] AND switch*[ti]) OR ((stomach[ti] OR gastric[ti]) AND (band*[ti] OR bypass*[ti] OR partiti*[ti] OR plicati*[ti] OR sleeve*[ti])) OR (sleeve[ti] AND gastrectom*[ti]) OR (roux[ti] AND y[ti])) AND ("Laparoscopy"[Mesh:NoExp] OR "Minimally Invasive Surgical Procedures"[Mesh:NoExp] OR laparoscop*[tiab] OR minimally invasive[tiab] OR minimal invasive[tiab] OR minimal access[tiab] OR minimal surg*[tiab] OR (minimal*[ti] AND invasive[ti]) OR (minimal[ti] AND (access[ti] OR surg*[ti])))) OR (lagb*[tiab] OR lrygb*[tiab] OR lap band*[tiab] OR lrygb*[tiab]) | [**13065**](https://www.ncbi.nlm.nih.gov/pubmed/?cmd=HistorySearch&querykey=25) |

### T3 Q8+Q9: Inferior vena cava filter (IVCF) (5 SRs, 3 RCTs, 11 cohort studies)

| **Search** | **Query** | **Items found** |
| --- | --- | --- |
| [**#64**](https://www.ncbi.nlm.nih.gov/pubmed) | (#61 AND #34) NOT (#62 OR #63) | [**11**](https://www.ncbi.nlm.nih.gov/pubmed/?cmd=HistorySearch&querykey=64) |
| [**#63**](https://www.ncbi.nlm.nih.gov/pubmed) | (#61 AND #32) NOT #62 | [**3**](https://www.ncbi.nlm.nih.gov/pubmed/?cmd=HistorySearch&querykey=63) |
| [**#62**](https://www.ncbi.nlm.nih.gov/pubmed) | #61 AND #30 | [**5**](https://www.ncbi.nlm.nih.gov/pubmed/?cmd=HistorySearch&querykey=62) |
| [**#61**](https://www.ncbi.nlm.nih.gov/pubmed) | #60 NOT (("Adolescent"[Mesh] OR "Child"[Mesh] OR "Infant"[Mesh] OR adolescen*[tiab] OR child*[tiab] OR schoolchild*[tiab] OR infant*[tiab] OR girl*[tiab] OR boy*[tiab] OR teen[tiab] OR teens[tiab] OR teenager*[tiab] OR youth*[tiab] OR pediatr*[tiab] OR paediatr*[tiab] OR puber*[tiab]) NOT ("Adult"[Mesh] OR adult*[tiab] OR man[tiab] OR men[tiab] OR woman[tiab] OR women[tiab])) | [**22**](https://www.ncbi.nlm.nih.gov/pubmed/?cmd=HistorySearch&querykey=61) |
| [**#60**](https://www.ncbi.nlm.nih.gov/pubmed) | #59 NOT ("Animals"[Mesh] NOT "Humans"[Mesh]) AND ("2004"[Date - Entrez] : "3000"[Date - Entrez]) | [**22**](https://www.ncbi.nlm.nih.gov/pubmed/?cmd=HistorySearch&querykey=60) |
| [**#59**](https://www.ncbi.nlm.nih.gov/pubmed) | (#25 AND #58) | [**22**](https://www.ncbi.nlm.nih.gov/pubmed/?cmd=HistorySearch&querykey=59) |
| [**#58**](https://www.ncbi.nlm.nih.gov/pubmed) | "Vena Cava Filters"[Mesh] OR vena cava filter*[tiab] OR umbrella filter*[tiab] OR ivcf*[tiab] OR vcf*[tiab] | [**5206**](https://www.ncbi.nlm.nih.gov/pubmed/?cmd=HistorySearch&querykey=58) |
| [**#34**](https://www.ncbi.nlm.nih.gov/pubmed) | "Epidemiologic Studies"[Mesh] OR "Prognosis"[Mesh] OR cohort[tiab] OR (case[tiab] AND (control[tiab] OR controll*[tiab] OR comparison[tiab] OR referent[tiab])) OR risk[tiab] OR causation[tiab] OR causal[tiab] OR "odds ratio"[tiab] OR etiol*[tiab] OR aetiol*[tiab] OR "natural history"[tiab] OR predict*[tiab] OR outcome[tiab] OR course[tiab] OR retrospect*[tiab] OR “follow up”[tiab] OR followup[tiab] OR prognos*[tiab] | [**6595486**](https://www.ncbi.nlm.nih.gov/pubmed/?cmd=HistorySearch&querykey=34) |
| [**#32**](https://www.ncbi.nlm.nih.gov/pubmed) | "Randomized Controlled Trial"[Publication Type] OR "Controlled Clinical Trial"[Publication Type] OR random*[tiab] OR placebo[tiab] OR "Drug Therapy"[Subheading] OR trial[tiab] OR groups[tiab] | [**4567282**](https://www.ncbi.nlm.nih.gov/pubmed/?cmd=HistorySearch&querykey=32) |
| [**#30**](https://www.ncbi.nlm.nih.gov/pubmed) | (review[tiab] OR "Review"[Publication Type] OR "Meta-Analysis as Topic"[Mesh] OR meta-analysis[tiab] OR "Meta-Analysis"[Publication Type]) NOT ("Letter"[Publication Type] OR "Editorial"[Publication Type] OR "Comment"[Publication Type]) | [**2991355**](https://www.ncbi.nlm.nih.gov/pubmed/?cmd=HistorySearch&querykey=30) |
| [**#25**](https://www.ncbi.nlm.nih.gov/pubmed) | (("Bariatric Surgery"[Mesh] OR "Obesity/surgery"[Mesh:NoExp] OR "Obesity, Abdominal/surgery"[Mesh] OR "Obesity, Metabolically Benign/surgery"[Mesh] OR "Obesity, Morbid/surgery"[Mesh] OR "Anastomosis, Roux-en-Y"[Mesh] OR "Biliopancreatic Diversion"[Mesh] OR "Overweight/surgery"[Mesh] OR "Gastrectomy"[Mesh] OR bariatric surg*[tiab] OR bariatric operati*[tiab] OR bariatric procedure*[tiab] OR bilio pancreatic bypass*[tiab] OR bilio pancreatic diversion*[tiab] OR biliopancreatic bypass*[tiab] OR biliopancreatic diversion*[tiab] OR duodenal switch*[tiab] OR gastric band*[tiab] OR stomach band*[tiab] OR gastric bypass*[tiab] OR gastric partiti*[tiab] OR gastric plicati*[tiab] OR gastroplast*[tiab] OR mgbp*[tiab] OR oagb*[tiab] OR obese surg*[tiab] OR weight loss surg*[tiab] OR obesity surg*[tiab] OR metabolic surg*[tiab] OR roux en y*[tiab] OR “roux y”[tiab] OR “roux n y”[tiab] OR “roux in y”[tiab] OR rya[tiab] OR rygb*[tiab] OR sadis[tiab] OR “sadi s”[tiab] OR sagb*[tiab] OR sleeve gastrectom*[tiab] OR gastric sleeve*[tiab] OR gastrectom*[tiab] OR ((bariatric[ti] OR obes*[ti] OR weight loss[ti] OR metabolic[ti]) AND (surg*[ti] OR operati*[ti] OR procedure*[ti])) OR ((biliopancreatic[ti] OR “bilio pancreatic”[ti]) AND (diversion*[ti] OR bypass*[ti])) OR (duodenal[ti] AND switch*[ti]) OR ((stomach[ti] OR gastric[ti]) AND (band*[ti] OR bypass*[ti] OR partiti*[ti] OR plicati*[ti] OR sleeve*[ti])) OR (sleeve[ti] AND gastrectom*[ti]) OR (roux[ti] AND y[ti])) AND ("Laparoscopy"[Mesh:NoExp] OR "Minimally Invasive Surgical Procedures"[Mesh:NoExp] OR laparoscop*[tiab] OR minimally invasive[tiab] OR minimal invasive[tiab] OR minimal access[tiab] OR minimal surg*[tiab] OR (minimal*[ti] AND invasive[ti]) OR (minimal[ti] AND (access[ti] OR surg*[ti])))) OR (lagb*[tiab] OR lrygb*[tiab] OR lap band*[tiab] OR lrygb*[tiab]) | [**13065**](https://www.ncbi.nlm.nih.gov/pubmed/?cmd=HistorySearch&querykey=25) |

### T3Q10: ERAS (18 SRs, 39 RCTs, 38 cohort studies) (11 February 2019)

| **Search** | **Query** | **Items found** |
| --- | --- | --- |
| [**#11**](https://www.ncbi.nlm.nih.gov/pubmed) | ((#4 AND #10) NOT (#9 OR #7)) | [**38**](https://www.ncbi.nlm.nih.gov/pubmed/?cmd=HistorySearch&querykey=11) |
| [**#10**](https://www.ncbi.nlm.nih.gov/pubmed) | ("Epidemiologic Studies"[Mesh] OR "Prognosis"[Mesh] OR cohort[tiab] OR (case[tiab] AND (control[tiab] OR controll*[tiab] OR comparison[tiab] OR referent[tiab])) OR risk[tiab] OR causation[tiab] OR causal[tiab] OR "odds ratio"[tiab] OR etiol*[tiab] OR aetiol*[tiab] OR "natural history"[tiab] OR predict*[tiab] OR outcome[tiab] OR course[tiab] OR retrospect*[tiab] OR “follow up”[tiab] OR followup[tiab] OR prognos*[tiab]) | [**6709293**](https://www.ncbi.nlm.nih.gov/pubmed/?cmd=HistorySearch&querykey=10) |
| [**#9**](https://www.ncbi.nlm.nih.gov/pubmed) | (#4 AND #8 NOT #7) | [**39**](https://www.ncbi.nlm.nih.gov/pubmed/?cmd=HistorySearch&querykey=9) |
| [**#8**](https://www.ncbi.nlm.nih.gov/pubmed) | ("Randomized Controlled Trial"[Publication Type] OR "Controlled Clinical Trial"[Publication Type] OR random*[tiab] OR placebo[tiab] OR "Drug Therapy"[Subheading] OR trial[tiab] OR groups[tiab]) | [**4631826**](https://www.ncbi.nlm.nih.gov/pubmed/?cmd=HistorySearch&querykey=8) |
| [**#7**](https://www.ncbi.nlm.nih.gov/pubmed) | (#5 AND #6) | [**18**](https://www.ncbi.nlm.nih.gov/pubmed/?cmd=HistorySearch&querykey=7) |
| [**#6**](https://www.ncbi.nlm.nih.gov/pubmed) | ((review[tiab] OR "Review"[Publication Type] OR "Meta-Analysis as Topic"[Mesh] OR meta-analysis[tiab] OR "Meta-Analysis"[Publication Type]) NOT ("Letter"[Publication Type] OR "Editorial"[Publication Type] OR "Comment"[Publication Type])) | [**3035678**](https://www.ncbi.nlm.nih.gov/pubmed/?cmd=HistorySearch&querykey=6) |
| [**#5**](https://www.ncbi.nlm.nih.gov/pubmed) | (#4 NOT (("Adolescent"[Mesh] OR "Child"[Mesh] OR "Infant"[Mesh] OR adolescen*[tiab] OR child*[tiab] OR schoolchild*[tiab] OR infant*[tiab] OR girl*[tiab] OR boy*[tiab] OR teen[tiab] OR teens[tiab] OR teenager*[tiab] OR youth*[tiab] OR pediatr*[tiab] OR paediatr*[tiab] OR puber*[tiab]) NOT ("Adult"[Mesh] OR adult*[tiab] OR man[tiab] OR men[tiab] OR woman[tiab] OR women[tiab]))) | [**107**](https://www.ncbi.nlm.nih.gov/pubmed/?cmd=HistorySearch&querykey=5) |
| [**#4**](https://www.ncbi.nlm.nih.gov/pubmed) | (#3 NOT ("Animals"[Mesh] NOT "Humans"[Mesh]) AND ("2004"[Date - Entrez] : "3000"[Date - Entrez])) | [**107**](https://www.ncbi.nlm.nih.gov/pubmed/?cmd=HistorySearch&querykey=4) |
| [**#3**](https://www.ncbi.nlm.nih.gov/pubmed) | (#1 AND #2) | [**107**](https://www.ncbi.nlm.nih.gov/pubmed/?cmd=HistorySearch&querykey=3) |
| [**#2**](https://www.ncbi.nlm.nih.gov/pubmed) | (enhanced recovery after surgery[tiab] OR ERAS protocol*[tiab] OR ERAS program*[tiab] OR enhanced recovery protocol*[tiab] OR fast-track*[tiab] OR fasttrack*[tiab] OR “early recovery protocol”[tiab] OR enhanced recovery program*[tiab] OR early recovery program*[tiab]) | [**5149**](https://www.ncbi.nlm.nih.gov/pubmed/?cmd=HistorySearch&querykey=2) |
| [**#1**](https://www.ncbi.nlm.nih.gov/pubmed) | ((("Bariatric Surgery"[Mesh] OR "Obesity/surgery"[Mesh:NoExp] OR "Obesity, Abdominal/surgery"[Mesh] OR "Obesity, Metabolically Benign/surgery"[Mesh] OR "Obesity, Morbid/surgery"[Mesh] OR "Anastomosis, Roux-en-Y"[Mesh] OR "Biliopancreatic Diversion"[Mesh] OR "Overweight/surgery"[Mesh] OR "Gastrectomy"[Mesh] OR bariatric surg*[tiab] OR bariatric operati*[tiab] OR bariatric procedure*[tiab] OR bilio pancreatic bypass*[tiab] OR bilio pancreatic diversion*[tiab] OR biliopancreatic bypass*[tiab] OR biliopancreatic diversion*[tiab] OR duodenal switch*[tiab] OR gastric band*[tiab] OR stomach band*[tiab] OR gastric bypass*[tiab] OR gastric partiti*[tiab] OR gastric plicati*[tiab] OR gastroplast*[tiab] OR mgbp*[tiab] OR oagb*[tiab] OR obese surg*[tiab] OR weight loss surg*[tiab] OR obesity surg*[tiab] OR metabolic surg*[tiab] OR roux en y*[tiab] OR “roux y”[tiab] OR “roux n y”[tiab] OR “roux in y”[tiab] OR rya[tiab] OR rygb*[tiab] OR sadis[tiab] OR “sadi s”[tiab] OR sagb*[tiab] OR sleeve gastrectom*[tiab] OR gastric sleeve*[tiab] OR gastrectom*[tiab] OR ((bariatric[ti] OR obes*[ti] OR weight loss[ti] OR metabolic[ti]) AND (surg*[ti] OR operati*[ti] OR procedure*[ti])) OR ((biliopancreatic[ti] OR “bilio pancreatic”[ti]) AND (diversion*[ti] OR bypass*[ti])) OR (duodenal[ti] AND switch*[ti]) OR ((stomach[ti] OR gastric[ti]) AND (band*[ti] OR bypass*[ti] OR partiti*[ti] OR plicati*[ti] OR sleeve*[ti])) OR (sleeve[ti] AND gastrectom*[ti]) OR (roux[ti] AND y[ti])) AND ("Laparoscopy"[Mesh:NoExp] OR "Minimally Invasive Surgical Procedures"[Mesh:NoExp] OR laparoscop*[tiab] OR minimally invasive[tiab] OR minimal invasive[tiab] OR minimal access[tiab] OR minimal surg*[tiab] OR (minimal*[ti] AND invasive[ti]) OR (minimal[ti] AND (access[ti] OR surg*[ti])))) OR (lagb*[tiab] OR lrygb*[tiab] OR lap band*[tiab] OR lrygb*[tiab])) | [**13393**](https://www.ncbi.nlm.nih.gov/pubmed/?cmd=HistorySearch&querykey=1) |

## Embase.com (9 November 2018 + 11 February 2019)

### T3Q1: OSA + CPAP (61 SRs, 15 RCTs, 71 cohort studies)

| **No.** | **Query** | **Results** |
| --- | --- | --- |
| **#12** | #6 AND #11 NOT (#8 OR #10) | **71** |
| **#11** | 'epidemiology'/de OR (((cohort OR case) NEAR/3 (control OR controll* OR comparison OR referent)):ti,ab,kw) OR risk:ti,ab,kw OR causation:ti,ab,kw OR causal:ti,ab,kw OR 'odds ratio':ti,ab,kw OR etiol*:ti,ab,kw OR aetiol*:ti,ab,kw OR 'natural history':ti,ab,kw OR outcome:ti,ab,kw OR course:ti,ab,kw OR retrospect*:ti,ab,kw OR 'follow up':ti,ab,kw OR followup:ti,ab,kw OR predict*:ti,ab,kw OR prognos*:ti,ab,kw | **7313214** |
| **#10** | #6 AND #9 NOT #8 | **15** |
| **#9** | 'clinical study':ti,ab,kw AND 'trial':ti,ab,kw OR 'clinical trial'/exp OR random*:ti,ab,kw | **2191510** |
| **#8** | #6 AND #7 | **61** |
| **#7** | 'meta-analysis':ti,ab,kw OR 'meta analysis'/exp OR 'review'/exp OR review:ti,ab,kw | **3431289** |
| **#6** | #5 NOT (('adolescent'/exp OR 'child'/exp OR adolescent*:ti,ab OR child*:ti,ab OR schoolchild*:ti,ab OR infant*:ti,ab OR girl*:ti,ab OR boy*:ti,ab OR teen:ti,ab OR teens:ti,ab OR teenager*:ti,ab OR youth*:ti,ab OR pediatr*:ti,ab OR paediatr*:ti,ab OR puber*:ti,ab) NOT ('adult'/exp OR 'aged'/exp OR 'middle aged'/exp OR adult*:ti,ab OR man:ti,ab OR men:ti,ab OR woman:ti,ab OR women:ti,ab)) | **189** |
| **#5** | #4 NOT ('conference abstract'/it OR 'conference paper'/it OR 'letter'/it OR 'note'/it) | **201** |
| **#4** | #3 NOT ([animals]/lim NOT [humans]/lim) AND [1-1-2004]/sd | **314** |
| **#3** | #1 AND #2 | **329** |
| **#2** | ('sleep disordered breathing'/exp OR 'sleep apnea*':ti,ab,kw OR 'nocturnal apnea*':ti,ab,kw OR 'sleep hypopnea*':ti,ab,kw OR 'sleep apnoea*':ti,ab,kw OR 'nocturnal apnoea*':ti,ab,kw OR 'sleep hypopnoea*':ti,ab,kw OR 'sleep-disordered breathing':ti,ab,kw OR osahs:ti,ab,kw OR osas:ti,ab,kw) AND ('positive end expiratory pressure'/exp OR 'cpap device'/exp OR 'continuous positive airway pressure*':ti,ab,kw OR cpap*:ti,ab,kw OR ncpap*:ti,ab,kw OR 'airway pressure release ventilati*':ti,ab,kw OR aprv*:ti,ab,kw) | **16988** |
| **#1** | ('obesity'/exp/mj AND 'surgery'/lnk OR 'bariatric surgery'/exp/mj OR 'roux y anastomosis'/exp/mj OR 'gastric bypass surgery'/exp/mj OR ((('bilio pancreatic' OR biliopancreatic) NEAR/3 (diversion* OR bypass*)):ti,kw) OR ((duodenal NEAR/3 switch*):ti,kw) OR (((stomach OR gastric) NEAR/3 (band* OR bypass* OR partiti* OR plicati* OR sleeve*)):ti,kw) OR ((sleeve NEAR/3 gastrectom*):ti,kw) OR ((roux NEAR/2 y):ti,kw) OR gastroplast*:ti,kw OR mgbp*:ti,kw OR oagb*:ti,kw OR rya:ti,kw OR rygb*:ti,kw OR sadis:ti,kw OR 'sadi s':ti,kw OR (((bariatric OR obes* OR 'weight loss' OR metabolic) NEAR/3 (surg* OR operati* OR procedure*)):ti,kw) OR sagb*:ti,kw) AND ('laparoscopy'/de OR 'laparoendoscopic single site surgery'/exp OR 'laparoscopic surgery'/exp OR 'minimally invasive procedure'/exp OR 'minimally invasive surgery'/exp OR laparoscop*:ti,ab,kw OR ((minimal* NEAR/3 invasive):ti,ab,kw) OR access:ti,ab,kw OR surg*:ti,ab,kw) OR 'laparoscopic sleeve gastrectomy'/exp OR lagb*:ti,ab,kw OR 'lap band*':ti,ab,kw OR lrygb*:ti,ab,kw | **40535** |

### T3Q2: Multimodal analgesia (10 SRs, 11 RCTs, 7 cohort studies)

| **No.** | **Query** | **Results** |
| --- | --- | --- |
| **#12** | #9 AND #4 NOT (#10 OR #11) | **7** |
| **#11** | #9 AND #3 NOT #10 | **11** |
| **#10** | #2 AND #9 | **10** |
| **#9** | #8 NOT (('adolescent'/exp OR 'child'/exp OR adolescent*:ti,ab OR child*:ti,ab OR schoolchild*:ti,ab OR infant*:ti,ab OR girl*:ti,ab OR boy*:ti,ab OR teen:ti,ab OR teens:ti,ab OR teenager*:ti,ab OR youth*:ti,ab OR pediatr*:ti,ab OR paediatr*:ti,ab OR puber*:ti,ab) NOT ('adult'/exp OR 'aged'/exp OR 'middle aged'/exp OR adult*:ti,ab OR man:ti,ab OR men:ti,ab OR woman:ti,ab OR women:ti,ab)) | **33** |
| **#8** | #7 NOT ('conference abstract'/it OR 'conference paper'/it OR 'letter'/it OR 'note'/it) | **33** |
| **#7** | #6 NOT ([animals]/lim NOT [humans]/lim) AND [1-1-2004]/sd | **67** |
| **#6** | #1 AND #5 | **69** |
| **#5** | multimodal*:ti,ab,kw AND ('analgesia'/exp OR 'analgesic agent'/exp OR 'anesthesia'/exp OR 'anesthetic agent'/exp OR analgesi*:ti,ab,kw OR anesthe*:ti,ab,kw OR anaesthe*:ti,ab,kw OR 'nerve block*':ti,ab,kw) | **6926** |
| **#4** | 'epidemiology'/de OR (((cohort OR case) NEAR/3 (control OR controll* OR comparison OR referent)):ti,ab,kw) OR risk:ti,ab,kw OR causation:ti,ab,kw OR causal:ti,ab,kw OR 'odds ratio':ti,ab,kw OR etiol*:ti,ab,kw OR aetiol*:ti,ab,kw OR 'natural history':ti,ab,kw OR outcome:ti,ab,kw OR course:ti,ab,kw OR retrospect*:ti,ab,kw OR 'follow up':ti,ab,kw OR followup:ti,ab,kw OR predict*:ti,ab,kw OR prognos*:ti,ab,kw | **7313214** |
| **#3** | 'clinical study':ti,ab,kw AND 'trial':ti,ab,kw OR 'clinical trial'/exp OR random*:ti,ab,kw | **2191510** |
| **#2** | 'meta-analysis':ti,ab,kw OR 'meta analysis'/exp OR 'review'/exp OR review:ti,ab,kw | **3431289** |
| **#1** | ('obesity'/exp/mj AND 'surgery'/lnk OR 'bariatric surgery'/exp/mj OR 'roux y anastomosis'/exp/mj OR 'gastric bypass surgery'/exp/mj OR ((('bilio pancreatic' OR biliopancreatic) NEAR/3 (diversion* OR bypass*)):ti,kw) OR ((duodenal NEAR/3 switch*):ti,kw) OR (((stomach OR gastric) NEAR/3 (band* OR bypass* OR partiti* OR plicati* OR sleeve*)):ti,kw) OR ((sleeve NEAR/3 gastrectom*):ti,kw) OR ((roux NEAR/2 y):ti,kw) OR gastroplast*:ti,kw OR mgbp*:ti,kw OR oagb*:ti,kw OR rya:ti,kw OR rygb*:ti,kw OR sadis:ti,kw OR 'sadi s':ti,kw OR (((bariatric OR obes* OR 'weight loss' OR metabolic) NEAR/3 (surg* OR operati* OR procedure*)):ti,kw) OR sagb*:ti,kw) AND ('laparoscopy'/de OR 'laparoendoscopic single site surgery'/exp OR 'laparoscopic surgery'/exp OR 'minimally invasive procedure'/exp OR 'minimally invasive surgery'/exp OR laparoscop*:ti,ab,kw OR ((minimal* NEAR/3 invasive):ti,ab,kw) OR access:ti,ab,kw OR surg*:ti,ab,kw) OR 'laparoscopic sleeve gastrectomy'/exp OR lagb*:ti,ab,kw OR 'lap band*':ti,ab,kw OR lrygb*:ti,ab,kw | **40535** |

### T3Q3: Antibiotic prophylaxis (57 SRs, 27 RCTs, 52 cohort studies)

| **No.** | **Query** | **Results** |
| --- | --- | --- |
| **#12** | #9 AND #4 NOT (#10 OR #11) | **52** |
| **#11** | #9 AND #3 NOT #10 | **27** |
| **#10** | #2 AND #9 | **57** |
| **#9** | #8 NOT (('adolescent'/exp OR 'child'/exp OR adolescent*:ti,ab OR child*:ti,ab OR schoolchild*:ti,ab OR infant*:ti,ab OR girl*:ti,ab OR boy*:ti,ab OR teen:ti,ab OR teens:ti,ab OR teenager*:ti,ab OR youth*:ti,ab OR pediatr*:ti,ab OR paediatr*:ti,ab OR puber*:ti,ab) NOT ('adult'/exp OR 'aged'/exp OR 'middle aged'/exp OR adult*:ti,ab OR man:ti,ab OR men:ti,ab OR woman:ti,ab OR women:ti,ab)) | **171** |
| **#8** | #7 NOT ('conference abstract'/it OR 'conference paper'/it OR 'letter'/it OR 'note'/it) | **176** |
| **#7** | #6 NOT ([animals]/lim NOT [humans]/lim) AND [1-1-2004]/sd | **235** |
| **#6** | #1 AND #5 | **263** |
| **#5** | 'antibiotic prophylaxis'/exp OR 'antimicrobial stewardship'/exp OR (('perioperative period'/de OR 'prevention'/de OR 'secondary prevention'/exp OR 'prophylaxis'/de OR 'primary prevention'/exp OR preventi*:ti,ab,kw OR prophyla*:ti,ab,kw OR premedicati*:ti,ab,kw OR prescrib*:ti,ab,kw OR stewardship*:ti,ab,kw) AND ('antiinfective agent'/exp OR antibacterial*:ti,ab,kw OR 'anti bacterial*':ti,ab,kw OR 'anti microbi*':ti,ab,kw OR antimicrobi*:ti,ab,kw OR antibiotic*:ti,ab,kw)) | **284691** |
| **#4** | 'epidemiology'/de OR (((cohort OR case) NEAR/3 (control OR controll* OR comparison OR referent)):ti,ab,kw) OR risk:ti,ab,kw OR causation:ti,ab,kw OR causal:ti,ab,kw OR 'odds ratio':ti,ab,kw OR etiol*:ti,ab,kw OR aetiol*:ti,ab,kw OR 'natural history':ti,ab,kw OR outcome:ti,ab,kw OR course:ti,ab,kw OR retrospect*:ti,ab,kw OR 'follow up':ti,ab,kw OR followup:ti,ab,kw OR predict*:ti,ab,kw OR prognos*:ti,ab,kw | **7313214** |
| **#3** | 'clinical study':ti,ab,kw AND 'trial':ti,ab,kw OR 'clinical trial'/exp OR random*:ti,ab,kw | **2191510** |
| **#2** | 'meta-analysis':ti,ab,kw OR 'meta analysis'/exp OR 'review'/exp OR review:ti,ab,kw | **3431289** |
| **#1** | ('obesity'/exp/mj AND 'surgery'/lnk OR 'bariatric surgery'/exp/mj OR 'roux y anastomosis'/exp/mj OR 'gastric bypass surgery'/exp/mj OR ((('bilio pancreatic' OR biliopancreatic) NEAR/3 (diversion* OR bypass*)):ti,kw) OR ((duodenal NEAR/3 switch*):ti,kw) OR (((stomach OR gastric) NEAR/3 (band* OR bypass* OR partiti* OR plicati* OR sleeve*)):ti,kw) OR ((sleeve NEAR/3 gastrectom*):ti,kw) OR ((roux NEAR/2 y):ti,kw) OR gastroplast*:ti,kw OR mgbp*:ti,kw OR oagb*:ti,kw OR rya:ti,kw OR rygb*:ti,kw OR sadis:ti,kw OR 'sadi s':ti,kw OR (((bariatric OR obes* OR 'weight loss' OR metabolic) NEAR/3 (surg* OR operati* OR procedure*)):ti,kw) OR sagb*:ti,kw) AND ('laparoscopy'/de OR 'laparoendoscopic single site surgery'/exp OR 'laparoscopic surgery'/exp OR 'minimally invasive procedure'/exp OR 'minimally invasive surgery'/exp OR laparoscop*:ti,ab,kw OR ((minimal* NEAR/3 invasive):ti,ab,kw) OR access:ti,ab,kw OR surg*:ti,ab,kw) OR 'laparoscopic sleeve gastrectomy'/exp OR lagb*:ti,ab,kw OR 'lap band*':ti,ab,kw OR lrygb*:ti,ab,kw | **40535** |

### T3Q4: Intermittent pneumatic compression (IPC) (18 all study types)

| **No.** | **Query** | **Results** |
| --- | --- | --- |
| **#6** | #1 AND #5 | **18** |
| **#5** | 'intermittent pneumatic compression'/exp OR 'intermittent pneumatic compression*':ti,ab,kw OR 'pneumatic intermittent impulse*':ti,ab,kw OR 'pneumatic compression stocking*':ti,ab,kw OR 'pneumatic hose*':ti,ab,kw OR 'pneumatic compression hose*':ti,ab,kw | **1045** |
| **#4** | 'epidemiology'/de OR (((cohort OR case) NEAR/3 (control OR controll* OR comparison OR referent)):ti,ab,kw) OR risk:ti,ab,kw OR causation:ti,ab,kw OR causal:ti,ab,kw OR 'odds ratio':ti,ab,kw OR etiol*:ti,ab,kw OR aetiol*:ti,ab,kw OR 'natural history':ti,ab,kw OR outcome:ti,ab,kw OR course:ti,ab,kw OR retrospect*:ti,ab,kw OR 'follow up':ti,ab,kw OR followup:ti,ab,kw OR predict*:ti,ab,kw OR prognos*:ti,ab,kw | **7313214** |
| **#3** | 'clinical study':ti,ab,kw AND 'trial':ti,ab,kw OR 'clinical trial'/exp OR random*:ti,ab,kw | **2191510** |
| **#2** | 'meta-analysis':ti,ab,kw OR 'meta analysis'/exp OR 'review'/exp OR review:ti,ab,kw | **3431289** |
| **#1** | ('obesity'/exp/mj AND 'surgery'/lnk OR 'bariatric surgery'/exp/mj OR 'roux y anastomosis'/exp/mj OR 'gastric bypass surgery'/exp/mj OR ((('bilio pancreatic' OR biliopancreatic) NEAR/3 (diversion* OR bypass*)):ti,kw) OR ((duodenal NEAR/3 switch*):ti,kw) OR (((stomach OR gastric) NEAR/3 (band* OR bypass* OR partiti* OR plicati* OR sleeve*)):ti,kw) OR ((sleeve NEAR/3 gastrectom*):ti,kw) OR ((roux NEAR/2 y):ti,kw) OR gastroplast*:ti,kw OR mgbp*:ti,kw OR oagb*:ti,kw OR rya:ti,kw OR rygb*:ti,kw OR sadis:ti,kw OR 'sadi s':ti,kw OR (((bariatric OR obes* OR 'weight loss' OR metabolic) NEAR/3 (surg* OR operati* OR procedure*)):ti,kw) OR sagb*:ti,kw) AND ('laparoscopy'/de OR 'laparoendoscopic single site surgery'/exp OR 'laparoscopic surgery'/exp OR 'minimally invasive procedure'/exp OR 'minimally invasive surgery'/exp OR laparoscop*:ti,ab,kw OR ((minimal* NEAR/3 invasive):ti,ab,kw) OR access:ti,ab,kw OR surg*:ti,ab,kw) OR 'laparoscopic sleeve gastrectomy'/exp OR lagb*:ti,ab,kw OR 'lap band*':ti,ab,kw OR lrygb*:ti,ab,kw | **40535** |

### T3Q5+Q6+Q7: Low molecular weight heparin (LMWH) (97 SRs, 27 RCTs, 107 cohort studies)

| **No.** | **Query** | **Results** |
| --- | --- | --- |
| **#12** | #9 AND #4 NOT (#10 OR #11) | **107** |
| **#11** | #9 AND #3 NOT #10 | **27** |
| **#10** | #2 AND #9 | **97** |
| **#9** | #8 NOT (('adolescent'/exp OR 'child'/exp OR adolescent*:ti,ab OR child*:ti,ab OR schoolchild*:ti,ab OR infant*:ti,ab OR girl*:ti,ab OR boy*:ti,ab OR teen:ti,ab OR teens:ti,ab OR teenager*:ti,ab OR youth*:ti,ab OR pediatr*:ti,ab OR paediatr*:ti,ab OR puber*:ti,ab) NOT ('adult'/exp OR 'aged'/exp OR 'middle aged'/exp OR adult*:ti,ab OR man:ti,ab OR men:ti,ab OR woman:ti,ab OR women:ti,ab)) | **284** |
| **#8** | #7 NOT ('conference abstract'/it OR 'conference paper'/it OR 'letter'/it OR 'note'/it) | **289** |
| **#7** | #6 NOT ([animals]/lim NOT [humans]/lim) AND [1-1-2004]/sd | **382** |
| **#6** | #1 AND #5 | **396** |
| **#5** | 'low molecular weight heparin'/exp OR lmwh* OR 'low molecular weight heparin*':ti,ab,kw OR tedelparin*:ti,ab,kw OR dalteparin*:ti,ab,kw OR fragmin*:ti,ab,kw OR enoxaparin*:ti,ab,kw OR lovenox:ti,ab,kw OR clexan*:ti,ab,kw OR nadroparin*:ti,ab,kw OR fraxiparin*:ti,ab,kw | **60317** |
| **#4** | 'epidemiology'/de OR (((cohort OR case) NEAR/3 (control OR controll* OR comparison OR referent)):ti,ab,kw) OR risk:ti,ab,kw OR causation:ti,ab,kw OR causal:ti,ab,kw OR 'odds ratio':ti,ab,kw OR etiol*:ti,ab,kw OR aetiol*:ti,ab,kw OR 'natural history':ti,ab,kw OR outcome:ti,ab,kw OR course:ti,ab,kw OR retrospect*:ti,ab,kw OR 'follow up':ti,ab,kw OR followup:ti,ab,kw OR predict*:ti,ab,kw OR prognos*:ti,ab,kw | **7313214** |
| **#3** | 'clinical study':ti,ab,kw AND 'trial':ti,ab,kw OR 'clinical trial'/exp OR random*:ti,ab,kw | **2191510** |
| **#2** | 'meta-analysis':ti,ab,kw OR 'meta analysis'/exp OR 'review'/exp OR review:ti,ab,kw | **3431289** |
| **#1** | ('obesity'/exp/mj AND 'surgery'/lnk OR 'bariatric surgery'/exp/mj OR 'roux y anastomosis'/exp/mj OR 'gastric bypass surgery'/exp/mj OR ((('bilio pancreatic' OR biliopancreatic) NEAR/3 (diversion* OR bypass*)):ti,kw) OR ((duodenal NEAR/3 switch*):ti,kw) OR (((stomach OR gastric) NEAR/3 (band* OR bypass* OR partiti* OR plicati* OR sleeve*)):ti,kw) OR ((sleeve NEAR/3 gastrectom*):ti,kw) OR ((roux NEAR/2 y):ti,kw) OR gastroplast*:ti,kw OR mgbp*:ti,kw OR oagb*:ti,kw OR rya:ti,kw OR rygb*:ti,kw OR sadis:ti,kw OR 'sadi s':ti,kw OR (((bariatric OR obes* OR 'weight loss' OR metabolic) NEAR/3 (surg* OR operati* OR procedure*)):ti,kw) OR sagb*:ti,kw) AND ('laparoscopy'/de OR 'laparoendoscopic single site surgery'/exp OR 'laparoscopic surgery'/exp OR 'minimally invasive procedure'/exp OR 'minimally invasive surgery'/exp OR laparoscop*:ti,ab,kw OR ((minimal* NEAR/3 invasive):ti,ab,kw) OR access:ti,ab,kw OR surg*:ti,ab,kw) OR 'laparoscopic sleeve gastrectomy'/exp OR lagb*:ti,ab,kw OR 'lap band*':ti,ab,kw OR lrygb*:ti,ab,kw | **40535** |

### T3Q8+Q9: Inferior vena cava filter (IVCF) (24 SRs, 5 RCTs, 18 cohort studies)

| **No.** | **Query** | **Results** |
| --- | --- | --- |
| **#12** | #9 AND #4 NOT (#10 OR #11) | **18** |
| **#11** | #9 AND #3 NOT #10 | **5** |
| **#10** | #2 AND #9 | **24** |
| **#9** | #8 NOT (('adolescent'/exp OR 'child'/exp OR adolescent*:ti,ab OR child*:ti,ab OR schoolchild*:ti,ab OR infant*:ti,ab OR girl*:ti,ab OR boy*:ti,ab OR teen:ti,ab OR teens:ti,ab OR teenager*:ti,ab OR youth*:ti,ab OR pediatr*:ti,ab OR paediatr*:ti,ab OR puber*:ti,ab) NOT ('adult'/exp OR 'aged'/exp OR 'middle aged'/exp OR adult*:ti,ab OR man:ti,ab OR men:ti,ab OR woman:ti,ab OR women:ti,ab)) | **55** |
| **#8** | #7 NOT ('conference abstract'/it OR 'conference paper'/it OR 'letter'/it OR 'note'/it) | **55** |
| **#7** | #6 NOT ([animals]/lim NOT [humans]/lim) AND [1-1-2004]/sd | **73** |
| **#6** | #1 AND #5 | **73** |
| **#5** | 'vena cava filter'/exp OR 'vena cava filter*':ti,ab,kw OR 'umbrella filter*':ti,ab,kw OR ivcf*:ti,ab,kw OR vcf*:ti,ab,kw | **7868** |
| **#4** | 'epidemiology'/de OR (((cohort OR case) NEAR/3 (control OR controll* OR comparison OR referent)):ti,ab,kw) OR risk:ti,ab,kw OR causation:ti,ab,kw OR causal:ti,ab,kw OR 'odds ratio':ti,ab,kw OR etiol*:ti,ab,kw OR aetiol*:ti,ab,kw OR 'natural history':ti,ab,kw OR outcome:ti,ab,kw OR course:ti,ab,kw OR retrospect*:ti,ab,kw OR 'follow up':ti,ab,kw OR followup:ti,ab,kw OR predict*:ti,ab,kw OR prognos*:ti,ab,kw | **7313214** |
| **#3** | 'clinical study':ti,ab,kw AND 'trial':ti,ab,kw OR 'clinical trial'/exp OR random*:ti,ab,kw | **2191510** |
| **#2** | 'meta-analysis':ti,ab,kw OR 'meta analysis'/exp OR 'review'/exp OR review:ti,ab,kw | **3431289** |
| **#1** | ('obesity'/exp/mj AND 'surgery'/lnk OR 'bariatric surgery'/exp/mj OR 'roux y anastomosis'/exp/mj OR 'gastric bypass surgery'/exp/mj OR ((('bilio pancreatic' OR biliopancreatic) NEAR/3 (diversion* OR bypass*)):ti,kw) OR ((duodenal NEAR/3 switch*):ti,kw) OR (((stomach OR gastric) NEAR/3 (band* OR bypass* OR partiti* OR plicati* OR sleeve*)):ti,kw) OR ((sleeve NEAR/3 gastrectom*):ti,kw) OR ((roux NEAR/2 y):ti,kw) OR gastroplast*:ti,kw OR mgbp*:ti,kw OR oagb*:ti,kw OR rya:ti,kw OR rygb*:ti,kw OR sadis:ti,kw OR 'sadi s':ti,kw OR (((bariatric OR obes* OR 'weight loss' OR metabolic) NEAR/3 (surg* OR operati* OR procedure*)):ti,kw) OR sagb*:ti,kw) AND ('laparoscopy'/de OR 'laparoendoscopic single site surgery'/exp OR 'laparoscopic surgery'/exp OR 'minimally invasive procedure'/exp OR 'minimally invasive surgery'/exp OR laparoscop*:ti,ab,kw OR ((minimal* NEAR/3 invasive):ti,ab,kw) OR access:ti,ab,kw OR surg*:ti,ab,kw) OR 'laparoscopic sleeve gastrectomy'/exp OR lagb*:ti,ab,kw OR 'lap band*':ti,ab,kw OR lrygb*:ti,ab,kw | **40535** |

### T3Q10: ERAS (15 SRs, 15 RCTs, 18 cohort studies) (11 February 2019)

| **No.** | **Query** | **Results** |
| --- | --- | --- |
| **#12** | #6 AND an AND #11 NOT (#10 OR #8) | **18** |
| **#11** | 'epidemiology'/de OR (((cohort OR case) NEAR/3 (control OR controll* OR comparison OR referent)):ti,ab,kw) OR risk:ti,ab,kw OR causation:ti,ab,kw OR causal:ti,ab,kw OR 'odds ratio':ti,ab,kw OR etiol*:ti,ab,kw OR aetiol*:ti,ab,kw OR 'natural history':ti,ab,kw OR outcome:ti,ab,kw OR course:ti,ab,kw OR retrospect*:ti,ab,kw OR 'follow up':ti,ab,kw OR followup:ti,ab,kw OR predict*:ti,ab,kw OR prognos*:ti,ab,kw | **7449307** |
| **#10** | #6 AND #9 NOT #8 | **15** |
| **#9** | 'clinical':ti,ab,kw AND 'trial':ti,ab,kw OR 'clinical trial'/exp OR random*:ti,ab,kw | **2325077** |
| **#8** | #6 AND #7 | **15** |
| **#7** | 'meta-analysis':ti,ab,kw OR 'meta analysis'/exp OR 'review'/exp OR review:ti,ab,kw | **3483264** |
| **#6** | #5 NOT (('adolescent'/exp OR 'child'/exp OR adolescent*:ti,ab OR child*:ti,ab OR schoolchild*:ti,ab OR infant*:ti,ab OR girl*:ti,ab OR boy*:ti,ab OR teen:ti,ab OR teens:ti,ab OR teenager*:ti,ab OR youth*:ti,ab OR pediatr*:ti,ab OR paediatr*:ti,ab OR puber*:ti,ab) NOT ('adult'/exp OR 'aged'/exp OR 'middle aged'/exp OR adult*:ti,ab OR man:ti,ab OR men:ti,ab OR woman:ti,ab OR women:ti,ab)) | **77** |
| **#5** | #4 NOT ('conference abstract'/it OR 'conference paper'/it OR 'letter'/it OR 'note'/it) | **77** |
| **#4** | #3 NOT ([animals]/lim NOT [humans]/lim) AND [1-1-2004]/sd | **178** |
| **#3** | #1 AND #2 | **179** |
| **#2** | 'enhanced recovery after surgery'/exp OR 'enhanced recovery after surgery':ti,ab,kw OR 'eras protocol*':ti,ab,kw OR 'eras program*':ti,ab,kw OR 'enhanced recovery protocol*':ti,ab,kw OR 'fast-track*':ti,ab,kw OR 'fasttrack*':ti,ab,kw OR 'early recovery protocol*':ti,ab,kw OR 'enhanced recovery program*':ti,ab,kw OR 'early recovery program*':ti,ab,kw | **8684** |
| **#1** | ('obesity'/exp/mj AND 'surgery'/lnk OR 'bariatric surgery'/exp/mj OR 'roux y anastomosis'/exp/mj OR 'gastric bypass surgery'/exp/mj OR ((('bilio pancreatic' OR biliopancreatic) NEAR/3 (diversion* OR bypass*)):ti,kw) OR ((duodenal NEAR/3 switch*):ti,kw) OR (((stomach OR gastric) NEAR/3 (band* OR bypass* OR partiti* OR plicati* OR sleeve*)):ti,kw) OR ((sleeve NEAR/3 gastrectom*):ti,kw) OR ((roux NEAR/2 y):ti,kw) OR gastroplast*:ti,kw OR mgbp*:ti,kw OR oagb*:ti,kw OR rya:ti,kw OR rygb*:ti,kw OR sadis:ti,kw OR 'sadi s':ti,kw OR (((bariatric OR obes* OR 'weight loss' OR metabolic) NEAR/3 (surg* OR operati* OR procedure*)):ti,kw) OR sagb*:ti,kw) AND ('laparoscopy'/de OR 'laparoendoscopic single site surgery'/exp OR 'laparoscopic surgery'/exp OR 'minimally invasive procedure'/exp OR 'minimally invasive surgery'/exp OR laparoscop*:ti,ab,kw OR ((minimal* NEAR/3 invasive):ti,ab,kw) OR access:ti,ab,kw OR surg*:ti,ab,kw) OR 'laparoscopic sleeve gastrectomy'/exp OR lagb*:ti,ab,kw OR 'lap band*':ti,ab,kw OR lrygb*:ti,ab,kw | **41336** |

# Topic 4: Primary bariatric surgery

## PubMed (21 November 2018)

### T4Qa: Gastric plication (21 SRs, 20 RCTs, 30 cohort studies)

| **Search** | **Query** | **Items found** |
| --- | --- | --- |
| [**#16**](https://www.ncbi.nlm.nih.gov/pubmed) | ((#10 AND #15) NOT (#12 OR #14)) | [**30**](https://www.ncbi.nlm.nih.gov/pubmed/?cmd=HistorySearch&querykey=16) |
| [**#15**](https://www.ncbi.nlm.nih.gov/pubmed) | "Epidemiologic Studies"[Mesh] OR "Prognosis"[Mesh] OR cohort[tiab] OR (case[tiab] AND (control[tiab] OR controll*[tiab] OR comparison[tiab] OR referent[tiab])) OR risk[tiab] OR causation[tiab] OR causal[tiab] OR "odds ratio"[tiab] OR etiol*[tiab] OR aetiol*[tiab] OR "natural history"[tiab] OR predict*[tiab] OR outcome[tiab] OR course[tiab] OR retrospect*[tiab] OR “follow up”[tiab] OR followup[tiab] OR prognos*[tiab] | [**6610007**](https://www.ncbi.nlm.nih.gov/pubmed/?cmd=HistorySearch&querykey=15) |
| [**#14**](https://www.ncbi.nlm.nih.gov/pubmed) | ((#10 AND #13) NOT #12) | [**20**](https://www.ncbi.nlm.nih.gov/pubmed/?cmd=HistorySearch&querykey=14) |
| [**#13**](https://www.ncbi.nlm.nih.gov/pubmed) | "Randomized Controlled Trial"[Publication Type] OR "Controlled Clinical Trial"[Publication Type] OR random*[tiab] OR placebo[tiab] OR "Drug Therapy"[Subheading] OR trial[tiab] OR groups[tiab] | [**4575298**](https://www.ncbi.nlm.nih.gov/pubmed/?cmd=HistorySearch&querykey=13) |
| [**#12**](https://www.ncbi.nlm.nih.gov/pubmed) | (#10 AND #11) | [**21**](https://www.ncbi.nlm.nih.gov/pubmed/?cmd=HistorySearch&querykey=12) |
| [**#11**](https://www.ncbi.nlm.nih.gov/pubmed) | (review[tiab] OR "Review"[Publication Type] OR "Meta-Analysis as Topic"[Mesh] OR meta-analysis[tiab] OR "Meta-Analysis"[Publication Type]) NOT ("Letter"[Publication Type] OR "Editorial"[Publication Type] OR "Comment"[Publication Type]) | [**2997369**](https://www.ncbi.nlm.nih.gov/pubmed/?cmd=HistorySearch&querykey=11) |
| [**#10**](https://www.ncbi.nlm.nih.gov/pubmed) | (#9 NOT (("Adolescent"[Mesh] OR "Child"[Mesh] OR "Infant"[Mesh] OR adolescen*[tiab] OR child*[tiab] OR schoolchild*[tiab] OR infant*[tiab] OR girl*[tiab] OR boy*[tiab] OR teen[tiab] OR teens[tiab] OR teenager*[tiab] OR youth*[tiab] OR pediatr*[tiab] OR paediatr*[tiab] OR puber*[tiab]) NOT ("Adult"[Mesh] OR adult*[tiab] OR man[tiab] OR men[tiab] OR woman[tiab] OR women[tiab]))) | [**81**](https://www.ncbi.nlm.nih.gov/pubmed/?cmd=HistorySearch&querykey=10) |
| [**#9**](https://www.ncbi.nlm.nih.gov/pubmed) | (#8 NOT ("Animals"[Mesh] NOT "Humans"[Mesh]) AND ("2004"[Date - Entrez] : "3000"[Date - Entrez])) | [**82**](https://www.ncbi.nlm.nih.gov/pubmed/?cmd=HistorySearch&querykey=9) |
| [**#8**](https://www.ncbi.nlm.nih.gov/pubmed) | (#4 AND #7) | [**84**](https://www.ncbi.nlm.nih.gov/pubmed/?cmd=HistorySearch&querykey=8) |
| [**#7**](https://www.ncbi.nlm.nih.gov/pubmed) | ((#5 AND #2) OR #6) | [**8446**](https://www.ncbi.nlm.nih.gov/pubmed/?cmd=HistorySearch&querykey=7) |
| [**#6**](https://www.ncbi.nlm.nih.gov/pubmed) | lagb*[tiab] OR lrygb*[tiab] OR lap band*[tiab] | [**2058**](https://www.ncbi.nlm.nih.gov/pubmed/?cmd=HistorySearch&querykey=6) |
| [**#5**](https://www.ncbi.nlm.nih.gov/pubmed) | "Anastomosis, Roux-en-Y"[Mesh] OR "Biliopancreatic Diversion"[Mesh] OR bilio pancreatic bypass*[tiab] OR bilio pancreatic diversion*[tiab] OR biliopancreatic bypass*[tiab] OR biliopancreatic diversion*[tiab] OR duodenal switch*[tiab] OR gastric band*[tiab] OR stomach band*[tiab] OR gastric bypass*[tiab] OR mgbp*[tiab] OR oagb*[tiab] OR roux en y*[tiab] OR “roux y”[tiab] OR “roux n y”[tiab] OR “roux in y”[tiab] OR rya[tiab] OR rygb*[tiab] OR sadis[tiab] OR “sadi s”[tiab] OR sagb*[tiab] OR sleeve gastrectom*[tiab] OR gastric sleeve*[tiab] OR ((biliopancreatic[ti] OR “bilio pancreatic”[ti]) AND (diversion*[ti] OR bypass*[ti])) OR (duodenal[ti] AND switch*[ti]) OR ((stomach[ti] OR gastric[ti]) AND (band*[ti] OR bypass*[ti] OR partiti*[ti] OR sleeve*[ti])) OR (sleeve[ti] AND gastrectom*[ti]) OR (roux[ti] AND y[ti]) | [**22025**](https://www.ncbi.nlm.nih.gov/pubmed/?cmd=HistorySearch&querykey=5) |
| [**#4**](https://www.ncbi.nlm.nih.gov/pubmed) | ((#1 AND #2) OR #3) | [**961**](https://www.ncbi.nlm.nih.gov/pubmed/?cmd=HistorySearch&querykey=4) |
| [**#3**](https://www.ncbi.nlm.nih.gov/pubmed) | lgp*[tiab] OR lgcp*[tiab] | [**882**](https://www.ncbi.nlm.nih.gov/pubmed/?cmd=HistorySearch&querykey=3) |
| [**#2**](https://www.ncbi.nlm.nih.gov/pubmed) | "Laparoscopy"[Mesh:NoExp] OR "Minimally Invasive Surgical Procedures"[Mesh:NoExp] OR laparoscop*[tiab] OR minimally invasive[tiab] OR minimal invasive[tiab] OR minimal access[tiab] OR minimal surg*[tiab] OR (minimal*[ti] AND invasive[ti]) OR (minimal[ti] AND (access[ti] OR surg*[ti])) | [**184006**](https://www.ncbi.nlm.nih.gov/pubmed/?cmd=HistorySearch&querykey=2) |
| [**#1**](https://www.ncbi.nlm.nih.gov/pubmed) | gastric plicati*[tiab] OR curvature plicati*[tiab] OR (gastric[ti] AND plicati*[ti]) | [**180**](https://www.ncbi.nlm.nih.gov/pubmed/?cmd=HistorySearch&querykey=1) |

### T4Qb: SADIS or DS (27 SRs, 25 RCTs, 56 cohort studies)

| **Search** | **Query** | **Items found** |
| --- | --- | --- |
| [**#33**](https://www.ncbi.nlm.nih.gov/pubmed) | ((#30 AND #15) NOT (#31 OR #32)) | [**56**](https://www.ncbi.nlm.nih.gov/pubmed/?cmd=HistorySearch&querykey=33) |
| [**#32**](https://www.ncbi.nlm.nih.gov/pubmed) | (#30 AND #13 NOT #31) | [**25**](https://www.ncbi.nlm.nih.gov/pubmed/?cmd=HistorySearch&querykey=32) |
| [**#31**](https://www.ncbi.nlm.nih.gov/pubmed) | (#30 AND #11) | [**27**](https://www.ncbi.nlm.nih.gov/pubmed/?cmd=HistorySearch&querykey=31) |
| [**#30**](https://www.ncbi.nlm.nih.gov/pubmed) | (#29 NOT (("Adolescent"[Mesh] OR "Child"[Mesh] OR "Infant"[Mesh] OR adolescen*[tiab] OR child*[tiab] OR schoolchild*[tiab] OR infant*[tiab] OR girl*[tiab] OR boy*[tiab] OR teen[tiab] OR teens[tiab] OR teenager*[tiab] OR youth*[tiab] OR pediatr*[tiab] OR paediatr*[tiab] OR puber*[tiab]) NOT ("Adult"[Mesh] OR adult*[tiab] OR man[tiab] OR men[tiab] OR woman[tiab] OR women[tiab]))) | [**147**](https://www.ncbi.nlm.nih.gov/pubmed/?cmd=HistorySearch&querykey=30) |
| [**#29**](https://www.ncbi.nlm.nih.gov/pubmed) | (#28 NOT ("Animals"[Mesh] NOT "Humans"[Mesh]) AND ("2004"[Date - Entrez] : "3000"[Date - Entrez])) | [**147**](https://www.ncbi.nlm.nih.gov/pubmed/?cmd=HistorySearch&querykey=29) |
| [**#28**](https://www.ncbi.nlm.nih.gov/pubmed) | (#27 AND #21) | [**156**](https://www.ncbi.nlm.nih.gov/pubmed/?cmd=HistorySearch&querykey=28) |
| [**#27**](https://www.ncbi.nlm.nih.gov/pubmed) | (#26 AND #2) | [**167**](https://www.ncbi.nlm.nih.gov/pubmed/?cmd=HistorySearch&querykey=27) |
| [**#26**](https://www.ncbi.nlm.nih.gov/pubmed) | (duodenal switch*[ti] OR (duodenal[ti] AND switch*[ti]) OR sadis[ti] OR “sadi s”[ti] OR “single anastomosis bypass”[ti] OR “single anastomosis bypasses”[ti] OR “single anastomosis duodenoileal switch”[ti] OR “single anastomosis duodeno ileal switch”[ti] OR duodenal switch*[ot] OR (duodenal[ti] AND switch*[ti]) OR sadis[ot] OR “sadi s”[ot] OR “single anastomosis bypass”[ot] OR “single anastomosis bypasses”[ot] OR “single anastomosis duodenoileal switch”[ot] OR “single anastomosis duodeno ileal switch”[ot]) | [**347**](https://www.ncbi.nlm.nih.gov/pubmed/?cmd=HistorySearch&querykey=26) |
| [**#21**](https://www.ncbi.nlm.nih.gov/pubmed) | ((#20 AND #2) OR #6) | [**8434**](https://www.ncbi.nlm.nih.gov/pubmed/?cmd=HistorySearch&querykey=21) |
| [**#20**](https://www.ncbi.nlm.nih.gov/pubmed) | ("Anastomosis, Roux-en-Y"[Mesh] OR "Biliopancreatic Diversion"[Mesh] OR bilio pancreatic bypass*[tiab] OR bilio pancreatic diversion*[tiab] OR biliopancreatic bypass*[tiab] OR biliopancreatic diversion*[tiab] OR gastric band*[tiab] OR stomach band*[tiab] OR gastric bypass*[tiab] OR mgbp*[tiab] OR oagb*[tiab] OR roux en y*[tiab] OR “roux y”[tiab] OR “roux n y”[tiab] OR “roux in y”[tiab] OR rya[tiab] OR rygb*[tiab] OR sagb*[tiab] OR sleeve gastrectom*[tiab] OR gastric sleeve*[tiab] OR ((biliopancreatic[ti] OR “bilio pancreatic”[ti]) AND (diversion*[ti] OR bypass*[ti])) OR ((stomach[ti] OR gastric[ti]) AND (band*[ti] OR bypass*[ti] OR partiti*[ti] OR sleeve*[ti])) OR (sleeve[ti] AND gastrectom*[ti]) OR (roux[ti] AND y[ti])) | [**21958**](https://www.ncbi.nlm.nih.gov/pubmed/?cmd=HistorySearch&querykey=20) |
| [**#15**](https://www.ncbi.nlm.nih.gov/pubmed) | "Epidemiologic Studies"[Mesh] OR "Prognosis"[Mesh] OR cohort[tiab] OR (case[tiab] AND (control[tiab] OR controll*[tiab] OR comparison[tiab] OR referent[tiab])) OR risk[tiab] OR causation[tiab] OR causal[tiab] OR "odds ratio"[tiab] OR etiol*[tiab] OR aetiol*[tiab] OR "natural history"[tiab] OR predict*[tiab] OR outcome[tiab] OR course[tiab] OR retrospect*[tiab] OR “follow up”[tiab] OR followup[tiab] OR prognos*[tiab] | [**6610007**](https://www.ncbi.nlm.nih.gov/pubmed/?cmd=HistorySearch&querykey=15) |
| [**#13**](https://www.ncbi.nlm.nih.gov/pubmed) | "Randomized Controlled Trial"[Publication Type] OR "Controlled Clinical Trial"[Publication Type] OR random*[tiab] OR placebo[tiab] OR "Drug Therapy"[Subheading] OR trial[tiab] OR groups[tiab] | [**4575298**](https://www.ncbi.nlm.nih.gov/pubmed/?cmd=HistorySearch&querykey=13) |
| [**#11**](https://www.ncbi.nlm.nih.gov/pubmed) | (review[tiab] OR "Review"[Publication Type] OR "Meta-Analysis as Topic"[Mesh] OR meta-analysis[tiab] OR "Meta-Analysis"[Publication Type]) NOT ("Letter"[Publication Type] OR "Editorial"[Publication Type] OR "Comment"[Publication Type]) | [**2997369**](https://www.ncbi.nlm.nih.gov/pubmed/?cmd=HistorySearch&querykey=11) |
| [**#3**](https://www.ncbi.nlm.nih.gov/pubmed) | lgp*[tiab] OR lgcp*[tiab] | [**882**](https://www.ncbi.nlm.nih.gov/pubmed/?cmd=HistorySearch&querykey=3) |
| [**#2**](https://www.ncbi.nlm.nih.gov/pubmed) | "Laparoscopy"[Mesh:NoExp] OR "Minimally Invasive Surgical Procedures"[Mesh:NoExp] OR laparoscop*[tiab] OR minimally invasive[tiab] OR minimal invasive[tiab] OR minimal access[tiab] OR minimal surg*[tiab] OR (minimal*[ti] AND invasive[ti]) OR (minimal[ti] AND (access[ti] OR surg*[ti])) | [**184006**](https://www.ncbi.nlm.nih.gov/pubmed/?cmd=HistorySearch&querykey=2) |

### T4Qc: BPD (43 SRs, 27 RCTs, 58 cohort studies)

| **Search** | **Query** | **Items found** |
| --- | --- | --- |
| [**#47**](https://www.ncbi.nlm.nih.gov/pubmed) | ((#44 AND #15) NOT (#45 OR #46)) | [**58**](https://www.ncbi.nlm.nih.gov/pubmed/?cmd=HistorySearch&querykey=47) |
| [**#46**](https://www.ncbi.nlm.nih.gov/pubmed) | (#44 AND #13 NOT #45) | [**27**](https://www.ncbi.nlm.nih.gov/pubmed/?cmd=HistorySearch&querykey=46) |
| [**#45**](https://www.ncbi.nlm.nih.gov/pubmed) | (#44 AND #11) | [**43**](https://www.ncbi.nlm.nih.gov/pubmed/?cmd=HistorySearch&querykey=45) |
| [**#44**](https://www.ncbi.nlm.nih.gov/pubmed) | (#43 NOT (("Adolescent"[Mesh] OR "Child"[Mesh] OR "Infant"[Mesh] OR adolescen*[tiab] OR child*[tiab] OR schoolchild*[tiab] OR infant*[tiab] OR girl*[tiab] OR boy*[tiab] OR teen[tiab] OR teens[tiab] OR teenager*[tiab] OR youth*[tiab] OR pediatr*[tiab] OR paediatr*[tiab] OR puber*[tiab]) NOT ("Adult"[Mesh] OR adult*[tiab] OR man[tiab] OR men[tiab] OR woman[tiab] OR women[tiab]))) | [**157**](https://www.ncbi.nlm.nih.gov/pubmed/?cmd=HistorySearch&querykey=44) |
| [**#43**](https://www.ncbi.nlm.nih.gov/pubmed) | (#42 NOT ("Animals"[Mesh] NOT "Humans"[Mesh]) AND ("2004"[Date - Entrez] : "3000"[Date - Entrez])) | [**160**](https://www.ncbi.nlm.nih.gov/pubmed/?cmd=HistorySearch&querykey=43) |
| [**#42**](https://www.ncbi.nlm.nih.gov/pubmed) | (#41 AND #37) | [**181**](https://www.ncbi.nlm.nih.gov/pubmed/?cmd=HistorySearch&querykey=42) |
| [**#41**](https://www.ncbi.nlm.nih.gov/pubmed) | (#40 AND #2) | [**280**](https://www.ncbi.nlm.nih.gov/pubmed/?cmd=HistorySearch&querykey=41) |
| [**#40**](https://www.ncbi.nlm.nih.gov/pubmed) | ("Biliopancreatic Diversion"[Mesh] OR ((biliopancreatic[ti] OR “bilio pancreatic”[ti]) AND (diversion*[ti] OR bypass*[ti])) OR scopinaro*[ti] OR bilio pancreatic bypass*[ot] OR bilio pancreatic diversion*[ot] OR biliopancreatic bypass*[ot] OR biliopancreatic diversion*[ot]) | [**1097**](https://www.ncbi.nlm.nih.gov/pubmed/?cmd=HistorySearch&querykey=40) |
| [**#37**](https://www.ncbi.nlm.nih.gov/pubmed) | ((#36 AND #2) OR #6) | [**8332**](https://www.ncbi.nlm.nih.gov/pubmed/?cmd=HistorySearch&querykey=37) |
| [**#36**](https://www.ncbi.nlm.nih.gov/pubmed) | ("Anastomosis, Roux-en-Y"[Mesh] OR gastric band*[tiab] OR stomach band*[tiab] OR gastric bypass*[tiab] OR mgbp*[tiab] OR oagb*[tiab] OR roux en y*[tiab] OR “roux y”[tiab] OR “roux n y”[tiab] OR “roux in y”[tiab] OR rya[tiab] OR rygb*[tiab] OR sagb*[tiab] OR sleeve gastrectom*[tiab] OR gastric sleeve*[tiab] OR ((stomach[ti] OR gastric[ti]) AND (band*[ti] OR bypass*[ti] OR partiti*[ti] OR sleeve*[ti])) OR (sleeve[ti] AND gastrectom*[ti]) OR (roux[ti] AND y[ti])) | [**21193**](https://www.ncbi.nlm.nih.gov/pubmed/?cmd=HistorySearch&querykey=36) |
| [**#15**](https://www.ncbi.nlm.nih.gov/pubmed) | "Epidemiologic Studies"[Mesh] OR "Prognosis"[Mesh] OR cohort[tiab] OR (case[tiab] AND (control[tiab] OR controll*[tiab] OR comparison[tiab] OR referent[tiab])) OR risk[tiab] OR causation[tiab] OR causal[tiab] OR "odds ratio"[tiab] OR etiol*[tiab] OR aetiol*[tiab] OR "natural history"[tiab] OR predict*[tiab] OR outcome[tiab] OR course[tiab] OR retrospect*[tiab] OR “follow up”[tiab] OR followup[tiab] OR prognos*[tiab] | [**6610007**](https://www.ncbi.nlm.nih.gov/pubmed/?cmd=HistorySearch&querykey=15) |
| [**#13**](https://www.ncbi.nlm.nih.gov/pubmed) | "Randomized Controlled Trial"[Publication Type] OR "Controlled Clinical Trial"[Publication Type] OR random*[tiab] OR placebo[tiab] OR "Drug Therapy"[Subheading] OR trial[tiab] OR groups[tiab] | [**4575298**](https://www.ncbi.nlm.nih.gov/pubmed/?cmd=HistorySearch&querykey=13) |
| [**#11**](https://www.ncbi.nlm.nih.gov/pubmed) | (review[tiab] OR "Review"[Publication Type] OR "Meta-Analysis as Topic"[Mesh] OR meta-analysis[tiab] OR "Meta-Analysis"[Publication Type]) NOT ("Letter"[Publication Type] OR "Editorial"[Publication Type] OR "Comment"[Publication Type]) | [**2997369**](https://www.ncbi.nlm.nih.gov/pubmed/?cmd=HistorySearch&querykey=11) |
| [**#6**](https://www.ncbi.nlm.nih.gov/pubmed) | lagb*[tiab] OR lrygb*[tiab] OR lap band*[tiab] OR lrygb*[tiab] | [**2058**](https://www.ncbi.nlm.nih.gov/pubmed/?cmd=HistorySearch&querykey=6) |
| [**#2**](https://www.ncbi.nlm.nih.gov/pubmed) | "Laparoscopy"[Mesh:NoExp] OR "Minimally Invasive Surgical Procedures"[Mesh:NoExp] OR laparoscop*[tiab] OR minimally invasive[tiab] OR minimal invasive[tiab] OR minimal access[tiab] OR minimal surg*[tiab] OR (minimal*[ti] AND invasive[ti]) OR (minimal[ti] AND (access[ti] OR surg*[ti])) | [**184006**](https://www.ncbi.nlm.nih.gov/pubmed/?cmd=HistorySearch&querykey=2) |

### T4Qd: MGB (37 SRs, 32 RCTs, 93 cohort studies)

| **Search** | **Query** | **Items found** |
| --- | --- | --- |
| [**#55**](https://www.ncbi.nlm.nih.gov/pubmed) | ((#52 AND #15) NOT (#53 OR #54)) | [**93**](https://www.ncbi.nlm.nih.gov/pubmed/?cmd=HistorySearch&querykey=55) |
| [**#54**](https://www.ncbi.nlm.nih.gov/pubmed) | (#52 AND #13 NOT #53) | [**32**](https://www.ncbi.nlm.nih.gov/pubmed/?cmd=HistorySearch&querykey=54) |
| [**#53**](https://www.ncbi.nlm.nih.gov/pubmed) | (#52 AND #11) | [**37**](https://www.ncbi.nlm.nih.gov/pubmed/?cmd=HistorySearch&querykey=53) |
| [**#52**](https://www.ncbi.nlm.nih.gov/pubmed) | (#51 NOT (("Adolescent"[Mesh] OR "Child"[Mesh] OR "Infant"[Mesh] OR adolescen*[tiab] OR child*[tiab] OR schoolchild*[tiab] OR infant*[tiab] OR girl*[tiab] OR boy*[tiab] OR teen[tiab] OR teens[tiab] OR teenager*[tiab] OR youth*[tiab] OR pediatr*[tiab] OR paediatr*[tiab] OR puber*[tiab]) NOT ("Adult"[Mesh] OR adult*[tiab] OR man[tiab] OR men[tiab] OR woman[tiab] OR women[tiab]))) | [**205**](https://www.ncbi.nlm.nih.gov/pubmed/?cmd=HistorySearch&querykey=52) |
| [**#51**](https://www.ncbi.nlm.nih.gov/pubmed) | (#50 NOT ("Animals"[Mesh] NOT "Humans"[Mesh]) AND ("2004"[Date - Entrez] : "3000"[Date - Entrez])) | [**207**](https://www.ncbi.nlm.nih.gov/pubmed/?cmd=HistorySearch&querykey=51) |
| [**#50**](https://www.ncbi.nlm.nih.gov/pubmed) | (#49 AND #37) | [**208**](https://www.ncbi.nlm.nih.gov/pubmed/?cmd=HistorySearch&querykey=50) |
| [**#49**](https://www.ncbi.nlm.nih.gov/pubmed) | (#48 AND #2) | [**215**](https://www.ncbi.nlm.nih.gov/pubmed/?cmd=HistorySearch&querykey=49) |
| [**#48**](https://www.ncbi.nlm.nih.gov/pubmed) | (mini gastric bypass*[tiab] OR mgb*[tiab] OR “omega gastric bypass”[tiab] OR single anastomosis*[tiab] OR single anastomosis gastric bypass*[tiab] OR one anastomosis gastric bypass*[tiab] OR mgbp*[tiab]) | [**2352**](https://www.ncbi.nlm.nih.gov/pubmed/?cmd=HistorySearch&querykey=48) |
| [**#37**](https://www.ncbi.nlm.nih.gov/pubmed) | ((#36 AND #2) OR #6) | [**8332**](https://www.ncbi.nlm.nih.gov/pubmed/?cmd=HistorySearch&querykey=37) |
| [**#15**](https://www.ncbi.nlm.nih.gov/pubmed) | "Epidemiologic Studies"[Mesh] OR "Prognosis"[Mesh] OR cohort[tiab] OR (case[tiab] AND (control[tiab] OR controll*[tiab] OR comparison[tiab] OR referent[tiab])) OR risk[tiab] OR causation[tiab] OR causal[tiab] OR "odds ratio"[tiab] OR etiol*[tiab] OR aetiol*[tiab] OR "natural history"[tiab] OR predict*[tiab] OR outcome[tiab] OR course[tiab] OR retrospect*[tiab] OR “follow up”[tiab] OR followup[tiab] OR prognos*[tiab] | [**6610007**](https://www.ncbi.nlm.nih.gov/pubmed/?cmd=HistorySearch&querykey=15) |
| [**#13**](https://www.ncbi.nlm.nih.gov/pubmed) | "Randomized Controlled Trial"[Publication Type] OR "Controlled Clinical Trial"[Publication Type] OR random*[tiab] OR placebo[tiab] OR "Drug Therapy"[Subheading] OR trial[tiab] OR groups[tiab] | [**4575298**](https://www.ncbi.nlm.nih.gov/pubmed/?cmd=HistorySearch&querykey=13) |
| [**#11**](https://www.ncbi.nlm.nih.gov/pubmed) | (review[tiab] OR "Review"[Publication Type] OR "Meta-Analysis as Topic"[Mesh] OR meta-analysis[tiab] OR "Meta-Analysis"[Publication Type]) NOT ("Letter"[Publication Type] OR "Editorial"[Publication Type] OR "Comment"[Publication Type]) | [**2997369**](https://www.ncbi.nlm.nih.gov/pubmed/?cmd=HistorySearch&querykey=11) |
| [**#6**](https://www.ncbi.nlm.nih.gov/pubmed) | lagb*[tiab] OR lrygb*[tiab] OR lap band*[tiab] OR lrygb*[tiab] | [**2058**](https://www.ncbi.nlm.nih.gov/pubmed/?cmd=HistorySearch&querykey=6) |
| [**#2**](https://www.ncbi.nlm.nih.gov/pubmed) | "Laparoscopy"[Mesh:NoExp] OR "Minimally Invasive Surgical Procedures"[Mesh:NoExp] OR laparoscop*[tiab] OR minimally invasive[tiab] OR minimal invasive[tiab] OR minimal access[tiab] OR minimal surg*[tiab] OR (minimal*[ti] AND invasive[ti]) OR (minimal[ti] AND (access[ti] OR surg*[ti])) | [**184006**](https://www.ncbi.nlm.nih.gov/pubmed/?cmd=HistorySearch&querykey=2) |

### T4Qe: Banding (133 SRs, 152 RCTs, 246 cohort studies)

| **Search** | **Query** | **Items found** |
| --- | --- | --- |
| [**#79**](https://www.ncbi.nlm.nih.gov/pubmed) | ((#76 AND #15) NOT (#77 OR #78)) | [**246**](https://www.ncbi.nlm.nih.gov/pubmed/?cmd=HistorySearch&querykey=79) |
| [**#78**](https://www.ncbi.nlm.nih.gov/pubmed) | (#76 AND #13 NOT #77) | [**152**](https://www.ncbi.nlm.nih.gov/pubmed/?cmd=HistorySearch&querykey=78) |
| [**#77**](https://www.ncbi.nlm.nih.gov/pubmed) | (#76 AND #11) | [**133**](https://www.ncbi.nlm.nih.gov/pubmed/?cmd=HistorySearch&querykey=77) |
| [**#76**](https://www.ncbi.nlm.nih.gov/pubmed) | (#75 NOT (("Adolescent"[Mesh] OR "Child"[Mesh] OR "Infant"[Mesh] OR adolescen*[tiab] OR child*[tiab] OR schoolchild*[tiab] OR infant*[tiab] OR girl*[tiab] OR boy*[tiab] OR teen[tiab] OR teens[tiab] OR teenager*[tiab] OR youth*[tiab] OR pediatr*[tiab] OR paediatr*[tiab] OR puber*[tiab]) NOT ("Adult"[Mesh] OR adult*[tiab] OR man[tiab] OR men[tiab] OR woman[tiab] OR women[tiab]))) | [**592**](https://www.ncbi.nlm.nih.gov/pubmed/?cmd=HistorySearch&querykey=76) |
| [**#75**](https://www.ncbi.nlm.nih.gov/pubmed) | (#74 NOT ("Animals"[Mesh] NOT "Humans"[Mesh]) AND ("2004"[Date - Entrez] : "3000"[Date - Entrez])) | [**604**](https://www.ncbi.nlm.nih.gov/pubmed/?cmd=HistorySearch&querykey=75) |
| [**#74**](https://www.ncbi.nlm.nih.gov/pubmed) | (#73 NOT (revision*[ti] OR revising*[ti] OR redo[ti])) | [**641**](https://www.ncbi.nlm.nih.gov/pubmed/?cmd=HistorySearch&querykey=74) |
| [**#73**](https://www.ncbi.nlm.nih.gov/pubmed) | (#68 AND #72) | [**714**](https://www.ncbi.nlm.nih.gov/pubmed/?cmd=HistorySearch&querykey=73) |
| [**#72**](https://www.ncbi.nlm.nih.gov/pubmed) | ((#71 AND #2) OR #64) | [**6661**](https://www.ncbi.nlm.nih.gov/pubmed/?cmd=HistorySearch&querykey=72) |
| [**#71**](https://www.ncbi.nlm.nih.gov/pubmed) | ("Anastomosis, Roux-en-Y"[Mesh] OR gastric bypass*[tiab] OR mgbp*[tiab] OR oagb*[tiab] OR roux en y*[tiab] OR “roux y”[tiab] OR “roux n y”[tiab] OR “roux in y”[tiab] OR rya[tiab] OR rygb*[tiab] OR sleeve gastrectom*[tiab] OR gastric sleeve*[tiab] OR ((stomach[ti] OR gastric[ti]) AND (bypass*[ti] OR partiti*[ti] OR sleeve*[ti])) OR (sleeve[ti] AND gastrectom*[ti]) OR (roux[ti] AND y[ti])) | [**17504**](https://www.ncbi.nlm.nih.gov/pubmed/?cmd=HistorySearch&querykey=71) |
| [**#68**](https://www.ncbi.nlm.nih.gov/pubmed) | ((#67 AND #2) OR #61) | [**2168**](https://www.ncbi.nlm.nih.gov/pubmed/?cmd=HistorySearch&querykey=68) |
| [**#67**](https://www.ncbi.nlm.nih.gov/pubmed) | (sagb*[ti] OR sagb*[ot] OR ((stomach[ti] OR gastric[ti] OR esophagogastric[ti] OR oesophagogastric[ti]) AND band*[ti]) OR ((stomach[ot] OR gastric[ot] OR esophagogastric[ot] OR oesophagogastric[ot]) AND band*[ot])) | [**2579**](https://www.ncbi.nlm.nih.gov/pubmed/?cmd=HistorySearch&querykey=67) |
| [**#64**](https://www.ncbi.nlm.nih.gov/pubmed) | (lrygb*[tiab] OR lrygb*[tiab]) | [**897**](https://www.ncbi.nlm.nih.gov/pubmed/?cmd=HistorySearch&querykey=64) |
| [**#61**](https://www.ncbi.nlm.nih.gov/pubmed) | (lagb*[tiab] OR lap band*[tiab]) | [**1287**](https://www.ncbi.nlm.nih.gov/pubmed/?cmd=HistorySearch&querykey=61) |
| [**#15**](https://www.ncbi.nlm.nih.gov/pubmed) | "Epidemiologic Studies"[Mesh] OR "Prognosis"[Mesh] OR cohort[tiab] OR (case[tiab] AND (control[tiab] OR controll*[tiab] OR comparison[tiab] OR referent[tiab])) OR risk[tiab] OR causation[tiab] OR causal[tiab] OR "odds ratio"[tiab] OR etiol*[tiab] OR aetiol*[tiab] OR "natural history"[tiab] OR predict*[tiab] OR outcome[tiab] OR course[tiab] OR retrospect*[tiab] OR “follow up”[tiab] OR followup[tiab] OR prognos*[tiab] | [**6610007**](https://www.ncbi.nlm.nih.gov/pubmed/?cmd=HistorySearch&querykey=15) |
| [**#13**](https://www.ncbi.nlm.nih.gov/pubmed) | "Randomized Controlled Trial"[Publication Type] OR "Controlled Clinical Trial"[Publication Type] OR random*[tiab] OR placebo[tiab] OR "Drug Therapy"[Subheading] OR trial[tiab] OR groups[tiab] | [**4575298**](https://www.ncbi.nlm.nih.gov/pubmed/?cmd=HistorySearch&querykey=13) |
| [**#11**](https://www.ncbi.nlm.nih.gov/pubmed) | (review[tiab] OR "Review"[Publication Type] OR "Meta-Analysis as Topic"[Mesh] OR meta-analysis[tiab] OR "Meta-Analysis"[Publication Type]) NOT ("Letter"[Publication Type] OR "Editorial"[Publication Type] OR "Comment"[Publication Type]) | [**2997369**](https://www.ncbi.nlm.nih.gov/pubmed/?cmd=HistorySearch&querykey=11) |
| [**#2**](https://www.ncbi.nlm.nih.gov/pubmed) | "Laparoscopy"[Mesh:NoExp] OR "Minimally Invasive Surgical Procedures"[Mesh:NoExp] OR laparoscop*[tiab] OR minimally invasive[tiab] OR minimal invasive[tiab] OR minimal access[tiab] OR minimal surg*[tiab] OR (minimal*[ti] AND invasive[ti]) OR (minimal[ti] AND (access[ti] OR surg*[ti])) | [**184006**](https://www.ncbi.nlm.nih.gov/pubmed/?cmd=HistorySearch&querykey=2) |

### T4Qf: Sleeve (150 SRs, 162 RCTs, 284 cohort studies)

| **Search** | **Query** | **Items found** |
| --- | --- | --- |
| [**#93**](https://www.ncbi.nlm.nih.gov/pubmed) | ((#90 AND #15) NOT (#91 OR #92)) | [**284**](https://www.ncbi.nlm.nih.gov/pubmed/?cmd=HistorySearch&querykey=93) |
| [**#92**](https://www.ncbi.nlm.nih.gov/pubmed) | (#90 AND #13 NOT #91) | [**162**](https://www.ncbi.nlm.nih.gov/pubmed/?cmd=HistorySearch&querykey=92) |
| [**#91**](https://www.ncbi.nlm.nih.gov/pubmed) | (#90 AND #11) | [**150**](https://www.ncbi.nlm.nih.gov/pubmed/?cmd=HistorySearch&querykey=91) |
| [**#90**](https://www.ncbi.nlm.nih.gov/pubmed) | (#89 NOT (("Adolescent"[Mesh] OR "Child"[Mesh] OR "Infant"[Mesh] OR adolescen*[tiab] OR child*[tiab] OR schoolchild*[tiab] OR infant*[tiab] OR girl*[tiab] OR boy*[tiab] OR teen[tiab] OR teens[tiab] OR teenager*[tiab] OR youth*[tiab] OR pediatr*[tiab] OR paediatr*[tiab] OR puber*[tiab]) NOT ("Adult"[Mesh] OR adult*[tiab] OR man[tiab] OR men[tiab] OR woman[tiab] OR women[tiab]))) | [**662**](https://www.ncbi.nlm.nih.gov/pubmed/?cmd=HistorySearch&querykey=90) |
| [**#89**](https://www.ncbi.nlm.nih.gov/pubmed) | (#88 NOT ("Animals"[Mesh] NOT "Humans"[Mesh]) AND ("2004"[Date - Entrez] : "3000"[Date - Entrez])) | [**669**](https://www.ncbi.nlm.nih.gov/pubmed/?cmd=HistorySearch&querykey=89) |
| [**#88**](https://www.ncbi.nlm.nih.gov/pubmed) | (#87 NOT (revision*[ti] OR revising*[ti] OR redo[ti])) | [**678**](https://www.ncbi.nlm.nih.gov/pubmed/?cmd=HistorySearch&querykey=88) |
| [**#87**](https://www.ncbi.nlm.nih.gov/pubmed) | (#86 AND #84) | [**723**](https://www.ncbi.nlm.nih.gov/pubmed/?cmd=HistorySearch&querykey=87) |
| [**#86**](https://www.ncbi.nlm.nih.gov/pubmed) | (#85 AND #2) | [**2330**](https://www.ncbi.nlm.nih.gov/pubmed/?cmd=HistorySearch&querykey=86) |
| [**#85**](https://www.ncbi.nlm.nih.gov/pubmed) | (((stomach[ti] OR gastric[ti] OR gastrectom*[ti]) AND sleeve*[ti]) OR (vertical[ti] AND gastroplast*[ti]) OR ((stomach[ot] OR gastric[ot] OR gastrectom*[ot]) AND sleeve*[ot]) OR (vertical[ot] AND gastroplast*[ot])) | [**3570**](https://www.ncbi.nlm.nih.gov/pubmed/?cmd=HistorySearch&querykey=85) |
| [**#84**](https://www.ncbi.nlm.nih.gov/pubmed) | ((#82 AND #2) OR #83) | [**4951**](https://www.ncbi.nlm.nih.gov/pubmed/?cmd=HistorySearch&querykey=84) |
| [**#83**](https://www.ncbi.nlm.nih.gov/pubmed) | lrygb*[tiab] | [**897**](https://www.ncbi.nlm.nih.gov/pubmed/?cmd=HistorySearch&querykey=83) |
| [**#82**](https://www.ncbi.nlm.nih.gov/pubmed) | ("Anastomosis, Roux-en-Y"[Mesh] OR gastric bypass*[tiab] OR roux en y*[tiab] OR “roux y”[tiab] OR “roux n y”[tiab] OR “roux in y”[tiab] OR rya[tiab] OR rygb*[tiab] OR ((stomach[ti] OR gastric[ti]) AND bypass*[ti]) OR (roux[ti] AND y[ti])) | [**15004**](https://www.ncbi.nlm.nih.gov/pubmed/?cmd=HistorySearch&querykey=82) |
| [**#15**](https://www.ncbi.nlm.nih.gov/pubmed) | "Epidemiologic Studies"[Mesh] OR "Prognosis"[Mesh] OR cohort[tiab] OR (case[tiab] AND (control[tiab] OR controll*[tiab] OR comparison[tiab] OR referent[tiab])) OR risk[tiab] OR causation[tiab] OR causal[tiab] OR "odds ratio"[tiab] OR etiol*[tiab] OR aetiol*[tiab] OR "natural history"[tiab] OR predict*[tiab] OR outcome[tiab] OR course[tiab] OR retrospect*[tiab] OR “follow up”[tiab] OR followup[tiab] OR prognos*[tiab] | [**6610007**](https://www.ncbi.nlm.nih.gov/pubmed/?cmd=HistorySearch&querykey=15) |
| [**#13**](https://www.ncbi.nlm.nih.gov/pubmed) | "Randomized Controlled Trial"[Publication Type] OR "Controlled Clinical Trial"[Publication Type] OR random*[tiab] OR placebo[tiab] OR "Drug Therapy"[Subheading] OR trial[tiab] OR groups[tiab] | [**4575298**](https://www.ncbi.nlm.nih.gov/pubmed/?cmd=HistorySearch&querykey=13) |
| [**#11**](https://www.ncbi.nlm.nih.gov/pubmed) | (review[tiab] OR "Review"[Publication Type] OR "Meta-Analysis as Topic"[Mesh] OR meta-analysis[tiab] OR "Meta-Analysis"[Publication Type]) NOT ("Letter"[Publication Type] OR "Editorial"[Publication Type] OR "Comment"[Publication Type]) | [**2997369**](https://www.ncbi.nlm.nih.gov/pubmed/?cmd=HistorySearch&querykey=11) |
| [**#10**](https://www.ncbi.nlm.nih.gov/pubmed) | (#9 NOT (("Adolescent"[Mesh] OR "Child"[Mesh] OR "Infant"[Mesh] OR adolescen*[tiab] OR child*[tiab] OR schoolchild*[tiab] OR infant*[tiab] OR girl*[tiab] OR boy*[tiab] OR teen[tiab] OR teens[tiab] OR teenager*[tiab] OR youth*[tiab] OR pediatr*[tiab] OR paediatr*[tiab] OR puber*[tiab]) NOT ("Adult"[Mesh] OR adult*[tiab] OR man[tiab] OR men[tiab] OR woman[tiab] OR women[tiab]))) | [**81**](https://www.ncbi.nlm.nih.gov/pubmed/?cmd=HistorySearch&querykey=10) |
| [**#2**](https://www.ncbi.nlm.nih.gov/pubmed) | "Laparoscopy"[Mesh:NoExp] OR "Minimally Invasive Surgical Procedures"[Mesh:NoExp] OR laparoscop*[tiab] OR minimally invasive[tiab] OR minimal invasive[tiab] OR minimal access[tiab] OR minimal surg*[tiab] OR (minimal*[ti] AND invasive[ti]) OR (minimal[ti] AND (access[ti] OR surg*[ti])) | [**184006**](https://www.ncbi.nlm.nih.gov/pubmed/?cmd=HistorySearch&querykey=2) |

### T4Q14: Sleeve + resection, stapling, bougie (43 SRs, 58 RCTs, 86 cohort studies)

| **Search** | **Query** | **Items found** |
| --- | --- | --- |
| [**#118**](https://www.ncbi.nlm.nih.gov/pubmed) | ((#112 AND #117) NOT (#114 OR #116)) | [**86**](https://www.ncbi.nlm.nih.gov/pubmed/?cmd=HistorySearch&querykey=118) |
| [**#117**](https://www.ncbi.nlm.nih.gov/pubmed) | ("Epidemiologic Studies"[Mesh] OR "Prognosis"[Mesh] OR cohort[tiab] OR (case[tiab] AND (control[tiab] OR controll*[tiab] OR comparison[tiab] OR referent[tiab])) OR risk[tiab] OR causation[tiab] OR causal[tiab] OR "odds ratio"[tiab] OR etiol*[tiab] OR aetiol*[tiab] OR "natural history"[tiab] OR predict*[tiab] OR outcome[tiab] OR course[tiab] OR retrospect*[tiab] OR “follow up”[tiab] OR followup[tiab] OR prognos*[tiab]) | [**6610007**](https://www.ncbi.nlm.nih.gov/pubmed/?cmd=HistorySearch&querykey=117) |
| [**#116**](https://www.ncbi.nlm.nih.gov/pubmed) | (#112 AND #115 NOT #114) | [**58**](https://www.ncbi.nlm.nih.gov/pubmed/?cmd=HistorySearch&querykey=116) |
| [**#115**](https://www.ncbi.nlm.nih.gov/pubmed) | ("Randomized Controlled Trial"[Publication Type] OR "Controlled Clinical Trial"[Publication Type] OR random*[tiab] OR placebo[tiab] OR "Drug Therapy"[Subheading] OR trial[tiab] OR groups[tiab]) | [**4575298**](https://www.ncbi.nlm.nih.gov/pubmed/?cmd=HistorySearch&querykey=115) |
| [**#114**](https://www.ncbi.nlm.nih.gov/pubmed) | (#112 AND #113) | [**43**](https://www.ncbi.nlm.nih.gov/pubmed/?cmd=HistorySearch&querykey=114) |
| [**#113**](https://www.ncbi.nlm.nih.gov/pubmed) | ((review[tiab] OR "Review"[Publication Type] OR "Meta-Analysis as Topic"[Mesh] OR meta-analysis[tiab] OR "Meta-Analysis"[Publication Type]) NOT ("Letter"[Publication Type] OR "Editorial"[Publication Type] OR "Comment"[Publication Type])) | [**2997369**](https://www.ncbi.nlm.nih.gov/pubmed/?cmd=HistorySearch&querykey=113) |
| [**#112**](https://www.ncbi.nlm.nih.gov/pubmed) | (#111 NOT (("Adolescent"[Mesh] OR "Child"[Mesh] OR "Infant"[Mesh] OR adolescen*[tiab] OR child*[tiab] OR schoolchild*[tiab] OR infant*[tiab] OR girl*[tiab] OR boy*[tiab] OR teen[tiab] OR teens[tiab] OR teenager*[tiab] OR youth*[tiab] OR pediatr*[tiab] OR paediatr*[tiab] OR puber*[tiab]) NOT ("Adult"[Mesh] OR adult*[tiab] OR man[tiab] OR men[tiab] OR woman[tiab] OR women[tiab]))) | [**215**](https://www.ncbi.nlm.nih.gov/pubmed/?cmd=HistorySearch&querykey=112) |
| [**#111**](https://www.ncbi.nlm.nih.gov/pubmed) | (#110 NOT ("Animals"[Mesh] NOT "Humans"[Mesh]) AND ("2004"[Date - Entrez] : "3000"[Date - Entrez])) | [**215**](https://www.ncbi.nlm.nih.gov/pubmed/?cmd=HistorySearch&querykey=111) |
| [**#110**](https://www.ncbi.nlm.nih.gov/pubmed) | (#104 AND (#105 OR #107 OR #109)) | [**236**](https://www.ncbi.nlm.nih.gov/pubmed/?cmd=HistorySearch&querykey=110) |
| [**#109**](https://www.ncbi.nlm.nih.gov/pubmed) | bougie*[tiab] | [**1605**](https://www.ncbi.nlm.nih.gov/pubmed/?cmd=HistorySearch&querykey=109) |
| [**#107**](https://www.ncbi.nlm.nih.gov/pubmed) | (("Surgical Stapling"[Mesh] OR stapl*[tiab]) AND reinforc*[tiab]) | [**477**](https://www.ncbi.nlm.nih.gov/pubmed/?cmd=HistorySearch&querykey=107) |
| [**#105**](https://www.ncbi.nlm.nih.gov/pubmed) | (resecti*[tiab] AND ("Pylorus"[Mesh] OR pylor*[tiab])) | [**2238**](https://www.ncbi.nlm.nih.gov/pubmed/?cmd=HistorySearch&querykey=105) |
| [**#104**](https://www.ncbi.nlm.nih.gov/pubmed) | (sleeve gastrectom*[tiab] OR gastric sleeve*[tiab] OR ((stomach[ti] OR gastric[ti]) AND sleeve*[ti]) OR (vertical*[tiab] AND gastroplast*[tiab])) | [**5262**](https://www.ncbi.nlm.nih.gov/pubmed/?cmd=HistorySearch&querykey=104) |

## Embase.com (22 November 2018)

### T4Qa: Gastric plication (18 SRs, 10 RCTs, 29 cohort studies)

| **No.** | **Query** | **Results** |
| --- | --- | --- |
| **#17** | #11 AND #16 NOT (#13 OR #15) | **29** |
| **#16** | 'epidemiology'/de OR (((cohort OR case) NEAR/3 (control OR controll* OR comparison OR referent)):ti,ab,kw) OR risk:ti,ab,kw OR causation:ti,ab,kw OR causal:ti,ab,kw OR 'odds ratio':ti,ab,kw OR etiol*:ti,ab,kw OR aetiol*:ti,ab,kw OR 'natural history':ti,ab,kw OR outcome:ti,ab,kw OR course:ti,ab,kw OR retrospect*:ti,ab,kw OR 'follow up':ti,ab,kw OR followup:ti,ab,kw OR predict*:ti,ab,kw OR prognos*:ti,ab,kw | **7332240** |
| **#15** | #11 AND #14 NOT #13 | **10** |
| **#14** | 'clinical':ti,ab,kw AND 'trial':ti,ab,kw OR 'clinical trial'/exp OR random*:ti,ab,kw | **2291866** |
| **#13** | #11 AND #12 | **18** |
| **#12** | 'meta-analysis':ti,ab,kw OR 'meta analysis'/exp OR 'review'/exp OR review:ti,ab,kw | **3439352** |
| **#11** | #10 NOT (('adolescent'/exp OR 'child'/exp OR adolescent*:ti,ab OR child*:ti,ab OR schoolchild*:ti,ab OR infant*:ti,ab OR girl*:ti,ab OR boy*:ti,ab OR teen:ti,ab OR teens:ti,ab OR teenager*:ti,ab OR youth*:ti,ab OR pediatr*:ti,ab OR paediatr*:ti,ab OR puber*:ti,ab) NOT ('adult'/exp OR 'aged'/exp OR 'middle aged'/exp OR adult*:ti,ab OR man:ti,ab OR men:ti,ab OR woman:ti,ab OR women:ti,ab)) | **72** |
| **#10** | #9 NOT ('conference abstract'/it OR 'conference paper'/it OR 'letter'/it OR 'note'/it) | **72** |
| **#9** | #8 NOT ([animals]/lim NOT [humans]/lim) AND [1-1-2004]/sd | **222** |
| **#8** | #4 AND #7 | **224** |
| **#7** | #5 AND #2 OR #6 | **13941** |
| **#6** | lagb*:ti,ab,kw OR 'lap band*':ti,ab,kw OR lrygb*:ti,ab,kw OR 'laparoscopic sleeve gastrectomy'/exp | **6199** |
| **#5** | 'gastric bypass surgery'/exp/mj OR 'roux-en-y gastric bypass'/exp/mj OR 'vertical sleeve gastrectomy'/exp/mj OR ((('bilio pancreatic' OR biliopancreatic) NEAR/3 (diversion* OR bypass*)):ti,kw) OR ((duodenal NEAR/3 switch*):ti,kw) OR (((stomach OR gastric) NEAR/3 (band* OR bypass* OR sleeve*)):ti,kw) OR ((sleeve NEAR/3 gastrectom*):ti,kw) OR ((roux NEAR/2 y):ti,kw) OR mgbp*:ti,kw OR oagb*:ti,kw OR rya:ti,kw OR rygb*:ti,kw OR sadis:ti,kw OR 'sadi s':ti,kw OR sagb*:ti,kw | **23203** |
| **#4** | #1 AND #2 OR #3 | **1421** |
| **#3** | lgp*:ti,ab,kw OR lgcp*:ti,ab,kw | **1146** |
| **#2** | 'laparoscopy'/de OR 'laparoendoscopic single site surgery'/exp OR 'laparoscopic surgery'/exp OR 'minimally invasive procedure'/exp OR 'minimally invasive surgery'/exp OR laparoscop*:ti,ab,kw OR ((minimal* NEAR/3 (invasive OR access OR surg*)):ti,ab,kw) | **297422** |
| **#1** | 'gastric plication'/exp OR (((gastric* OR curvature) NEAR/3 plicati*):ti,ab,kw) | **665** |

### T4Qb: SADIS or DS (37 SRs, 6 RCTs, 55 cohort studies)

| **No.** | **Query** | **Results** |
| --- | --- | --- |
| **#16** | #13 AND #4 NOT (#14 OR #15) | **55** |
| **#15** | #13 AND #3 NOT #14 | **6** |
| **#14** | #2 AND #13 | **37** |
| **#13** | #12 NOT (('adolescent'/exp OR 'child'/exp OR adolescent*:ti,ab OR child*:ti,ab OR schoolchild*:ti,ab OR infant*:ti,ab OR girl*:ti,ab OR boy*:ti,ab OR teen:ti,ab OR teens:ti,ab OR teenager*:ti,ab OR youth*:ti,ab OR pediatr*:ti,ab OR paediatr*:ti,ab OR puber*:ti,ab) NOT ('adult'/exp OR 'aged'/exp OR 'middle aged'/exp OR adult*:ti,ab OR man:ti,ab OR men:ti,ab OR woman:ti,ab OR women:ti,ab)) | **149** |
| **#12** | #11 NOT ('conference abstract'/it OR 'conference paper'/it OR 'letter'/it OR 'note'/it) | **149** |
| **#11** | #10 NOT ([animals]/lim NOT [humans]/lim) AND [1-1-2004]/sd | **274** |
| **#10** | #6 AND #9 | **287** |
| **#9** | #7 AND #1 OR #8 | **13870** |
| **#8** | lagb*:ti,ab,kw OR 'lap band*':ti,ab,kw OR lrygb*:ti,ab,kw OR 'laparoscopic sleeve gastrectomy'/exp | **6199** |
| **#7** | 'gastric bypass surgery'/exp/mj OR 'roux-en-y gastric bypass'/exp/mj OR 'vertical sleeve gastrectomy'/exp/mj OR ((('bilio pancreatic' OR biliopancreatic) NEAR/3 (diversion* OR bypass*)):ti,kw) OR (((stomach OR gastric) NEAR/3 (band* OR bypass* OR sleeve*)):ti,kw) OR ((sleeveNEAR/3 gastrectom*):ti,kw) OR ((roux NEAR/2 y):ti,kw) OR mgbp*:ti,kw OR oagb*:ti,kw OR rya:ti,kw OR rygb*:ti,kw OR sagb*:ti,kw | **22996** |
| **#6** | #1 AND #5 | **358** |
| **#5** | ((duodenal NEAR/3 switch*):ti,kw) OR sadis:ti,kw OR 'sadi s':ti,kw OR 'single anastomosis bypass*':ti,kw OR 'single anastomosis duodenoileal switch':ti,kw OR 'single anastomosis duodeno ileal switch':ti,kw | **728** |
| **#4** | 'epidemiology'/de OR (((cohort OR case) NEAR/3 (control OR controll* OR comparison OR referent)):ti,ab,kw) OR risk:ti,ab,kw OR causation:ti,ab,kw OR causal:ti,ab,kw OR 'odds ratio':ti,ab,kw OR etiol*:ti,ab,kw OR aetiol*:ti,ab,kw OR 'natural history':ti,ab,kw OR outcome:ti,ab,kw OR course:ti,ab,kw OR retrospect*:ti,ab,kw OR 'follow up':ti,ab,kw OR followup:ti,ab,kw OR predict*:ti,ab,kw OR prognos*:ti,ab,kw | **7332240** |
| **#3** | 'clinical':ti,ab,kw AND 'trial':ti,ab,kw OR 'clinical trial'/exp OR random*:ti,ab,kw | **2291866** |
| **#2** | 'meta-analysis':ti,ab,kw OR 'meta analysis'/exp OR 'review'/exp OR review:ti,ab,kw | **3439352** |
| **#1** | 'laparoscopy'/de OR 'laparoendoscopic single site surgery'/exp OR 'laparoscopic surgery'/exp OR 'minimally invasive procedure'/exp OR 'minimally invasive surgery'/exp OR laparoscop*:ti,ab,kw OR ((minimal* NEAR/3 (invasive OR access OR surg*)):ti,ab,kw) | **297422** |

### T4Qc: BPD (42 SRs, 6 RCTs, 28 cohort studies)

| **No.** | **Query** | **Results** |
| --- | --- | --- |
| **#16** | #13 AND #4 NOT (#14 OR #15) | **28** |
| **#15** | #13 AND #3 NOT #14 | **6** |
| **#14** | #13 AND #2 | **42** |
| **#13** | #12 NOT (('adolescent'/exp OR 'child'/exp OR adolescent*:ti,ab OR child*:ti,ab OR schoolchild*:ti,ab OR infant*:ti,ab OR girl*:ti,ab OR boy*:ti,ab OR teen:ti,ab OR teens:ti,ab OR teenager*:ti,ab OR youth*:ti,ab OR pediatr*:ti,ab OR paediatr*:ti,ab OR puber*:ti,ab) NOT ('adult'/exp OR 'aged'/exp OR 'middle aged'/exp OR adult*:ti,ab OR man:ti,ab OR men:ti,ab OR woman:ti,ab OR women:ti,ab)) | **114** |
| **#12** | #11 NOT ('conference abstract'/it OR 'conference paper'/it OR 'letter'/it OR 'note'/it) | **115** |
| **#11** | #10 NOT ([animals]/lim NOT [humans]/lim) AND [1-1-2004]/sd | **149** |
| **#10** | #6 AND #9 | **156** |
| **#9** | #7 AND #1 OR #8 | **13744** |
| **#8** | lagb*:ti,ab,kw OR 'lap band*':ti,ab,kw OR lrygb*:ti,ab,kw OR 'laparoscopic sleeve gastrectomy'/exp | **6199** |
| **#7** | 'gastric bypass surgery'/exp/mj OR 'roux-en-y gastric bypass'/exp/mj OR 'vertical sleeve gastrectomy'/exp/mj OR (((stomach OR gastric) NEAR/3 (band* OR bypass* OR sleeve*)):ti,kw) OR ((sleeve NEAR/3 gastrectom*):ti,kw) OR ((roux NEAR/2 y):ti,kw) OR mgbp*:ti,kw OR oagb*:ti,kw OR rya:ti,kw OR rygb*:ti,kw OR sagb*:ti,kw | **22374** |
| **#6** | #1 AND #5 | **282** |
| **#5** | (('bilio pancreatic' OR biliopancreatic) NEAR/3 (diversion* OR bypass*)):ti,kw | **931** |
| **#4** | 'epidemiology'/de OR (((cohort OR case) NEAR/3 (control OR controll* OR comparison OR referent)):ti,ab,kw) OR risk:ti,ab,kw OR causation:ti,ab,kw OR causal:ti,ab,kw OR 'odds ratio':ti,ab,kw OR etiol*:ti,ab,kw OR aetiol*:ti,ab,kw OR 'natural history':ti,ab,kw OR outcome:ti,ab,kw OR course:ti,ab,kw OR retrospect*:ti,ab,kw OR 'follow up':ti,ab,kw OR followup:ti,ab,kw OR predict*:ti,ab,kw OR prognos*:ti,ab,kw | **7332240** |
| **#3** | 'clinical':ti,ab,kw AND 'trial':ti,ab,kw OR 'clinical trial'/exp OR random*:ti,ab,kw | **2291866** |
| **#2** | 'meta-analysis':ti,ab,kw OR 'meta analysis'/exp OR 'review'/exp OR review:ti,ab,kw | **3439352** |
| **#1** | 'laparoscopy'/de OR 'laparoendoscopic single site surgery'/exp OR 'laparoscopic surgery'/exp OR 'minimally invasive procedure'/exp OR 'minimally invasive surgery'/exp OR laparoscop*:ti,ab,kw OR ((minimal* NEAR/3 (invasive OR access OR surg*)):ti,ab,kw) | **297422** |

### T4Qd: MGB (30 SRs, 22 RCTs, 77 cohort studies)

| **No.** | **Query** | **Results** |
| --- | --- | --- |
| **#16** | #13 AND #4 NOT (#14 OR #15) | **77** |
| **#15** | #13 AND #3 NOT #14 | **22** |
| **#14** | #2 AND #13 | **30** |
| **#13** | #12 NOT (('adolescent'/exp OR 'child'/exp OR adolescent*:ti,ab OR child*:ti,ab OR schoolchild*:ti,ab OR infant*:ti,ab OR girl*:ti,ab OR boy*:ti,ab OR teen:ti,ab OR teens:ti,ab OR teenager*:ti,ab OR youth*:ti,ab OR pediatr*:ti,ab OR paediatr*:ti,ab OR puber*:ti,ab) NOT ('adult'/exp OR 'aged'/exp OR 'middle aged'/exp OR adult*:ti,ab OR man:ti,ab OR men:ti,ab OR woman:ti,ab OR women:ti,ab)) | **173** |
| **#12** | #11 NOT ('conference abstract'/it OR 'conference paper'/it OR 'letter'/it OR 'note'/it) | **175** |
| **#11** | #10 NOT ([animals]/lim NOT [humans]/lim) AND [1-1-2004]/sd | **530** |
| **#10** | #6 AND #9 | **533** |
| **#9** | #7 AND #1 OR #8 | **13744** |
| **#8** | lagb*:ti,ab,kw OR 'lap band*':ti,ab,kw OR lrygb*:ti,ab,kw OR 'laparoscopic sleeve gastrectomy'/exp | **6199** |
| **#7** | 'gastric bypass surgery'/exp/mj OR 'roux-en-y gastric bypass'/exp/mj OR 'vertical sleeve gastrectomy'/exp/mj OR (((stomach OR gastric) NEAR/3 (band* OR bypass* OR sleeve*)):ti,kw) OR ((sleeve NEAR/3 gastrectom*):ti,kw) OR ((roux NEAR/2 y):ti,kw) OR oagb*:ti,kw OR rya:ti,kw OR rygb*:ti,kw OR sagb*:ti,kw | **22354** |
| **#6** | #5 AND #1 | **633** |
| **#5** | 'mini gastric bypass*':ti,ab,kw OR mgb*:ti,ab,kw OR 'omega gastric bypass*':ti,ab,kw OR 'single anastomos*':ti,ab,kw OR 'single anastomosis gastric bypass*':ti,ab,kw OR 'one anastomosis gastric bypass*':ti,ab,kw OR mgbp*:ti,ab,kw | **3498** |
| **#4** | 'epidemiology'/de OR (((cohort OR case) NEAR/3 (control OR controll* OR comparison OR referent)):ti,ab,kw) OR risk:ti,ab,kw OR causation:ti,ab,kw OR causal:ti,ab,kw OR 'odds ratio':ti,ab,kw OR etiol*:ti,ab,kw OR aetiol*:ti,ab,kw OR 'natural history':ti,ab,kw OR outcome:ti,ab,kw OR course:ti,ab,kw OR retrospect*:ti,ab,kw OR 'follow up':ti,ab,kw OR followup:ti,ab,kw OR predict*:ti,ab,kw OR prognos*:ti,ab,kw | **7332240** |
| **#3** | 'clinical':ti,ab,kw AND 'trial':ti,ab,kw OR 'clinical trial'/exp OR random*:ti,ab,kw | **2291866** |
| **#2** | 'meta-analysis':ti,ab,kw OR 'meta analysis'/exp OR 'review'/exp OR review:ti,ab,kw | **3439352** |
| **#1** | 'laparoscopy'/de OR 'laparoendoscopic single site surgery'/exp OR 'laparoscopic surgery'/exp OR 'minimally invasive procedure'/exp OR 'minimally invasive surgery'/exp OR laparoscop*:ti,ab,kw OR ((minimal* NEAR/3 (invasive OR access OR surg*)):ti,ab,kw) | **297422** |

### T4Qe: Banding (146 SRs, 51 RCTs, 180 cohort studies)

| **No.** | **Query** | **Results** |
| --- | --- | --- |
| **#18** | #15 AND #4 NOT (#16 OR #17) | **180** |
| **#17** | #15 AND #3 NOT #16 | **51** |
| **#16** | #15 AND #2 | **146** |
| **#15** | #14 NOT (('adolescent'/exp OR 'child'/exp OR adolescent*:ti,ab OR child*:ti,ab OR schoolchild*:ti,ab OR infant*:ti,ab OR girl*:ti,ab OR boy*:ti,ab OR teen:ti,ab OR teens:ti,ab OR teenager*:ti,ab OR youth*:ti,ab OR pediatr*:ti,ab OR paediatr*:ti,ab OR puber*:ti,ab) NOT ('adult'/exp OR 'aged'/exp OR 'middle aged'/exp OR adult*:ti,ab OR man:ti,ab OR men:ti,ab OR woman:ti,ab OR women:ti,ab)) | **472** |
| **#14** | #13 NOT ('conference abstract'/it OR 'conference paper'/it OR 'letter'/it OR 'note'/it) | **480** |
| **#13** | #12 NOT ([animals]/lim NOT [humans]/lim) AND [1-1-2004]/sd | **969** |
| **#12** | #11 NOT (revision*:ti,kw OR revising*:ti,kw OR redo:ti,kw) | **1002** |
| **#11** | #7 AND #10 | **1223** |
| **#10** | #8 AND #1 OR #9 | **10350** |
| **#9** | lrygb*:ti,ab,kw | **2014** |
| **#8** | 'gastric bypass surgery'/exp/mj OR 'roux-en-y gastric bypass'/exp/mj OR 'vertical sleeve gastrectomy'/exp/mj OR (((stomach OR gastric) NEAR/3 (bypass* OR sleeve*)):ti,kw) OR ((sleeve NEAR/3 gastrectom*):ti,kw) OR ((roux NEAR/2 y):ti,kw) OR rya:ti,kw OR rygb*:ti,kw | **19548** |
| **#7** | #5 AND #1 OR #6 | **4163** |
| **#6** | lagb*:ti,ab,kw OR 'lap band*':ti,ab,kw | **2676** |
| **#5** | sagb*:ti,kw OR oagb*:ti,kw OR (((stomach OR gastric OR esophagogastric OR oesophagogastric) NEAR/3 band*):ti,kw) | **4131** |
| **#4** | 'epidemiology'/de OR (((cohort OR case) NEAR/3 (control OR controll* OR comparison OR referent)):ti,ab,kw) OR risk:ti,ab,kw OR causation:ti,ab,kw OR causal:ti,ab,kw OR 'odds ratio':ti,ab,kw OR etiol*:ti,ab,kw OR aetiol*:ti,ab,kw OR 'natural history':ti,ab,kw OR outcome:ti,ab,kw OR course:ti,ab,kw OR retrospect*:ti,ab,kw OR 'follow up':ti,ab,kw OR followup:ti,ab,kw OR predict*:ti,ab,kw OR prognos*:ti,ab,kw | **7332240** |
| **#3** | 'clinical':ti,ab,kw AND 'trial':ti,ab,kw OR 'clinical trial'/exp OR random*:ti,ab,kw | **2291866** |
| **#2** | 'meta-analysis':ti,ab,kw OR 'meta analysis'/exp OR 'review'/exp OR review:ti,ab,kw | **3439352** |
| **#1** | 'laparoscopy'/de OR 'laparoendoscopic single site surgery'/exp OR 'laparoscopic surgery'/exp OR 'minimally invasive procedure'/exp OR 'minimally invasive surgery'/exp OR laparoscop*:ti,ab,kw OR ((minimal* NEAR/3 (invasive OR access OR surg*)):ti,ab,kw) | **297422** |

### T4Qf: Sleeve (153 SRs, 78 RCTs, 226 cohort studies)

| **No.** | **Query** | **Results** |
| --- | --- | --- |
| **#18** | #15 AND #4 NOT (#16 OR #17) | **226** |
| **#17** | #15 AND #3 NOT #16 | **78** |
| **#16** | #15 AND #2 | **153** |
| **#15** | #14 NOT (('adolescent'/exp OR 'child'/exp OR adolescent*:ti,ab OR child*:ti,ab OR schoolchild*:ti,ab OR infant*:ti,ab OR girl*:ti,ab OR boy*:ti,ab OR teen:ti,ab OR teens:ti,ab OR teenager*:ti,ab OR youth*:ti,ab OR pediatr*:ti,ab OR paediatr*:ti,ab OR puber*:ti,ab) NOT ('adult'/exp OR 'aged'/exp OR 'middle aged'/exp OR adult*:ti,ab OR man:ti,ab OR men:ti,ab OR woman:ti,ab OR women:ti,ab)) | **569** |
| **#14** | #13 NOT ('conference abstract'/it OR 'conference paper'/it OR 'letter'/it OR 'note'/it) | **577** |
| **#13** | #12 NOT ([animals]/lim NOT [humans]/lim) AND [1-1-2004]/sd | **1086** |
| **#12** | #11 NOT (revision*:ti,kw OR revising*:ti,kw OR redo:ti,kw) | **1099** |
| **#11** | #7 AND #10 | **1241** |
| **#10** | #8 AND #1 OR #9 | **6603** |
| **#9** | lrygb*:ti,ab,kw | **2014** |
| **#8** | 'gastric bypass surgery'/exp/mj OR 'roux-en-y gastric bypass'/exp/mj OR (((stomach OR gastric) NEAR/3 bypass*):ti,kw) OR ((roux NEAR/2 y):ti,kw) OR rya:ti,kw OR rygb*:ti,kw | **14403** |
| **#7** | #5 AND #1 OR #6 | **5583** |
| **#6** | 'laparoscopic sleeve gastrectomy'/exp | **2117** |
| **#5** | 'vertical sleeve gastrectomy'/exp OR (((stomach OR gastric OR gastrectom*) NEAR/3 sleeve*):ti,kw) OR ((vertical NEAR/3 gastroplast*):ti,kw) | **7403** |
| **#4** | 'epidemiology'/de OR (((cohort OR case) NEAR/3 (control OR controll* OR comparison OR referent)):ti,ab,kw) OR risk:ti,ab,kw OR causation:ti,ab,kw OR causal:ti,ab,kw OR 'odds ratio':ti,ab,kw OR etiol*:ti,ab,kw OR aetiol*:ti,ab,kw OR 'natural history':ti,ab,kw OR outcome:ti,ab,kw OR course:ti,ab,kw OR retrospect*:ti,ab,kw OR 'follow up':ti,ab,kw OR followup:ti,ab,kw OR predict*:ti,ab,kw OR prognos*:ti,ab,kw | **7332240** |
| **#3** | 'clinical':ti,ab,kw AND 'trial':ti,ab,kw OR 'clinical trial'/exp OR random*:ti,ab,kw | **2291866** |
| **#2** | 'meta-analysis':ti,ab,kw OR 'meta analysis'/exp OR 'review'/exp OR review:ti,ab,kw | **3439352** |
| **#1** | 'laparoscopy'/de OR 'laparoendoscopic single site surgery'/exp OR 'laparoscopic surgery'/exp OR 'minimally invasive procedure'/exp OR 'minimally invasive surgery'/exp OR laparoscop*:ti,ab,kw OR ((minimal* NEAR/3 (invasive OR access OR surg*)):ti,ab,kw) | **297422** |

### T4Q14: Sleeve + resection, stapling, bougie (47 SRs, 42 RCTs, 94 cohort studies)

| **No.** | **Query** | **Results** |
| --- | --- | --- |
| **#17** | #14 AND #4 NOT (#15 OR #16) | **94** |
| **#16** | #14 AND #3 NOT #15 | **42** |
| **#15** | #14 AND #2 | **47** |
| **#14** | #13 NOT (('adolescent'/exp OR 'child'/exp OR adolescent*:ti,ab OR child*:ti,ab OR schoolchild*:ti,ab OR infant*:ti,ab OR girl*:ti,ab OR boy*:ti,ab OR teen:ti,ab OR teens:ti,ab OR teenager*:ti,ab OR youth*:ti,ab OR pediatr*:ti,ab OR paediatr*:ti,ab OR puber*:ti,ab) NOT ('adult'/exp OR 'aged'/exp OR 'middle aged'/exp OR adult*:ti,ab OR man:ti,ab OR men:ti,ab OR woman:ti,ab OR women:ti,ab)) | **227** |
| **#13** | #12 NOT ('conference abstract'/it OR 'conference paper'/it OR 'letter'/it OR 'note'/it) | **228** |
| **#12** | #11 NOT ([animals]/lim NOT [humans]/lim) AND [1-1-2004]/sd | **645** |
| **#11** | #7 AND (#8 OR #9 OR #10) | **660** |
| **#10** | 'bougie'/exp OR bougie*:ti,ab,kw | **3097** |
| **#9** | ('surgical stapling'/exp OR stapl*:ti,ab,kw) AND reinforc*:ti,ab,kw | **946** |
| **#8** | resecti*:ti,ab,kw AND ('pylorus'/de OR pylor*:ti,ab,kw) | **3569** |
| **#7** | #5 AND #1 OR #6 | **5583** |
| **#6** | 'laparoscopic sleeve gastrectomy'/exp | **2117** |
| **#5** | 'vertical sleeve gastrectomy'/exp OR (((stomach OR gastric OR gastrectom*) NEAR/3 sleeve*):ti,kw) OR ((vertical NEAR/3 gastroplast*):ti,kw) | **7403** |
| **#4** | 'epidemiology'/de OR (((cohort OR case) NEAR/3 (control OR controll* OR comparison OR referent)):ti,ab,kw) OR risk:ti,ab,kw OR causation:ti,ab,kw OR causal:ti,ab,kw OR 'odds ratio':ti,ab,kw OR etiol*:ti,ab,kw OR aetiol*:ti,ab,kw OR 'natural history':ti,ab,kw OR outcome:ti,ab,kw OR course:ti,ab,kw OR retrospect*:ti,ab,kw OR 'follow up':ti,ab,kw OR followup:ti,ab,kw OR predict*:ti,ab,kw OR prognos*:ti,ab,kw | **7332240** |
| **#3** | 'clinical':ti,ab,kw AND 'trial':ti,ab,kw OR 'clinical trial'/exp OR random*:ti,ab,kw | **2291866** |
| **#2** | 'meta-analysis':ti,ab,kw OR 'meta analysis'/exp OR 'review'/exp OR review:ti,ab,kw | **3439352** |
| **#1** | 'laparoscopy'/de OR 'laparoendoscopic single site surgery'/exp OR 'laparoscopic surgery'/exp OR 'minimally invasive procedure'/exp OR 'minimally invasive surgery'/exp OR laparoscop*:ti,ab,kw OR ((minimal* NEAR/3 (invasive OR access OR surg*)):ti,ab,kw) | **297422** |

# Topic 5: Revisional surgery

## PubMed (14 November 2018)

### T5Qa: Resleeve (30 all study types)

| **Search** | **Query** | **Items found** |
| --- | --- | --- |
| [**#12**](https://www.ncbi.nlm.nih.gov/pubmed) | (#11 NOT (("Adolescent"[Mesh] OR "Child"[Mesh] OR "Infant"[Mesh] OR adolescen*[tiab] OR child*[tiab] OR schoolchild*[tiab] OR infant*[tiab] OR girl*[tiab] OR boy*[tiab] OR teen[tiab] OR teens[tiab] OR teenager*[tiab] OR youth*[tiab] OR pediatr*[tiab] OR paediatr*[tiab] OR puber*[tiab]) NOT ("Adult"[Mesh] OR adult*[tiab] OR man[tiab] OR men[tiab] OR woman[tiab] OR women[tiab]))) | [**30**](https://www.ncbi.nlm.nih.gov/pubmed/?cmd=HistorySearch&querykey=12) |
| [**#11**](https://www.ncbi.nlm.nih.gov/pubmed) | (#10 NOT ("Animals"[Mesh] NOT "Humans"[Mesh]) AND ("2004"[Date - Entrez] : "3000"[Date - Entrez])) | [**30**](https://www.ncbi.nlm.nih.gov/pubmed/?cmd=HistorySearch&querykey=11) |
| [**#10**](https://www.ncbi.nlm.nih.gov/pubmed) | re sleev*[tiab] OR resleev*[tiab] | [**31**](https://www.ncbi.nlm.nih.gov/pubmed/?cmd=HistorySearch&querykey=10) |

### T5Qb: RYGB (37 SRs, 18 RCTs, 72 cohort studies)

| **Search** | **Query** | **Items found** |
| --- | --- | --- |
| [**#35**](https://www.ncbi.nlm.nih.gov/pubmed) | (#29 AND #34) NOT (#31 OR #33) | [**72**](https://www.ncbi.nlm.nih.gov/pubmed/?cmd=HistorySearch&querykey=35) |
| [**#34**](https://www.ncbi.nlm.nih.gov/pubmed) | "Epidemiologic Studies"[Mesh] OR "Prognosis"[Mesh] OR cohort[tiab] OR (case[tiab] AND (control[tiab] OR controll*[tiab] OR comparison[tiab] OR referent[tiab])) OR risk[tiab] OR causation[tiab] OR causal[tiab] OR "odds ratio"[tiab] OR etiol*[tiab] OR aetiol*[tiab] OR "natural history"[tiab] OR predict*[tiab] OR outcome[tiab] OR course[tiab] OR retrospect*[tiab] OR “follow up”[tiab] OR followup[tiab] OR prognos*[tiab] | [**6600830**](https://www.ncbi.nlm.nih.gov/pubmed/?cmd=HistorySearch&querykey=34) |
| [**#33**](https://www.ncbi.nlm.nih.gov/pubmed) | #29 AND #32 NOT #31 | [**18**](https://www.ncbi.nlm.nih.gov/pubmed/?cmd=HistorySearch&querykey=33) |
| [**#32**](https://www.ncbi.nlm.nih.gov/pubmed) | "Randomized Controlled Trial"[Publication Type] OR "Controlled Clinical Trial"[Publication Type] OR random*[tiab] OR placebo[tiab] OR "Drug Therapy"[Subheading] OR trial[tiab] OR groups[tiab] | [**4570304**](https://www.ncbi.nlm.nih.gov/pubmed/?cmd=HistorySearch&querykey=32) |
| [**#31**](https://www.ncbi.nlm.nih.gov/pubmed) | #29 AND #30 | [**37**](https://www.ncbi.nlm.nih.gov/pubmed/?cmd=HistorySearch&querykey=31) |
| [**#30**](https://www.ncbi.nlm.nih.gov/pubmed) | (review[tiab] OR "Review"[Publication Type] OR "Meta-Analysis as Topic"[Mesh] OR meta-analysis[tiab] OR "Meta-Analysis"[Publication Type]) NOT ("Letter"[Publication Type] OR "Editorial"[Publication Type] OR "Comment"[Publication Type]) | [**2993615**](https://www.ncbi.nlm.nih.gov/pubmed/?cmd=HistorySearch&querykey=30) |
| [**#29**](https://www.ncbi.nlm.nih.gov/pubmed) | #28 NOT (("Adolescent"[Mesh] OR "Child"[Mesh] OR "Infant"[Mesh] OR adolescen*[tiab] OR child*[tiab] OR schoolchild*[tiab] OR infant*[tiab] OR girl*[tiab] OR boy*[tiab] OR teen[tiab] OR teens[tiab] OR teenager*[tiab] OR youth*[tiab] OR pediatr*[tiab] OR paediatr*[tiab] OR puber*[tiab]) NOT ("Adult"[Mesh] OR adult*[tiab] OR man[tiab] OR men[tiab] OR woman[tiab] OR women[tiab])) | [**136**](https://www.ncbi.nlm.nih.gov/pubmed/?cmd=HistorySearch&querykey=29) |
| [**#28**](https://www.ncbi.nlm.nih.gov/pubmed) | #27 NOT ("Animals"[Mesh] NOT "Humans"[Mesh]) AND ("2004"[Date - Entrez] : "3000"[Date - Entrez]) | [**137**](https://www.ncbi.nlm.nih.gov/pubmed/?cmd=HistorySearch&querykey=28) |
| [**#27**](https://www.ncbi.nlm.nih.gov/pubmed) | #24 AND #25 AND #26 | [**141**](https://www.ncbi.nlm.nih.gov/pubmed/?cmd=HistorySearch&querykey=27) |
| [**#26**](https://www.ncbi.nlm.nih.gov/pubmed) | "Weight Gain"[Mesh] OR (weight[tiab] AND (gain*[tiab] OR regain*[tiab])) | [**89107**](https://www.ncbi.nlm.nih.gov/pubmed/?cmd=HistorySearch&querykey=26) |
| [**#25**](https://www.ncbi.nlm.nih.gov/pubmed) | "Reoperation"[Mesh] OR revision*[tiab] OR revising*[tiab] OR redo*[tiab] OR repeat*[tiab] | [**718108**](https://www.ncbi.nlm.nih.gov/pubmed/?cmd=HistorySearch&querykey=25) |
| [**#24**](https://www.ncbi.nlm.nih.gov/pubmed) | (("Anastomosis, Roux-en-Y"[Mesh] OR roux en y*[tiab] OR “roux y”[tiab] OR “roux n y”[tiab] OR “roux in y”[tiab] OR rya[tiab] OR rygb*[tiab] OR (roux[ti] AND y[ti])) AND ("Laparoscopy"[Mesh:NoExp] OR "Minimally Invasive Surgical Procedures"[Mesh:NoExp] OR laparoscop*[tiab] OR minimally invasive[tiab] OR minimal invasive[tiab] OR minimal access[tiab] OR minimal surg*[tiab] OR (minimal*[ti] AND invasive[ti]) OR (minimal[ti] AND (access[ti] OR surg*[ti])))) OR (lrygb*[tiab] OR lrygb*[tiab]) | [**3845**](https://www.ncbi.nlm.nih.gov/pubmed/?cmd=HistorySearch&querykey=24) |

### T5Qc: BPD/DS or SADI-S (28 SRs, 14 RCTs, 67 cohort studies)

| **Search** | **Query** | **Items found** |
| --- | --- | --- |
| [**#42**](https://www.ncbi.nlm.nih.gov/pubmed) | (#39 AND #34) NOT (#40 OR #41) | [**67**](https://www.ncbi.nlm.nih.gov/pubmed/?cmd=HistorySearch&querykey=42) |
| [**#41**](https://www.ncbi.nlm.nih.gov/pubmed) | #39 AND #32 NOT #40 | [**14**](https://www.ncbi.nlm.nih.gov/pubmed/?cmd=HistorySearch&querykey=41) |
| [**#40**](https://www.ncbi.nlm.nih.gov/pubmed) | #39 AND #30 | [**28**](https://www.ncbi.nlm.nih.gov/pubmed/?cmd=HistorySearch&querykey=40) |
| [**#39**](https://www.ncbi.nlm.nih.gov/pubmed) | #38 NOT (("Adolescent"[Mesh] OR "Child"[Mesh] OR "Infant"[Mesh] OR adolescen*[tiab] OR child*[tiab] OR schoolchild*[tiab] OR infant*[tiab] OR girl*[tiab] OR boy*[tiab] OR teen[tiab] OR teens[tiab] OR teenager*[tiab] OR youth*[tiab] OR pediatr*[tiab] OR paediatr*[tiab] OR puber*[tiab]) NOT ("Adult"[Mesh] OR adult*[tiab] OR man[tiab] OR men[tiab] OR woman[tiab] OR women[tiab])) | [**122**](https://www.ncbi.nlm.nih.gov/pubmed/?cmd=HistorySearch&querykey=39) |
| [**#38**](https://www.ncbi.nlm.nih.gov/pubmed) | #37 NOT ("Animals"[Mesh] NOT "Humans"[Mesh]) AND ("2004"[Date - Entrez] : "3000"[Date - Entrez]) | [**122**](https://www.ncbi.nlm.nih.gov/pubmed/?cmd=HistorySearch&querykey=38) |
| [**#37**](https://www.ncbi.nlm.nih.gov/pubmed) | #36 AND #25 | [**129**](https://www.ncbi.nlm.nih.gov/pubmed/?cmd=HistorySearch&querykey=37) |
| [**#36**](https://www.ncbi.nlm.nih.gov/pubmed) | ("Biliopancreatic Diversion"[Mesh] OR bilio pancreatic bypass*[tiab] OR bilio pancreatic diversion*[tiab] OR biliopancreatic bypass*[tiab] OR biliopancreatic diversion*[tiab] OR duodenal switch*[tiab] OR sadis[tiab] OR "sadi s"[tiab] OR ((biliopancreatic[ti] OR "bilio pancreatic"[ti]) AND (diversion*[ti] OR bypass*[ti])) OR (duodenal[ti] AND switch*[ti])) AND ("Laparoscopy"[Mesh:noexp] OR "Minimally Invasive Surgical Procedures"[Mesh:noexp] OR laparoscop*[tiab] OR minimally invasive[tiab] OR minimal invasive[tiab] OR minimal access[tiab] OR minimal surg*[tiab] OR (minimal*[ti] AND invasive[ti]) OR (minimal[ti] AND (access[ti] OR surg*[ti]))) | [**569**](https://www.ncbi.nlm.nih.gov/pubmed/?cmd=HistorySearch&querykey=36) |
| [**#34**](https://www.ncbi.nlm.nih.gov/pubmed) | "Epidemiologic Studies"[Mesh] OR "Prognosis"[Mesh] OR cohort[tiab] OR (case[tiab] AND (control[tiab] OR controll*[tiab] OR comparison[tiab] OR referent[tiab])) OR risk[tiab] OR causation[tiab] OR causal[tiab] OR "odds ratio"[tiab] OR etiol*[tiab] OR aetiol*[tiab] OR "natural history"[tiab] OR predict*[tiab] OR outcome[tiab] OR course[tiab] OR retrospect*[tiab] OR “follow up”[tiab] OR followup[tiab] OR prognos*[tiab] | [**6600830**](https://www.ncbi.nlm.nih.gov/pubmed/?cmd=HistorySearch&querykey=34) |
| [**#32**](https://www.ncbi.nlm.nih.gov/pubmed) | "Randomized Controlled Trial"[Publication Type] OR "Controlled Clinical Trial"[Publication Type] OR random*[tiab] OR placebo[tiab] OR "Drug Therapy"[Subheading] OR trial[tiab] OR groups[tiab] | [**4570304**](https://www.ncbi.nlm.nih.gov/pubmed/?cmd=HistorySearch&querykey=32) |
| [**#30**](https://www.ncbi.nlm.nih.gov/pubmed) | (review[tiab] OR "Review"[Publication Type] OR "Meta-Analysis as Topic"[Mesh] OR meta-analysis[tiab] OR "Meta-Analysis"[Publication Type]) NOT ("Letter"[Publication Type] OR "Editorial"[Publication Type] OR "Comment"[Publication Type]) | [**2993615**](https://www.ncbi.nlm.nih.gov/pubmed/?cmd=HistorySearch&querykey=30) |
| [**#25**](https://www.ncbi.nlm.nih.gov/pubmed) | "Reoperation"[Mesh] OR revision*[tiab] OR revising*[tiab] OR redo*[tiab] OR repeat*[tiab] | [**718108**](https://www.ncbi.nlm.nih.gov/pubmed/?cmd=HistorySearch&querykey=25) |

### T5Qd: Sleeve gastrectomy (20 SRs, 11 RCTs, 63 cohort studies)

| **Search** | **Query** | **Items found** |
| --- | --- | --- |
| [**#49**](https://www.ncbi.nlm.nih.gov/pubmed) | (#46 AND #34) NOT (#47 OR #48) | [**63**](https://www.ncbi.nlm.nih.gov/pubmed/?cmd=HistorySearch&querykey=49) |
| [**#48**](https://www.ncbi.nlm.nih.gov/pubmed) | #46 AND #32 NOT #47 | [**11**](https://www.ncbi.nlm.nih.gov/pubmed/?cmd=HistorySearch&querykey=48) |
| [**#47**](https://www.ncbi.nlm.nih.gov/pubmed) | #46 AND #30 | [**20**](https://www.ncbi.nlm.nih.gov/pubmed/?cmd=HistorySearch&querykey=47) |
| [**#46**](https://www.ncbi.nlm.nih.gov/pubmed) | #45 NOT (("Adolescent"[Mesh] OR "Child"[Mesh] OR "Infant"[Mesh] OR adolescen*[tiab] OR child*[tiab] OR schoolchild*[tiab] OR infant*[tiab] OR girl*[tiab] OR boy*[tiab] OR teen[tiab] OR teens[tiab] OR teenager*[tiab] OR youth*[tiab] OR pediatr*[tiab] OR paediatr*[tiab] OR puber*[tiab]) NOT ("Adult"[Mesh] OR adult*[tiab] OR man[tiab] OR men[tiab] OR woman[tiab] OR women[tiab])) | [**100**](https://www.ncbi.nlm.nih.gov/pubmed/?cmd=HistorySearch&querykey=46) |
| [**#45**](https://www.ncbi.nlm.nih.gov/pubmed) | #44 NOT ("Animals"[Mesh] NOT "Humans"[Mesh]) AND ("2004"[Date - Entrez] : "3000"[Date - Entrez]) | [**101**](https://www.ncbi.nlm.nih.gov/pubmed/?cmd=HistorySearch&querykey=45) |
| [**#44**](https://www.ncbi.nlm.nih.gov/pubmed) | #43 AND #25 AND #26 | [**102**](https://www.ncbi.nlm.nih.gov/pubmed/?cmd=HistorySearch&querykey=44) |
| [**#43**](https://www.ncbi.nlm.nih.gov/pubmed) | ((sleeve gastrectom*[tiab] OR ((stomach[ti] OR gastric[ti]) AND sleeve*[ti]) OR (sleeve[ti] AND (gastrectom*[ti] OR "Gastrectomy"[Mesh])) OR gastric sleeve*[tiab]) AND ("Laparoscopy"[Mesh:NoExp] OR "Minimally Invasive Surgical Procedures"[Mesh:NoExp] OR laparoscop*[tiab] OR minimally invasive[tiab] OR minimal invasive[tiab] OR minimal access[tiab] OR (minimal*[ti] AND invasive[ti]) OR (minimal[ti] AND (access[ti] OR surg*[ti])) OR minimal surg*[tiab])) | [**2849**](https://www.ncbi.nlm.nih.gov/pubmed/?cmd=HistorySearch&querykey=43) |
| [**#34**](https://www.ncbi.nlm.nih.gov/pubmed) | "Epidemiologic Studies"[Mesh] OR "Prognosis"[Mesh] OR cohort[tiab] OR (case[tiab] AND (control[tiab] OR controll*[tiab] OR comparison[tiab] OR referent[tiab])) OR risk[tiab] OR causation[tiab] OR causal[tiab] OR "odds ratio"[tiab] OR etiol*[tiab] OR aetiol*[tiab] OR "natural history"[tiab] OR predict*[tiab] OR outcome[tiab] OR course[tiab] OR retrospect*[tiab] OR “follow up”[tiab] OR followup[tiab] OR prognos*[tiab] | [**6600830**](https://www.ncbi.nlm.nih.gov/pubmed/?cmd=HistorySearch&querykey=34) |
| [**#32**](https://www.ncbi.nlm.nih.gov/pubmed) | "Randomized Controlled Trial"[Publication Type] OR "Controlled Clinical Trial"[Publication Type] OR random*[tiab] OR placebo[tiab] OR "Drug Therapy"[Subheading] OR trial[tiab] OR groups[tiab] | [**4570304**](https://www.ncbi.nlm.nih.gov/pubmed/?cmd=HistorySearch&querykey=32) |
| [**#30**](https://www.ncbi.nlm.nih.gov/pubmed) | (review[tiab] OR "Review"[Publication Type] OR "Meta-Analysis as Topic"[Mesh] OR meta-analysis[tiab] OR "Meta-Analysis"[Publication Type]) NOT ("Letter"[Publication Type] OR "Editorial"[Publication Type] OR "Comment"[Publication Type]) | [**2993615**](https://www.ncbi.nlm.nih.gov/pubmed/?cmd=HistorySearch&querykey=30) |
| [**#26**](https://www.ncbi.nlm.nih.gov/pubmed) | "Weight Gain"[Mesh] OR (weight[tiab] AND (gain*[tiab] OR regain*[tiab])) | [**89107**](https://www.ncbi.nlm.nih.gov/pubmed/?cmd=HistorySearch&querykey=26) |
| [**#25**](https://www.ncbi.nlm.nih.gov/pubmed) | "Reoperation"[Mesh] OR revision*[tiab] OR revising*[tiab] OR redo*[tiab] OR repeat*[tiab] | [**718108**](https://www.ncbi.nlm.nih.gov/pubmed/?cmd=HistorySearch&querykey=25) |

### T5Qe: Limb lengthening (9 all study types)

| **Search** | **Query** | **Items found** |
| --- | --- | --- |
| [**#54**](https://www.ncbi.nlm.nih.gov/pubmed/advanced) | #53 NOT (("Adolescent"[Mesh] OR "Child"[Mesh] OR "Infant"[Mesh] OR adolescen*[tiab] OR child*[tiab] OR schoolchild*[tiab] OR infant*[tiab] OR girl*[tiab] OR boy*[tiab] OR teen[tiab] OR teens[tiab] OR teenager*[tiab] OR youth*[tiab] OR pediatr*[tiab] OR paediatr*[tiab] OR puber*[tiab]) NOT ("Adult"[Mesh] OR adult*[tiab] OR man[tiab] OR men[tiab] OR woman[tiab] OR women[tiab])) | [**9**](https://www.ncbi.nlm.nih.gov/pubmed/?cmd=HistorySearch&querykey=54) |
| [**#53**](https://www.ncbi.nlm.nih.gov/pubmed/advanced) | #52 NOT ("Animals"[Mesh] NOT "Humans"[Mesh]) AND ("2004"[Date - Entrez] : "3000"[Date - Entrez]) | [**9**](https://www.ncbi.nlm.nih.gov/pubmed/?cmd=HistorySearch&querykey=53) |
| [**#52**](https://www.ncbi.nlm.nih.gov/pubmed/advanced) | #50 AND #51 | [**9**](https://www.ncbi.nlm.nih.gov/pubmed/?cmd=HistorySearch&querykey=52) |
| [**#51**](https://www.ncbi.nlm.nih.gov/pubmed/advanced) | (("Bariatric Surgery"[Mesh] OR "Obesity/surgery"[Mesh:NoExp] OR "Obesity, Abdominal/surgery"[Mesh] OR "Obesity, Metabolically Benign/surgery"[Mesh] OR "Obesity, Morbid/surgery"[Mesh] OR "Anastomosis, Roux-en-Y"[Mesh] OR "Biliopancreatic Diversion"[Mesh] OR "Overweight/surgery"[Mesh] OR "Gastrectomy"[Mesh] OR bariatric surg*[tiab] OR bariatric operati*[tiab] OR bariatric procedure*[tiab] OR bilio pancreatic bypass*[tiab] OR bilio pancreatic diversion*[tiab] OR biliopancreatic bypass*[tiab] OR biliopancreatic diversion*[tiab] OR duodenal switch*[tiab] OR gastric band*[tiab] OR stomach band*[tiab] OR gastric bypass*[tiab] OR gastric partiti*[tiab] OR gastric plicati*[tiab] OR gastroplast*[tiab] OR mgbp*[tiab] OR oagb*[tiab] OR obese surg*[tiab] OR weight loss surg*[tiab] OR obesity surg*[tiab] OR metabolic surg*[tiab] OR roux en y*[tiab] OR “roux y”[tiab] OR “roux n y”[tiab] OR “roux in y”[tiab] OR rya[tiab] OR rygb*[tiab] OR sadis[tiab] OR “sadi s”[tiab] OR sagb*[tiab] OR sleeve gastrectom*[tiab] OR gastric sleeve*[tiab] OR gastrectom*[tiab] OR ((bariatric[ti] OR obes*[ti] OR weight loss[ti] OR metabolic[ti]) AND (surg*[ti] OR operati*[ti] OR procedure*[ti])) OR ((biliopancreatic[ti] OR “bilio pancreatic”[ti]) AND (diversion*[ti] OR bypass*[ti])) OR (duodenal[ti] AND switch*[ti]) OR ((stomach[ti] OR gastric[ti]) AND (band*[ti] OR bypass*[ti] OR partiti*[ti] OR plicati*[ti] OR sleeve*[ti])) OR (sleeve[ti] AND gastrectom*[ti]) OR (roux[ti] AND y[ti])) AND ("Laparoscopy"[Mesh:NoExp] OR "Minimally Invasive Surgical Procedures"[Mesh:NoExp] OR laparoscop*[tiab] OR minimally invasive[tiab] OR minimal invasive[tiab] OR minimal access[tiab] OR minimal surg*[tiab] OR (minimal*[ti] AND invasive[ti]) OR (minimal[ti] AND (access[ti] OR surg*[ti])))) OR (lagb*[tiab] OR lrygb*[tiab] OR lap band*[tiab] OR lrygb*[tiab]) | [**13079**](https://www.ncbi.nlm.nih.gov/pubmed/?cmd=HistorySearch&querykey=51) |
| [**#50**](https://www.ncbi.nlm.nih.gov/pubmed/advanced) | limb[tiab] AND lengthen*[tiab] | [**1949**](https://www.ncbi.nlm.nih.gov/pubmed/?cmd=HistorySearch&querykey=50) |
| [**#26**](https://www.ncbi.nlm.nih.gov/pubmed/advanced) | "Weight Gain"[Mesh] OR (weight[tiab] AND (gain*[tiab] OR regain*[tiab])) | [**89107**](https://www.ncbi.nlm.nih.gov/pubmed/?cmd=HistorySearch&querykey=26) |
| [**#25**](https://www.ncbi.nlm.nih.gov/pubmed/advanced) | "Reoperation"[Mesh] OR revision*[tiab] OR revising*[tiab] OR redo*[tiab] OR repeat*[tiab] | [**718108**](https://www.ncbi.nlm.nih.gov/pubmed/?cmd=HistorySearch&querykey=25) |

### T5Qf: Gastric bypass banding (49 SRs, 24 RCTs, 117 cohort studies)

| **Search** | **Query** | **Items found** |
| --- | --- | --- |
| [#62](https://www.ncbi.nlm.nih.gov/pubmed) | (#59 AND #34) NOT (#60 OR #61) | [117](https://www.ncbi.nlm.nih.gov/pubmed/?cmd=HistorySearch&querykey=62) |
| [#61](https://www.ncbi.nlm.nih.gov/pubmed) | #59 AND #32 NOT #60 | [24](https://www.ncbi.nlm.nih.gov/pubmed/?cmd=HistorySearch&querykey=61) |
| [#60](https://www.ncbi.nlm.nih.gov/pubmed) | #59 AND #30 | [49](https://www.ncbi.nlm.nih.gov/pubmed/?cmd=HistorySearch&querykey=60) |
| [#59](https://www.ncbi.nlm.nih.gov/pubmed) | #58 NOT (("Adolescent"[Mesh] OR "Child"[Mesh] OR "Infant"[Mesh] OR adolescen*[tiab] OR child*[tiab] OR schoolchild*[tiab] OR infant*[tiab] OR girl*[tiab] OR boy*[tiab] OR teen[tiab] OR teens[tiab] OR teenager*[tiab] OR youth*[tiab] OR pediatr*[tiab] OR paediatr*[tiab] OR puber*[tiab]) NOT ("Adult"[Mesh] OR adult*[tiab] OR man[tiab] OR men[tiab] OR woman[tiab] OR women[tiab])) | [205](https://www.ncbi.nlm.nih.gov/pubmed/?cmd=HistorySearch&querykey=59) |
| [#58](https://www.ncbi.nlm.nih.gov/pubmed) | #57 NOT ("Animals"[Mesh] NOT "Humans"[Mesh]) AND ("2004"[Date - Entrez] : "3000"[Date - Entrez]) | [208](https://www.ncbi.nlm.nih.gov/pubmed/?cmd=HistorySearch&querykey=58) |
| [#57](https://www.ncbi.nlm.nih.gov/pubmed) | #55 AND #25 AND #26 | [218](https://www.ncbi.nlm.nih.gov/pubmed/?cmd=HistorySearch&querykey=57) |
| [#55](https://www.ncbi.nlm.nih.gov/pubmed) | ((gastric band*[tiab] OR stomach band*[tiab] OR mgbp*[tiab] OR oagb*[tiab] OR ((stomach[ti] OR gastric[ti]) AND (band*[ti] OR bypass*[ti])) OR gastric bypass*[tiab]) AND ("Laparoscopy"[Mesh:noexp] OR "Minimally Invasive Surgical Procedures"[Mesh:noexp] OR laparoscop*[tiab] OR minimally invasive[tiab] OR minimal invasive[tiab] OR minimal access[tiab] OR minimal surg*[tiab] OR (minimal*[ti] AND invasive[ti]) OR (minimal[ti] AND (access[ti] OR surg*[ti])))) OR (lagb*[tiab] OR lap band*[tiab]) | [5976](https://www.ncbi.nlm.nih.gov/pubmed/?cmd=HistorySearch&querykey=55) |
| [#34](https://www.ncbi.nlm.nih.gov/pubmed) | "Epidemiologic Studies"[Mesh] OR "Prognosis"[Mesh] OR cohort[tiab] OR (case[tiab] AND (control[tiab] OR controll*[tiab] OR comparison[tiab] OR referent[tiab])) OR risk[tiab] OR causation[tiab] OR causal[tiab] OR "odds ratio"[tiab] OR etiol*[tiab] OR aetiol*[tiab] OR "natural history"[tiab] OR predict*[tiab] OR outcome[tiab] OR course[tiab] OR retrospect*[tiab] OR “follow up”[tiab] OR followup[tiab] OR prognos*[tiab] | [6600830](https://www.ncbi.nlm.nih.gov/pubmed/?cmd=HistorySearch&querykey=34) |
| [#32](https://www.ncbi.nlm.nih.gov/pubmed) | "Randomized Controlled Trial"[Publication Type] OR "Controlled Clinical Trial"[Publication Type] OR random*[tiab] OR placebo[tiab] OR "Drug Therapy"[Subheading] OR trial[tiab] OR groups[tiab] | [4570304](https://www.ncbi.nlm.nih.gov/pubmed/?cmd=HistorySearch&querykey=32) |
| [#30](https://www.ncbi.nlm.nih.gov/pubmed) | (review[tiab] OR "Review"[Publication Type] OR "Meta-Analysis as Topic"[Mesh] OR meta-analysis[tiab] OR "Meta-Analysis"[Publication Type]) NOT ("Letter"[Publication Type] OR "Editorial"[Publication Type] OR "Comment"[Publication Type]) | [2993615](https://www.ncbi.nlm.nih.gov/pubmed/?cmd=HistorySearch&querykey=30) |
| [#26](https://www.ncbi.nlm.nih.gov/pubmed) | "Weight Gain"[Mesh] OR (weight[tiab] AND (gain*[tiab] OR regain*[tiab])) | [89107](https://www.ncbi.nlm.nih.gov/pubmed/?cmd=HistorySearch&querykey=26) |
| [#25](https://www.ncbi.nlm.nih.gov/pubmed) | "Reoperation"[Mesh] OR revision*[tiab] OR revising*[tiab] OR redo*[tiab] OR repeat*[tiab] | [718108](https://www.ncbi.nlm.nih.gov/pubmed/?cmd=HistorySearch&querykey=25) |

### T5Qg: Pouch resizing (33 SRs, 25 RCTs, 130 cohort studies)

| **Search** | **Query** | **Items found** |
| --- | --- | --- |
| [**#38**](https://www.ncbi.nlm.nih.gov/pubmed) | (#32 AND #37) NOT (#34 OR #36) | [**130**](https://www.ncbi.nlm.nih.gov/pubmed/?cmd=HistorySearch&querykey=38) |
| [**#37**](https://www.ncbi.nlm.nih.gov/pubmed) | "Epidemiologic Studies"[Mesh] OR "Prognosis"[Mesh] OR cohort[tiab] OR (case[tiab] AND (control[tiab] OR controll*[tiab] OR comparison[tiab] OR referent[tiab])) OR risk[tiab] OR causation[tiab] OR causal[tiab] OR "odds ratio"[tiab] OR etiol*[tiab] OR aetiol*[tiab] OR "natural history"[tiab] OR predict*[tiab] OR outcome[tiab] OR course[tiab] OR retrospect*[tiab] OR “follow up”[tiab] OR followup[tiab] OR prognos*[tiab] | [**6600830**](https://www.ncbi.nlm.nih.gov/pubmed/?cmd=HistorySearch&querykey=37) |
| [**#36**](https://www.ncbi.nlm.nih.gov/pubmed) | #32 AND #35 NOT #34 | [**25**](https://www.ncbi.nlm.nih.gov/pubmed/?cmd=HistorySearch&querykey=36) |
| [**#35**](https://www.ncbi.nlm.nih.gov/pubmed) | "Randomized Controlled Trial"[Publication Type] OR "Controlled Clinical Trial"[Publication Type] OR random*[tiab] OR placebo[tiab] OR "Drug Therapy"[Subheading] OR trial[tiab] OR groups[tiab] | [**4570304**](https://www.ncbi.nlm.nih.gov/pubmed/?cmd=HistorySearch&querykey=35) |
| [**#34**](https://www.ncbi.nlm.nih.gov/pubmed) | #32 AND #33 | [**33**](https://www.ncbi.nlm.nih.gov/pubmed/?cmd=HistorySearch&querykey=34) |
| [**#33**](https://www.ncbi.nlm.nih.gov/pubmed) | (review[tiab] OR "Review"[Publication Type] OR "Meta-Analysis as Topic"[Mesh] OR meta-analysis[tiab] OR "Meta-Analysis"[Publication Type]) NOT ("Letter"[Publication Type] OR "Editorial"[Publication Type] OR "Comment"[Publication Type]) | [**2993615**](https://www.ncbi.nlm.nih.gov/pubmed/?cmd=HistorySearch&querykey=33) |
| [**#32**](https://www.ncbi.nlm.nih.gov/pubmed) | #31 NOT (("Adolescent"[Mesh] OR "Child"[Mesh] OR "Infant"[Mesh] OR adolescen*[tiab] OR child*[tiab] OR schoolchild*[tiab] OR infant*[tiab] OR girl*[tiab] OR boy*[tiab] OR teen[tiab] OR teens[tiab] OR teenager*[tiab] OR youth*[tiab] OR pediatr*[tiab] OR paediatr*[tiab] OR puber*[tiab]) NOT ("Adult"[Mesh] OR adult*[tiab] OR man[tiab] OR men[tiab] OR woman[tiab] OR women[tiab])) | [**222**](https://www.ncbi.nlm.nih.gov/pubmed/?cmd=HistorySearch&querykey=32) |
| [**#31**](https://www.ncbi.nlm.nih.gov/pubmed) | #30 NOT ("Animals"[Mesh] NOT "Humans"[Mesh]) AND ("2004"[Date - Entrez] : "3000"[Date - Entrez]) | [**224**](https://www.ncbi.nlm.nih.gov/pubmed/?cmd=HistorySearch&querykey=31) |
| [**#30**](https://www.ncbi.nlm.nih.gov/pubmed) | #25 AND #29 | [**280**](https://www.ncbi.nlm.nih.gov/pubmed/?cmd=HistorySearch&querykey=30) |
| [**#29**](https://www.ncbi.nlm.nih.gov/pubmed) | (pouch*[tiab] AND (resiz*[tiab] OR upsiz*[tiab] OR downsiz*[tiab] OR size[tiab] OR sizing*[tiab] OR sizes[tiab] OR reshap*[tiab] OR shape*[tiab] OR shaping*[tiab] OR formation*[tiab] OR reconstruct*[tiab] OR "Reoperation"[Mesh] OR revision*[tiab] OR revising*[tiab] OR redo*[tiab] OR repeat*[tiab])) | [**5032**](https://www.ncbi.nlm.nih.gov/pubmed/?cmd=HistorySearch&querykey=29) |
| [**#25**](https://www.ncbi.nlm.nih.gov/pubmed) | (("Bariatric Surgery"[Mesh] OR "Obesity/surgery"[Mesh:NoExp] OR "Obesity, Abdominal/surgery"[Mesh] OR "Obesity, Metabolically Benign/surgery"[Mesh] OR "Obesity, Morbid/surgery"[Mesh] OR "Anastomosis, Roux-en-Y"[Mesh] OR "Biliopancreatic Diversion"[Mesh] OR "Overweight/surgery"[Mesh] OR "Gastrectomy"[Mesh] OR bariatric surg*[tiab] OR bariatric operati*[tiab] OR bariatric procedure*[tiab] OR bilio pancreatic bypass*[tiab] OR bilio pancreatic diversion*[tiab] OR biliopancreatic bypass*[tiab] OR biliopancreatic diversion*[tiab] OR duodenal switch*[tiab] OR gastric band*[tiab] OR stomach band*[tiab] OR gastric bypass*[tiab] OR gastric partiti*[tiab] OR gastric plicati*[tiab] OR gastroplast*[tiab] OR mgbp*[tiab] OR oagb*[tiab] OR obese surg*[tiab] OR weight loss surg*[tiab] OR obesity surg*[tiab] OR metabolic surg*[tiab] OR roux en y*[tiab] OR “roux y”[tiab] OR “roux n y”[tiab] OR “roux in y”[tiab] OR rya[tiab] OR rygb*[tiab] OR sadis[tiab] OR “sadi s”[tiab] OR sagb*[tiab] OR sleeve gastrectom*[tiab] OR gastric sleeve*[tiab] OR gastrectom*[tiab] OR ((bariatric[ti] OR obes*[ti] OR weight loss[ti] OR metabolic[ti]) AND (surg*[ti] OR operati*[ti] OR procedure*[ti])) OR ((biliopancreatic[ti] OR “bilio pancreatic”[ti]) AND (diversion*[ti] OR bypass*[ti])) OR (duodenal[ti] AND switch*[ti]) OR ((stomach[ti] OR gastric[ti]) AND (band*[ti] OR bypass*[ti] OR partiti*[ti] OR plicati*[ti] OR sleeve*[ti])) OR (sleeve[ti] AND gastrectom*[ti]) OR (roux[ti] AND y[ti])) AND ("Laparoscopy"[Mesh:NoExp] OR "Minimally Invasive Surgical Procedures"[Mesh:NoExp] OR laparoscop*[tiab] OR minimally invasive[tiab] OR minimal invasive[tiab] OR minimal access[tiab] OR minimal surg*[tiab] OR (minimal*[ti] AND invasive[ti]) OR (minimal[ti] AND (access[ti] OR surg*[ti])))) OR (lagb*[tiab] OR lrygb*[tiab] OR lap band*[tiab] OR lrygb*[tiab]) | [**13079**](https://www.ncbi.nlm.nih.gov/pubmed/?cmd=HistorySearch&querykey=25) |

### T5Qh: Alimentary limb (12 SRs, 23 RCTs, 50 cohort studies)

| **Search** | **Query** | **Items found** |
| --- | --- | --- |
| [**#51**](https://www.ncbi.nlm.nih.gov/pubmed) | (#48 AND #37) NOT (#49 OR #50) | [**50**](https://www.ncbi.nlm.nih.gov/pubmed/?cmd=HistorySearch&querykey=51) |
| [**#50**](https://www.ncbi.nlm.nih.gov/pubmed) | #48 AND #35 NOT #49 | [**23**](https://www.ncbi.nlm.nih.gov/pubmed/?cmd=HistorySearch&querykey=50) |
| [**#49**](https://www.ncbi.nlm.nih.gov/pubmed) | #48 AND #33 | [**12**](https://www.ncbi.nlm.nih.gov/pubmed/?cmd=HistorySearch&querykey=49) |
| [**#48**](https://www.ncbi.nlm.nih.gov/pubmed) | #47 NOT (("Adolescent"[Mesh] OR "Child"[Mesh] OR "Infant"[Mesh] OR adolescen*[tiab] OR child*[tiab] OR schoolchild*[tiab] OR infant*[tiab] OR girl*[tiab] OR boy*[tiab] OR teen[tiab] OR teens[tiab] OR teenager*[tiab] OR youth*[tiab] OR pediatr*[tiab] OR paediatr*[tiab] OR puber*[tiab]) NOT ("Adult"[Mesh] OR adult*[tiab] OR man[tiab] OR men[tiab] OR woman[tiab] OR women[tiab])) | [**107**](https://www.ncbi.nlm.nih.gov/pubmed/?cmd=HistorySearch&querykey=48) |
| [**#47**](https://www.ncbi.nlm.nih.gov/pubmed) | #46 NOT ("Animals"[Mesh] NOT "Humans"[Mesh]) AND ("2004"[Date - Entrez] : "3000"[Date - Entrez]) | [**108**](https://www.ncbi.nlm.nih.gov/pubmed/?cmd=HistorySearch&querykey=47) |
| [**#46**](https://www.ncbi.nlm.nih.gov/pubmed) | #25 AND #45 | [**118**](https://www.ncbi.nlm.nih.gov/pubmed/?cmd=HistorySearch&querykey=46) |
| [**#45**](https://www.ncbi.nlm.nih.gov/pubmed) | alimentary limb*[tiab] OR biliopancreatic limb*[tiab] OR bilio pancreatic limb*[tiab] OR long limb*[tiab] OR short limb*[tiab] OR standard limb*[tiab] | [**1344**](https://www.ncbi.nlm.nih.gov/pubmed/?cmd=HistorySearch&querykey=45) |
| [**#37**](https://www.ncbi.nlm.nih.gov/pubmed) | "Epidemiologic Studies"[Mesh] OR "Prognosis"[Mesh] OR cohort[tiab] OR (case[tiab] AND (control[tiab] OR controll*[tiab] OR comparison[tiab] OR referent[tiab])) OR risk[tiab] OR causation[tiab] OR causal[tiab] OR "odds ratio"[tiab] OR etiol*[tiab] OR aetiol*[tiab] OR "natural history"[tiab] OR predict*[tiab] OR outcome[tiab] OR course[tiab] OR retrospect*[tiab] OR “follow up”[tiab] OR followup[tiab] OR prognos*[tiab] | [**6600830**](https://www.ncbi.nlm.nih.gov/pubmed/?cmd=HistorySearch&querykey=37) |
| [**#35**](https://www.ncbi.nlm.nih.gov/pubmed) | "Randomized Controlled Trial"[Publication Type] OR "Controlled Clinical Trial"[Publication Type] OR random*[tiab] OR placebo[tiab] OR "Drug Therapy"[Subheading] OR trial[tiab] OR groups[tiab] | [**4570304**](https://www.ncbi.nlm.nih.gov/pubmed/?cmd=HistorySearch&querykey=35) |
| [**#33**](https://www.ncbi.nlm.nih.gov/pubmed) | (review[tiab] OR "Review"[Publication Type] OR "Meta-Analysis as Topic"[Mesh] OR meta-analysis[tiab] OR "Meta-Analysis"[Publication Type]) NOT ("Letter"[Publication Type] OR "Editorial"[Publication Type] OR "Comment"[Publication Type]) | [**2993615**](https://www.ncbi.nlm.nih.gov/pubmed/?cmd=HistorySearch&querykey=33) |
| [**#25**](https://www.ncbi.nlm.nih.gov/pubmed) | (("Bariatric Surgery"[Mesh] OR "Obesity/surgery"[Mesh:NoExp] OR "Obesity, Abdominal/surgery"[Mesh] OR "Obesity, Metabolically Benign/surgery"[Mesh] OR "Obesity, Morbid/surgery"[Mesh] OR "Anastomosis, Roux-en-Y"[Mesh] OR "Biliopancreatic Diversion"[Mesh] OR "Overweight/surgery"[Mesh] OR "Gastrectomy"[Mesh] OR bariatric surg*[tiab] OR bariatric operati*[tiab] OR bariatric procedure*[tiab] OR bilio pancreatic bypass*[tiab] OR bilio pancreatic diversion*[tiab] OR biliopancreatic bypass*[tiab] OR biliopancreatic diversion*[tiab] OR duodenal switch*[tiab] OR gastric band*[tiab] OR stomach band*[tiab] OR gastric bypass*[tiab] OR gastric partiti*[tiab] OR gastric plicati*[tiab] OR gastroplast*[tiab] OR mgbp*[tiab] OR oagb*[tiab] OR obese surg*[tiab] OR weight loss surg*[tiab] OR obesity surg*[tiab] OR metabolic surg*[tiab] OR roux en y*[tiab] OR “roux y”[tiab] OR “roux n y”[tiab] OR “roux in y”[tiab] OR rya[tiab] OR rygb*[tiab] OR sadis[tiab] OR “sadi s”[tiab] OR sagb*[tiab] OR sleeve gastrectom*[tiab] OR gastric sleeve*[tiab] OR gastrectom*[tiab] OR ((bariatric[ti] OR obes*[ti] OR weight loss[ti] OR metabolic[ti]) AND (surg*[ti] OR operati*[ti] OR procedure*[ti])) OR ((biliopancreatic[ti] OR “bilio pancreatic”[ti]) AND (diversion*[ti] OR bypass*[ti])) OR (duodenal[ti] AND switch*[ti]) OR ((stomach[ti] OR gastric[ti]) AND (band*[ti] OR bypass*[ti] OR partiti*[ti] OR plicati*[ti] OR sleeve*[ti])) OR (sleeve[ti] AND gastrectom*[ti]) OR (roux[ti] AND y[ti])) AND ("Laparoscopy"[Mesh:NoExp] OR "Minimally Invasive Surgical Procedures"[Mesh:NoExp] OR laparoscop*[tiab] OR minimally invasive[tiab] OR minimal invasive[tiab] OR minimal access[tiab] OR minimal surg*[tiab] OR (minimal*[ti] AND invasive[ti]) OR (minimal[ti] AND (access[ti] OR surg*[ti])))) OR (lagb*[tiab] OR lrygb*[tiab] OR lap band*[tiab] OR lrygb*[tiab]) | [**13079**](https://www.ncbi.nlm.nih.gov/pubmed/?cmd=HistorySearch&querykey=25) |

### T5Qi: Watchful waiting (3 all study types)

| **Search** | **Query** | **Items found** |
| --- | --- | --- |
| [**#56**](https://www.ncbi.nlm.nih.gov/pubmed/advanced) | #25 AND #53 | [**3**](https://www.ncbi.nlm.nih.gov/pubmed/?cmd=HistorySearch&querykey=56) |
| [**#55**](https://www.ncbi.nlm.nih.gov/pubmed/advanced) | #25 AND #53 AND #54 | [**0**](https://www.ncbi.nlm.nih.gov/pubmed/?cmd=HistorySearch&querykey=55) |
| [**#54**](https://www.ncbi.nlm.nih.gov/pubmed/advanced) | "Reoperation"[Mesh] OR revision*[tiab] OR revising*[tiab] OR redo*[tiab] OR repeat*[tiab] | [**718108**](https://www.ncbi.nlm.nih.gov/pubmed/?cmd=HistorySearch&querykey=54) |
| [**#53**](https://www.ncbi.nlm.nih.gov/pubmed/advanced) | "Watchful Waiting"[Mesh] OR watchful wait*[tiab] OR “watch and wait” [tiab] OR “wait and watch”[tiab] | [**5436**](https://www.ncbi.nlm.nih.gov/pubmed/?cmd=HistorySearch&querykey=53) |
| [**#25**](https://www.ncbi.nlm.nih.gov/pubmed/advanced) | (("Bariatric Surgery"[Mesh] OR "Obesity/surgery"[Mesh:NoExp] OR "Obesity, Abdominal/surgery"[Mesh] OR "Obesity, Metabolically Benign/surgery"[Mesh] OR "Obesity, Morbid/surgery"[Mesh] OR "Anastomosis, Roux-en-Y"[Mesh] OR "Biliopancreatic Diversion"[Mesh] OR "Overweight/surgery"[Mesh] OR "Gastrectomy"[Mesh] OR bariatric surg*[tiab] OR bariatric operati*[tiab] OR bariatric procedure*[tiab] OR bilio pancreatic bypass*[tiab] OR bilio pancreatic diversion*[tiab] OR biliopancreatic bypass*[tiab] OR biliopancreatic diversion*[tiab] OR duodenal switch*[tiab] OR gastric band*[tiab] OR stomach band*[tiab] OR gastric bypass*[tiab] OR gastric partiti*[tiab] OR gastric plicati*[tiab] OR gastroplast*[tiab] OR mgbp*[tiab] OR oagb*[tiab] OR obese surg*[tiab] OR weight loss surg*[tiab] OR obesity surg*[tiab] OR metabolic surg*[tiab] OR roux en y*[tiab] OR “roux y”[tiab] OR “roux n y”[tiab] OR “roux in y”[tiab] OR rya[tiab] OR rygb*[tiab] OR sadis[tiab] OR “sadi s”[tiab] OR sagb*[tiab] OR sleeve gastrectom*[tiab] OR gastric sleeve*[tiab] OR gastrectom*[tiab] OR ((bariatric[ti] OR obes*[ti] OR weight loss[ti] OR metabolic[ti]) AND (surg*[ti] OR operati*[ti] OR procedure*[ti])) OR ((biliopancreatic[ti] OR “bilio pancreatic”[ti]) AND (diversion*[ti] OR bypass*[ti])) OR (duodenal[ti] AND switch*[ti]) OR ((stomach[ti] OR gastric[ti]) AND (band*[ti] OR bypass*[ti] OR partiti*[ti] OR plicati*[ti] OR sleeve*[ti])) OR (sleeve[ti] AND gastrectom*[ti]) OR (roux[ti] AND y[ti])) AND ("Laparoscopy"[Mesh:NoExp] OR "Minimally Invasive Surgical Procedures"[Mesh:NoExp] OR laparoscop*[tiab] OR minimally invasive[tiab] OR minimal invasive[tiab] OR minimal access[tiab] OR minimal surg*[tiab] OR (minimal*[ti] AND invasive[ti]) OR (minimal[ti] AND (access[ti] OR surg*[ti])))) OR (lagb*[tiab] OR lrygb*[tiab] OR lap band*[tiab] OR lrygb*[tiab]) | [**13079**](https://www.ncbi.nlm.nih.gov/pubmed/?cmd=HistorySearch&querykey=25) |

### T5Qj: Hiatal hernia repair (48 all study types)

| **Search** | **Query** | **Items found** |
| --- | --- | --- |
| [**#66**](https://www.ncbi.nlm.nih.gov/pubmed) | #65 NOT (("Adolescent"[Mesh] OR "Child"[Mesh] OR "Infant"[Mesh] OR adolescen*[tiab] OR child*[tiab] OR schoolchild*[tiab] OR infant*[tiab] OR girl*[tiab] OR boy*[tiab] OR teen[tiab] OR teens[tiab] OR teenager*[tiab] OR youth*[tiab] OR pediatr*[tiab] OR paediatr*[tiab] OR puber*[tiab]) NOT ("Adult"[Mesh] OR adult*[tiab] OR man[tiab] OR men[tiab] OR woman[tiab] OR women[tiab])) | [**48**](https://www.ncbi.nlm.nih.gov/pubmed/?cmd=HistorySearch&querykey=66) |
| [**#65**](https://www.ncbi.nlm.nih.gov/pubmed) | #64 NOT ("Animals"[Mesh] NOT "Humans"[Mesh]) AND ("2004"[Date - Entrez] : "3000"[Date - Entrez]) | [**49**](https://www.ncbi.nlm.nih.gov/pubmed/?cmd=HistorySearch&querykey=65) |
| [**#64**](https://www.ncbi.nlm.nih.gov/pubmed) | #59 AND #25 AND #63 | [**50**](https://www.ncbi.nlm.nih.gov/pubmed/?cmd=HistorySearch&querykey=64) |
| [**#63**](https://www.ncbi.nlm.nih.gov/pubmed) | "Reoperation"[Mesh] OR revision*[tiab] OR revising*[tiab] OR redo*[tiab] OR repeat*[tiab] repair*[tiab] | [**20158**](https://www.ncbi.nlm.nih.gov/pubmed/?cmd=HistorySearch&querykey=63) |
| [**#59**](https://www.ncbi.nlm.nih.gov/pubmed) | "Hernia, Hiatal"[Mesh] OR hiatal hernia*[tiab] OR hiatus hernia*[tiab] OR esophageal hernia*[tiab] OR paraesophageal hernia*[tiab] OR oesophageal hernia*[tiab] OR paraoesophageal hernia*[tiab] | [**8894**](https://www.ncbi.nlm.nih.gov/pubmed/?cmd=HistorySearch&querykey=59) |
| [**#25**](https://www.ncbi.nlm.nih.gov/pubmed) | (("Bariatric Surgery"[Mesh] OR "Obesity/surgery"[Mesh:NoExp] OR "Obesity, Abdominal/surgery"[Mesh] OR "Obesity, Metabolically Benign/surgery"[Mesh] OR "Obesity, Morbid/surgery"[Mesh] OR "Anastomosis, Roux-en-Y"[Mesh] OR "Biliopancreatic Diversion"[Mesh] OR "Overweight/surgery"[Mesh] OR "Gastrectomy"[Mesh] OR bariatric surg*[tiab] OR bariatric operati*[tiab] OR bariatric procedure*[tiab] OR bilio pancreatic bypass*[tiab] OR bilio pancreatic diversion*[tiab] OR biliopancreatic bypass*[tiab] OR biliopancreatic diversion*[tiab] OR duodenal switch*[tiab] OR gastric band*[tiab] OR stomach band*[tiab] OR gastric bypass*[tiab] OR gastric partiti*[tiab] OR gastric plicati*[tiab] OR gastroplast*[tiab] OR mgbp*[tiab] OR oagb*[tiab] OR obese surg*[tiab] OR weight loss surg*[tiab] OR obesity surg*[tiab] OR metabolic surg*[tiab] OR roux en y*[tiab] OR “roux y”[tiab] OR “roux n y”[tiab] OR “roux in y”[tiab] OR rya[tiab] OR rygb*[tiab] OR sadis[tiab] OR “sadi s”[tiab] OR sagb*[tiab] OR sleeve gastrectom*[tiab] OR gastric sleeve*[tiab] OR gastrectom*[tiab] OR ((bariatric[ti] OR obes*[ti] OR weight loss[ti] OR metabolic[ti]) AND (surg*[ti] OR operati*[ti] OR procedure*[ti])) OR ((biliopancreatic[ti] OR “bilio pancreatic”[ti]) AND (diversion*[ti] OR bypass*[ti])) OR (duodenal[ti] AND switch*[ti]) OR ((stomach[ti] OR gastric[ti]) AND (band*[ti] OR bypass*[ti] OR partiti*[ti] OR plicati*[ti] OR sleeve*[ti])) OR (sleeve[ti] AND gastrectom*[ti]) OR (roux[ti] AND y[ti])) AND ("Laparoscopy"[Mesh:NoExp] OR "Minimally Invasive Surgical Procedures"[Mesh:NoExp] OR laparoscop*[tiab] OR minimally invasive[tiab] OR minimal invasive[tiab] OR minimal access[tiab] OR minimal surg*[tiab] OR (minimal*[ti] AND invasive[ti]) OR (minimal[ti] AND (access[ti] OR surg*[ti])))) OR (lagb*[tiab] OR lrygb*[tiab] OR lap band*[tiab] OR lrygb*[tiab]) | [**13079**](https://www.ncbi.nlm.nih.gov/pubmed/?cmd=HistorySearch&querykey=25) |

## Embase.com (16 November 2018)

### T5Qa: Resleeve (12 SRs, 4 RCTs, 63 cohort studies)

| **No.** | **Query** | **Results** |
| --- | --- | --- |
| **#10** | #4 AND #9 NOT (#6 OR #8) | **63** |
| **#9** | 'epidemiology'/de OR (((cohort OR case) NEAR/3 (control OR controll* OR comparison OR referent)):ti,ab,kw) OR risk:ti,ab,kw OR causation:ti,ab,kw OR causal:ti,ab,kw OR 'odds ratio':ti,ab,kw OR etiol*:ti,ab,kw OR aetiol*:ti,ab,kw OR 'natural history':ti,ab,kw OR outcome:ti,ab,kw OR course:ti,ab,kw OR retrospect*:ti,ab,kw OR 'follow up':ti,ab,kw OR followup:ti,ab,kw OR predict*:ti,ab,kw OR prognos*:ti,ab,kw | **7321688** |
| **#8** | #4 AND #7 NOT #6 | **4** |
| **#7** | 'clinical':ti,ab,kw AND 'trial':ti,ab,kw OR 'clinical trial'/exp OR random*:ti,ab,kw | **2289070** |
| **#6** | #4 AND #5 | **12** |
| **#5** | 'meta-analysis':ti,ab,kw OR 'meta analysis'/exp OR 'review'/exp OR review:ti,ab,kw | **3434819** |
| **#4** | #3 NOT (('adolescent'/exp OR 'child'/exp OR adolescent*:ti,ab OR child*:ti,ab OR schoolchild*:ti,ab OR infant*:ti,ab OR girl*:ti,ab OR boy*:ti,ab OR teen:ti,ab OR teens:ti,ab OR teenager*:ti,ab OR youth*:ti,ab OR pediatr*:ti,ab OR paediatr*:ti,ab OR puber*:ti,ab) NOT ('adult'/exp OR 'aged'/exp OR 'middle aged'/exp OR adult*:ti,ab OR man:ti,ab OR men:ti,ab OR woman:ti,ab OR women:ti,ab)) | **117** |
| **#3** | #2 | **119** |
| **#2** | #1 NOT ([animals]/lim NOT [humans]/lim) AND [1-1-2004]/sd | **119** |
| **#1** | 're sleev*':ti,ab,kw OR resleev*:ti,ab,kw | **120** |

### T5Qb: RYGB (56 SRs, 14 RCTs, 93 cohort studies)

| **No.** | **Query** | **Results** |
| --- | --- | --- |
| **#13** | #10 AND #3 NOT (#11 OR #12) | **93** |
| **#12** | #10 AND #2 NOT #11 | **14** |
| **#11** | #1 AND #10 | **56** |
| **#10** | #9 NOT (('adolescent'/exp OR 'child'/exp OR adolescent*:ti,ab OR child*:ti,ab OR schoolchild*:ti,ab OR infant*:ti,ab OR girl*:ti,ab OR boy*:ti,ab OR teen:ti,ab OR teens:ti,ab OR teenager*:ti,ab OR youth*:ti,ab OR pediatr*:ti,ab OR paediatr*:ti,ab OR puber*:ti,ab) NOT ('adult'/exp OR 'aged'/exp OR 'middle aged'/exp OR adult*:ti,ab OR man:ti,ab OR men:ti,ab OR woman:ti,ab OR women:ti,ab)) | **211** |
| **#9** | #8 NOT ('conference abstract'/it OR 'conference paper'/it OR 'letter'/it OR 'note'/it) | **213** |
| **#8** | #7 NOT ([animals]/lim NOT [humans]/lim) AND [1-1-2004]/sd | **621** |
| **#7** | #4 AND #5 AND #6 | **637** |
| **#6** | 'reoperation'/exp OR revision*:ti,ab,kw OR revising*:ti,ab,kw OR redo*:ti,ab,kw OR repeat*:ti,ab,kw | **909545** |
| **#5** | 'body weight gain'/de OR ((weight NEAR/3 (gain* OR regain*)):ti,ab,kw) | **132031** |
| **#4** | ('roux y anastomosis'/exp OR ((roux NEAR/2 y):ti,ab,kw) OR rygb:ti,ab,kw) AND ('laparoscopy'/de OR 'laparoendoscopic single site surgery'/exp OR 'laparoscopic surgery'/exp OR 'minimally invasive procedure'/exp OR 'minimally invasive surgery'/exp OR laparoscop*:ti,ab,kw OR ((minimal*NEAR/3 invasive):ti,ab,kw) OR access:ti,ab,kw OR surg*:ti,ab,kw) OR lrygb*:ti,ab,kw | **18028** |
| **#3** | 'epidemiology'/de OR (((cohort OR case) NEAR/3 (control OR controll* OR comparison OR referent)):ti,ab,kw) OR risk:ti,ab,kw OR causation:ti,ab,kw OR causal:ti,ab,kw OR 'odds ratio':ti,ab,kw OR etiol*:ti,ab,kw OR aetiol*:ti,ab,kw OR 'natural history':ti,ab,kw OR outcome:ti,ab,kw OR course:ti,ab,kw OR retrospect*:ti,ab,kw OR 'follow up':ti,ab,kw OR followup:ti,ab,kw OR predict*:ti,ab,kw OR prognos*:ti,ab,kw | **7321688** |
| **#2** | 'clinical':ti,ab,kw AND 'trial':ti,ab,kw OR 'clinical trial'/exp OR random*:ti,ab,kw | **2289070** |
| **#1** | 'meta-analysis':ti,ab,kw OR 'meta analysis'/exp OR 'review'/exp OR review:ti,ab,kw | **3434819** |

### T5Qc: BPD/DS or SADI-S (44 SRs, 6 RCTs, 109 cohort studies)

| **No.** | **Query** | **Results** |
| --- | --- | --- |
| **#12** | #9 AND #3 NOT (#10 OR #11) | **109** |
| **#11** | #9 AND #2 NOT #10 | **6** |
| **#10** | #1 AND #9 | **44** |
| **#9** | #8 NOT (('adolescent'/exp OR 'child'/exp OR adolescent*:ti,ab OR child*:ti,ab OR schoolchild*:ti,ab OR infant*:ti,ab OR girl*:ti,ab OR boy*:ti,ab OR teen:ti,ab OR teens:ti,ab OR teenager*:ti,ab OR youth*:ti,ab OR pediatr*:ti,ab OR paediatr*:ti,ab OR puber*:ti,ab) NOT ('adult'/exp OR 'aged'/exp OR 'middle aged'/exp OR adult*:ti,ab OR man:ti,ab OR men:ti,ab OR woman:ti,ab OR women:ti,ab)) | **211** |
| **#8** | #7 NOT ('conference abstract'/it OR 'conference paper'/it OR 'letter'/it OR 'note'/it) | **211** |
| **#7** | #6 NOT ([animals]/lim NOT [humans]/lim) AND [1-1-2004]/sd | **446** |
| **#6** | #4 AND #5 | **448** |
| **#5** | 'reoperation'/exp/mj OR revision*:ti,kw OR revising*:ti,kw OR redo*:ti,kw OR repeat*:ti,kw | **134485** |
| **#4** | ('biliopancreatic bypass'/exp/mj OR ((('bilio pancreatic' OR biliopancreatic) NEAR/3 (diversion* OR bypass*)):ti,kw) OR ((duodenal NEAR/3 switch*):ti,kw) OR (((stomach OR gastric) NEAR/3 bypass*):ti,kw) OR sadis:ti,kw OR 'sadi s':ti,kw) AND ('laparoscopy'/de OR 'laparoendoscopic single site surgery'/exp OR 'laparoscopic surgery'/exp OR 'minimally invasive procedure'/exp OR 'minimally invasive surgery'/exp OR laparoscop*:ti,ab,kw OR ((minimal* NEAR/3 (invasive OR access OR surg*)):ti,ab,kw)) | **5670** |
| **#3** | 'epidemiology'/de OR (((cohort OR case) NEAR/3 (control OR controll* OR comparison OR referent)):ti,ab,kw) OR risk:ti,ab,kw OR causation:ti,ab,kw OR causal:ti,ab,kw OR 'odds ratio':ti,ab,kw OR etiol*:ti,ab,kw OR aetiol*:ti,ab,kw OR 'natural history':ti,ab,kw OR outcome:ti,ab,kw OR course:ti,ab,kw OR retrospect*:ti,ab,kw OR 'follow up':ti,ab,kw OR followup:ti,ab,kw OR predict*:ti,ab,kw OR prognos*:ti,ab,kw | **7321688** |
| **#2** | 'clinical':ti,ab,kw AND 'trial':ti,ab,kw OR 'clinical trial'/exp OR random*:ti,ab,kw | **2289070** |
| **#1** | 'meta-analysis':ti,ab,kw OR 'meta analysis'/exp OR 'review'/exp OR review:ti,ab,kw | **3434819** |

### T5Qd: Sleeve gastrectomy (17 SRs, 4 RCTs, 54 cohort studies)

| **No.** | **Query** | **Results** |
| --- | --- | --- |
| **#13** | #10 AND #3 NOT (#11 OR #12) | **54** |
| **#12** | #10 AND #2 NOT #11 | **4** |
| **#11** | #1 AND #10 | **17** |
| **#10** | #9 NOT (('adolescent'/exp OR 'child'/exp OR adolescent*:ti,ab OR child*:ti,ab OR schoolchild*:ti,ab OR infant*:ti,ab OR girl*:ti,ab OR boy*:ti,ab OR teen:ti,ab OR teens:ti,ab OR teenager*:ti,ab OR youth*:ti,ab OR pediatr*:ti,ab OR paediatr*:ti,ab OR puber*:ti,ab) NOT ('adult'/exp OR 'aged'/exp OR 'middle aged'/exp OR adult*:ti,ab OR man:ti,ab OR men:ti,ab OR woman:ti,ab OR women:ti,ab)) | **95** |
| **#9** | #8 NOT ('conference abstract'/it OR 'conference paper'/it OR 'letter'/it OR 'note'/it) | **96** |
| **#8** | #7 NOT ([animals]/lim NOT [humans]/lim) AND [1-1-2004]/sd | **301** |
| **#7** | #4 AND #5 AND #6 | **303** |
| **#6** | 'reoperation'/exp OR revision*:ti,ab,kw OR revising*:ti,ab,kw OR redo*:ti,ab,kw OR repeat*:ti,ab,kw | **909545** |
| **#5** | 'body weight gain'/de OR ((weight NEAR/3 (gain* OR regain*)):ti,ab,kw) | **132031** |
| **#4** | ('gastric sleeve'/exp OR (((stomach OR gastric OR gastrectom*) NEAR/3 sleeve*):ti,ab,kw)) AND ('laparoscopy'/de OR 'laparoendoscopic single site surgery'/exp OR 'laparoscopic surgery'/exp OR 'minimally invasive procedure'/exp OR 'minimally invasive surgery'/exp OR laparoscop*:ti,ab,kw OR ((minimal* NEAR/3 (invasive OR access OR surg*)):ti,ab,kw)) OR 'laparoscopic sleeve gastrectomy'/exp | **7276** |
| **#3** | 'epidemiology'/de OR (((cohort OR case) NEAR/3 (control OR controll* OR comparison OR referent)):ti,ab,kw) OR risk:ti,ab,kw OR causation:ti,ab,kw OR causal:ti,ab,kw OR 'odds ratio':ti,ab,kw OR etiol*:ti,ab,kw OR aetiol*:ti,ab,kw OR 'natural history':ti,ab,kw OR outcome:ti,ab,kw OR course:ti,ab,kw OR retrospect*:ti,ab,kw OR 'follow up':ti,ab,kw OR followup:ti,ab,kw OR predict*:ti,ab,kw OR prognos*:ti,ab,kw | **7321688** |
| **#2** | 'clinical':ti,ab,kw AND 'trial':ti,ab,kw OR 'clinical trial'/exp OR random*:ti,ab,kw | **2289070** |
| **#1** | 'meta-analysis':ti,ab,kw OR 'meta analysis'/exp OR 'review'/exp OR review:ti,ab,kw | **3434819** |

### T5Qe: Limb lengthening (10 all study types)

| **No.** | **Query** | **Results** |
| --- | --- | --- |
| **#9** | #8 NOT (('adolescent'/exp OR 'child'/exp OR adolescent*:ti,ab OR child*:ti,ab OR schoolchild*:ti,ab OR infant*:ti,ab OR girl*:ti,ab OR boy*:ti,ab OR teen:ti,ab OR teens:ti,ab OR teenager*:ti,ab OR youth*:ti,ab OR pediatr*:ti,ab OR paediatr*:ti,ab OR puber*:ti,ab) NOT ('adult'/exp OR 'aged'/exp OR 'middle aged'/exp OR adult*:ti,ab OR man:ti,ab OR men:ti,ab OR woman:ti,ab OR women:ti,ab)) | **10** |
| **#8** | #7 NOT ('conference abstract'/it OR 'conference paper'/it OR 'letter'/it OR 'note'/it) | **10** |
| **#7** | #6 NOT ([animals]/lim NOT [humans]/lim) AND [1-1-2004]/sd | **32** |
| **#6** | #4 AND #5 | **32** |
| **#5** | ('obesity'/exp/mj AND 'surgery'/lnk OR 'bariatric surgery'/exp/mj OR 'roux y anastomosis'/exp/mj OR 'gastric bypass surgery'/exp/mj OR ((('bilio pancreatic' OR biliopancreatic) NEAR/3 (diversion* OR bypass*)):ti,kw) OR ((duodenal NEAR/3 switch*):ti,kw) OR (((stomach OR gastric) NEAR/3 (band* OR bypass* OR partiti* OR plicati* OR sleeve*)):ti,kw) OR ((sleeve NEAR/3 gastrectom*):ti,kw) OR ((roux NEAR/2 y):ti,kw) OR gastroplast*:ti,kw OR mgbp*:ti,kw OR oagb*:ti,kw OR rya:ti,kw OR rygb*:ti,kw OR sadis:ti,kw OR 'sadi s':ti,kw OR (((bariatric OR obes* OR 'weight loss' OR metabolic) NEAR/3 (surg* OR operati* OR procedure*)):ti,kw) OR sagb*:ti,kw) AND ('laparoscopy'/de OR 'laparoendoscopic single site surgery'/exp OR 'laparoscopic surgery'/exp OR 'minimally invasive procedure'/exp OR 'minimally invasive surgery'/exp OR laparoscop*:ti,ab,kw OR ((minimal* NEAR/3 invasive):ti,ab,kw) OR access:ti,ab,kw OR surg*:ti,ab,kw) OR 'laparoscopic sleeve gastrectomy'/exp OR lagb*:ti,ab,kw OR 'lap band*':ti,ab,kw OR lrygb*:ti,ab,kw | **40615** |
| **#4** | (limb* NEAR/3 lengthen*):ti,ab,kw | **1255** |
| **#3** | 'epidemiology'/de OR (((cohort OR case) NEAR/3 (control OR controll* OR comparison OR referent)):ti,ab,kw) OR risk:ti,ab,kw OR causation:ti,ab,kw OR causal:ti,ab,kw OR 'odds ratio':ti,ab,kw OR etiol*:ti,ab,kw OR aetiol*:ti,ab,kw OR 'natural history':ti,ab,kw OR outcome:ti,ab,kw OR course:ti,ab,kw OR retrospect*:ti,ab,kw OR 'follow up':ti,ab,kw OR followup:ti,ab,kw OR predict*:ti,ab,kw OR prognos*:ti,ab,kw | **7321688** |
| **#2** | 'clinical':ti,ab,kw AND 'trial':ti,ab,kw OR 'clinical trial'/exp OR random*:ti,ab,kw | **2289070** |
| **#1** | 'meta-analysis':ti,ab,kw OR 'meta analysis'/exp OR 'review'/exp OR review:ti,ab,kw | **3434819** |

### T5Qf: Gastric bypass banding (30 SRs, 3 RCTs, 54 cohort studies)

| **No.** | **Query** | **Results** |
| --- | --- | --- |
| **#13** | #10 AND #3 NOT (#11 OR #12) | **54** |
| **#12** | #10 AND #2 NOT #11 | **3** |
| **#11** | #1 AND #10 | **30** |
| **#10** | #9 NOT (('adolescent'/exp OR 'child'/exp OR adolescent*:ti,ab OR child*:ti,ab OR schoolchild*:ti,ab OR infant*:ti,ab OR girl*:ti,ab OR boy*:ti,ab OR teen:ti,ab OR teens:ti,ab OR teenager*:ti,ab OR youth*:ti,ab OR pediatr*:ti,ab OR paediatr*:ti,ab OR puber*:ti,ab) NOT ('adult'/exp OR 'aged'/exp OR 'middle aged'/exp OR adult*:ti,ab OR man:ti,ab OR men:ti,ab OR woman:ti,ab OR women:ti,ab)) | **113** |
| **#9** | #8 NOT ('conference abstract'/it OR 'conference paper'/it OR 'letter'/it OR 'note'/it) | **113** |
| **#8** | #7 NOT ([animals]/lim NOT [humans]/lim) AND [1-1-2004]/sd | **294** |
| **#7** | #4 AND #5 AND #6 | **297** |
| **#6** | 'reoperation'/exp/mj OR revision*:ti,kw OR revising*:ti,kw OR redo*:ti,kw OR repeat*:ti,kw | **134485** |
| **#5** | 'body weight gain'/de OR ((weight NEAR/3 (gain* OR regain*)):ti,ab,kw) | **132031** |
| **#4** | ('gastric bypass surgery'/exp OR 'gastric banding'/exp OR (((stomach OR gastric) NEAR/3 (band* OR bypass*)):ti,ab,kw) OR mgbp*:ti,ab,kw OR oagb*:ti,ab,kw) AND ('laparoscopy'/de OR 'laparoendoscopic single site surgery'/exp OR 'laparoscopic surgery'/exp OR 'minimally invasive procedure'/exp OR 'minimally invasive surgery'/exp OR laparoscop*:ti,ab,kw OR ((minimal* NEAR/3 (invasive OR access OR surg*)):ti,ab,kw)) | **12510** |
| **#3** | 'epidemiology'/de OR (((cohort OR case) NEAR/3 (control OR controll* OR comparison OR referent)):ti,ab,kw) OR risk:ti,ab,kw OR causation:ti,ab,kw OR causal:ti,ab,kw OR 'odds ratio':ti,ab,kw OR etiol*:ti,ab,kw OR aetiol*:ti,ab,kw OR 'natural history':ti,ab,kw OR outcome:ti,ab,kw OR course:ti,ab,kw OR retrospect*:ti,ab,kw OR 'follow up':ti,ab,kw OR followup:ti,ab,kw OR predict*:ti,ab,kw OR prognos*:ti,ab,kw | **7321688** |
| **#2** | 'clinical':ti,ab,kw AND 'trial':ti,ab,kw OR 'clinical trial'/exp OR random*:ti,ab,kw | **2289070** |
| **#1** | 'meta-analysis':ti,ab,kw OR 'meta analysis'/exp OR 'review'/exp OR review:ti,ab,kw | **3434819** |

### T5Qg: Pouch resizing (6 SRs, 6 RCTs, 27 cohort studies)

| **No.** | **Query** | **Results** |
| --- | --- | --- |
| **#12** | #9 AND #3 NOT (#10 OR #11) | **27** |
| **#11** | #9 AND #2 NOT #10 | **6** |
| **#10** | #1 AND #9 | **6** |
| **#9** | #8 NOT (('adolescent'/exp OR 'child'/exp OR adolescent*:ti,ab OR child*:ti,ab OR schoolchild*:ti,ab OR infant*:ti,ab OR girl*:ti,ab OR boy*:ti,ab OR teen:ti,ab OR teens:ti,ab OR teenager*:ti,ab OR youth*:ti,ab OR pediatr*:ti,ab OR paediatr*:ti,ab OR puber*:ti,ab) NOT ('adult'/exp OR 'aged'/exp OR 'middle aged'/exp OR adult*:ti,ab OR man:ti,ab OR men:ti,ab OR woman:ti,ab OR women:ti,ab)) | **57** |
| **#8** | #7 NOT ('conference abstract'/it OR 'conference paper'/it OR 'letter'/it OR 'note'/it) | **57** |
| **#7** | #6 NOT ([animals]/lim NOT [humans]/lim) AND [1-1-2004]/sd | **87** |
| **#6** | #4 AND #5 | **96** |
| **#5** | ('stomach pouch'/exp/mj OR pouch*:ti,kw) AND (resiz*:ti,kw OR upsiz*:ti,kw OR downsiz*:ti,kw OR size:ti,kw OR sizing*:ti,kw OR sizes:ti,kw OR reshap*:ti,kw OR shape*:ti,kw OR shaping*:ti,kw OR formation*:ti,kw OR reconstruct*:ti,kw OR revision*:ti,kw OR revising*:ti,kw OR redo*:ti,kw OR repeat*:ti,kw) OR (pouch*:ti,kw AND 'reoperation'/exp/mj) | **618** |
| **#4** | ('obesity'/exp/mj AND 'surgery'/lnk OR 'bariatric surgery'/exp/mj OR 'roux y anastomosis'/exp/mj OR 'gastric bypass surgery'/exp/mj OR ((('bilio pancreatic' OR biliopancreatic) NEAR/3 (diversion* OR bypass*)):ti,kw) OR ((duodenal NEAR/3 switch*):ti,kw) OR (((stomach OR gastric) NEAR/3 (band* OR bypass* OR partiti* OR plicati* OR sleeve*)):ti,kw) OR ((sleeve NEAR/3 gastrectom*):ti,kw) OR ((roux NEAR/2 y):ti,kw) OR gastroplast*:ti,kw OR mgbp*:ti,kw OR oagb*:ti,kw OR rya:ti,kw OR rygb*:ti,kw OR sadis:ti,kw OR 'sadi s':ti,kw OR (((bariatric OR obes* OR 'weight loss' OR metabolic) NEAR/3 (surg* OR operati* OR procedure*)):ti,kw) OR sagb*:ti,kw) AND ('laparoscopy'/de OR 'laparoendoscopic single site surgery'/exp OR 'laparoscopic surgery'/exp OR 'minimally invasive procedure'/exp OR 'minimally invasive surgery'/exp OR laparoscop*:ti,ab,kw OR ((minimal* NEAR/3 invasive):ti,ab,kw) OR access:ti,ab,kw OR surg*:ti,ab,kw) OR 'laparoscopic sleeve gastrectomy'/exp OR lagb*:ti,ab,kw OR 'lap band*':ti,ab,kw OR lrygb*:ti,ab,kw | **40615** |
| **#3** | 'epidemiology'/de OR (((cohort OR case) NEAR/3 (control OR controll* OR comparison OR referent)):ti,ab,kw) OR risk:ti,ab,kw OR causation:ti,ab,kw OR causal:ti,ab,kw OR 'odds ratio':ti,ab,kw OR etiol*:ti,ab,kw OR aetiol*:ti,ab,kw OR 'natural history':ti,ab,kw OR outcome:ti,ab,kw OR course:ti,ab,kw OR retrospect*:ti,ab,kw OR 'follow up':ti,ab,kw OR followup:ti,ab,kw OR predict*:ti,ab,kw OR prognos*:ti,ab,kw | **7321688** |
| **#2** | 'clinical':ti,ab,kw AND 'trial':ti,ab,kw OR 'clinical trial'/exp OR random*:ti,ab,kw | **2289070** |
| **#1** | 'meta-analysis':ti,ab,kw OR 'meta analysis'/exp OR 'review'/exp OR review:ti,ab,kw | **3434819** |

### T5Qh: Alimentary limb (32 SRs, 17 RCTs, 92 cohort studies)

| **No.** | **Query** | **Results** |
| --- | --- | --- |
| **#12** | #9 AND #3 NOT (#10 OR #11) | **92** |
| **#11** | #9 AND #2 NOT #10 | **17** |
| **#10** | #1 AND #9 | **32** |
| **#9** | #8 NOT (('adolescent'/exp OR 'child'/exp OR adolescent*:ti,ab OR child*:ti,ab OR schoolchild*:ti,ab OR infant*:ti,ab OR girl*:ti,ab OR boy*:ti,ab OR teen:ti,ab OR teens:ti,ab OR teenager*:ti,ab OR youth*:ti,ab OR pediatr*:ti,ab OR paediatr*:ti,ab OR puber*:ti,ab) NOT ('adult'/exp OR 'aged'/exp OR 'middle aged'/exp OR adult*:ti,ab OR man:ti,ab OR men:ti,ab OR woman:ti,ab OR women:ti,ab)) | **203** |
| **#8** | #7 NOT ('conference abstract'/it OR 'conference paper'/it OR 'letter'/it OR 'note'/it) | **203** |
| **#7** | #6 NOT ([animals]/lim NOT [humans]/lim) AND [1-1-2004]/sd | **552** |
| **#6** | #4 AND #5 | **612** |
| **#5** | ('obesity'/exp/mj AND 'surgery'/lnk OR 'bariatric surgery'/exp/mj OR 'roux y anastomosis'/exp/mj OR 'gastric bypass surgery'/exp/mj OR ((('bilio pancreatic' OR biliopancreatic) NEAR/3 (diversion* OR bypass*)):ti,kw) OR ((duodenal NEAR/3 switch*):ti,kw) OR (((stomach OR gastric) NEAR/3 (band* OR bypass* OR partiti* OR plicati* OR sleeve*)):ti,kw) OR ((sleeve NEAR/3 gastrectom*):ti,kw) OR ((roux NEAR/2 y):ti,kw) OR gastroplast*:ti,kw OR mgbp*:ti,kw OR oagb*:ti,kw OR rya:ti,kw OR rygb*:ti,kw OR sadis:ti,kw OR 'sadi s':ti,kw OR (((bariatric OR obes* OR 'weight loss' OR metabolic) NEAR/3 (surg* OR operati* OR procedure*)):ti,kw) OR sagb*:ti,kw) AND ('laparoscopy'/de OR 'laparoendoscopic single site surgery'/exp OR 'laparoscopic surgery'/exp OR 'minimally invasive procedure'/exp OR 'minimally invasive surgery'/exp OR laparoscop*:ti,ab,kw OR ((minimal* NEAR/3 invasive):ti,ab,kw) OR access:ti,ab,kw OR surg*:ti,ab,kw) OR 'laparoscopic sleeve gastrectomy'/exp OR lagb*:ti,ab,kw OR 'lap band*':ti,ab,kw OR lrygb*:ti,ab,kw | **40615** |
| **#4** | 'alimentary limb*':ti,ab,kw OR 'biliopancreatic limb*':ti,ab,kw OR 'bilio pancreatic limb*':ti,ab,kw OR 'long limb*':ti,ab,kw OR 'short limb*':ti,ab,kw OR 'standard limb*':ti,ab,kw | **2102** |
| **#3** | 'epidemiology'/de OR (((cohort OR case) NEAR/3 (control OR controll* OR comparison OR referent)):ti,ab,kw) OR risk:ti,ab,kw OR causation:ti,ab,kw OR causal:ti,ab,kw OR 'odds ratio':ti,ab,kw OR etiol*:ti,ab,kw OR aetiol*:ti,ab,kw OR 'natural history':ti,ab,kw OR outcome:ti,ab,kw OR course:ti,ab,kw OR retrospect*:ti,ab,kw OR 'follow up':ti,ab,kw OR followup:ti,ab,kw OR predict*:ti,ab,kw OR prognos*:ti,ab,kw | **7321688** |
| **#2** | 'clinical':ti,ab,kw AND 'trial':ti,ab,kw OR 'clinical trial'/exp OR random*:ti,ab,kw | **2289070** |
| **#1** | 'meta-analysis':ti,ab,kw OR 'meta analysis'/exp OR 'review'/exp OR review:ti,ab,kw | **3434819** |

### T5Qi: Watchful waiting (2 all study types)

| **No.** | **Query** | **Results** |
| --- | --- | --- |
| **#7** | #4 AND #5 AND #6 | **2** |
| **#6** | ('obesity'/exp/mj AND 'surgery'/lnk OR 'bariatric surgery'/exp/mj OR 'roux y anastomosis'/exp/mj OR 'gastric bypass surgery'/exp/mj OR ((('bilio pancreatic' OR biliopancreatic) NEAR/3 (diversion* OR bypass*)):ti,kw) OR ((duodenal NEAR/3 switch*):ti,kw) OR (((stomach OR gastric) NEAR/3 (band* OR bypass* OR partiti* OR plicati* OR sleeve*)):ti,kw) OR ((sleeve NEAR/3 gastrectom*):ti,kw) OR ((roux NEAR/2 y):ti,kw) OR gastroplast*:ti,kw OR mgbp*:ti,kw OR oagb*:ti,kw OR rya:ti,kw OR rygb*:ti,kw OR sadis:ti,kw OR 'sadi s':ti,kw OR (((bariatric OR obes* OR 'weight loss' OR metabolic) NEAR/3 (surg* OR operati* OR procedure*)):ti,kw) OR sagb*:ti,kw) AND ('laparoscopy'/de OR 'laparoendoscopic single site surgery'/exp OR 'laparoscopic surgery'/exp OR 'minimally invasive procedure'/exp OR 'minimally invasive surgery'/exp OR laparoscop*:ti,ab,kw OR ((minimal* NEAR/3 invasive):ti,ab,kw) OR access:ti,ab,kw OR surg*:ti,ab,kw) OR 'laparoscopic sleeve gastrectomy'/exp OR lagb*:ti,ab,kw OR 'lap band*':ti,ab,kw OR lrygb*:ti,ab,kw | **40615** |
| **#5** | 'reoperation'/exp OR revision*:ti,ab,kw OR revising*:ti,ab,kw OR redo*:ti,ab,kw OR repeat*:ti,ab,kw | **909545** |
| **#4** | 'watchful waiting'/exp OR 'watchful wait*':ti,ab,kw OR 'watch and wait':ti,ab,kw OR 'wait and watch':ti,ab,kw | **6804** |
| **#3** | 'epidemiology'/de OR (((cohort OR case) NEAR/3 (control OR controll* OR comparison OR referent)):ti,ab,kw) OR risk:ti,ab,kw OR causation:ti,ab,kw OR causal:ti,ab,kw OR 'odds ratio':ti,ab,kw OR etiol*:ti,ab,kw OR aetiol*:ti,ab,kw OR 'natural history':ti,ab,kw OR outcome:ti,ab,kw OR course:ti,ab,kw OR retrospect*:ti,ab,kw OR 'follow up':ti,ab,kw OR followup:ti,ab,kw OR predict*:ti,ab,kw OR prognos*:ti,ab,kw | **7321688** |
| **#2** | 'clinical':ti,ab,kw AND 'trial':ti,ab,kw OR 'clinical trial'/exp OR random*:ti,ab,kw | **2289070** |
| **#1** | 'meta-analysis':ti,ab,kw OR 'meta analysis'/exp OR 'review'/exp OR review:ti,ab,kw | **3434819** |

### T5Qj: Hiatal hernia repair (48 SRs, 10 RCTs, 84 cohort studies)

| **No.** | **Query** | **Results** |
| --- | --- | --- |
| **#13** | #10 AND #3 NOT (#11 OR #12) | **84** |
| **#12** | #10 AND #2 NOT #11 | **10** |
| **#11** | #1 AND #10 | **48** |
| **#10** | #9 NOT (('adolescent'/exp OR 'child'/exp OR adolescent*:ti,ab OR child*:ti,ab OR schoolchild*:ti,ab OR infant*:ti,ab OR girl*:ti,ab OR boy*:ti,ab OR teen:ti,ab OR teens:ti,ab OR teenager*:ti,ab OR youth*:ti,ab OR pediatr*:ti,ab OR paediatr*:ti,ab OR puber*:ti,ab) NOT ('adult'/exp OR 'aged'/exp OR 'middle aged'/exp OR adult*:ti,ab OR man:ti,ab OR men:ti,ab OR woman:ti,ab OR women:ti,ab)) | **184** |
| **#9** | #8 NOT ('conference abstract'/it OR 'conference paper'/it OR 'letter'/it OR 'note'/it) | **186** |
| **#8** | #7 NOT ([animals]/lim NOT [humans]/lim) AND [1-1-2004]/sd | **585** |
| **#7** | #4 AND #5 AND #6 | **602** |
| **#6** | ('obesity'/exp/mj AND 'surgery'/lnk OR 'bariatric surgery'/exp/mj OR 'roux y anastomosis'/exp/mj OR 'gastric bypass surgery'/exp/mj OR ((('bilio pancreatic' OR biliopancreatic) NEAR/3 (diversion* OR bypass*)):ti,kw) OR ((duodenal NEAR/3 switch*):ti,kw) OR (((stomach OR gastric) NEAR/3 (band* OR bypass* OR partiti* OR plicati* OR sleeve*)):ti,kw) OR ((sleeve NEAR/3 gastrectom*):ti,kw) OR ((roux NEAR/2 y):ti,kw) OR gastroplast*:ti,kw OR mgbp*:ti,kw OR oagb*:ti,kw OR rya:ti,kw OR rygb*:ti,kw OR sadis:ti,kw OR 'sadi s':ti,kw OR (((bariatric OR obes* OR 'weight loss' OR metabolic) NEAR/3 (surg* OR operati* OR procedure*)):ti,kw) OR sagb*:ti,kw) AND ('laparoscopy'/de OR 'laparoendoscopic single site surgery'/exp OR 'laparoscopic surgery'/exp OR 'minimally invasive procedure'/exp OR 'minimally invasive surgery'/exp OR laparoscop*:ti,ab,kw OR ((minimal* NEAR/3 invasive):ti,ab,kw) OR access:ti,ab,kw OR surg*:ti,ab,kw) OR 'laparoscopic sleeve gastrectomy'/exp OR lagb*:ti,ab,kw OR 'lap band*':ti,ab,kw OR lrygb*:ti,ab,kw | **40615** |
| **#5** | 'reoperation'/exp OR revision*:ti,ab,kw OR revising*:ti,ab,kw OR redo*:ti,ab,kw OR repeat*:ti,ab,kw OR repair*:ti,ab,kw | **1282180** |
| **#4** | 'hiatus hernia'/exp OR 'hiatal hernia*':ti,ab,kw OR 'hiatus hernia*':ti,ab,kw OR 'esophageal hernia*':ti,ab,kw OR 'paraesophageal hernia*':ti,ab,kw OR 'oesophageal hernia*':ti,ab,kw OR 'paraoesophageal hernia*':ti,ab,kw | **13933** |
| **#3** | 'epidemiology'/de OR (((cohort OR case) NEAR/3 (control OR controll* OR comparison OR referent)):ti,ab,kw) OR risk:ti,ab,kw OR causation:ti,ab,kw OR causal:ti,ab,kw OR 'odds ratio':ti,ab,kw OR etiol*:ti,ab,kw OR aetiol*:ti,ab,kw OR 'natural history':ti,ab,kw OR outcome:ti,ab,kw OR course:ti,ab,kw OR retrospect*:ti,ab,kw OR 'follow up':ti,ab,kw OR followup:ti,ab,kw OR predict*:ti,ab,kw OR prognos*:ti,ab,kw | **7321688** |
| **#2** | 'clinical':ti,ab,kw AND 'trial':ti,ab,kw OR 'clinical trial'/exp OR random*:ti,ab,kw | **2289070** |
| **#1** | 'meta-analysis':ti,ab,kw OR 'meta analysis'/exp OR 'review'/exp OR review:ti,ab,kw | **3434819** |

# Topic 6: Postoperative Care

## PubMed (23 November 2018 + 11 February + 20 February 2019)

### T6Q1: Nutrients (36 SRs, 60 RCTs, 81 cohort studies)

| **Search** | **Query** | **Items found** |
| --- | --- | --- |
| [**#40**](https://www.ncbi.nlm.nih.gov/pubmed) | ((#34 AND #39) NOT (#36 OR #38)) | [**81**](https://www.ncbi.nlm.nih.gov/pubmed/?cmd=HistorySearch&querykey=40) |
| [**#39**](https://www.ncbi.nlm.nih.gov/pubmed) | ("Epidemiologic Studies"[Mesh] OR "Prognosis"[Mesh] OR cohort[tiab] OR (case[tiab] AND (control[tiab] OR controll*[tiab] OR comparison[tiab] OR referent[tiab])) OR risk[tiab] OR causation[tiab] OR causal[tiab] OR "odds ratio"[tiab] OR etiol*[tiab] OR aetiol*[tiab] OR "natural history"[tiab] OR predict*[tiab] OR outcome[tiab] OR course[tiab] OR retrospect*[tiab] OR “follow up”[tiab] OR followup[tiab] OR prognos*[tiab]) | [**6612503**](https://www.ncbi.nlm.nih.gov/pubmed/?cmd=HistorySearch&querykey=39) |
| [**#38**](https://www.ncbi.nlm.nih.gov/pubmed) | (#34 AND #37 NOT #36) | [**60**](https://www.ncbi.nlm.nih.gov/pubmed/?cmd=HistorySearch&querykey=38) |
| [**#37**](https://www.ncbi.nlm.nih.gov/pubmed) | ("Randomized Controlled Trial"[Publication Type] OR "Controlled Clinical Trial"[Publication Type] OR random*[tiab] OR placebo[tiab] OR "Drug Therapy"[Subheading] OR trial[tiab] OR groups[tiab]) | [**4576640**](https://www.ncbi.nlm.nih.gov/pubmed/?cmd=HistorySearch&querykey=37) |
| [**#36**](https://www.ncbi.nlm.nih.gov/pubmed) | (#34 AND #35) | [**36**](https://www.ncbi.nlm.nih.gov/pubmed/?cmd=HistorySearch&querykey=36) |
| [**#35**](https://www.ncbi.nlm.nih.gov/pubmed) | ((review[tiab] OR "Review"[Publication Type] OR "Meta-Analysis as Topic"[Mesh] OR meta-analysis[tiab] OR "Meta-Analysis"[Publication Type]) NOT ("Letter"[Publication Type] OR "Editorial"[Publication Type] OR "Comment"[Publication Type])) | [**2998395**](https://www.ncbi.nlm.nih.gov/pubmed/?cmd=HistorySearch&querykey=35) |
| [**#34**](https://www.ncbi.nlm.nih.gov/pubmed) | (#33 NOT (("Adolescent"[Mesh] OR "Child"[Mesh] OR "Infant"[Mesh] OR adolescen*[tiab] OR child*[tiab] OR schoolchild*[tiab] OR infant*[tiab] OR girl*[tiab] OR boy*[tiab] OR teen[tiab] OR teens[tiab] OR teenager*[tiab] OR youth*[tiab] OR pediatr*[tiab] OR paediatr*[tiab] OR puber*[tiab]) NOT ("Adult"[Mesh] OR adult*[tiab] OR man[tiab] OR men[tiab] OR woman[tiab] OR women[tiab]))) | [**202**](https://www.ncbi.nlm.nih.gov/pubmed/?cmd=HistorySearch&querykey=34) |
| [**#33**](https://www.ncbi.nlm.nih.gov/pubmed) | (#32 NOT ("Animals"[Mesh] NOT "Humans"[Mesh]) AND ("2004"[Date - Entrez] : "3000"[Date - Entrez])) | [**211**](https://www.ncbi.nlm.nih.gov/pubmed/?cmd=HistorySearch&querykey=33) |
| [**#32**](https://www.ncbi.nlm.nih.gov/pubmed) | (#30 AND #31) | [**220**](https://www.ncbi.nlm.nih.gov/pubmed/?cmd=HistorySearch&querykey=32) |
| [**#31**](https://www.ncbi.nlm.nih.gov/pubmed) | ("Micronutrients"[Mesh] OR "Dietary Supplements"[Mesh] OR "Dietary Fiber"[Mesh] OR "Dietary Proteins"[Mesh] OR micronutrient*[tiab] OR macronutrient*[tiab] OR micro nutrient*[tiab] OR macro nutrient*[tiab] OR vitamin*[tiab] OR provitamin*[tiab] OR trace element*[tiab] OR biometal*[tiab] OR prebiotic*[tiab] OR probiotic*[tiab] OR synbiotic*[tiab] OR dietary protein*[tiab] OR protein supplement*[tiab] OR dietary supplement*[tiab] OR food supplement*[tiab] OR nutraceutical*[tiab] OR nutriceutical*[tiab] OR neutraceutical*[tiab] OR dietary fiber*[tiab] OR wheat bran*[tiab] OR roughage*[tiab]) | [**426840**](https://www.ncbi.nlm.nih.gov/pubmed/?cmd=HistorySearch&querykey=31) |
| [**#30**](https://www.ncbi.nlm.nih.gov/pubmed) | ((("Bariatric Surgery"[Mesh] OR "Obesity/surgery"[Mesh:NoExp] OR "Obesity, Abdominal/surgery"[Mesh] OR "Obesity, Metabolically Benign/surgery"[Mesh] OR "Obesity, Morbid/surgery"[Mesh] OR "Anastomosis, Roux-en-Y"[Mesh] OR "Biliopancreatic Diversion"[Mesh] OR "Overweight/surgery"[Mesh] OR "Gastrectomy"[Mesh] OR bariatric surg*[tiab] OR bariatric operati*[tiab] OR bariatric procedure*[tiab] OR bilio pancreatic bypass*[tiab] OR bilio pancreatic diversion*[tiab] OR biliopancreatic bypass*[tiab] OR biliopancreatic diversion*[tiab] OR duodenal switch*[tiab] OR gastric band*[tiab] OR stomach band*[tiab] OR gastric bypass*[tiab] OR gastric partiti*[tiab] OR gastric plicati*[tiab] OR gastroplast*[tiab] OR mgbp*[tiab] OR oagb*[tiab] OR obese surg*[tiab] OR weight loss surg*[tiab] OR obesity surg*[tiab] OR metabolic surg*[tiab] OR roux en y*[tiab] OR “roux y”[tiab] OR “roux n y”[tiab] OR “roux in y”[tiab] OR rya[tiab] OR rygb*[tiab] OR sadis[tiab] OR “sadi s”[tiab] OR sagb*[tiab] OR sleeve gastrectom*[tiab] OR gastric sleeve*[tiab] OR gastrectom*[tiab] OR ((bariatric[ti] OR obes*[ti] OR weight loss[ti] OR metabolic[ti]) AND (surg*[ti] OR operati*[ti] OR procedure*[ti])) OR ((biliopancreatic[ti] OR “bilio pancreatic”[ti]) AND (diversion*[ti] OR bypass*[ti])) OR (duodenal[ti] AND switch*[ti]) OR ((stomach[ti] OR gastric[ti]) AND (band*[ti] OR bypass*[ti] OR partiti*[ti] OR plicati*[ti] OR sleeve*[ti])) OR (sleeve[ti] AND gastrectom*[ti]) OR (roux[ti] AND y[ti])) AND ("Laparoscopy"[Mesh:NoExp] OR "Minimally Invasive Surgical Procedures"[Mesh:NoExp] OR laparoscop*[tiab] OR minimally invasive[tiab] OR minimal invasive[tiab] OR minimal access[tiab] OR minimal surg*[tiab] OR (minimal*[ti] AND invasive[ti]) OR (minimal[ti] AND (access[ti] OR surg*[ti])))) OR (lagb*[tiab] OR lrygb*[tiab] OR lap band*[tiab] OR lrygb*[tiab])) | [**13120**](https://www.ncbi.nlm.nih.gov/pubmed/?cmd=HistorySearch&querykey=30) |

### T6Q2: Ursodeoxycholic acid (18 all study types)

| **Search** | **Query** | **Items found** |
| --- | --- | --- |
| [**#43**](https://www.ncbi.nlm.nih.gov/pubmed) | (#42 NOT (("Adolescent"[Mesh] OR "Child"[Mesh] OR "Infant"[Mesh] OR adolescen*[tiab] OR child*[tiab] OR schoolchild*[tiab] OR infant*[tiab] OR girl*[tiab] OR boy*[tiab] OR teen[tiab] OR teens[tiab] OR teenager*[tiab] OR youth*[tiab] OR pediatr*[tiab] OR paediatr*[tiab] OR puber*[tiab]) NOT ("Adult"[Mesh] OR adult*[tiab] OR man[tiab] OR men[tiab] OR woman[tiab] OR women[tiab]))) | [**18**](https://www.ncbi.nlm.nih.gov/pubmed/?cmd=HistorySearch&querykey=43) |
| [**#42**](https://www.ncbi.nlm.nih.gov/pubmed) | (#41 NOT ("Animals"[Mesh] NOT "Humans"[Mesh]) AND ("2004"[Date - Entrez] : "3000"[Date - Entrez])) | [**19**](https://www.ncbi.nlm.nih.gov/pubmed/?cmd=HistorySearch&querykey=42) |
| [**#41**](https://www.ncbi.nlm.nih.gov/pubmed) | (#30 AND #40) | [**21**](https://www.ncbi.nlm.nih.gov/pubmed/?cmd=HistorySearch&querykey=41) |
| [**#40**](https://www.ncbi.nlm.nih.gov/pubmed) | ("Ursodeoxycholic Acid"[Mesh] OR actigall[tiab] OR adursal[tiab] OR arsacol[tiab] OR bilifalk[tiab] OR “cgs 21240”[tiab] OR cgs21240[tiab] OR cholacid[tiab] OR “cholid ursan”[tiab] OR “cholit ursan”[tiab] OR cholofalk[tiab] OR “de ursil”[tiab] OR delursan[tiab] OR desoxil[tiab] OR destolit[tiab] OR deursil[tiab] OR estazor[tiab] OR litoff[tiab] OR litursol[tiab] OR peptarom[tiab] OR pramur[tiab] OR udihep[tiab] OR urdafalk[tiab] OR urosomix[tiab] OR ursacol[tiab] OR ursilon[tiab] OR urso[tiab] OR ursobil[tiab] OR ursobilin[tiab] OR ursochol[tiab] OR ursodeoxychol*[tiab] OR ursodiol[tiab] OR ursofalk[tiab] OR ursolin[tiab] OR ursolisin[tiab] OR ursolit[tiab] OR ursolvan[tiab] OR ursomedica[tiab] OR ursopol[tiab] OR ursosan[tiab] OR ursultec[tiab]) | [**5712**](https://www.ncbi.nlm.nih.gov/pubmed/?cmd=HistorySearch&querykey=40) |
| [**#30**](https://www.ncbi.nlm.nih.gov/pubmed) | ((("Bariatric Surgery"[Mesh] OR "Obesity/surgery"[Mesh:NoExp] OR "Obesity, Abdominal/surgery"[Mesh] OR "Obesity, Metabolically Benign/surgery"[Mesh] OR "Obesity, Morbid/surgery"[Mesh] OR "Anastomosis, Roux-en-Y"[Mesh] OR "Biliopancreatic Diversion"[Mesh] OR "Overweight/surgery"[Mesh] OR "Gastrectomy"[Mesh] OR bariatric surg*[tiab] OR bariatric operati*[tiab] OR bariatric procedure*[tiab] OR bilio pancreatic bypass*[tiab] OR bilio pancreatic diversion*[tiab] OR biliopancreatic bypass*[tiab] OR biliopancreatic diversion*[tiab] OR duodenal switch*[tiab] OR gastric band*[tiab] OR stomach band*[tiab] OR gastric bypass*[tiab] OR gastric partiti*[tiab] OR gastric plicati*[tiab] OR gastroplast*[tiab] OR mgbp*[tiab] OR oagb*[tiab] OR obese surg*[tiab] OR weight loss surg*[tiab] OR obesity surg*[tiab] OR metabolic surg*[tiab] OR roux en y*[tiab] OR “roux y”[tiab] OR “roux n y”[tiab] OR “roux in y”[tiab] OR rya[tiab] OR rygb*[tiab] OR sadis[tiab] OR “sadi s”[tiab] OR sagb*[tiab] OR sleeve gastrectom*[tiab] OR gastric sleeve*[tiab] OR gastrectom*[tiab] OR ((bariatric[ti] OR obes*[ti] OR weight loss[ti] OR metabolic[ti]) AND (surg*[ti] OR operati*[ti] OR procedure*[ti])) OR ((biliopancreatic[ti] OR “bilio pancreatic”[ti]) AND (diversion*[ti] OR bypass*[ti])) OR (duodenal[ti] AND switch*[ti]) OR ((stomach[ti] OR gastric[ti]) AND (band*[ti] OR bypass*[ti] OR partiti*[ti] OR plicati*[ti] OR sleeve*[ti])) OR (sleeve[ti] AND gastrectom*[ti]) OR (roux[ti] AND y[ti])) AND ("Laparoscopy"[Mesh:NoExp] OR "Minimally Invasive Surgical Procedures"[Mesh:NoExp] OR laparoscop*[tiab] OR minimally invasive[tiab] OR minimal invasive[tiab] OR minimal access[tiab] OR minimal surg*[tiab] OR (minimal*[ti] AND invasive[ti]) OR (minimal[ti] AND (access[ti] OR surg*[ti])))) OR (lagb*[tiab] OR lrygb*[tiab] OR lap band*[tiab] OR lrygb*[tiab])) | [**13120**](https://www.ncbi.nlm.nih.gov/pubmed/?cmd=HistorySearch&querykey=30) |

### T6Q3-4: Pregnancy (40 SRs, 14 RCTs, 43 cohort studies)

| **Search** | **Query** | **Items found** |
| --- | --- | --- |
| [**#50**](https://www.ncbi.nlm.nih.gov/pubmed) | ((#47 AND #39) NOT (#48 OR #49)) | [**43**](https://www.ncbi.nlm.nih.gov/pubmed/?cmd=HistorySearch&querykey=50) |
| [**#49**](https://www.ncbi.nlm.nih.gov/pubmed) | (#47 AND #37 NOT #48) | [**14**](https://www.ncbi.nlm.nih.gov/pubmed/?cmd=HistorySearch&querykey=49) |
| [**#48**](https://www.ncbi.nlm.nih.gov/pubmed) | (#47 AND #35) | [**40**](https://www.ncbi.nlm.nih.gov/pubmed/?cmd=HistorySearch&querykey=48) |
| [**#47**](https://www.ncbi.nlm.nih.gov/pubmed) | (#46 NOT (("Adolescent"[Mesh] OR "Child"[Mesh] OR "Infant"[Mesh] OR adolescen*[tiab] OR child*[tiab] OR schoolchild*[tiab] OR infant*[tiab] OR girl*[tiab] OR boy*[tiab] OR teen[tiab] OR teens[tiab] OR teenager*[tiab] OR youth*[tiab] OR pediatr*[tiab] OR paediatr*[tiab] OR puber*[tiab]) NOT ("Adult"[Mesh] OR adult*[tiab] OR man[tiab] OR men[tiab] OR woman[tiab] OR women[tiab]))) | [**126**](https://www.ncbi.nlm.nih.gov/pubmed/?cmd=HistorySearch&querykey=47) |
| [**#46**](https://www.ncbi.nlm.nih.gov/pubmed) | (#45 NOT ("Animals"[Mesh] NOT "Humans"[Mesh]) AND ("2004"[Date - Entrez] : "3000"[Date - Entrez])) | [**130**](https://www.ncbi.nlm.nih.gov/pubmed/?cmd=HistorySearch&querykey=46) |
| [**#45**](https://www.ncbi.nlm.nih.gov/pubmed) | (#30 AND #44) | [**143**](https://www.ncbi.nlm.nih.gov/pubmed/?cmd=HistorySearch&querykey=45) |
| [**#44**](https://www.ncbi.nlm.nih.gov/pubmed) | ("Pregnancy"[Mesh] OR "Time-to-Pregnancy"[Mesh] OR pregnan*[tiab]) | [**952052**](https://www.ncbi.nlm.nih.gov/pubmed/?cmd=HistorySearch&querykey=44) |
| [**#43**](https://www.ncbi.nlm.nih.gov/pubmed) | (#42 NOT (("Adolescent"[Mesh] OR "Child"[Mesh] OR "Infant"[Mesh] OR adolescen*[tiab] OR child*[tiab] OR schoolchild*[tiab] OR infant*[tiab] OR girl*[tiab] OR boy*[tiab] OR teen[tiab] OR teens[tiab] OR teenager*[tiab] OR youth*[tiab] OR pediatr*[tiab] OR paediatr*[tiab] OR puber*[tiab]) NOT ("Adult"[Mesh] OR adult*[tiab] OR man[tiab] OR men[tiab] OR woman[tiab] OR women[tiab]))) | [**18**](https://www.ncbi.nlm.nih.gov/pubmed/?cmd=HistorySearch&querykey=43) |
| [**#42**](https://www.ncbi.nlm.nih.gov/pubmed) | (#41 NOT ("Animals"[Mesh] NOT "Humans"[Mesh]) AND ("2004"[Date - Entrez] : "3000"[Date - Entrez])) | [**19**](https://www.ncbi.nlm.nih.gov/pubmed/?cmd=HistorySearch&querykey=42) |
| [**#41**](https://www.ncbi.nlm.nih.gov/pubmed) | (#30 AND #40) | [**21**](https://www.ncbi.nlm.nih.gov/pubmed/?cmd=HistorySearch&querykey=41) |
| [**#40**](https://www.ncbi.nlm.nih.gov/pubmed) | ("Ursodeoxycholic Acid"[Mesh] OR actigall[tiab] OR adursal[tiab] OR arsacol[tiab] OR bilifalk[tiab] OR “cgs 21240”[tiab] OR cgs21240[tiab] OR cholacid[tiab] OR “cholid ursan”[tiab] OR “cholit ursan”[tiab] OR cholofalk[tiab] OR “de ursil”[tiab] OR delursan[tiab] OR desoxil[tiab] OR destolit[tiab] OR deursil[tiab] OR estazor[tiab] OR litoff[tiab] OR litursol[tiab] OR peptarom[tiab] OR pramur[tiab] OR udihep[tiab] OR urdafalk[tiab] OR urosomix[tiab] OR ursacol[tiab] OR ursilon[tiab] OR urso[tiab] OR ursobil[tiab] OR ursobilin[tiab] OR ursochol[tiab] OR ursodeoxychol*[tiab] OR ursodiol[tiab] OR ursofalk[tiab] OR ursolin[tiab] OR ursolisin[tiab] OR ursolit[tiab] OR ursolvan[tiab] OR ursomedica[tiab] OR ursopol[tiab] OR ursosan[tiab] OR ursultec[tiab]) | [**5712**](https://www.ncbi.nlm.nih.gov/pubmed/?cmd=HistorySearch&querykey=40) |
| [**#39**](https://www.ncbi.nlm.nih.gov/pubmed) | ("Epidemiologic Studies"[Mesh] OR "Prognosis"[Mesh] OR cohort[tiab] OR (case[tiab] AND (control[tiab] OR controll*[tiab] OR comparison[tiab] OR referent[tiab])) OR risk[tiab] OR causation[tiab] OR causal[tiab] OR "odds ratio"[tiab] OR etiol*[tiab] OR aetiol*[tiab] OR "natural history"[tiab] OR predict*[tiab] OR outcome[tiab] OR course[tiab] OR retrospect*[tiab] OR “follow up”[tiab] OR followup[tiab] OR prognos*[tiab]) | [**6612503**](https://www.ncbi.nlm.nih.gov/pubmed/?cmd=HistorySearch&querykey=39) |
| [**#37**](https://www.ncbi.nlm.nih.gov/pubmed) | ("Randomized Controlled Trial"[Publication Type] OR "Controlled Clinical Trial"[Publication Type] OR random*[tiab] OR placebo[tiab] OR "Drug Therapy"[Subheading] OR trial[tiab] OR groups[tiab]) | [**4576640**](https://www.ncbi.nlm.nih.gov/pubmed/?cmd=HistorySearch&querykey=37) |
| [**#35**](https://www.ncbi.nlm.nih.gov/pubmed) | ((review[tiab] OR "Review"[Publication Type] OR "Meta-Analysis as Topic"[Mesh] OR meta-analysis[tiab] OR "Meta-Analysis"[Publication Type]) NOT ("Letter"[Publication Type] OR "Editorial"[Publication Type] OR "Comment"[Publication Type])) | [**2998395**](https://www.ncbi.nlm.nih.gov/pubmed/?cmd=HistorySearch&querykey=35) |
| [**#34**](https://www.ncbi.nlm.nih.gov/pubmed) | (#33 NOT (("Adolescent"[Mesh] OR "Child"[Mesh] OR "Infant"[Mesh] OR adolescen*[tiab] OR child*[tiab] OR schoolchild*[tiab] OR infant*[tiab] OR girl*[tiab] OR boy*[tiab] OR teen[tiab] OR teens[tiab] OR teenager*[tiab] OR youth*[tiab] OR pediatr*[tiab] OR paediatr*[tiab] OR puber*[tiab]) NOT ("Adult"[Mesh] OR adult*[tiab] OR man[tiab] OR men[tiab] OR woman[tiab] OR women[tiab]))) | [**202**](https://www.ncbi.nlm.nih.gov/pubmed/?cmd=HistorySearch&querykey=34) |
| [**#33**](https://www.ncbi.nlm.nih.gov/pubmed) | (#32 NOT ("Animals"[Mesh] NOT "Humans"[Mesh]) AND ("2004"[Date - Entrez] : "3000"[Date - Entrez])) | [**211**](https://www.ncbi.nlm.nih.gov/pubmed/?cmd=HistorySearch&querykey=33) |
| [**#32**](https://www.ncbi.nlm.nih.gov/pubmed) | (#30 AND #31) | [**220**](https://www.ncbi.nlm.nih.gov/pubmed/?cmd=HistorySearch&querykey=32) |
| [**#31**](https://www.ncbi.nlm.nih.gov/pubmed) | ("Micronutrients"[Mesh] OR "Dietary Supplements"[Mesh] OR "Dietary Fiber"[Mesh] OR "Dietary Proteins"[Mesh] OR micronutrient*[tiab] OR macronutrient*[tiab] OR micro nutrient*[tiab] OR macro nutrient*[tiab] OR vitamin*[tiab] OR provitamin*[tiab] OR trace element*[tiab] OR biometal*[tiab] OR prebiotic*[tiab] OR probiotic*[tiab] OR synbiotic*[tiab] OR dietary protein*[tiab] OR protein supplement*[tiab] OR dietary supplement*[tiab] OR food supplement*[tiab] OR nutraceutical*[tiab] OR nutriceutical*[tiab] OR neutraceutical*[tiab] OR dietary fiber*[tiab] OR wheat bran*[tiab] OR roughage*[tiab]) | [**426840**](https://www.ncbi.nlm.nih.gov/pubmed/?cmd=HistorySearch&querykey=31) |
| [**#30**](https://www.ncbi.nlm.nih.gov/pubmed) | ((("Bariatric Surgery"[Mesh] OR "Obesity/surgery"[Mesh:NoExp] OR "Obesity, Abdominal/surgery"[Mesh] OR "Obesity, Metabolically Benign/surgery"[Mesh] OR "Obesity, Morbid/surgery"[Mesh] OR "Anastomosis, Roux-en-Y"[Mesh] OR "Biliopancreatic Diversion"[Mesh] OR "Overweight/surgery"[Mesh] OR "Gastrectomy"[Mesh] OR bariatric surg*[tiab] OR bariatric operati*[tiab] OR bariatric procedure*[tiab] OR bilio pancreatic bypass*[tiab] OR bilio pancreatic diversion*[tiab] OR biliopancreatic bypass*[tiab] OR biliopancreatic diversion*[tiab] OR duodenal switch*[tiab] OR gastric band*[tiab] OR stomach band*[tiab] OR gastric bypass*[tiab] OR gastric partiti*[tiab] OR gastric plicati*[tiab] OR gastroplast*[tiab] OR mgbp*[tiab] OR oagb*[tiab] OR obese surg*[tiab] OR weight loss surg*[tiab] OR obesity surg*[tiab] OR metabolic surg*[tiab] OR roux en y*[tiab] OR “roux y”[tiab] OR “roux n y”[tiab] OR “roux in y”[tiab] OR rya[tiab] OR rygb*[tiab] OR sadis[tiab] OR “sadi s”[tiab] OR sagb*[tiab] OR sleeve gastrectom*[tiab] OR gastric sleeve*[tiab] OR gastrectom*[tiab] OR ((bariatric[ti] OR obes*[ti] OR weight loss[ti] OR metabolic[ti]) AND (surg*[ti] OR operati*[ti] OR procedure*[ti])) OR ((biliopancreatic[ti] OR “bilio pancreatic”[ti]) AND (diversion*[ti] OR bypass*[ti])) OR (duodenal[ti] AND switch*[ti]) OR ((stomach[ti] OR gastric[ti]) AND (band*[ti] OR bypass*[ti] OR partiti*[ti] OR plicati*[ti] OR sleeve*[ti])) OR (sleeve[ti] AND gastrectom*[ti]) OR (roux[ti] AND y[ti])) AND ("Laparoscopy"[Mesh:NoExp] OR "Minimally Invasive Surgical Procedures"[Mesh:NoExp] OR laparoscop*[tiab] OR minimally invasive[tiab] OR minimal invasive[tiab] OR minimal access[tiab] OR minimal surg*[tiab] OR (minimal*[ti] AND invasive[ti]) OR (minimal[ti] AND (access[ti] OR surg*[ti])))) OR (lagb*[tiab] OR lrygb*[tiab] OR lap band*[tiab] OR lrygb*[tiab])) | [**13120**](https://www.ncbi.nlm.nih.gov/pubmed/?cmd=HistorySearch&querykey=30) |

### T6Q5: PPI (29 SRs, 39 RCTs, 45 cohort studies) (11 February 2019)

| **Search** | **Query** | **Items found** |
| --- | --- | --- |
| [**#24**](https://www.ncbi.nlm.nih.gov/pubmed) | ((#18 AND #23) NOT (#20 OR #22)) | [**45**](https://www.ncbi.nlm.nih.gov/pubmed/?cmd=HistorySearch&querykey=24) |
| [**#23**](https://www.ncbi.nlm.nih.gov/pubmed) | ("Epidemiologic Studies"[Mesh] OR "Prognosis"[Mesh] OR cohort[tiab] OR (case[tiab] AND (control[tiab] OR controll*[tiab] OR comparison[tiab] OR referent[tiab])) OR risk[tiab] OR causation[tiab] OR causal[tiab] OR "odds ratio"[tiab] OR etiol*[tiab] OR aetiol*[tiab] OR "natural history"[tiab] OR predict*[tiab] OR outcome[tiab] OR course[tiab] OR retrospect*[tiab] OR “follow up”[tiab] OR followup[tiab] OR prognos*[tiab]) | [**6709293**](https://www.ncbi.nlm.nih.gov/pubmed/?cmd=HistorySearch&querykey=23) |
| [**#22**](https://www.ncbi.nlm.nih.gov/pubmed) | ((#18 AND #21) NOT #20) | [**39**](https://www.ncbi.nlm.nih.gov/pubmed/?cmd=HistorySearch&querykey=22) |
| [**#21**](https://www.ncbi.nlm.nih.gov/pubmed) | ("Randomized Controlled Trial"[Publication Type] OR "Controlled Clinical Trial"[Publication Type] OR random*[tiab] OR placebo[tiab] OR "Drug Therapy"[Subheading] OR trial[tiab] OR groups[tiab]) | [**4631826**](https://www.ncbi.nlm.nih.gov/pubmed/?cmd=HistorySearch&querykey=21) |
| [**#20**](https://www.ncbi.nlm.nih.gov/pubmed) | (#18 AND #19) | [**29**](https://www.ncbi.nlm.nih.gov/pubmed/?cmd=HistorySearch&querykey=20) |
| [**#19**](https://www.ncbi.nlm.nih.gov/pubmed) | ((review[tiab] OR "Review"[Publication Type] OR "Meta-Analysis as Topic"[Mesh] OR meta-analysis[tiab] OR "Meta-Analysis"[Publication Type]) NOT ("Letter"[Publication Type] OR "Editorial"[Publication Type] OR "Comment"[Publication Type])) | [**3035678**](https://www.ncbi.nlm.nih.gov/pubmed/?cmd=HistorySearch&querykey=19) |
| [**#18**](https://www.ncbi.nlm.nih.gov/pubmed) | (#17 NOT (("Adolescent"[Mesh] OR "Child"[Mesh] OR "Infant"[Mesh] OR adolescen*[tiab] OR child*[tiab] OR schoolchild*[tiab] OR infant*[tiab] OR girl*[tiab] OR boy*[tiab] OR teen[tiab] OR teens[tiab] OR teenager*[tiab] OR youth*[tiab] OR pediatr*[tiab] OR paediatr*[tiab] OR puber*[tiab]) NOT ("Adult"[Mesh] OR adult*[tiab] OR man[tiab] OR men[tiab] OR woman[tiab] OR women[tiab]))) | [**132**](https://www.ncbi.nlm.nih.gov/pubmed/?cmd=HistorySearch&querykey=18) |
| [**#17**](https://www.ncbi.nlm.nih.gov/pubmed) | (#16 NOT ("Animals"[Mesh] NOT "Humans"[Mesh]) AND ("2004"[Date - Entrez] : "3000"[Date - Entrez])) | [**133**](https://www.ncbi.nlm.nih.gov/pubmed/?cmd=HistorySearch&querykey=17) |
| [**#16**](https://www.ncbi.nlm.nih.gov/pubmed) | (#14 AND #15) | [**147**](https://www.ncbi.nlm.nih.gov/pubmed/?cmd=HistorySearch&querykey=16) |
| [**#15**](https://www.ncbi.nlm.nih.gov/pubmed) | "Proton Pump Inhibitors"[Mesh] OR "Proton Pump Inhibitors" [Pharmacological Action] OR proton pump inhibit*[tiab] OR ppi[tiab] OR ppis OR dexlansoprazol*[tiab] OR lansoprazol*[tiab] OR esomeprazol*[tiab] OR nexium[tiab] OR lansoprazol*[tiab] OR omeprazol*[tiab] OR prilosec[tiab] OR pantoprazol*[tiab] OR protonix[tiab] OR rabeprazol*[tiab] OR dexrabeprazol*[tiab] OR aciphex[tiab] OR ogastro[tiab] OR agopton[tiab] OR bamalite[tiab] OR lansol[tiab] OR lanzor[tiab] OR monolitum[tiab] OR opiren[tiab] OR prevacid[tiab] OR “pro ulco”[tiab] OR promeco[tiab] OR takepron[tiab] OR ulpax[tiab] OR zoton[tiab] OR ogast[tiab] OR prezal[tiab] | [**36833**](https://www.ncbi.nlm.nih.gov/pubmed/?cmd=HistorySearch&querykey=15) |
| [**#14**](https://www.ncbi.nlm.nih.gov/pubmed) | (("Bariatric Surgery"[Mesh] OR "Obesity/surgery"[Mesh:NoExp] OR "Obesity, Abdominal/surgery"[Mesh] OR "Obesity, Metabolically Benign/surgery"[Mesh] OR "Obesity, Morbid/surgery"[Mesh] OR "Anastomosis, Roux-en-Y"[Mesh] OR "Biliopancreatic Diversion"[Mesh] OR "Overweight/surgery"[Mesh] OR "Gastrectomy"[Mesh] OR bariatric surg*[tiab] OR bariatric operati*[tiab] OR bariatric procedure*[tiab] OR bilio pancreatic bypass*[tiab] OR bilio pancreatic diversion*[tiab] OR biliopancreatic bypass*[tiab] OR biliopancreatic diversion*[tiab] OR duodenal switch*[tiab] OR gastric band*[tiab] OR stomach band*[tiab] OR gastric bypass*[tiab] OR gastric partiti*[tiab] OR gastric plicati*[tiab] OR gastroplast*[tiab] OR mgbp*[tiab] OR oagb*[tiab] OR obese surg*[tiab] OR weight loss surg*[tiab] OR obesity surg*[tiab] OR metabolic surg*[tiab] OR roux en y*[tiab] OR “roux y”[tiab] OR “roux n y”[tiab] OR “roux in y”[tiab] OR rya[tiab] OR rygb*[tiab] OR sadis[tiab] OR “sadi s”[tiab] OR sagb*[tiab] OR sleeve gastrectom*[tiab] OR gastric sleeve*[tiab] OR gastrectom*[tiab] OR ((bariatric[ti] OR obes*[ti] OR weight loss[ti] OR metabolic[ti]) AND (surg*[ti] OR operati*[ti] OR procedure*[ti])) OR ((biliopancreatic[ti] OR “bilio pancreatic”[ti]) AND (diversion*[ti] OR bypass*[ti])) OR (duodenal[ti] AND switch*[ti]) OR ((stomach[ti] OR gastric[ti]) AND (band*[ti] OR bypass*[ti] OR partiti*[ti] OR plicati*[ti] OR sleeve*[ti])) OR (sleeve[ti] AND gastrectom*[ti]) OR (roux[ti] AND y[ti])) AND ("Laparoscopy"[Mesh:NoExp] OR "Minimally Invasive Surgical Procedures"[Mesh:NoExp] OR laparoscop*[tiab] OR minimally invasive[tiab] OR minimal invasive[tiab] OR minimal access[tiab] OR minimal surg*[tiab] OR (minimal*[ti] AND invasive[ti]) OR (minimal[ti] AND (access[ti] OR surg*[ti])))) OR (lagb*[tiab] OR lrygb*[tiab] OR lap band*[tiab] OR lrygb*[tiab]) | [**13393**](https://www.ncbi.nlm.nih.gov/pubmed/?cmd=HistorySearch&querykey=14) |

### T6Q6: Postoperative control schedule (12 SRs, 62 RCTs, 59 cohort studies) (20 February 2019)

| **Search** | **Query** | **Items found** |
| --- | --- | --- |
| [**#47**](https://www.ncbi.nlm.nih.gov/pubmed) | ((#41 AND #46) NOT (#43 OR #45)) | [**59**](https://www.ncbi.nlm.nih.gov/pubmed/?cmd=HistorySearch&querykey=47) |
| [**#46**](https://www.ncbi.nlm.nih.gov/pubmed) | ("Epidemiologic Studies"[Mesh] OR "Prognosis"[Mesh] OR cohort[tiab] OR (case[tiab] AND (control[tiab] OR controll*[tiab] OR comparison[tiab] OR referent[tiab])) OR risk[tiab] OR causation[tiab] OR causal[tiab] OR "odds ratio"[tiab] OR etiol*[tiab] OR aetiol*[tiab] OR "natural history"[tiab] OR predict*[tiab] OR outcome[tiab] OR course[tiab] OR retrospect*[tiab] OR “follow up”[tiab] OR followup[tiab] OR prognos*[tiab]) | [**6721455**](https://www.ncbi.nlm.nih.gov/pubmed/?cmd=HistorySearch&querykey=46) |
| [**#45**](https://www.ncbi.nlm.nih.gov/pubmed) | ((#41 AND #44) NOT #43) | [**62**](https://www.ncbi.nlm.nih.gov/pubmed/?cmd=HistorySearch&querykey=45) |
| [**#44**](https://www.ncbi.nlm.nih.gov/pubmed) | ("Randomized Controlled Trial"[Publication Type] OR "Controlled Clinical Trial"[Publication Type] OR random*[tiab] OR placebo[tiab] OR "Drug Therapy"[Subheading] OR trial[tiab] OR groups[tiab]) | [**4638364**](https://www.ncbi.nlm.nih.gov/pubmed/?cmd=HistorySearch&querykey=44) |
| [**#43**](https://www.ncbi.nlm.nih.gov/pubmed) | (#41 AND #42) | [**12**](https://www.ncbi.nlm.nih.gov/pubmed/?cmd=HistorySearch&querykey=43) |
| [**#42**](https://www.ncbi.nlm.nih.gov/pubmed) | ((review[tiab] OR "Review"[Publication Type] OR "Meta-Analysis as Topic"[Mesh] OR meta-analysis[tiab] OR "Meta-Analysis"[Publication Type]) NOT ("Letter"[Publication Type] OR "Editorial"[Publication Type] OR "Comment"[Publication Type])) | [**3040205**](https://www.ncbi.nlm.nih.gov/pubmed/?cmd=HistorySearch&querykey=42) |
| [**#41**](https://www.ncbi.nlm.nih.gov/pubmed) | (#40 NOT (("Adolescent"[Mesh] OR "Child"[Mesh] OR "Infant"[Mesh] OR adolescen*[tiab] OR child*[tiab] OR schoolchild*[tiab] OR infant*[tiab] OR girl*[tiab] OR boy*[tiab] OR teen[tiab] OR teens[tiab] OR teenager*[tiab] OR youth*[tiab] OR pediatr*[tiab] OR paediatr*[tiab] OR puber*[tiab]) NOT ("Adult"[Mesh] OR adult*[tiab] OR man[tiab] OR men[tiab] OR woman[tiab] OR women[tiab]))) | [**145**](https://www.ncbi.nlm.nih.gov/pubmed/?cmd=HistorySearch&querykey=41) |
| [**#40**](https://www.ncbi.nlm.nih.gov/pubmed) | (#39 NOT ("Animals"[Mesh] NOT "Humans"[Mesh]) AND ("2004"[Date - Entrez] : "3000"[Date - Entrez])) | [**145**](https://www.ncbi.nlm.nih.gov/pubmed/?cmd=HistorySearch&querykey=40) |
| [**#39**](https://www.ncbi.nlm.nih.gov/pubmed) | (#37 AND #38) | [**158**](https://www.ncbi.nlm.nih.gov/pubmed/?cmd=HistorySearch&querykey=39) |
| [**#38**](https://www.ncbi.nlm.nih.gov/pubmed) | ((("Postoperative Complications"[Mesh] OR "Postoperative Period"[Mesh] OR "Postoperative Care"[Mesh] OR postoperati*[tiab] OR post operati*[tiab] OR after surg*[tiab]) AND (intens*[tiab] OR schedul*[tiab]) AND ("Monitoring, Physiologic"[Mesh] OR monitor*[tiab] OR check up*[tiab] OR checkup*[tiab] OR office visit*[tiab] OR schedul*[tiab] OR control[tiab]))) | [**23133**](https://www.ncbi.nlm.nih.gov/pubmed/?cmd=HistorySearch&querykey=38) |
| [**#37**](https://www.ncbi.nlm.nih.gov/pubmed) | ((("Bariatric Surgery"[Mesh] OR "Obesity/surgery"[Mesh:NoExp] OR "Obesity, Abdominal/surgery"[Mesh] OR "Obesity, Metabolically Benign/surgery"[Mesh] OR "Obesity, Morbid/surgery"[Mesh] OR "Anastomosis, Roux-en-Y"[Mesh] OR "Biliopancreatic Diversion"[Mesh] OR "Overweight/surgery"[Mesh] OR "Gastrectomy"[Mesh] OR bariatric surg*[tiab] OR bariatric operati*[tiab] OR bariatric procedure*[tiab] OR bilio pancreatic bypass*[tiab] OR bilio pancreatic diversion*[tiab] OR biliopancreatic bypass*[tiab] OR biliopancreatic diversion*[tiab] OR duodenal switch*[tiab] OR gastric band*[tiab] OR stomach band*[tiab] OR gastric bypass*[tiab] OR gastric partiti*[tiab] OR gastric plicati*[tiab] OR gastroplast*[tiab] OR mgbp*[tiab] OR oagb*[tiab] OR obese surg*[tiab] OR weight loss surg*[tiab] OR obesity surg*[tiab] OR metabolic surg*[tiab] OR roux en y*[tiab] OR “roux y”[tiab] OR “roux n y”[tiab] OR “roux in y”[tiab] OR rya[tiab] OR rygb*[tiab] OR sadis[tiab] OR “sadi s”[tiab] OR sagb*[tiab] OR sleeve gastrectom*[tiab] OR gastric sleeve*[tiab] OR gastrectom*[tiab] OR ((bariatric[ti] OR obes*[ti] OR weight loss[ti] OR metabolic[ti]) AND (surg*[ti] OR operati*[ti] OR procedure*[ti])) OR ((biliopancreatic[ti] OR “bilio pancreatic”[ti]) AND (diversion*[ti] OR bypass*[ti])) OR (duodenal[ti] AND switch*[ti]) OR ((stomach[ti] OR gastric[ti]) AND (band*[ti] OR bypass*[ti] OR partiti*[ti] OR plicati*[ti] OR sleeve*[ti])) OR (sleeve[ti] AND gastrectom*[ti]) OR (roux[ti] AND y[ti])) AND ("Laparoscopy"[Mesh:NoExp] OR "Minimally Invasive Surgical Procedures"[Mesh:NoExp] OR laparoscop*[tiab] OR minimally invasive[tiab] OR minimal invasive[tiab] OR minimal access[tiab] OR minimal surg*[tiab] OR (minimal*[ti] AND invasive[ti]) OR (minimal[ti] AND (access[ti] OR surg*[ti])))) OR (lagb*[tiab] OR lrygb*[tiab] OR lap band*[tiab] OR lrygb*[tiab])) | [**13446**](https://www.ncbi.nlm.nih.gov/pubmed/?cmd=HistorySearch&querykey=37) |

### T6Q7: Sequential diet regimen (30 SRs, 73 RCTs, 105 cohort studies) (20 February 2019)

| **Search** | **Query** | **Items found** |
| --- | --- | --- |
| [**#67**](https://www.ncbi.nlm.nih.gov/pubmed) | ((#64 AND #46) NOT (#65 OR #66)) | [**105**](https://www.ncbi.nlm.nih.gov/pubmed/?cmd=HistorySearch&querykey=67) |
| [**#66**](https://www.ncbi.nlm.nih.gov/pubmed) | ((#64 AND #44) NOT #65) | [**73**](https://www.ncbi.nlm.nih.gov/pubmed/?cmd=HistorySearch&querykey=66) |
| [**#65**](https://www.ncbi.nlm.nih.gov/pubmed) | (#64 AND #42) | [**30**](https://www.ncbi.nlm.nih.gov/pubmed/?cmd=HistorySearch&querykey=65) |
| [**#64**](https://www.ncbi.nlm.nih.gov/pubmed) | (#63 NOT (("Adolescent"[Mesh] OR "Child"[Mesh] OR "Infant"[Mesh] OR adolescen*[tiab] OR child*[tiab] OR schoolchild*[tiab] OR infant*[tiab] OR girl*[tiab] OR boy*[tiab] OR teen[tiab] OR teens[tiab] OR teenager*[tiab] OR youth*[tiab] OR pediatr*[tiab] OR paediatr*[tiab] OR puber*[tiab]) NOT ("Adult"[Mesh] OR adult*[tiab] OR man[tiab] OR men[tiab] OR woman[tiab] OR women[tiab]))) | [**235**](https://www.ncbi.nlm.nih.gov/pubmed/?cmd=HistorySearch&querykey=64) |
| [**#63**](https://www.ncbi.nlm.nih.gov/pubmed) | (#62 NOT ("Animals"[Mesh] NOT "Humans"[Mesh]) AND ("2004"[Date - Entrez] : "3000"[Date - Entrez])) | [**248**](https://www.ncbi.nlm.nih.gov/pubmed/?cmd=HistorySearch&querykey=63) |
| [**#62**](https://www.ncbi.nlm.nih.gov/pubmed) | (#37 AND #60 AND #61) | [**275**](https://www.ncbi.nlm.nih.gov/pubmed/?cmd=HistorySearch&querykey=62) |
| [**#61**](https://www.ncbi.nlm.nih.gov/pubmed) | ("Postoperative Complications"[Mesh] OR "Postoperative Period"[Mesh] OR "Postoperative Care"[Mesh] OR postoperati*[tiab] OR post operati*[tiab] OR after surg*[tiab]) | [**1022008**](https://www.ncbi.nlm.nih.gov/pubmed/?cmd=HistorySearch&querykey=61) |
| [**#60**](https://www.ncbi.nlm.nih.gov/pubmed) | ("Diet Therapy"[Mesh] OR ((diet[tiab] OR diets[tiab] OR dietary[tiab] OR calor*[tiab]) AND (regimen*[tiab] OR restrict*[tiab] OR low carb*[tiab] OR reduc*[tiab] OR gluten[tiab] OR fat[tiab] OR fats[tiab] OR carbohydrat*[tiab] OR loading[tiab] OR low calor*[tiab] OR protein[tiab] OR proteins[tiab] OR south beach[tiab] OR atkins[tiab] OR ketogen*[tiab] OR sequenti*[tiab] OR weight loss[tiab] OR modificat*[tiab]))) | [**326155**](https://www.ncbi.nlm.nih.gov/pubmed/?cmd=HistorySearch&querykey=60) |
| [**#46**](https://www.ncbi.nlm.nih.gov/pubmed) | ("Epidemiologic Studies"[Mesh] OR "Prognosis"[Mesh] OR cohort[tiab] OR (case[tiab] AND (control[tiab] OR controll*[tiab] OR comparison[tiab] OR referent[tiab])) OR risk[tiab] OR causation[tiab] OR causal[tiab] OR "odds ratio"[tiab] OR etiol*[tiab] OR aetiol*[tiab] OR "natural history"[tiab] OR predict*[tiab] OR outcome[tiab] OR course[tiab] OR retrospect*[tiab] OR “follow up”[tiab] OR followup[tiab] OR prognos*[tiab]) | [**6721455**](https://www.ncbi.nlm.nih.gov/pubmed/?cmd=HistorySearch&querykey=46) |
| [**#44**](https://www.ncbi.nlm.nih.gov/pubmed) | ("Randomized Controlled Trial"[Publication Type] OR "Controlled Clinical Trial"[Publication Type] OR random*[tiab] OR placebo[tiab] OR "Drug Therapy"[Subheading] OR trial[tiab] OR groups[tiab]) | [**4638364**](https://www.ncbi.nlm.nih.gov/pubmed/?cmd=HistorySearch&querykey=44) |
| [**#42**](https://www.ncbi.nlm.nih.gov/pubmed) | ((review[tiab] OR "Review"[Publication Type] OR "Meta-Analysis as Topic"[Mesh] OR meta-analysis[tiab] OR "Meta-Analysis"[Publication Type]) NOT ("Letter"[Publication Type] OR "Editorial"[Publication Type] OR "Comment"[Publication Type])) | [**3040205**](https://www.ncbi.nlm.nih.gov/pubmed/?cmd=HistorySearch&querykey=42) |
| [**#37**](https://www.ncbi.nlm.nih.gov/pubmed) | ((("Bariatric Surgery"[Mesh] OR "Obesity/surgery"[Mesh:NoExp] OR "Obesity, Abdominal/surgery"[Mesh] OR "Obesity, Metabolically Benign/surgery"[Mesh] OR "Obesity, Morbid/surgery"[Mesh] OR "Anastomosis, Roux-en-Y"[Mesh] OR "Biliopancreatic Diversion"[Mesh] OR "Overweight/surgery"[Mesh] OR "Gastrectomy"[Mesh] OR bariatric surg*[tiab] OR bariatric operati*[tiab] OR bariatric procedure*[tiab] OR bilio pancreatic bypass*[tiab] OR bilio pancreatic diversion*[tiab] OR biliopancreatic bypass*[tiab] OR biliopancreatic diversion*[tiab] OR duodenal switch*[tiab] OR gastric band*[tiab] OR stomach band*[tiab] OR gastric bypass*[tiab] OR gastric partiti*[tiab] OR gastric plicati*[tiab] OR gastroplast*[tiab] OR mgbp*[tiab] OR oagb*[tiab] OR obese surg*[tiab] OR weight loss surg*[tiab] OR obesity surg*[tiab] OR metabolic surg*[tiab] OR roux en y*[tiab] OR “roux y”[tiab] OR “roux n y”[tiab] OR “roux in y”[tiab] OR rya[tiab] OR rygb*[tiab] OR sadis[tiab] OR “sadi s”[tiab] OR sagb*[tiab] OR sleeve gastrectom*[tiab] OR gastric sleeve*[tiab] OR gastrectom*[tiab] OR ((bariatric[ti] OR obes*[ti] OR weight loss[ti] OR metabolic[ti]) AND (surg*[ti] OR operati*[ti] OR procedure*[ti])) OR ((biliopancreatic[ti] OR “bilio pancreatic”[ti]) AND (diversion*[ti] OR bypass*[ti])) OR (duodenal[ti] AND switch*[ti]) OR ((stomach[ti] OR gastric[ti]) AND (band*[ti] OR bypass*[ti] OR partiti*[ti] OR plicati*[ti] OR sleeve*[ti])) OR (sleeve[ti] AND gastrectom*[ti]) OR (roux[ti] AND y[ti])) AND ("Laparoscopy"[Mesh:NoExp] OR "Minimally Invasive Surgical Procedures"[Mesh:NoExp] OR laparoscop*[tiab] OR minimally invasive[tiab] OR minimal invasive[tiab] OR minimal access[tiab] OR minimal surg*[tiab] OR (minimal*[ti] AND invasive[ti]) OR (minimal[ti] AND (access[ti] OR surg*[ti])))) OR (lagb*[tiab] OR lrygb*[tiab] OR lap band*[tiab] OR lrygb*[tiab])) | [**13446**](https://www.ncbi.nlm.nih.gov/pubmed/?cmd=HistorySearch&querykey=37) |

## Embase.com (23 November 2018 + 11 February + 20 February 2019)

### T6Q1: Nutrients (67 SRs, 33 RCTs, 84 cohort studies)

| **No.** | **Query** | **Results** |
| --- | --- | --- |
| **#13** | #8 AND #12 NOT (#9 OR #11) | **84** |
| **#12** | 'epidemiology'/de OR (((cohort OR case) NEAR/3 (control OR controll* OR comparison OR referent)):ti,ab,kw) OR risk:ti,ab,kw OR causation:ti,ab,kw OR causal:ti,ab,kw OR 'odds ratio':ti,ab,kw OR etiol*:ti,ab,kw OR aetiol*:ti,ab,kw OR 'natural history':ti,ab,kw OR outcome:ti,ab,kw OR course:ti,ab,kw OR retrospect*:ti,ab,kw OR 'follow up':ti,ab,kw OR followup:ti,ab,kw OR predict*:ti,ab,kw OR prognos*:ti,ab,kw | **7337087** |
| **#11** | #8 AND #10 NOT #9 | **33** |
| **#10** | 'clinical':ti,ab,kw AND 'trial':ti,ab,kw OR 'clinical trial'/exp OR random*:ti,ab,kw | **2293116** |
| **#9** | #8 AND #2 | **67** |
| **#8** | #7 NOT (('adolescent'/exp OR 'child'/exp OR adolescent*:ti,ab OR child*:ti,ab OR schoolchild*:ti,ab OR infant*:ti,ab OR girl*:ti,ab OR boy*:ti,ab OR teen:ti,ab OR teens:ti,ab OR teenager*:ti,ab OR youth*:ti,ab OR pediatr*:ti,ab OR paediatr*:ti,ab OR puber*:ti,ab) NOT ('adult'/exp OR 'aged'/exp OR 'middle aged'/exp OR adult*:ti,ab OR man:ti,ab OR men:ti,ab OR woman:ti,ab OR women:ti,ab)) | **243** |
| **#7** | #6 NOT ('conference abstract'/it OR 'conference paper'/it OR 'letter'/it OR 'note'/it) | **247** |
| **#6** | #5 NOT ([animals]/lim NOT [humans]/lim) AND [1-1-2004]/sd | **377** |
| **#5** | #1 AND #3 AND #4 | **401** |
| **#4** | 'trace element'/exp/mj OR 'dietary supplement'/exp/mj OR 'dietary fiber'/exp/mj OR 'protein intake'/exp/mj OR 'macronutrient'/exp/mj OR 'vitamin'/exp/mj OR 'prebiotic agent'/exp/mj OR 'probiotic agent'/exp/mj OR micronutrient*:ti,kw OR macronutrient*:ti,kw OR 'micro nutrient*':ti,kw OR 'macro nutrient*':ti,kw OR vitamin*:ti,kw OR provitamin*:ti,kw OR 'trace element*':ti,kw OR biometal*:ti,kw OR prebiotic*:ti,kw OR probiotic*:ti,kw OR synbiotic*:ti,kw OR 'dietary protein*':ti,kw OR 'protein supplement*':ti,kw OR 'dietary supplement*':ti,kw OR 'food supplement*':ti,kw OR nutraceutical*:ti,kw OR nutriceutical*:ti,kw OR neutraceutical*:ti,kw OR 'dietary fiber*':ti,kw OR 'wheat bran*':ti,kw OR roughage*:ti,kw | **419006** |
| **#3** | 'postoperative period'/exp OR 'postoperative complication'/exp OR postoperati*:ti,ab,kw OR 'post operati*':ti,ab,kw OR postsurg*:ti,ab,kw OR 'post surg*':ti,ab,kw | **1424169** |
| **#2** | 'meta-analysis':ti,ab,kw OR 'meta analysis'/exp OR 'review'/exp OR review:ti,ab,kw | **3441138** |
| **#1** | ('obesity'/exp/mj AND 'surgery'/lnk OR 'bariatric surgery'/exp/mj OR 'roux y anastomosis'/exp/mj OR 'gastric bypass surgery'/exp/mj OR ((('bilio pancreatic' OR biliopancreatic) NEAR/3 (diversion* OR bypass*)):ti,kw) OR ((duodenal NEAR/3 switch*):ti,kw) OR (((stomach OR gastric) NEAR/3 (band* OR bypass* OR partiti* OR plicati* OR sleeve*)):ti,kw) OR ((sleeve NEAR/3 gastrectom*):ti,kw) OR ((roux NEAR/2 y):ti,kw) OR gastroplast*:ti,kw OR mgbp*:ti,kw OR oagb*:ti,kw OR rya:ti,kw OR rygb*:ti,kw OR sadis:ti,kw OR 'sadi s':ti,kw OR (((bariatric OR obes* OR 'weight loss' OR metabolic) NEAR/3 (surg* OR operati* OR procedure*)):ti,kw) OR sagb*:ti,kw) AND ('laparoscopy'/de OR 'laparoendoscopic single site surgery'/exp OR 'laparoscopic surgery'/exp OR 'minimally invasive procedure'/exp OR 'minimally invasive surgery'/exp OR laparoscop*:ti,ab,kw OR ((minimal* NEAR/3 invasive):ti,ab,kw) OR access:ti,ab,kw OR surg*:ti,ab,kw) OR 'laparoscopic sleeve gastrectomy'/exp OR lagb*:ti,ab,kw OR 'lap band*':ti,ab,kw OR lrygb*:ti,ab,kw | **40731** |

### T6Q2: Ursodeoxycholic acid (39 SRs, 11 RCTs, 26 cohort studies)

| **No.** | **Query** | **Results** |
| --- | --- | --- |
| **#13** | #10 AND #5 NOT (#11 OR #12) | **26** |
| **#12** | #10 AND #4 NOT #11 | **11** |
| **#11** | #2 AND #10 | **39** |
| **#10** | #9 NOT (('adolescent'/exp OR 'child'/exp OR adolescent*:ti,ab OR child*:ti,ab OR schoolchild*:ti,ab OR infant*:ti,ab OR girl*:ti,ab OR boy*:ti,ab OR teen:ti,ab OR teens:ti,ab OR teenager*:ti,ab OR youth*:ti,ab OR pediatr*:ti,ab OR paediatr*:ti,ab OR puber*:ti,ab) NOT ('adult'/exp OR 'aged'/exp OR 'middle aged'/exp OR adult*:ti,ab OR man:ti,ab OR men:ti,ab OR woman:ti,ab OR women:ti,ab)) | **80** |
| **#9** | #8 NOT ('conference abstract'/it OR 'conference paper'/it OR 'letter'/it OR 'note'/it) | **85** |
| **#8** | #7 NOT ([animals]/lim NOT [humans]/lim) AND [1-1-2004]/sd | **107** |
| **#7** | #6 AND #1 | **116** |
| **#6** | 'ursodeoxycholic acid'/exp OR 'ursodeoxycholic acid derivative'/exp OR actigall:ti,ab,kw OR adursal:ti,ab,kw OR arsacol:ti,ab,kw OR bilifalk:ti,ab,kw OR 'cgs 21240':ti,ab,kw OR cgs21240:ti,ab,kw OR cholacid:ti,ab,kw OR 'cholid ursan':ti,ab,kw OR 'cholit ursan':ti,ab,kw OR cholofalk:ti,ab,kw OR 'de ursil':ti,ab,kw OR delursan:ti,ab,kw OR desoxil:ti,ab,kw OR destolit:ti,ab,kw OR deursil:ti,ab,kw OR estazor:ti,ab,kw OR litoff:ti,ab,kw OR litursol:ti,ab,kw OR peptarom:ti,ab,kw OR pramur:ti,ab,kw OR udihep:ti,ab,kw OR urdafalk:ti,ab,kw OR urosomix:ti,ab,kw OR ursacol:ti,ab,kw OR ursilon:ti,ab,kw OR urso:ti,ab,kw OR ursobil:ti,ab,kw OR ursobilin:ti,ab,kw OR ursochol:ti,ab,kw OR ursodeoxychol*:ti,ab,kw OR ursodiol:ti,ab,kw OR ursofalk:ti,ab,kw OR ursolin:ti,ab,kw OR ursolisin:ti,ab,kw OR ursolit:ti,ab,kw OR ursolvan:ti,ab,kw OR ursomedica:ti,ab,kw OR ursopol:ti,ab,kw OR ursosan:ti,ab,kw OR ursultec:ti,ab,kw | **13590** |
| **#5** | 'epidemiology'/de OR (((cohort OR case) NEAR/3 (control OR controll* OR comparison OR referent)):ti,ab,kw) OR risk:ti,ab,kw OR causation:ti,ab,kw OR causal:ti,ab,kw OR 'odds ratio':ti,ab,kw OR etiol*:ti,ab,kw OR aetiol*:ti,ab,kw OR 'natural history':ti,ab,kw OR outcome:ti,ab,kw OR course:ti,ab,kw OR retrospect*:ti,ab,kw OR 'follow up':ti,ab,kw OR followup:ti,ab,kw OR predict*:ti,ab,kw OR prognos*:ti,ab,kw | **7337087** |
| **#4** | 'clinical':ti,ab,kw AND 'trial':ti,ab,kw OR 'clinical trial'/exp OR random*:ti,ab,kw | **2293116** |
| **#2** | 'meta-analysis':ti,ab,kw OR 'meta analysis'/exp OR 'review'/exp OR review:ti,ab,kw | **3441138** |
| **#1** | ('obesity'/exp/mj AND 'surgery'/lnk OR 'bariatric surgery'/exp/mj OR 'roux y anastomosis'/exp/mj OR 'gastric bypass surgery'/exp/mj OR ((('bilio pancreatic' OR biliopancreatic) NEAR/3 (diversion* OR bypass*)):ti,kw) OR ((duodenal NEAR/3 switch*):ti,kw) OR (((stomach OR gastric) NEAR/3 (band* OR bypass* OR partiti* OR plicati* OR sleeve*)):ti,kw) OR ((sleeve NEAR/3 gastrectom*):ti,kw) OR ((roux NEAR/2 y):ti,kw) OR gastroplast*:ti,kw OR mgbp*:ti,kw OR oagb*:ti,kw OR rya:ti,kw OR rygb*:ti,kw OR sadis:ti,kw OR 'sadi s':ti,kw OR (((bariatric OR obes* OR 'weight loss' OR metabolic) NEAR/3 (surg* OR operati* OR procedure*)):ti,kw) OR sagb*:ti,kw) AND ('laparoscopy'/de OR 'laparoendoscopic single site surgery'/exp OR 'laparoscopic surgery'/exp OR 'minimally invasive procedure'/exp OR 'minimally invasive surgery'/exp OR laparoscop*:ti,ab,kw OR ((minimal* NEAR/3 invasive):ti,ab,kw) OR access:ti,ab,kw OR surg*:ti,ab,kw) OR 'laparoscopic sleeve gastrectomy'/exp OR lagb*:ti,ab,kw OR 'lap band*':ti,ab,kw OR lrygb*:ti,ab,kw | **40731** |

### T6Q3-4: Pregnancy (58 SRs, 4 RCTs, 76 cohort studies)

| **No.** | **Query** | **Results** |
| --- | --- | --- |
| **#13** | #10 AND #5 NOT (#11 OR #12) | **76** |
| **#12** | #10 AND #4 NOT #11 | **4** |
| **#11** | #10 AND #2 | **58** |
| **#10** | #9 NOT (('adolescent'/exp OR 'child'/exp OR adolescent*:ti,ab OR child*:ti,ab OR schoolchild*:ti,ab OR infant*:ti,ab OR girl*:ti,ab OR boy*:ti,ab OR teen:ti,ab OR teens:ti,ab OR teenager*:ti,ab OR youth*:ti,ab OR pediatr*:ti,ab OR paediatr*:ti,ab OR puber*:ti,ab) NOT ('adult'/exp OR 'aged'/exp OR 'middle aged'/exp OR adult*:ti,ab OR man:ti,ab OR men:ti,ab OR woman:ti,ab OR women:ti,ab)) | **192** |
| **#9** | #8 NOT ('conference abstract'/it OR 'conference paper'/it OR 'letter'/it OR 'note'/it) | **197** |
| **#8** | #7 NOT ([animals]/lim NOT [humans]/lim) AND [1-1-2004]/sd | **310** |
| **#7** | #1 AND #3 AND #6 | **324** |
| **#6** | 'pregnancy'/exp OR 'time to pregnancy'/exp OR pregnan*:ti,ab,kw | **941980** |
| **#5** | 'epidemiology'/de OR (((cohort OR case) NEAR/3 (control OR controll* OR comparison OR referent)):ti,ab,kw) OR risk:ti,ab,kw OR causation:ti,ab,kw OR causal:ti,ab,kw OR 'odds ratio':ti,ab,kw OR etiol*:ti,ab,kw OR aetiol*:ti,ab,kw OR 'natural history':ti,ab,kw OR outcome:ti,ab,kw OR course:ti,ab,kw OR retrospect*:ti,ab,kw OR 'follow up':ti,ab,kw OR followup:ti,ab,kw OR predict*:ti,ab,kw OR prognos*:ti,ab,kw | **7337087** |
| **#4** | 'clinical':ti,ab,kw AND 'trial':ti,ab,kw OR 'clinical trial'/exp OR random*:ti,ab,kw | **2293116** |
| **#3** | 'postoperative period'/exp OR 'postoperative complication'/exp OR postoperati*:ti,ab,kw OR 'post operati*':ti,ab,kw OR postsurg*:ti,ab,kw OR 'post surg*':ti,ab,kw | **1424169** |
| **#2** | 'meta-analysis':ti,ab,kw OR 'meta analysis'/exp OR 'review'/exp OR review:ti,ab,kw | **3441138** |
| **#1** | ('obesity'/exp/mj AND 'surgery'/lnk OR 'bariatric surgery'/exp/mj OR 'roux y anastomosis'/exp/mj OR 'gastric bypass surgery'/exp/mj OR ((('bilio pancreatic' OR biliopancreatic) NEAR/3 (diversion* OR bypass*)):ti,kw) OR ((duodenal NEAR/3 switch*):ti,kw) OR (((stomach OR gastric) NEAR/3 (band* OR bypass* OR partiti* OR plicati* OR sleeve*)):ti,kw) OR ((sleeve NEAR/3 gastrectom*):ti,kw) OR ((roux NEAR/2 y):ti,kw) OR gastroplast*:ti,kw OR mgbp*:ti,kw OR oagb*:ti,kw OR rya:ti,kw OR rygb*:ti,kw OR sadis:ti,kw OR 'sadi s':ti,kw OR (((bariatric OR obes* OR 'weight loss' OR metabolic) NEAR/3 (surg* OR operati* OR procedure*)):ti,kw) OR sagb*:ti,kw) AND ('laparoscopy'/de OR 'laparoendoscopic single site surgery'/exp OR 'laparoscopic surgery'/exp OR 'minimally invasive procedure'/exp OR 'minimally invasive surgery'/exp OR laparoscop*:ti,ab,kw OR ((minimal* NEAR/3 invasive):ti,ab,kw) OR access:ti,ab,kw OR surg*:ti,ab,kw) OR 'laparoscopic sleeve gastrectomy'/exp OR lagb*:ti,ab,kw OR 'lap band*':ti,ab,kw OR lrygb*:ti,ab,kw | **40731** |

### T6Q5: PPI (75 SRs, 33 RCTs, 127 cohort studies) (11 February 2019)

| **No.** | **Query** | **Results** |
| --- | --- | --- |
| **#13** | #10 AND #3 NOT (#11 OR #12) | **127** |
| **#12** | #10 AND #2 NOT #11 | **33** |
| **#11** | #1 AND #10 | **75** |
| **#10** | #9 NOT (('adolescent'/exp OR 'child'/exp OR adolescent*:ti,ab OR child*:ti,ab OR schoolchild*:ti,ab OR infant*:ti,ab OR girl*:ti,ab OR boy*:ti,ab OR teen:ti,ab OR teens:ti,ab OR teenager*:ti,ab OR youth*:ti,ab OR pediatr*:ti,ab OR paediatr*:ti,ab OR puber*:ti,ab) NOT ('adult'/exp OR 'aged'/exp OR 'middle aged'/exp OR adult*:ti,ab OR man:ti,ab OR men:ti,ab OR woman:ti,ab OR women:ti,ab)) | **332** |
| **#9** | #8 NOT ('conference abstract'/it OR 'conference paper'/it OR 'letter'/it OR 'note'/it) | **338** |
| **#8** | #7 NOT ([animals]/lim NOT [humans]/lim) AND [1-1-2004]/sd | **516** |
| **#7** | #4 AND #5 AND #6 | **530** |
| **#6** | 'postoperative period'/exp OR 'postoperative complication'/exp OR postoperati*:ti,ab,kw OR 'post operati*':ti,ab,kw OR postsurg*:ti,ab,kw OR 'post surg*':ti,ab,kw | **1440984** |
| **#5** | 'proton pump inhibitor'/exp OR 'proton pump inhibit*':ti,ab,kw OR ppi:ti,ab,kw OR ppis:ti,ab,kw OR dexlansoprazol*:ti,ab,kw OR esomeprazol*:ti,ab,kw OR nexium:ti,ab,kw OR lansoprazol*:ti,ab,kw OR omeprazol*:ti,ab,kw OR prilosec:ti,ab,kw OR pantoprazol*:ti,ab,kw OR protonix:ti,ab,kw OR rabeprazol*:ti,ab,kw OR dexrabeprazol*:ti,ab,kw OR aciphex:ti,ab,kw OR ogastro:ti,ab,kw OR agopton:ti,ab,kw OR bamalite:ti,ab,kw OR lansol:ti,ab,kw OR lanzor:ti,ab,kw OR monolitum:ti,ab,kw OR opiren:ti,ab,kw OR prevacid:ti,ab,kw OR 'pro ulco':ti,ab,kw OR promeco:ti,ab,kw OR takepron:ti,ab,kw OR ulpax:ti,ab,kw OR zoton:ti,ab,kw OR ogast:ti,ab,kw OR prezal:ti,ab,kw | **89199** |
| **#4** | ('obesity'/exp/mj AND 'surgery'/lnk OR 'bariatric surgery'/exp/mj OR 'roux y anastomosis'/exp/mj OR 'gastric bypass surgery'/exp/mj OR ((('bilio pancreatic' OR biliopancreatic) NEAR/3 (diversion* OR bypass*)):ti,kw) OR ((duodenal NEAR/3 switch*):ti,kw) OR (((stomach OR gastric) NEAR/3 (band* OR bypass* OR partiti* OR plicati* OR sleeve*)):ti,kw) OR ((sleeve NEAR/3 gastrectom*):ti,kw) OR ((roux NEAR/2 y):ti,kw) OR gastroplast*:ti,kw OR mgbp*:ti,kw OR oagb*:ti,kw OR rya:ti,kw OR rygb*:ti,kw OR sadis:ti,kw OR 'sadi s':ti,kw OR (((bariatric OR obes* OR 'weight loss' OR metabolic) NEAR/3 (surg* OR operati* OR procedure*)):ti,kw) OR sagb*:ti,kw) AND ('laparoscopy'/de OR 'laparoendoscopic single site surgery'/exp OR 'laparoscopic surgery'/exp OR 'minimally invasive procedure'/exp OR 'minimally invasive surgery'/exp OR laparoscop*:ti,ab,kw OR ((minimal* NEAR/3 invasive):ti,ab,kw) OR access:ti,ab,kw OR surg*:ti,ab,kw) OR 'laparoscopic sleeve gastrectomy'/exp OR lagb*:ti,ab,kw OR 'lap band*':ti,ab,kw OR lrygb*:ti,ab,kw | **41336** |
| **#3** | 'epidemiology'/de OR (((cohort OR case) NEAR/3 (control OR controll* OR comparison OR referent)):ti,ab,kw) OR risk:ti,ab,kw OR causation:ti,ab,kw OR causal:ti,ab,kw OR 'odds ratio':ti,ab,kw OR etiol*:ti,ab,kw OR aetiol*:ti,ab,kw OR 'natural history':ti,ab,kw OR outcome:ti,ab,kw OR course:ti,ab,kw OR retrospect*:ti,ab,kw OR 'follow up':ti,ab,kw OR followup:ti,ab,kw OR predict*:ti,ab,kw OR prognos*:ti,ab,kw | **7449307** |
| **#2** | 'clinical':ti,ab,kw AND 'trial':ti,ab,kw OR 'clinical trial'/exp OR random*:ti,ab,kw | **2325077** |
| **#1** | 'meta-analysis':ti,ab,kw OR 'meta analysis'/exp OR 'review'/exp OR review:ti,ab,kw | **3483264** |

### T6Q6: Postoperative control schedule (33 SRs, 86 RCTs, 104 cohort studies) (20 February 2019)

| **No.** | **Query** | **Results** |
| --- | --- | --- |
| **#13** | #7 AND #11 NOT (#12 OR #9) | **104** |
| **#12** | #7 AND #10 NOT #9 | **86** |
| **#11** | 'epidemiology'/de OR (((cohort OR case) NEAR/3 (control OR controll* OR comparison OR referent)):ti,ab,kw) OR risk:ti,ab,kw OR causation:ti,ab,kw OR causal:ti,ab,kw OR 'odds ratio':ti,ab,kw OR etiol*:ti,ab,kw OR aetiol*:ti,ab,kw OR 'natural history':ti,ab,kw OR outcome:ti,ab,kw OR course:ti,ab,kw OR retrospect*:ti,ab,kw OR 'follow up':ti,ab,kw OR followup:ti,ab,kw OR predict*:ti,ab,kw OR prognos*:ti,ab,kw | **7464354** |
| **#10** | 'clinical':ti,ab,kw AND 'trial':ti,ab,kw OR 'clinical trial'/exp OR random*:ti,ab,kw | **2329167** |
| **#9** | #7 AND #8 | **33** |
| **#8** | 'meta-analysis':ti,ab,kw OR 'meta analysis'/exp OR 'review'/exp OR review:ti,ab,kw | **3489659** |
| **#7** | #6 NOT (('adolescent'/exp OR 'child'/exp OR adolescent*:ti,ab OR child*:ti,ab OR schoolchild*:ti,ab OR infant*:ti,ab OR girl*:ti,ab OR boy*:ti,ab OR teen:ti,ab OR teens:ti,ab OR teenager*:ti,ab OR youth*:ti,ab OR pediatr*:ti,ab OR paediatr*:ti,ab OR puber*:ti,ab) NOT ('adult'/exp OR 'aged'/exp OR 'middle aged'/exp OR adult*:ti,ab OR man:ti,ab OR men:ti,ab OR woman:ti,ab OR women:ti,ab)) | **280** |
| **#6** | #5 NOT ('conference abstract'/it OR 'conference paper'/it OR 'letter'/it OR 'note'/it) | **286** |
| **#5** | #4 NOT ([animals]/lim NOT [humans]/lim) AND [1-1-2004]/sd | **530** |
| **#4** | #1 AND #2 AND #3 | **570** |
| **#3** | 'postoperative period'/exp OR 'postoperative complication'/exp OR postoperati*:ti,ab,kw OR 'post operati*':ti,ab,kw OR postsurg*:ti,ab,kw OR 'post surg*':ti,ab,kw OR 'after surg*':ti,ab,kw | **1551212** |
| **#2** | (intens*:ti,ab,kw OR schedul*:ti,ab,kw) AND ('patient monitoring'/exp OR 'physiologic monitoring'/exp OR 'patient scheduling'/exp OR monitor*:ti,ab,kw OR 'check up*':ti,ab,kw OR checkup*:ti,ab,kw OR 'office visit*':ti,ab,kw OR schedul*:ti,ab,kw OR control:ti,ab,kw) | **400955** |
| **#1** | ('obesity'/exp/mj AND 'surgery'/lnk OR 'bariatric surgery'/exp/mj OR 'roux y anastomosis'/exp/mj OR 'gastric bypass surgery'/exp/mj OR ((('bilio pancreatic' OR biliopancreatic) NEAR/3 (diversion* OR bypass*)):ti,kw) OR ((duodenal NEAR/3 switch*):ti,kw) OR (((stomach OR gastric) NEAR/3 (band* OR bypass* OR partiti* OR plicati* OR sleeve*)):ti,kw) OR ((sleeve NEAR/3 gastrectom*):ti,kw) OR ((roux NEAR/2 y):ti,kw) OR gastroplast*:ti,kw OR mgbp*:ti,kw OR oagb*:ti,kw OR rya:ti,kw OR rygb*:ti,kw OR sadis:ti,kw OR 'sadi s':ti,kw OR (((bariatric OR obes* OR 'weight loss' OR metabolic) NEAR/3 (surg* OR operati* OR procedure*)):ti,kw) OR sagb*:ti,kw) AND ('laparoscopy'/de OR 'laparoendoscopic single site surgery'/exp OR 'laparoscopic surgery'/exp OR 'minimally invasive procedure'/exp OR 'minimally invasive surgery'/exp OR laparoscop*:ti,ab,kw OR ((minimal* NEAR/3 invasive):ti,ab,kw) OR access:ti,ab,kw OR surg*:ti,ab,kw) OR 'laparoscopic sleeve gastrectomy'/exp OR lagb*:ti,ab,kw OR 'lap band*':ti,ab,kw OR lrygb*:ti,ab,kw | **41445** |

### T6Q7: Sequential diet regimen (45 SRs, 47 RCTs, 57 cohort studies) (20 February 2019)

| **No.** | **Query** | **Results** |
| --- | --- | --- |
| **#15** | #11 AND #4 NOT (#13 OR #14) | **57** |
| **#14** | #11 AND #3 NOT #13 | **47** |
| **#13** | #11 AND #12 | **45** |
| **#12** | 'meta-analysis':ti,ab,kw OR 'meta analysis'/exp OR 'review'/exp OR review:ti,ab,kw | **3489659** |
| **#11** | #10 NOT (('adolescent'/exp OR 'child'/exp OR adolescent*:ti,ab OR child*:ti,ab OR schoolchild*:ti,ab OR infant*:ti,ab OR girl*:ti,ab OR boy*:ti,ab OR teen:ti,ab OR teens:ti,ab OR teenager*:ti,ab OR youth*:ti,ab OR pediatr*:ti,ab OR paediatr*:ti,ab OR puber*:ti,ab) NOT ('adult'/exp OR 'aged'/exp OR 'middle aged'/exp OR adult*:ti,ab OR man:ti,ab OR men:ti,ab OR woman:ti,ab OR women:ti,ab)) | **188** |
| **#10** | #9 NOT ('conference abstract'/it OR 'conference paper'/it OR 'letter'/it OR 'note'/it) | **192** |
| **#9** | #8 NOT ([animals]/lim NOT [humans]/lim) AND [1-1-2004]/sd | **271** |
| **#8** | #1 AND #2 AND #7 | **321** |
| **#7** | 'diet therapy'/exp/mj OR (((diet OR diets OR dietary OR calor*) NEAR/2 (regimen* OR restrict* OR 'low carb*' OR reduc* OR gluten OR fat OR fats OR carbohydrat* OR loading OR 'low calor*' OR protein OR proteins OR 'south beach' OR atkins OR ketogen* OR sequenti* OR 'weight loss' OR modificat*)):ti,kw) | **129183** |
| **#4** | 'epidemiology'/de OR (((cohort OR case) NEAR/3 (control OR controll* OR comparison OR referent)):ti,ab,kw) OR risk:ti,ab,kw OR causation:ti,ab,kw OR causal:ti,ab,kw OR 'odds ratio':ti,ab,kw OR etiol*:ti,ab,kw OR aetiol*:ti,ab,kw OR 'natural history':ti,ab,kw OR outcome:ti,ab,kw OR course:ti,ab,kw OR retrospect*:ti,ab,kw OR 'follow up':ti,ab,kw OR followup:ti,ab,kw OR predict*:ti,ab,kw OR prognos*:ti,ab,kw | **7464354** |
| **#3** | 'clinical':ti,ab,kw AND 'trial':ti,ab,kw OR 'clinical trial'/exp OR random*:ti,ab,kw | **2329167** |
| **#2** | 'postoperative period'/exp OR 'postoperative complication'/exp OR postoperati*:ti,ab,kw OR 'post operati*':ti,ab,kw OR postsurg*:ti,ab,kw OR 'post surg*':ti,ab,kw OR 'after surg*':ti,ab,kw | **1551212** |
| **#1** | ('obesity'/exp/mj AND 'surgery'/lnk OR 'bariatric surgery'/exp/mj OR 'roux y anastomosis'/exp/mj OR 'gastric bypass surgery'/exp/mj OR ((('bilio pancreatic' OR biliopancreatic) NEAR/3 (diversion* OR bypass*)):ti,kw) OR ((duodenal NEAR/3 switch*):ti,kw) OR (((stomach OR gastric) NEAR/3 (band* OR bypass* OR partiti* OR plicati* OR sleeve*)):ti,kw) OR ((sleeve NEAR/3 gastrectom*):ti,kw) OR ((roux NEAR/2 y):ti,kw) OR gastroplast*:ti,kw OR mgbp*:ti,kw OR oagb*:ti,kw OR rya:ti,kw OR rygb*:ti,kw OR sadis:ti,kw OR 'sadi s':ti,kw OR (((bariatric OR obes* OR 'weight loss' OR metabolic) NEAR/3 (surg* OR operati* OR procedure*)):ti,kw) OR sagb*:ti,kw) AND ('laparoscopy'/de OR 'laparoendoscopic single site surgery'/exp OR 'laparoscopic surgery'/exp OR 'minimally invasive procedure'/exp OR 'minimally invasive surgery'/exp OR laparoscop*:ti,ab,kw OR ((minimal* NEAR/3 invasive):ti,ab,kw) OR access:ti,ab,kw OR surg*:ti,ab,kw) OR 'laparoscopic sleeve gastrectomy'/exp OR lagb*:ti,ab,kw OR 'lap band*':ti,ab,kw OR lrygb*:ti,ab,kw | **41445** |

# Topic 7: Investigational procedures

## Embase.com (23 November 2018)

### T7: All questions and all results

| **No.** | **Query** | **Results** | **Question** |
| --- | --- | --- | --- |
| **#41** | #40 NOT ([animals]/lim NOT [humans]/lim) AND [1-1-2004]/sd | **37** | **18** |
| **#40** | 'gastric contractility modulat*':ti,ab,kw OR 'tantalus':ti,ab,kw | **77** |  |
| **#39** | #38 NOT ([animals]/lim NOT [humans]/lim) AND [1-1-2004]/sd | **1** | **17** |
| **#38** | 'selective vagal nerve stimulat*':ti,ab,kw OR 'transcend implantable gastric stimulat*':ti,ab,kw | **9** |  |
| **#37** | 'enterra system*':ti,ab,kw | **13** | **16** |
| **#36** | 'abiliti system*':ti,ab,kw | **11** | **15** |
| **#35** | #33 AND #34 | **23** | **14** |
| **#34** | 'obesity'/exp/mj AND 'surgery'/lnk OR 'bariatric surgery'/exp/mj OR 'roux y anastomosis'/exp/mj OR 'gastric bypass surgery'/exp/mj OR ((('bilio pancreatic' OR biliopancreatic) NEAR/3 (diversion* OR bypass*)):ti,kw) OR ((duodenal NEAR/3 switch*):ti,kw) OR (((stomach OR gastric) NEAR/3 (band* OR bypass* OR partiti* OR plicati* OR sleeve*)):ti,kw) OR ((sleeve NEAR/3 gastrectom*):ti,kw) OR ((roux NEAR/2 y):ti,kw) OR gastroplast*:ti,kw OR mgbp*:ti,kw OR oagb*:ti,kw OR rya:ti,kw OR rygb*:ti,kw OR sadis:ti,kw OR 'sadi s':ti,kw OR (((bariatric OR obes* OR 'weight loss' OR metabolic) NEAR/3 (surg* OR operati* OR procedure*)):ti,kw) OR sagb*:ti,kw OR 'laparoscopic sleeve gastrectomy'/exp OR lagb*:ti,ab,kw OR 'lap band*':ti,ab,kw OR lrygb*:ti,ab,kw | **48129** |  |
| **#33** | 'gastric electrical stimulat*':ti,ab,kw | **649** |  |
| **#31** | #30 AND #5 | **39** | **13** |
| **#30** | ((pacing NEAR/3 vagal):ti,ab,kw) AND nerve*:ti,ab,kw OR 'vagal nerve block*':ti,ab,kw OR 'vbloc*':ti,ab,kw OR 'maestro rechargeable system*':ti,ab,kw | **73** |  |
| **#29** | #28 AND #5 | **17** | **12** |
| **#28** | 'endoscopic aspiration therap*':ti,ab,kw OR 'aspireassist':ti,ab,kw OR 'aspire assist':ti,ab,kw OR 'aspiration therapy system*':ti,ab,kw | **26** |  |
| **#27** | 'circular endoscopic stapl*':ti,ab,kw | **6** | **11** |
| **#26** | 'articular circular endoscopic stapl*':ti,ab,kw | **0** |  |
| **#25** | 'self assembling magnet*':ti,ab,kw OR 'selfassembling magnet*':ti,ab,kw | **17** | **10** |
| **#24** | #23 AND #5 | **22** | **9** |
| **#23** | 'duodenal mucosal resurfac*':ti,ab,kw | **30** |  |
| **#22** | 'transoral anterior-to-posterior greater curvature plication':ti,ab,kw OR 'endomina':ti,ab,kw | **7** | **8** |
| **#21** | #20 NOT (('adolescent'/exp OR 'child'/exp OR adolescent*:ti,ab OR child*:ti,ab OR schoolchild*:ti,ab OR infant*:ti,ab OR girl*:ti,ab OR boy*:ti,ab OR teen:ti,ab OR teens:ti,ab OR teenager*:ti,ab OR youth*:ti,ab OR pediatr*:ti,ab OR paediatr*:ti,ab OR puber*:ti,ab) NOT ('adult'/exp OR 'aged'/exp OR 'middle aged'/exp OR adult*:ti,ab OR man:ti,ab OR men:ti,ab OR woman:ti,ab OR women:ti,ab)) | **48** | **7** |
| **#20** | #19 NOT ('conference abstract'/it OR 'conference paper'/it OR 'letter'/it OR 'note'/it) | **48** |  |
| **#19** | #18 NOT ([animals]/lim NOT [humans]/lim) AND [1-1-2004]/sd | **165** |  |
| **#18** | #17 AND #5 | **169** |  |
| **#17** | 'endoscopic sleeve gastroplast*':ti,ab,kw OR 'overstitch':ti,ab,kw | **237** |  |
| **#16** | 'transpyloric shuttle*':ti,ab,kw OR 'trans pyloric shuttle*':ti,ab,kw | **6** | **6** |
| **#15** | 'sense bariatric device*':ti,ab,kw | **0** | **5** |
| **#14** | 'full sense bariatric device*':ti,ab,kw | **0** | **5** |
| **#13** | #12 AND #5 | **35** | **4** |
| **#12** | 'primary obesity surgery endoluminal*':ti,ab,kw OR 'incisionless operating platform*':ti,ab,kw | **39** |  |
| **#11** | 'transoral endoscopic vertical gastroplast*':ti,ab,kw OR 'toga system*':ti,ab,kw | **7** | **3** |
| **#10** | valentx:ti,ab,kw | **7** | **2** |
| **#9** | #8 NOT (('adolescent'/exp OR 'child'/exp OR adolescent*:ti,ab OR child*:ti,ab OR schoolchild*:ti,ab OR infant*:ti,ab OR girl*:ti,ab OR boy*:ti,ab OR teen:ti,ab OR teens:ti,ab OR teenager*:ti,ab OR youth*:ti,ab OR pediatr*:ti,ab OR paediatr*:ti,ab OR puber*:ti,ab) NOT ('adult'/exp OR 'aged'/exp OR 'middle aged'/exp OR adult*:ti,ab OR man:ti,ab OR men:ti,ab OR woman:ti,ab OR women:ti,ab)) | **73** | **1** |
| **#8** | #7 NOT ('conference abstract'/it OR 'conference paper'/it OR 'letter'/it OR 'note'/it) | **75** |  |
| **#7** | #6 NOT ([animals]/lim NOT [humans]/lim) AND [1-1-2004]/sd | **179** |  |
| **#6** | #1 AND #5 | **182** |  |
| **#5** | #2 OR #3 OR #4 | **10725194** |  |
| **#4** | 'epidemiology'/de OR (((cohort OR case) NEAR/3 (control OR controll* OR comparison OR referent)):ti,ab,kw) OR risk:ti,ab,kw OR causation:ti,ab,kw OR causal:ti,ab,kw OR 'odds ratio':ti,ab,kw OR etiol*:ti,ab,kw OR aetiol*:ti,ab,kw OR 'natural history':ti,ab,kw OR outcome:ti,ab,kw OR course:ti,ab,kw OR retrospect*:ti,ab,kw OR 'follow up':ti,ab,kw OR followup:ti,ab,kw OR predict*:ti,ab,kw OR prognos*:ti,ab,kw | **7337087** |  |
| **#3** | 'clinical':ti,ab,kw AND 'trial':ti,ab,kw OR 'clinical trial'/exp OR random*:ti,ab,kw | **2293116** |  |
| **#2** | 'meta-analysis':ti,ab,kw OR 'meta analysis'/exp OR 'review'/exp OR review:ti,ab,kw | **3441138** |  |
| **#1** | 'duodenal-jejunal bypass liner':ti,ab,kw OR 'duodenal-jejunal bypass sleeve':ti,ab,kw OR endobarrier:ti,ab,kw | **277** |  |
