# Supplementary material for: Clinical practice guidelines of the European Association for Endoscopic Surgery (EAES) on bariatric surgery: update 2020 endorsed by IFSO-EC, EASO and ESPCOP
Source: Surg Endosc. 2020 Apr 23;34(6):2332–58. doi: 10.1007/s00464-020-07555-y (PMC7214495; doi:10.1007/s00464-020-07555-y)

**Supplementary file 4**

**PRISMA FLOWCHARTS**

**Topic 1:** Bariatric surgery vs. non0surgical management

**Topic 2:** Preoperative workup


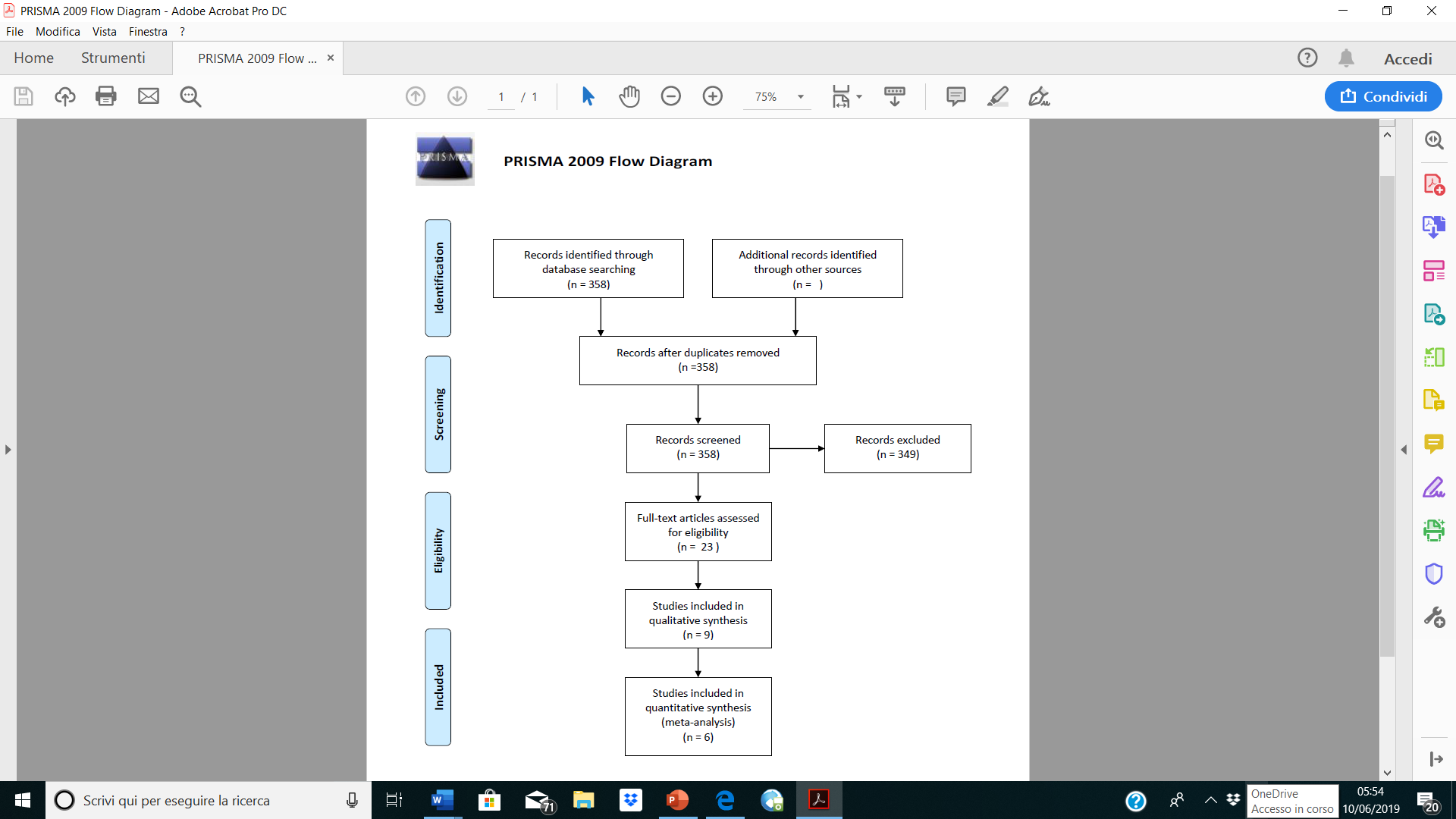


**Topic 3:** Perioperative management


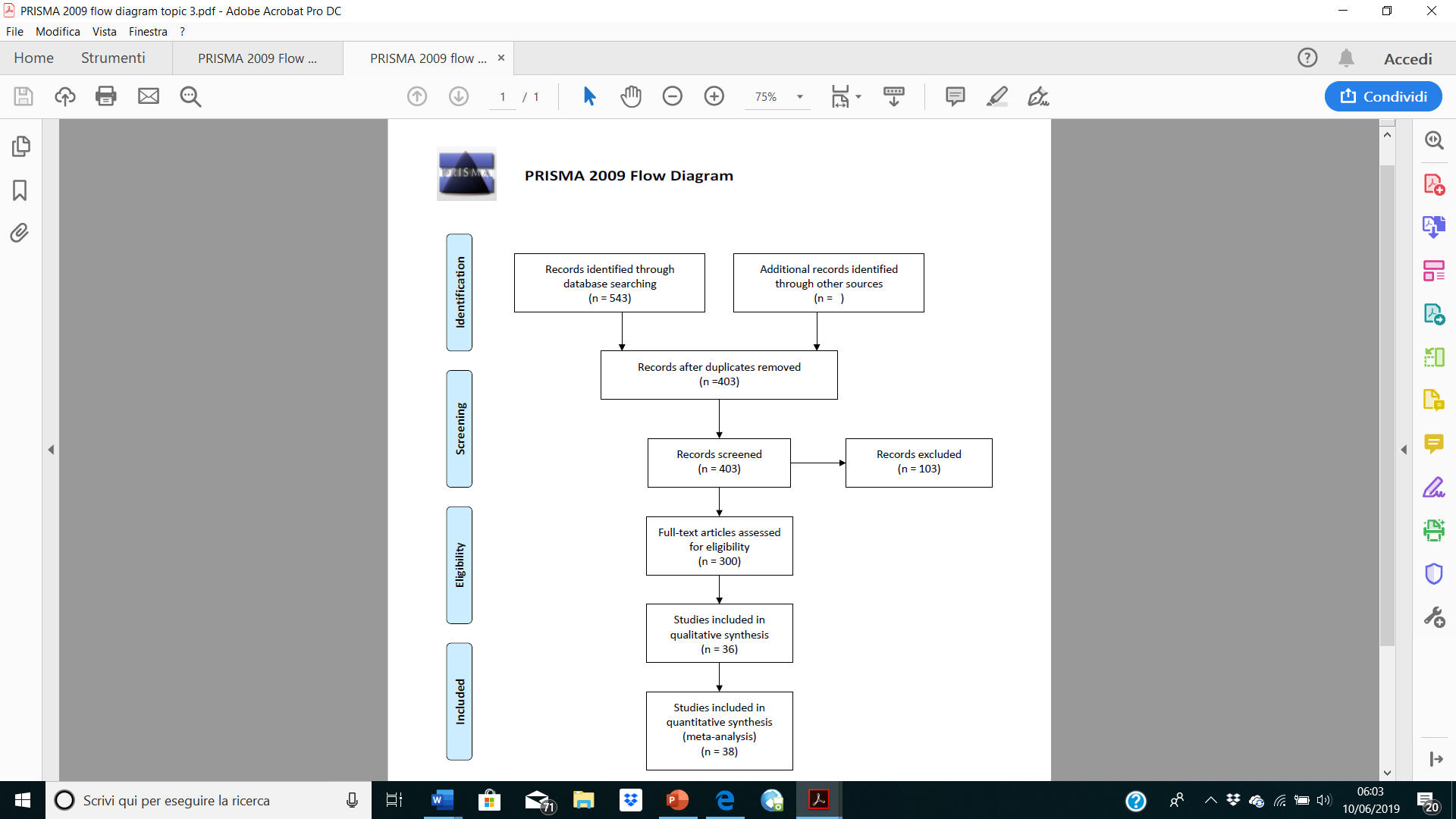


**Topic 4:** Adjustable gastric banding


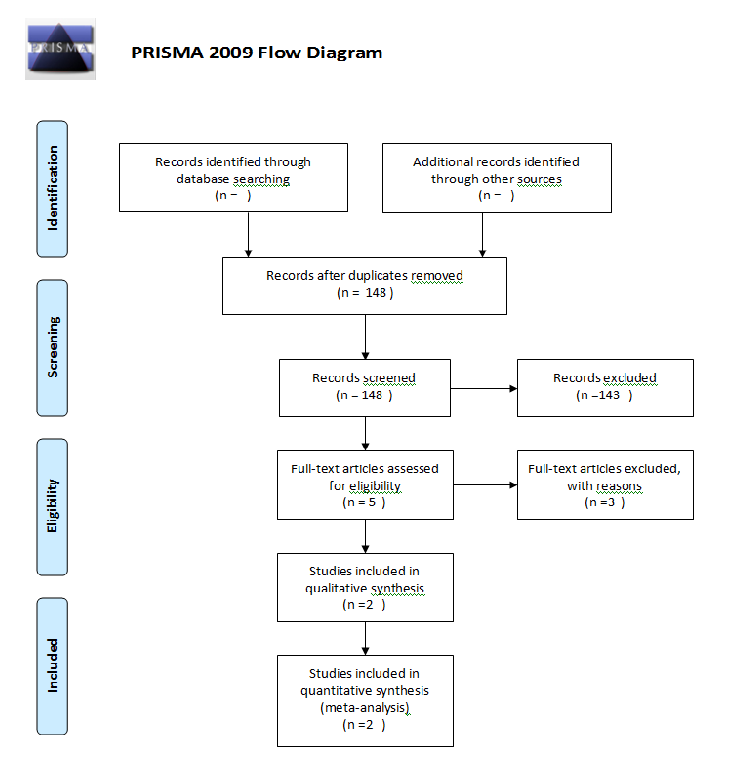


**Topic 4:** Sleeve gastrectomy


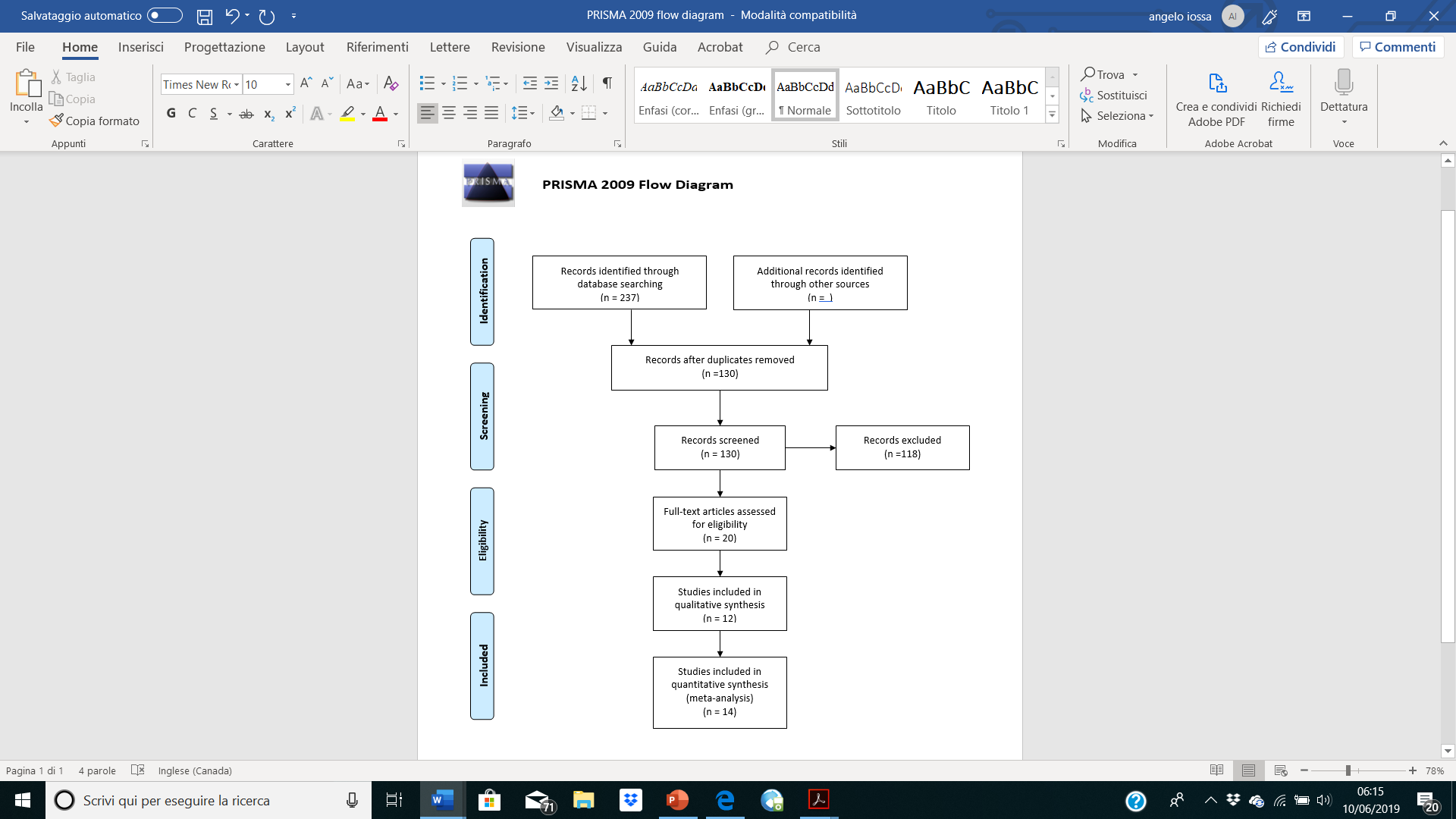


**Topic 4:** Roux-en-Y gastric bypass


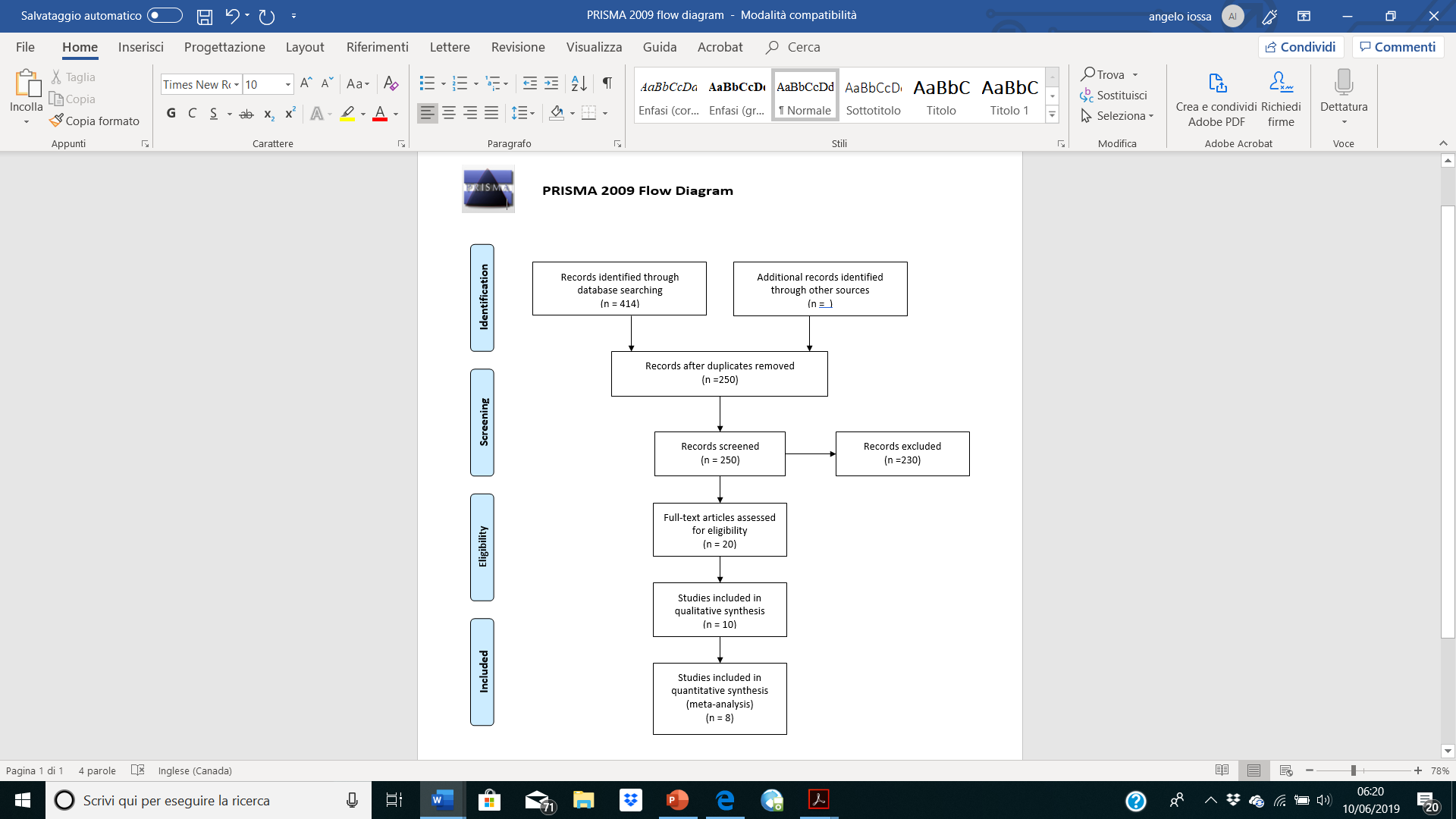


**Topic 4:** One anastomosis gastric bypass

**Topic 4:** Single-anastomosis duodeno-ileal switch

**Topic 4:** Biliopancreatic diversion with duodenal switch


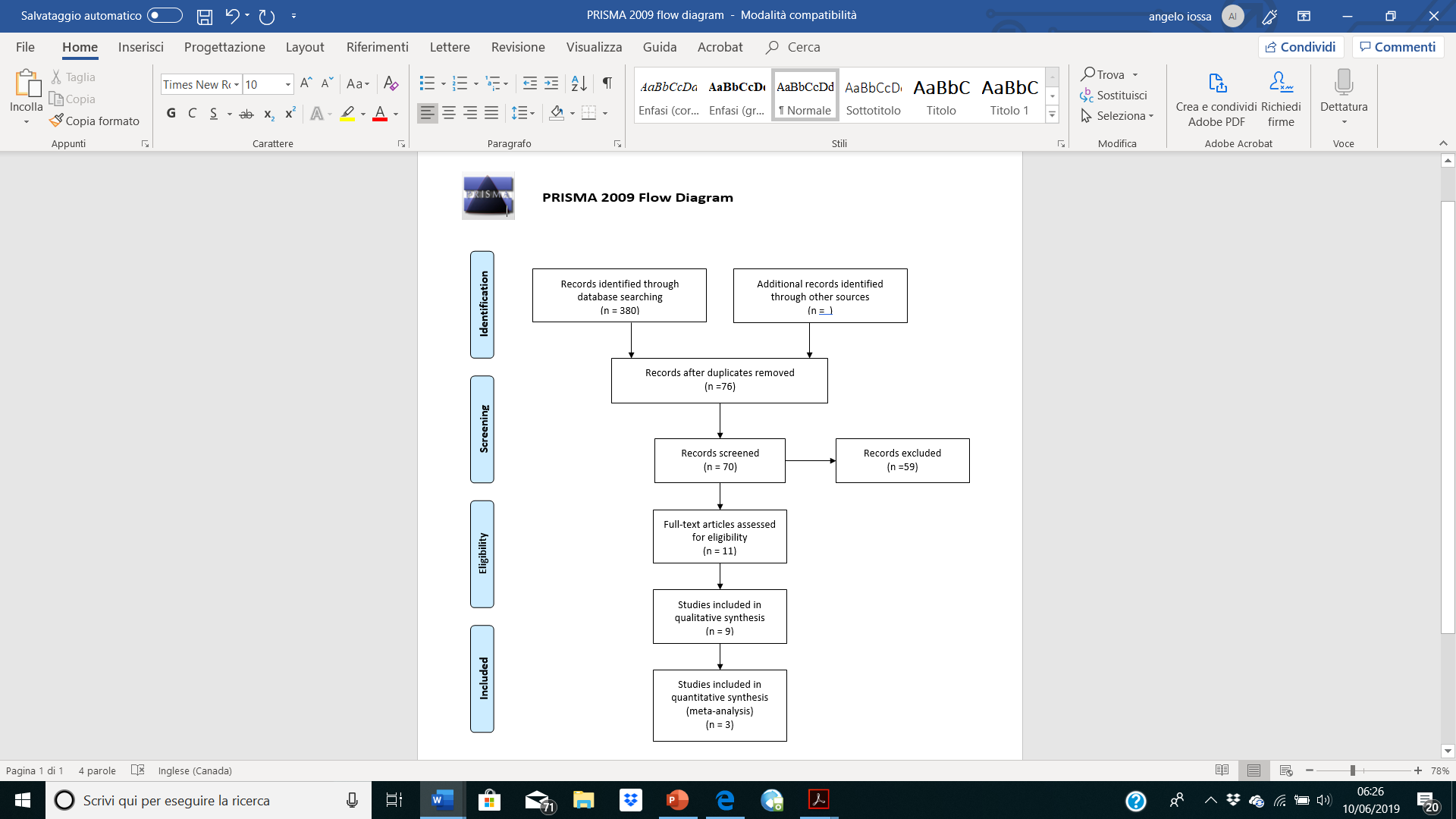


**Topic 5:** Revisional surgery


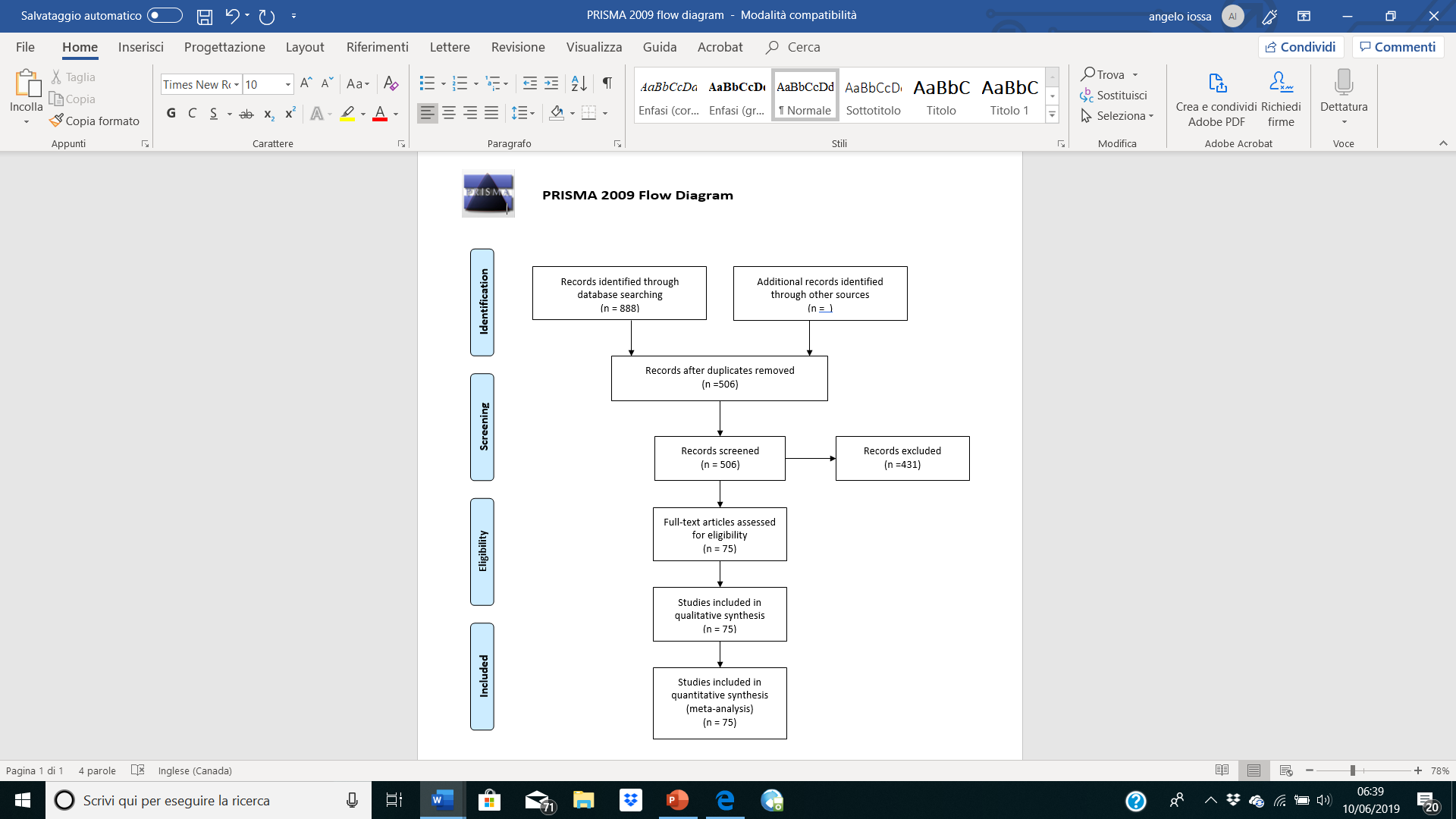


**Topic 6:** Postoperative care


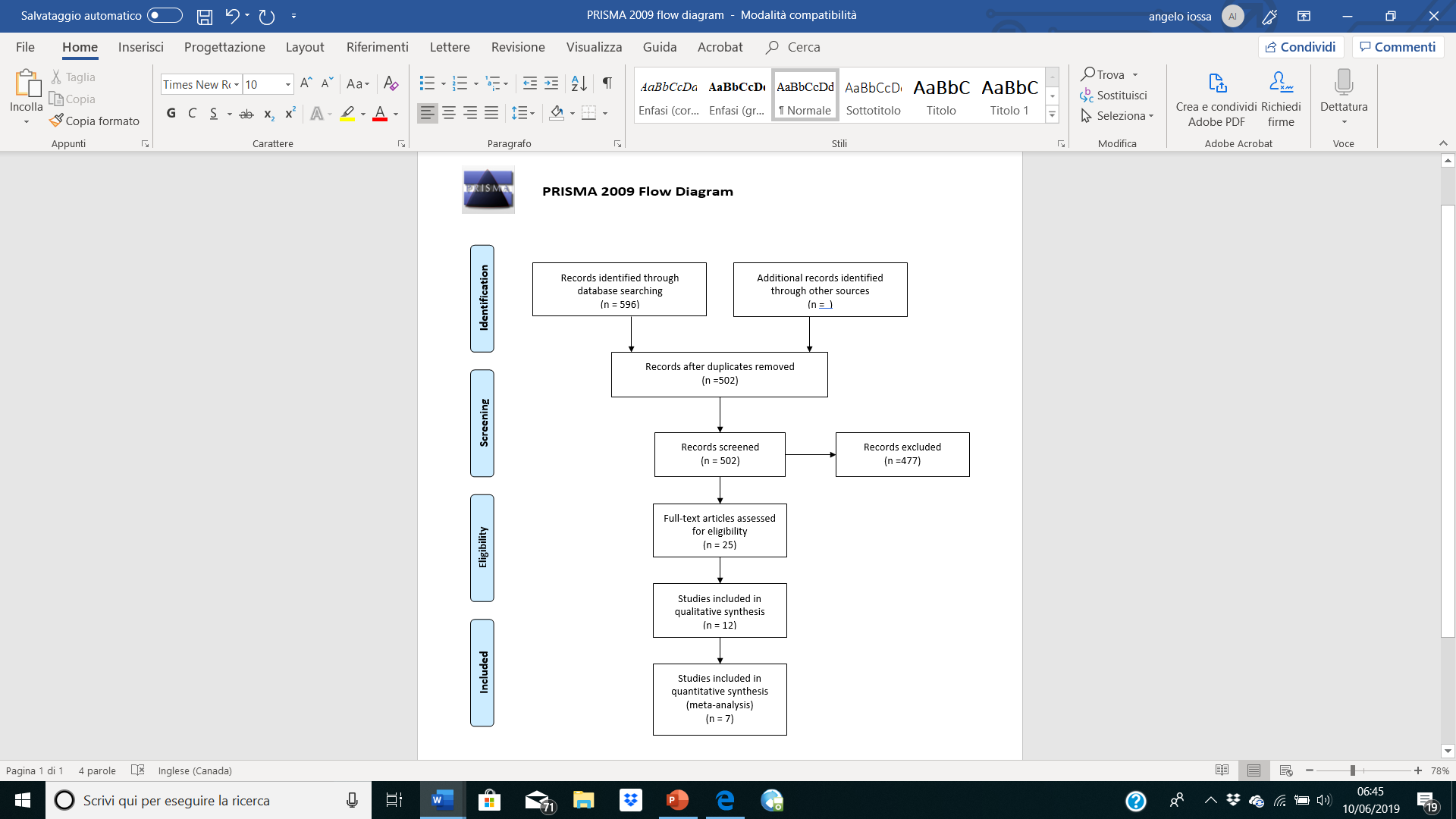


**Topic 7:** Investigational procedures


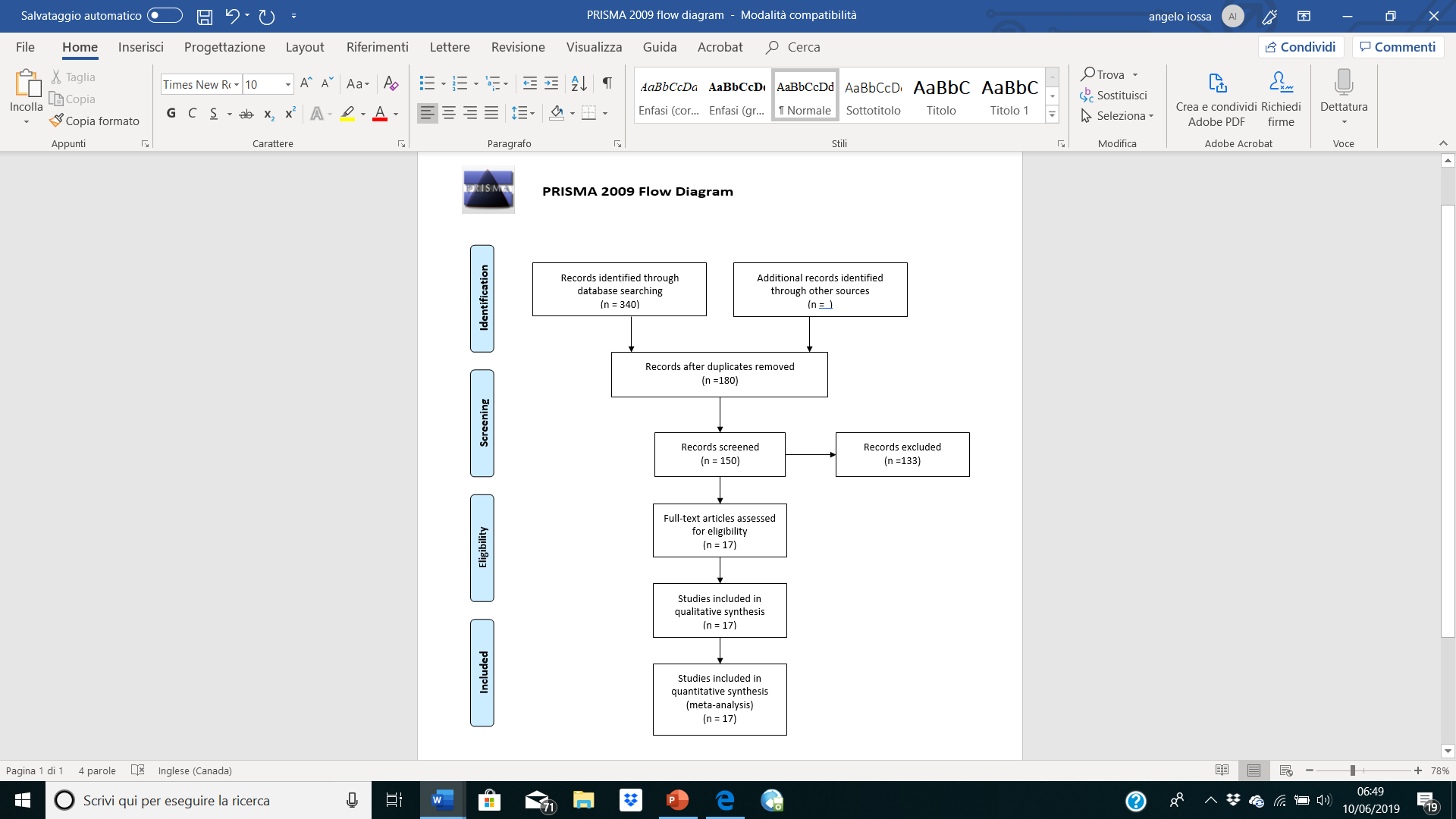

Supplement: Supplementary file 5 — Supplementary file5 (DOCX 1792 kb) [file 464_2020_7555_MOESM5_ESM.docx]
