# Supplementary material for: Clinical practice guidelines of the European Association for Endoscopic Surgery (EAES) on bariatric surgery: update 2020 endorsed by IFSO-EC, EASO and ESPCOP
Source: Surg Endosc. 2020 Apr 23;34(6):2332–58. doi: 10.1007/s00464-020-07555-y (PMC7214495; doi:10.1007/s00464-020-07555-y)

**Supplementary file 2**

**RESULTS OF ONLINE SURVEY**

**May 30 – June 12, 2019**

**Q1: Are you involved in the management of patients with obesity?**

Answered: 220 Skipped: 0


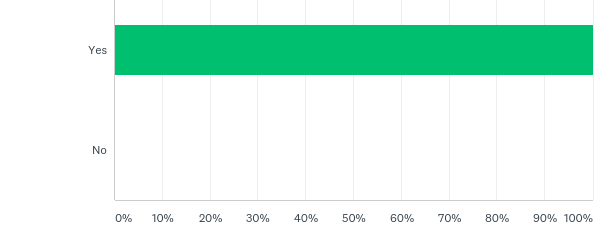


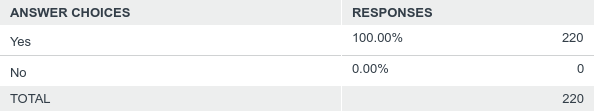


**Q2: You are**


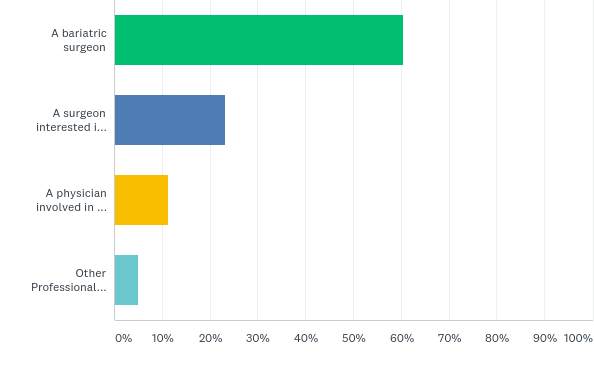
 Answered: 220 Skipped: 0

**
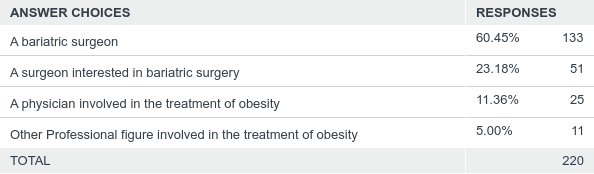
**

**Q3: Are you a member of any of the following Scientific Societies? Please select all that apply**

Answered: 220 Skipped: 0

**
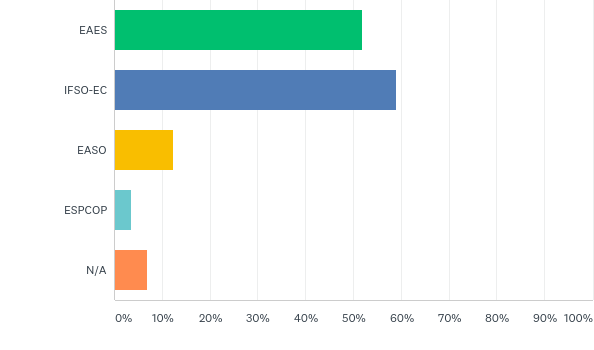
**

**
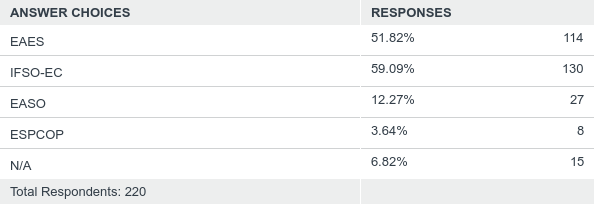
**

**Q4: On average, how many bariatric operations do you perform each year?**

Answered: 220 Skipped: 0

**
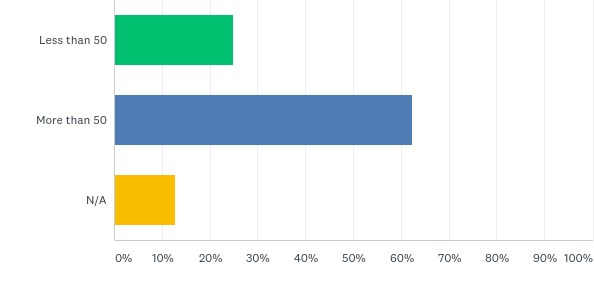

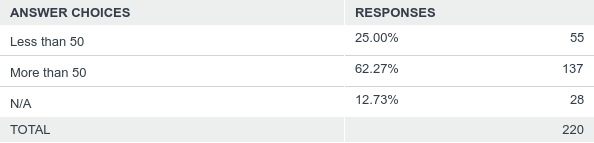
**

**Q5: Recommendation: "Laparoscopic bariatric surgery should be considered for patients with BMI >40 kg/m2 and for patients with BMI 35-40 kg/m2 and associated co-morbidities that are expected to improve after surgery.Laparoscopic bariatric/metabolic surgery should be considered for patients with BMI 30-35 kg/m2 and type 2 diabetes and/or arterial hypertension with poor control despite optimal medical therapy". Is this applicable to your practice?**

Answered: 220 Skipped: 0

**
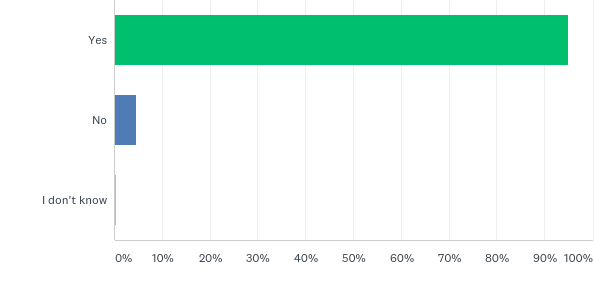

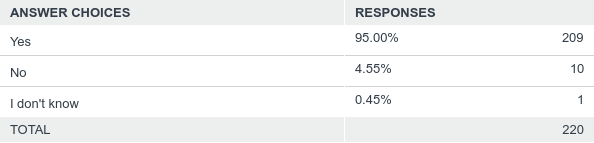
**

**Q6: Recommendation: "No recommendation can be made for either routine Helicobacter pylori eradication or no eradication prior to bariatric surgery, on the basis of available evidence". Is this applicable to your practice?**

Answered: 220 Skipped: 0

**
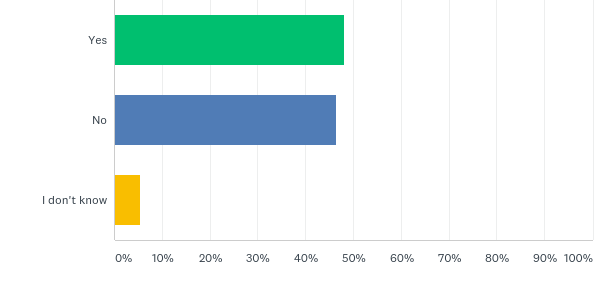

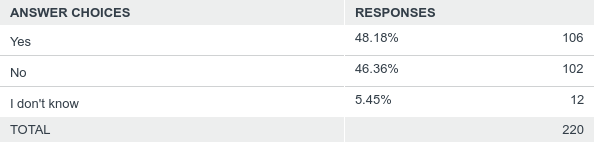
**

**Q7: Recommendation: "Preoperative Dietician consultation should be considered for patients undergoing laparoscopic bariatric surgery". Is this applicable to your practice?**

Answered: 220 Skipped: 0

**
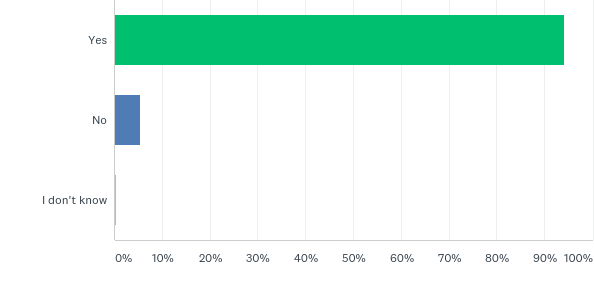
** **
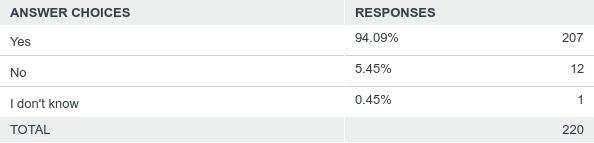
**

**Q8: Recommendation: "Esophagogastroscopy can be considered as routine diagnostic test prior to laparoscopic bariatric surgery.". Is this applicable to your practice?**

Answered: 220 Skipped: 0

**
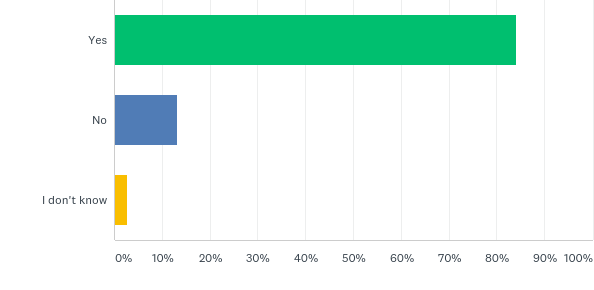
** **
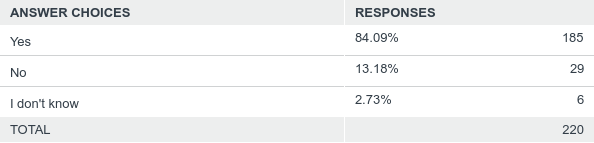
**

**Q9: Recommendation: "Psychological evaluation can be considered before bariatric surgery.The presence of a previous diagnosis of binge eating or depression may not be considered an absolute contraindication to surgery". Is this applicable to your practice?**

Answered: 220 Skipped: 0

**
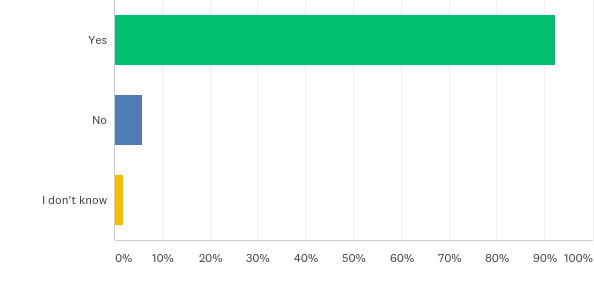
**

**
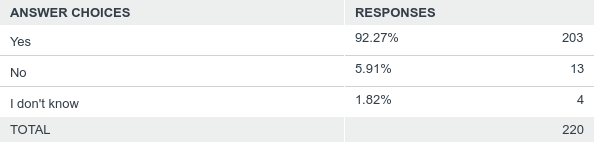
**

**Q10: Recommendation: "Screening for obstructive sleep apnea using the STOP-BANG criteria can be considered prior to bariatric surgery".Is this applicable to your practice?**

Answered: 219 Skipped: 1

**
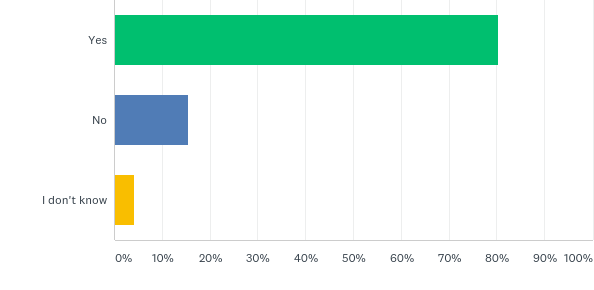
** **
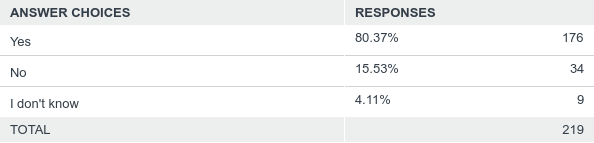
**

**Q11: Recommendation: "Perioperative CPAP should be considered in patients with severe obstructive sleep apnea syndrome which are candidates for bariatric surgery".Is this applicable to your practice?**

Answered: 220 Skipped: 0

**
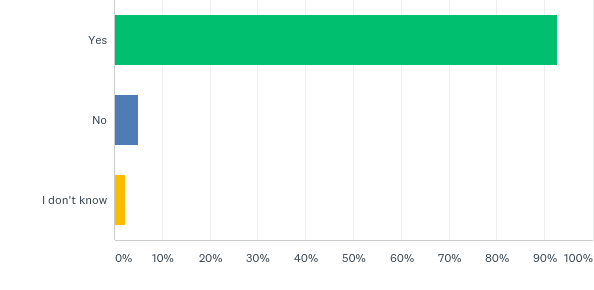
** **
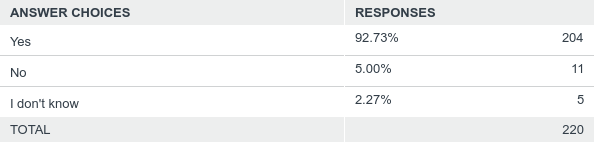
**

**Q12: Recommendation: "No recommendation can be made on the dose and duration of pharmacological prophylaxis in patients after bariatric surgery". Is this applicable to your practice?**

Answered: 220 Skipped: 0

**
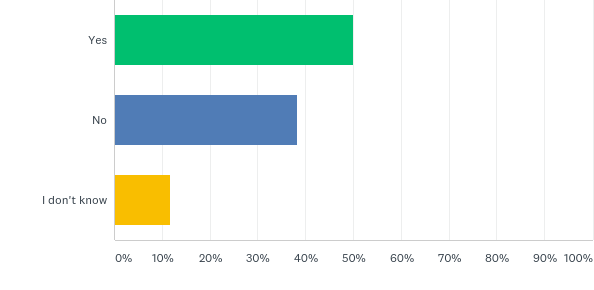
** **
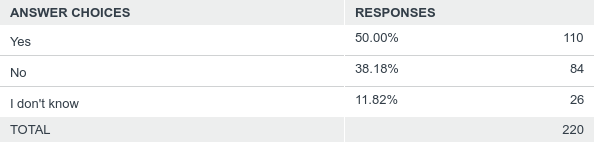
**

**Q13: Recommendation: "Inferior vena cava filter is not recommended in patients undergoing bariatric surgery".Is this applicable to your practice?**

Answered: 220 Skipped: 0**
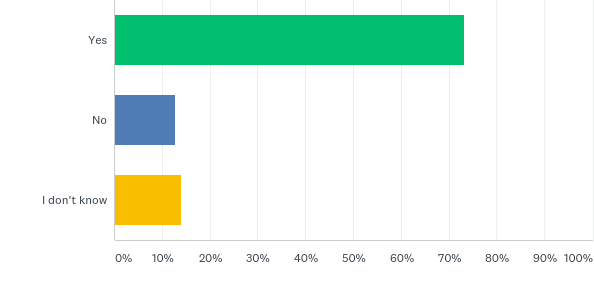
**
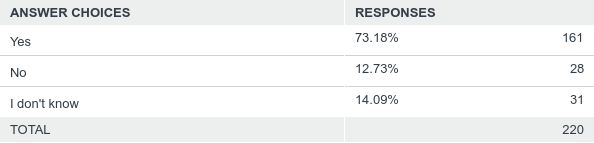


**Q14: Recommendation: "No recommendation for either an ERAS protocol or standard care can be made on the basis of available evidence". Is this applicable to your practice?**

Answered: 219 Skipped: 1

**
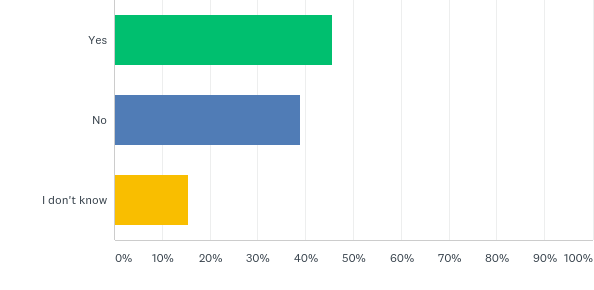
**
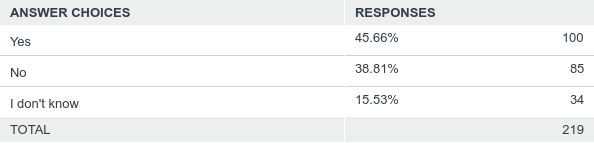


**Q15: Recommendation: "Perioperative multimodal analgesia with minimal opioid use may be considered in patients undergoing bariatric surgery". Is this applicable to your practice?**

Answered: 219 Skipped: 1

**
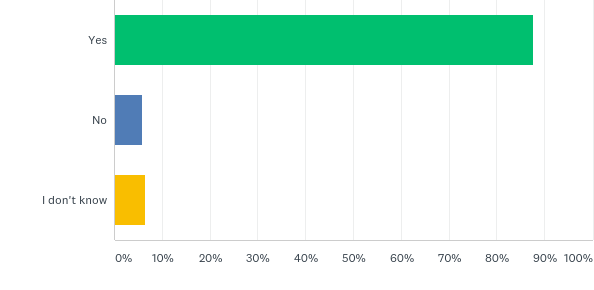
** **
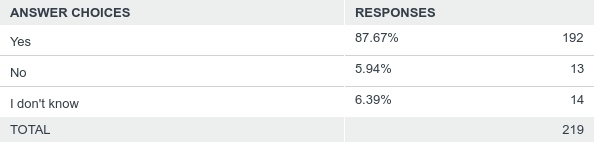
**

**Q16: Position statement: "Adjustable Gastric Banding is associated with a high rate of reoperations for complications or conversion to another bariatric procedure for insufficient weight loss in the long term". Is this applicable to your practice?**

Answered: 220 Skipped: 0


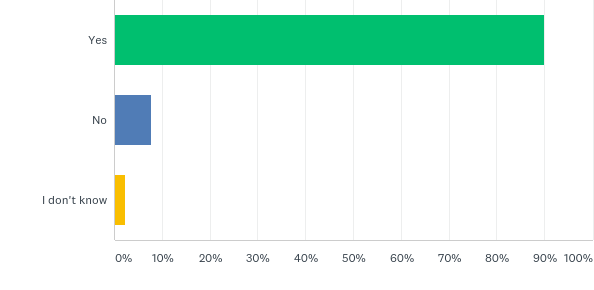

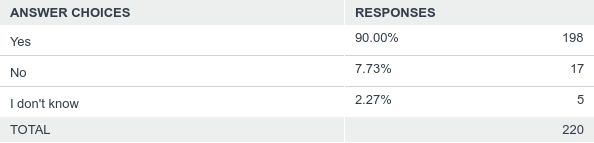


**Q17: Recommendation: "Sleeve gastrectomy may be preferred over Adjustable Gastric Banding for weight loss and control or resolution of metabolic co-morbidities". Is this applicable to your practice?**

Answered: 220 Skipped: 0


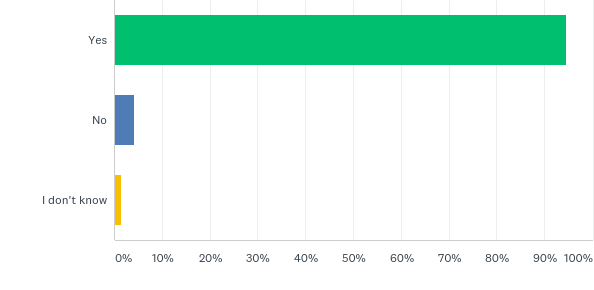

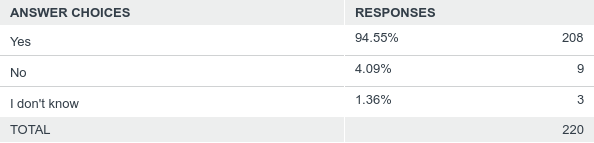


**Q18: Position statement: "Sleeve gastrectomy may offer improved short-term weight loss and resolution of type 2 diabetes compared to gastric plication. No significant differences are observed at mid-term. Long-term comparative data on weight-loss and metabolic effects are, however, lacking". Is this applicable to your practice?**

Answered: 220 Skipped: 0


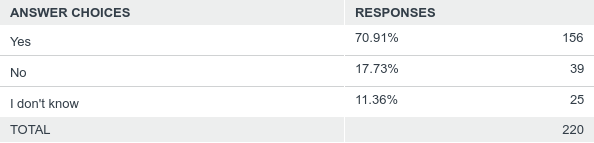

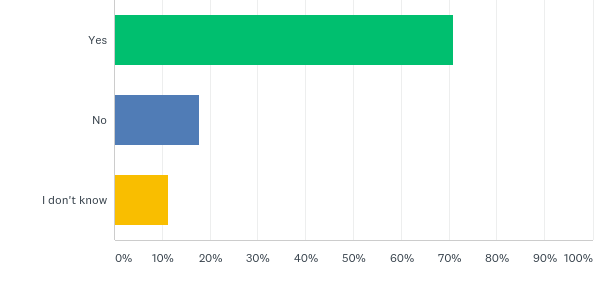


**Q19: Position statement: "More extensive antral resection (2-3 cm from the pylorus versus >5cm antral preservation) seems to offer larger weight loss at short term without significant increase of the risk for post-operative complications but we are very uncertain. Long term data are, hovewer, lacking". Is this applicable to your practice?**

Answered: 220 Skipped: 0


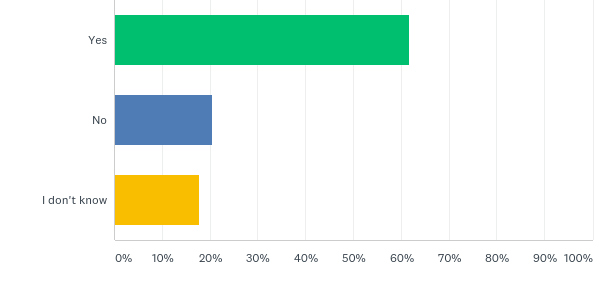

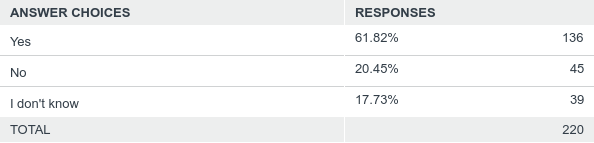


**Q20: Recommendation: "A bougie size smaller than 36F compared to a bougie size 36F or more may be recommended for calibration in sleeve gastrectomy, as it is associated with larger weight loss in the mid-term". Is this applicable to your practice?**

Answered: 220 Skipped: 0


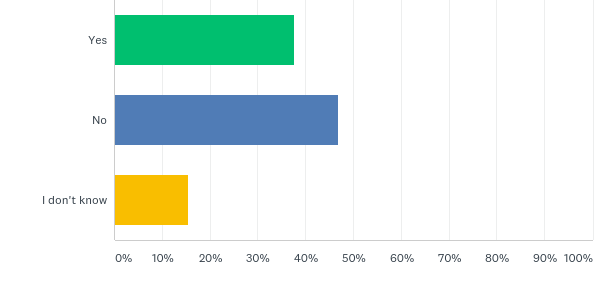

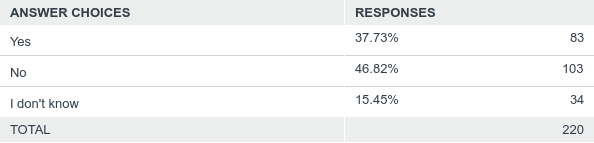


**Q21: Recommendation: "Staple line reinforcement* in sleeve gastrectomy should be considered to reduce the risk of perioperative complications** ". Is this applicable to your practice?
*Buttress, glues, suturing, clips ** Overall mortality, bleeding**

Answered: 220 Skipped: 0


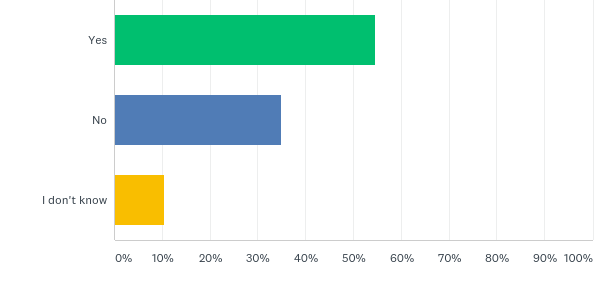

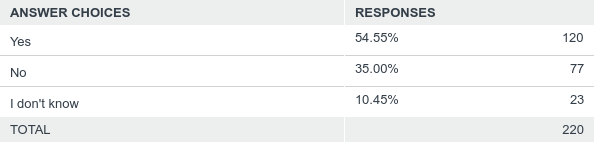


**Q22: Position statement:"There is insufficient evidence to recommend routine staple line reinforcement to reduce the leak rate". Is this applicable to your practice?**

Answered: 21 Skipped: 199


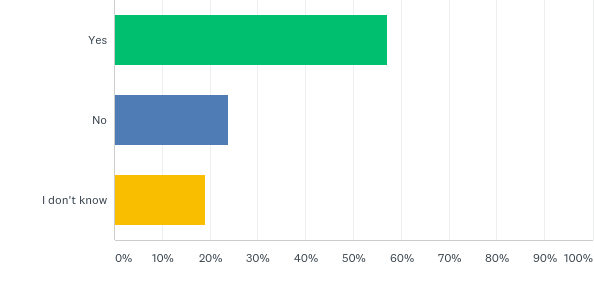

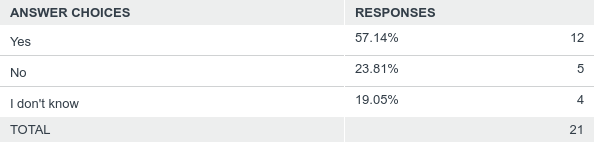


**Q23: Position statement: "RYGB results in better weight loss and control/remission of insulin resistance and type 2 diabetes compared with gastric plication ". Is this applicable to your practice?**

Answered: 220 Skipped: 0


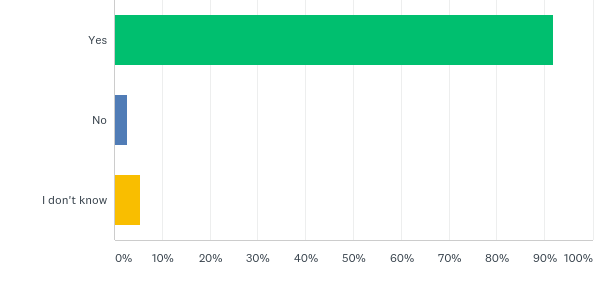

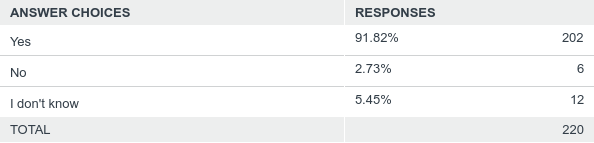


**Q24: Recommendation: "RYGB should be preferred over Adjustable Gastric Banding". Is this applicable to your practice?**

Answered: 220 Skipped: 0


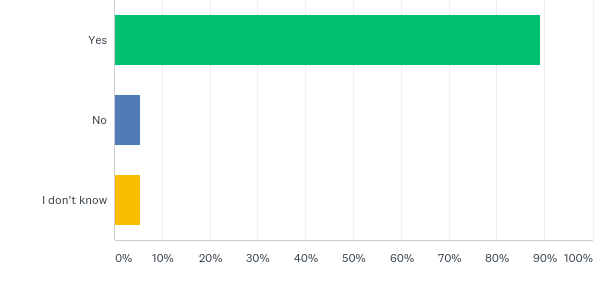

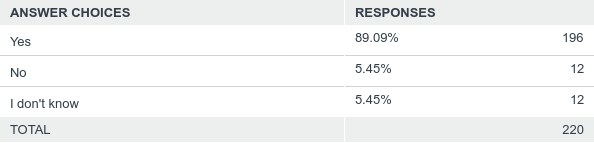


**Q25: Position statement: "RYGB offers similar mid-term weight loss and control/remission of metabolic co-morbidities compared to sleeve gastrectomy. Long-term comparative data are, however, lacking". Is this applicable to your practice?**

Answered: 220 Skipped: 0


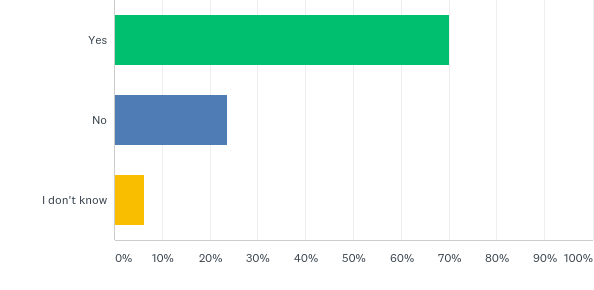

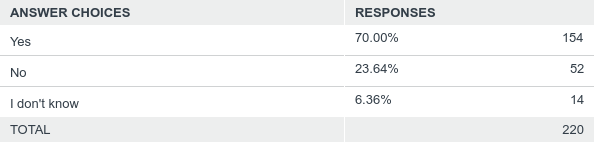


**Q26: Recommendation: "RYGB may be preferred over sleeve gastrectomy in patients with severe gastroesophageal reflux disease and/or severe esophagitis". Is this applicable to your practice?**

Answered: 220 Skipped: 0


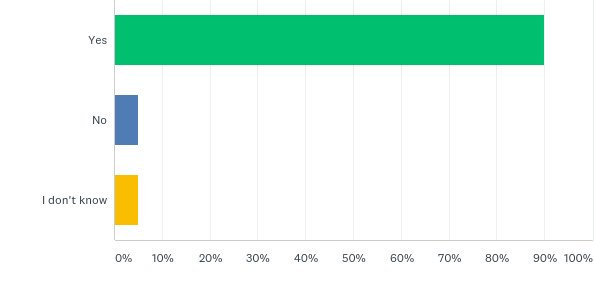

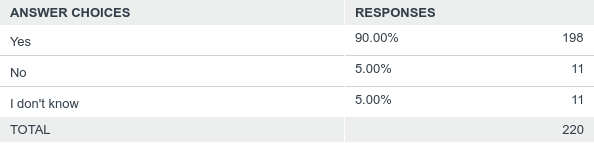


**Q27: Position statement: "OAGB may offer better short-term weight loss compared to RYGB, gastric plication, adjustable gastric banding or sleeve gastrectomy. Long-term comparative data are, however, lacking. Nutritional deficiencies remain a controversial issue". Is this applicable to your practice?**

Answered: 220 Skipped: 0


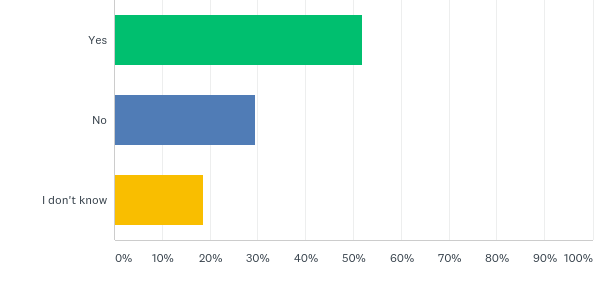

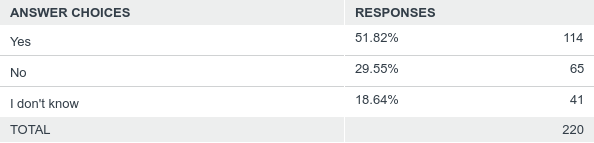


**Q28: Recommendation: "No recommendation for either SADI-S, BPD/DS, RYGB or sleeve gastrectomy can be made on the basis of available comparative evidence".Is this applicable to your practice?**

Answered: 220 Skipped: 0


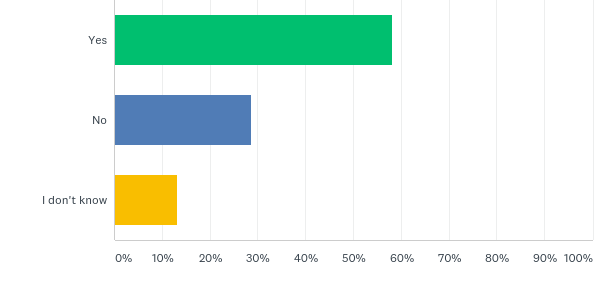

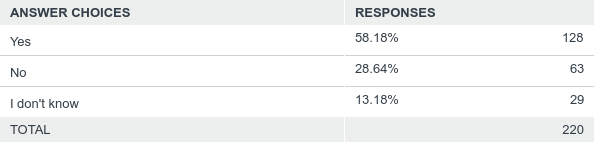


**Q29: Positions statement: "With regard to mid-term weight loss there is no difference between BPD/DS and RYGB. BPD/DS is superior to RYGB for control/remission of type 2 diabetes. Long-term comparative data are, however, lacking". Is this applicable to your practice?**

Answered: 220 Skipped: 0


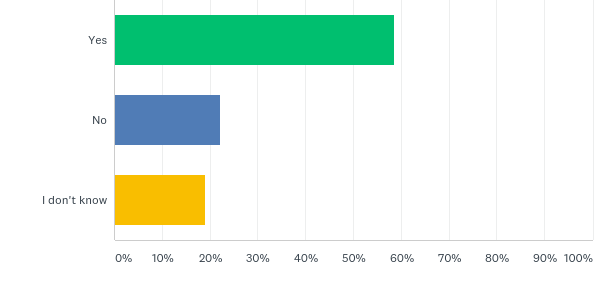

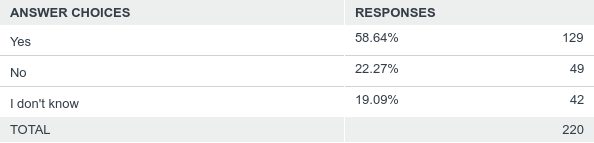


**Q30: Recommendation: "No recommendation for either BPD/DS or sleeve gastrectomy can be made on the basis of available comparative evidence"**

Answered: 220 Skipped: 0


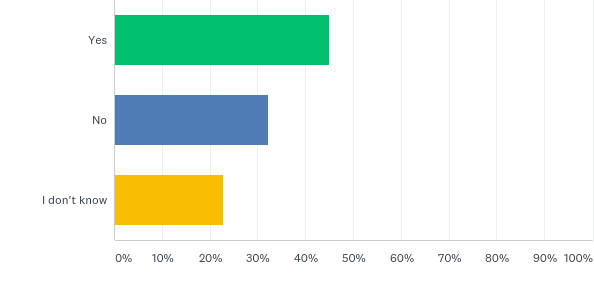

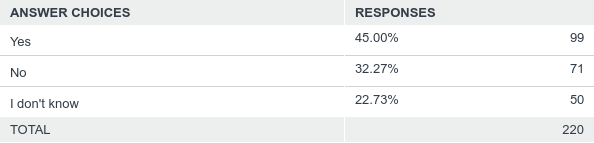


**Q31: Position statement:"No evidence-based criteria for indication to Revisional Metabolic/Bariatric Surgery are available to date. The expert panel advises that the clinical decision to proceed to revisional bariatric surgery should be based on a complete multidisciplinary assessment of the patient, as recommended for the primary procedure" Is this applicable to your practice ?**

Answered: 220 Skipped: 0


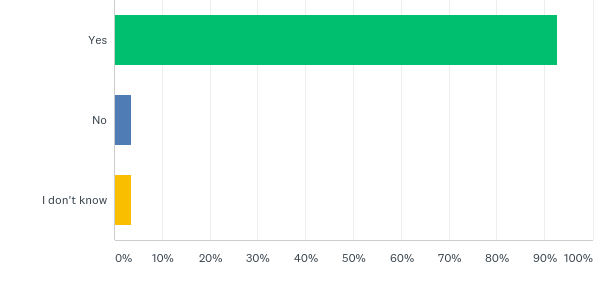

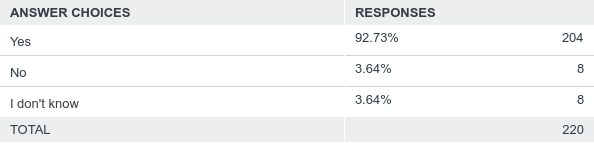


**Q32: Recommendation: "Scheduled multidisciplinary post-operative follow up should be provided to every patient undergoing laparoscopic bariatric/metabolic surgery". Is this applicable to your practice?**

Answered: 220 Skipped: 0


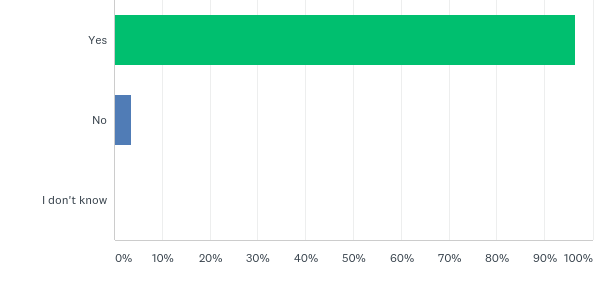

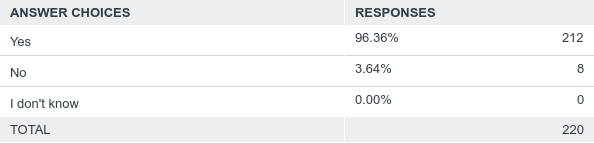


**Q33: Recommendation: " Treatment with ursodeoxycolic acid could be considered during the weight loss phase to prevent gallstones formation". Is this applicable to your practice?**

Answered: 220 Skipped: 0


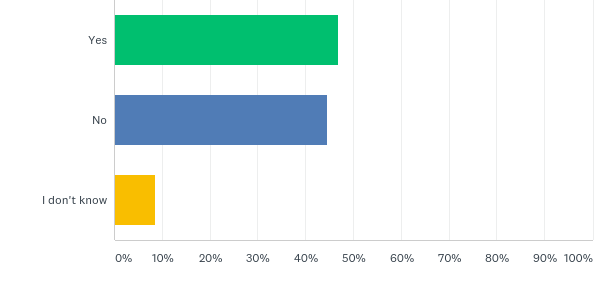

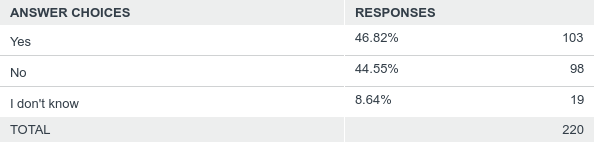


**Q34: Recommendation: "Micro and/or macronutrients supplementation is recommended after bariatric surgery according to the type of the procedure and to the deficiencies documented during the follow up". Is this applicable to your practice?**

Answered: 220 Skipped: 0


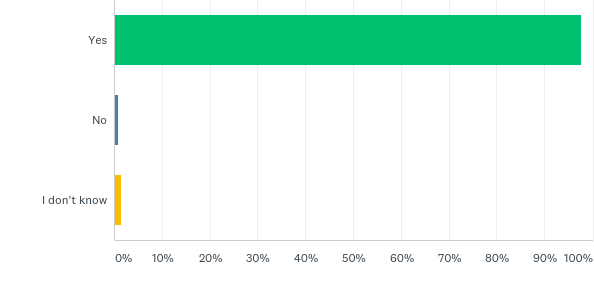

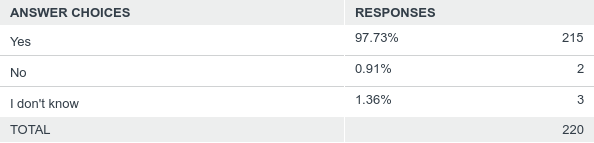


**Q35: Recommendation: "PPI therapy should be given to patients undergoing bypass procedures for the prevention of marginal ulcers". Is this applicable to your practice?**

Answered: 220 Skipped: 0


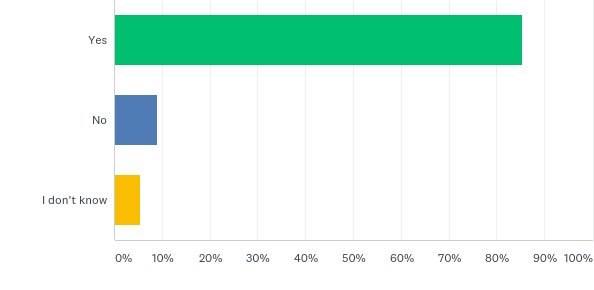

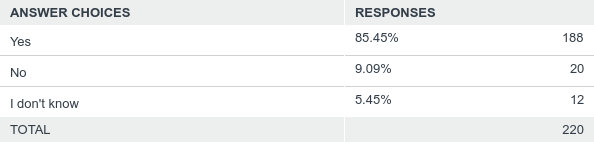


**Q36: Recommendation: "Post-operative nutritional and behavioral advice should be provided to patients undergoing bariatric surgery". Is this applicable to your practice?**

Answered: 220 Skipped: 0


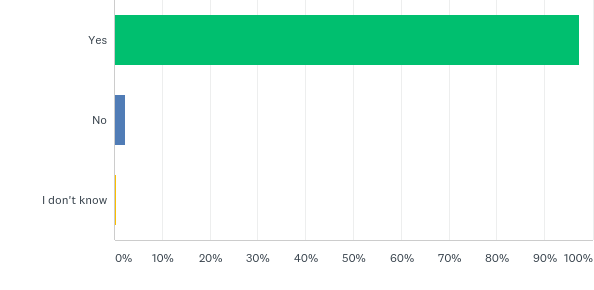


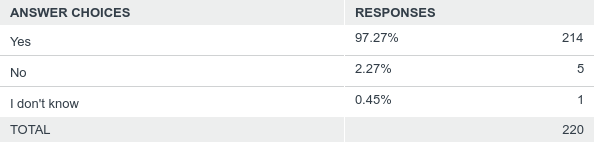


**Q37: Recommendation: "Pregnancy following bariatric surgery should be delayed during the weight loss phase". Is this applicable to your practice?**

Answered: 220 Skipped: 0


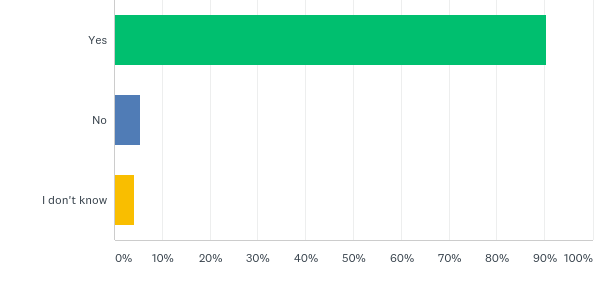


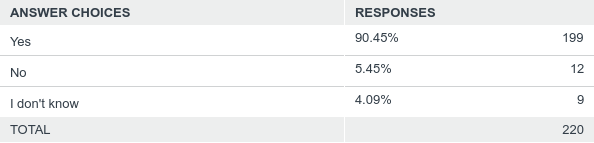


**Q38: Position statement: " For duodenal-jejunal bypass sleeves, aspiration devices, gastric electrical stimulation, vagal blockade and duodenal mucosal resurfacing, the quality of evidence was too low to provide any recommendationsEndoluminal suturing procedures may have a role in the treatment of obese patients with BMI below 40 kg/m2". Is this applicable to your practice?**

Answered: 220 Skipped: 0


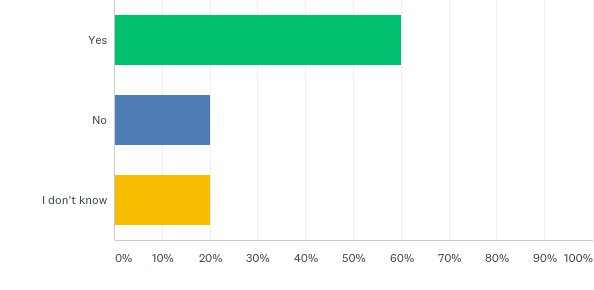

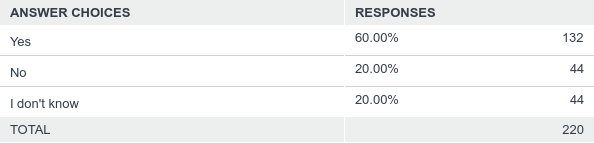

Supplement: Supplementary file 6 — Supplementary file6 (DOCX 733 kb) [file 464_2020_7555_MOESM6_ESM.docx]
