# Supplementary material for: Clinical practice guidelines of the European Association for Endoscopic Surgery (EAES) on bariatric surgery: update 2020 endorsed by IFSO-EC, EASO and ESPCOP
Source: Surg Endosc. 2020 Apr 23;34(6):2332–58. doi: 10.1007/s00464-020-07555-y (PMC7214495; doi:10.1007/s00464-020-07555-y)
Supplement: Supplementary file 7 — Supplementary file7 (DOCX 125 kb) [file 464_2020_7555_MOESM7_ESM.docx]

**Supplementary file 7**

**RESULTS OF EXTERNAL REVIEW USING THE AGREE II INSTRUMENT**

| **Domain** | **Item** | **Mean AGREE II Rating (Scale 1-7)** | | |
| --- | --- | --- | --- | --- |
| Scope and purpose | 1. The overall objective(s) of the guideline is (are) specifically described. | 6.5 | | |
|  | 1. The health question(s) covered by the guideline is (are) specifically described. | 6.5 | | |
|  | 1. The population (patients, public, etc.) to whom the guideline is meant to apply is specifically described. | 6.5 | | |
| Stakeholder involvement | 1. The guideline development group includes individuals from all the relevant professional groups. | 6.5 | | |
|  | 1. The views and preferences of the target population (patients, public, etc.) have been sought. | 6.0 | | |
|  | 1. The target users of the guideline are clearly defined. | 6.5 | | |
| Rigor of development | 1. Systematic methods were used to search for evidence. | 6.5 | | |
|  | 1. The criteria for selecting the evidence are clearly described. | 6.5 | | |
|  | 1. The strengths and limitations of the body of evidence are clearly described. | 7.0 | | |
|  | 1. The methods for formulating the recommendations are clearly described. | 6.5 | | |
|  | 1. The health benefits, side effects and risks have been considered in formulating the recommendations. | 6.5 | | |
|  | 1. There is an explicit link between the recommendations and the supporting evidence. | 6.5 | | |
|  | 1. The guideline has been externally reviewed by experts prior to its publication. | 7.0 | | |
|  | 1. A procedure for updating the guideline is provided. | 7.0 | | |
| Clarity of presentation | 1. The recommendations are specific and unambiguous. | 6.5 | | |
|  | 1. The different options for management of the condition or health issue are clearly presented. | 6.5 | | |
|  | 1. Key recommendations are easily identifiable. | 6.5 | | |
| Applicability | 1. The guideline describes facilitators and barriers to its application. | 7.0 | | |
|  | 1. The guideline provides advice and/or tools on how the recommendations can be put into practice. | 7.0 | | |
|  | 1. The potential resource implications of applying the recommendations have been considered. | 7.0 | | |
|  | 1. The guideline presents monitoring and/ or auditing criteria. | 7.0 | | |
| Editorial independence | 1. The views of the funding body have not influenced the content of the guideline. | 7.0 | | |
|  | 1. Competing interests of guideline development group members have been recorded and addressed. | 7.0 | | |
| Overall Guideline Assessment | 1. Rate the overall quality of this guideline. | 6.5 | | |
| Overall Guideline Assessment | 1. I would recommend this guideline for use. | *Yes* | *Yes, with modifi-cations* | *No* |
|  |  | * |  |  |
| * Both reviewers indicated that they would recommend this guideline for use | | | | |
