# Supplementary material for: Clinical practice guidelines of the European Association for Endoscopic Surgery (EAES) on bariatric surgery: update 2020 endorsed by IFSO-EC, EASO and ESPCOP
Source: Surg Endosc. 2020 Apr 23;34(6):2332–58. doi: 10.1007/s00464-020-07555-y (PMC7214495; doi:10.1007/s00464-020-07555-y)
Supplement: Supplementary file 8 — Supplementary file8 (PDF 149 kb) [file 464_2020_7555_MOESM8_ESM.pdf]

Question: Should bariatric surgery vs. non-surgical management be used for weight loss?

Certainty assessment							№ of patients		Effect		Certainty	Importance
№ of studies	Study design	Risk of bias	Inconsistency	Indirectness	Imprecision	Other considerations	Bariatric surgery	Non surgical management	Relative (95% CI)	Absolute (95% CI)		
**EWL (assessed with: %)**												
1	randomised trials	serious	not serious	very serious	not serious	very strong association	20	24	-	MD **52.6 % higher** (42.07 higher to 63.13 higher)	⨁⨁⨁◯ MODERATE	CRITICAL
**Post-intervention weight (assessed with: kg)**												
4	randomised trials	serious	serious	serious	not serious	strong association	129	132	-	MD **19.25 lower** (26.94 lower to 11.57 lower)	⨁⨁◯◯ LOW	CRITICAL
**Complications**												
10	randomised trials	serious	serious	serious	serious	publication bias strongly suspected	226/572 (39.5%)	109/423 (25.8%)	**OR 2.44** (1.47 to 4.06)	**201 more per 1.000** (from 80 more to 327 more)	⨁◯◯◯ VERY LOW	CRITICAL
**Mortality**												
10	randomised trials	serious	not serious	serious	not serious	none	0/393 (0.0%)	3/296 (1.0%)	**RD -0.01** (-0.03 to 0.01)	**10 more per 1.000** (from 10 fewer to 30 more)	⨁⨁◯◯ LOW	CRITICAL

**HbA1c (assessed with: mg/dL)**												
7	randomised trials	serious	very serious	serious	not serious	none	289	220	-	MD **0.04 lower** (1.18 lower to 1.09 higher)	⊕◯◯◯ VERY LOW	IMPORTANT
**Systolic blood pressure (assessed with: mmHg)**												
6	randomised trials	serious	not serious	serious	not serious	none	260	184	-	MD **2.13 lower** (4.36 lower to 0.09 higher)	⊕⊕◯◯ LOW	IMPORTANT
**Diastolic blood pressure (assessed with: mmHg)**												
6	randomised trials	serious	serious	serious	not serious	none	260	184	-	MD **1.15 lower** (3.5 lower to 1.21 higher)	⊕◯◯◯ VERY LOW	IMPORTANT
**Total cholesterol**												
7	randomised trials	serious	very serious	serious	not serious	none	294	223	-	SMD **0.56 lower** (1.33 lower to 0.2 higher)	⊕◯◯◯ VERY LOW	IMPORTANT
**Triglycerides**												
6	randomised trials	serious	very serious	serious	not serious	none	195	182	-	SMD **1.09 lower** (1.74 lower to 0.44 lower)	⊕◯◯◯ VERY LOW	IMPORTANT
**LDL**												
6	randomised trials	serious	very serious	serious	not serious	none	262	174	-	SMD **0.71 lower** (1.6 lower to 0.18 higher)	⊕◯◯◯ VERY LOW	IMPORTANT

**HDL**												
6	randomised trials	serious	very serious	serious	not serious	none	260	184	-	SMD **0.82 higher** (0.14 higher to 1.51 higher)	⨁◯◯◯ VERY LOW	IMPORTANT
**T2DM resolution**												
5	randomised trials	serious	not serious	serious	very serious	very strong association	135/213 (63.4%)	6/115 (5.2%)	**OR 29.07** (12.69 to 66.63)	**563 more per 1.000** (from 359 more to 734 more)	⨁⨁◯◯ LOW	IMPORTANT

CI: Confidence interval; **MD:** Mean difference; **OR:** Odds ratio; **SMD:** Standardised mean difference
