# Supplementary material for: Clinical practice guidelines of the European Association for Endoscopic Surgery (EAES) on bariatric surgery: update 2020 endorsed by IFSO-EC, EASO and ESPCOP
Source: Surg Endosc. 2020 Apr 23;34(6):2332–58. doi: 10.1007/s00464-020-07555-y (PMC7214495; doi:10.1007/s00464-020-07555-y)
Supplement: Supplementary file 9 — Supplementary file9 (PDF 113 kb) [file 464_2020_7555_MOESM9_ESM.pdf]

Question: Should preoperative Helicobacter pylori eradication vs. standard care be used for prevention of foregut symptoms in patients undergoing bariatric surgery?

Certainty assessment							№ of patients		Effect		Certainty	Importance
№ of studies	Study design	Risk of bias	Inconsistency	Indirectness	Imprecision	Other considerations	preoperative Helicobacter pylori eradication	standard care	Relative (95% CI)	Absolute (95% CI)		
**Marginal ulcer (follow up: range 3 months to 60 months)**												
4	observational studies	not serious	very serious	very serious	very serious	none	The OR for H. pylori positive status versus H. pylori negative status was 0.51, 95% CI 0.03 to 8.35.				⊕◯◯◯ VERY LOW	IMPORTANT
**Postoperative complications**												
5	not serious<br>observational studies		very serious	very serious	very serious	none	The OR for H. pylori positive status versus H. pylori negative status was 2.86, 95% CI 0.26 to 31.27.				⊕◯◯◯ VERY LOW	CRITICAL
**Incidence of bleeding**												
3	observational studies	not serious	not serious	very serious	very serious	none	The OR for H. pylori positive status versus H. pylori negative status was 0.90, 95% CI 0.23–3.52.				⊕◯◯◯ VERY LOW	CRITICAL
**Incidence of leakage**												
4	observational studies	not serious	very serious	very serious	very serious	none	The OR for H. pylori positive status versus H. pylori negative status was 1.62, 95% CI 0.17 to 15.62.				⊕◯◯◯ VERY LOW	CRITICAL

CI: Confidence interval
