# Supplementary material for: Clinical practice guidelines of the European Association for Endoscopic Surgery (EAES) on bariatric surgery: update 2020 endorsed by IFSO-EC, EASO and ESPCOP
Source: Surg Endosc. 2020 Apr 23;34(6):2332–58. doi: 10.1007/s00464-020-07555-y (PMC7214495; doi:10.1007/s00464-020-07555-y)
Supplement: Supplementary file 10 — Supplementary file10 (PDF 107 kb) [file 464_2020_7555_MOESM10_ESM.pdf]

Question: Should preoperative diet consultation vs. standard care be used for weight loss in patients undergoing bariatric surgery?

Certainty assessment							№ of patients		Effect		Certainty	Importance
№ of studies	Study design	Risk of bias	Inconsistency	Indirectness	Imprecision	Other considerations	preoperative diet consultation	standard care	Relative (95% CI)	Absolute (95% CI)		
**Weight loss (follow up: range 2 weeks to 6 months)**												
3	randomised trials	serious	very serious	not serious	serious	none	220	218	-	SMD **0.4 SD higher** (0.03 higher to 0.78 higher)	⨁◯◯◯ VERY LOW	CRITICAL
**Postoperative complications (follow up: range 2 weeks to 6 months)**												
2	randomised trials	serious	serious	not serious	not serious	none	0/163 (0.0%)	0/171 (0.0%)	**RR 0.80** (0.22 to 2.86)	**0 fewer per 1.000** (from 0 fewer to 0 fewer)	⨁⨁◯◯ LOW	CRITICAL

CI: Confidence interval; **SMD:** Standardised mean difference; **RR:** Risk ratio
