# Supplementary material for: Clinical practice guidelines of the European Association for Endoscopic Surgery (EAES) on bariatric surgery: update 2020 endorsed by IFSO-EC, EASO and ESPCOP
Source: Surg Endosc. 2020 Apr 23;34(6):2332–58. doi: 10.1007/s00464-020-07555-y (PMC7214495; doi:10.1007/s00464-020-07555-y)
Supplement: Supplementary file 11 — Supplementary file11 (PDF 107 kb) [file 464_2020_7555_MOESM11_ESM.pdf]

Question: Should preoperative esophagogastroscopy vs. no endoscopy be used for preoperative evaluation in patients undergoing bariatric surgery?

Certainty assessment							№ of patients		Effect		Certainty	Importance
**№ of studies**	**Study design**	**Risk of bias**	**Inconsistency**	**Indirectness**	**Imprecision**	**Other considerations**	**preoperative esophagogastroscopy**	**no endoscopy**	**Relative (95% CI)**	**Absolute (95% CI)**		
**Change of surgical management**												
23	observational studies	serious	serious	serious	not serious	publication bias strongly suspected	Meta-analysis yielded a proportion of change in surgical management after EGD in 7.8% (95% CI, 6.1 to 9.5; I^2 = 98.1%).				⊕◯◯◯ VERY LOW	IMPORTANT
**Change of medical management**												
20	observational studies	serious	serious	serious	not serious	publication bias strongly suspected	Meta-analysis found a proportion of change in medical management after EGD of 27.5% (95% CI, 20.2–34.8; I^2 = 98.6%).				⊕◯◯◯ VERY LOW	IMPORTANT

CI: Confidence interval
