# Supplementary material for: Clinical practice guidelines of the European Association for Endoscopic Surgery (EAES) on bariatric surgery: update 2020 endorsed by IFSO-EC, EASO and ESPCOP
Source: Surg Endosc. 2020 Apr 23;34(6):2332–58. doi: 10.1007/s00464-020-07555-y (PMC7214495; doi:10.1007/s00464-020-07555-y)
Supplement: Supplementary file 12 — Supplementary file12 (PDF 57 kb) [file 464_2020_7555_MOESM12_ESM.pdf]

Question: Should assessment of pre-operative psychological conditions vs. no assesment preoperative psychological conditions be used in bariatric surgery prior to operation?

Certainty assessment							Impact	Certainty	Importance
**№ of studies**	**Study design**	**Risk of bias**	**Inconsistency**	**Indirectness**	**Imprecision**	**Other considerations**			
**Weight loss**									
9	observational studies	serious	serious	not serious	serious	publication bias strongly suspected	The authors report "There was conflicting evidence regarding the association between preoperative mental health conditions and postoperative weight loss. Neither depression nor binge eating disorder was consistently associated with differences in weight outcomes."	⨁◯◯◯ VERY LOW	IMPORTANT

CI: Confidence interval
