# Supplementary material for: Clinical practice guidelines of the European Association for Endoscopic Surgery (EAES) on bariatric surgery: update 2020 endorsed by IFSO-EC, EASO and ESPCOP
Source: Surg Endosc. 2020 Apr 23;34(6):2332–58. doi: 10.1007/s00464-020-07555-y (PMC7214495; doi:10.1007/s00464-020-07555-y)
Supplement: Supplementary file 13 — Supplementary file13 (PDF 121 kb) [file 464_2020_7555_MOESM13_ESM.pdf]

Question: Should perioperative OSA screening vs. no OSA screening be used for prevention of respiratory complications after bariatric surgery?

Certainty assessment							№ of patients		Effect		Certainty	Importance
№ of studies	Study design	Risk of bias	Inconsistency	Indirectness	Imprecision	Other considerations	Perioperative OSA screening	No OSA screening	Relative (95% CI)	Absolute (95% CI)		
**Mortality**												
5	observational studies	not serious	not serious	serious	not serious	none	68/34882 (0.2%)	174/60858 (0.3%)	**OR 3.39** (0.12 to 92.08)	**7 more per 1.000** (from 3 fewer to 206 more)	⨁◯◯◯ VERY LOW	CRITICAL
**Pulmonary complications**												
7	observational studies	not serious	not serious	serious	not serious	none	1819/67481 (2.7%)	327/116601 (0.3%)	**OR 1.85** (0.46 to 7.42)	**2 more per 1.000** (from 2 fewer to 18 more)	⨁◯◯◯ VERY LOW	CRITICAL
**Pneumonia**												
5	observational studies	not serious	not serious	serious	not serious	none	239/34328 (0.7%)	593/60665 (1.0%)	**OR 2.51** (0.68 to 9.21)	**14 more per 1.000** (from 3 fewer to 74 more)	⨁◯◯◯ VERY LOW	IMPORTANT
**Cardiac complications**												
4	observational studies	not serious	not serious	serious	not serious	none	80/34196 (0.2%)	77/58690 (0.1%)	**OR 1.85** (0.72 to 4.75)	**1 more per 1.000** (from 0 fewer to 5 more)	⨁◯◯◯ VERY LOW	CRITICAL

**Cardiac complications (assessed with: Atrial fibrillation)**											
2	observational studies	not serious	not serious	serious	not serious	none	623/33814 (1.8%)	698/58011 (1.2%)	**OR 1.51** (1.36 to 1.69)	**6 more per 1.000** (from 4 more to 8 more)	⨁◯◯◯ VERY LOW	CRITICAL
**Length of Stay**											
5	observational studies	not serious	not serious	serious	not serious	none	33787	59475	-	MD **0.69 days higher** (0.17 lower to 1.57 higher)	⨁◯◯◯ VERY LOW	IMPORTANT
**Hypoxemia (assessed with: SpO2 (%) day 0,1(mean, SD))**											
3	observational studies	not serious	not serious	serious	not serious	none	395	833	-	MD **3.8 % lower** (5.38 lower to 2.21 lower)	⨁◯◯◯ VERY LOW	CRITICAL

CI: Confidence interval; **OR:** Odds ratio; **MD:** Mean difference
