# Supplementary material for: Clinical practice guidelines of the European Association for Endoscopic Surgery (EAES) on bariatric surgery: update 2020 endorsed by IFSO-EC, EASO and ESPCOP
Source: Surg Endosc. 2020 Apr 23;34(6):2332–58. doi: 10.1007/s00464-020-07555-y (PMC7214495; doi:10.1007/s00464-020-07555-y)
Supplement: Supplementary file 14 — Supplementary file14 (PDF 102 kb) [file 464_2020_7555_MOESM14_ESM.pdf]

Question: Should perioperative CPAP vs. no CPAP be used for prevention of respiratory complications after bariatric surgery?

Certainty assessment							№ of patients		Effect		Certainty	Importance
№ of studies	Study design	Risk of bias	Inconsistency	Indirectness	Imprecision	Other considerations	perioperative CPAP	no CPAP	Relative (95% CI)	Absolute (95% CI)		
**Mortality**												
3	observational studies	not serious	not serious	not serious	serious	none	0/268 (0.0%)	2/350 (0.6%)	**OR 0.36** (0.02 to 7.63)	**4 fewer per 1.000** (from 6 fewer to 36 more)	⨁◯◯◯ VERY LOW	CRITICAL
