# Supplementary material for: Clinical practice guidelines of the European Association for Endoscopic Surgery (EAES) on bariatric surgery: update 2020 endorsed by IFSO-EC, EASO and ESPCOP
Source: Surg Endosc. 2020 Apr 23;34(6):2332–58. doi: 10.1007/s00464-020-07555-y (PMC7214495; doi:10.1007/s00464-020-07555-y)
Supplement: Supplementary file 15 — Supplementary file15 (PDF 67 kb) [file 464_2020_7555_MOESM15_ESM.pdf]

Question: Should high dose pharmacological antithrombotic prophylaxis vs. standard dose antithrombotic prophylaxis be used for prevention of thromboembolism in patients undergoing bariatric surgery?

Certainty assessment							№ of patients		Effect		Certainty	Importance
**№ of studies**	**Study design**	**Risk of bias**	**Inconsistency**	**Indirectness**	**Imprecision**	**Other considerations**	**high dose pharmacological antithrombotic prophylaxis**	**standard dose antithrombotic prophylaxis**	**Relative (95% CI)**	**Absolute (95% CI)**		
**DVT**												
2	randomised trials	not serious	not serious	not serious	very serious	none	2/231 (0.9%)	1/217 (0.5%)	**OR 1.83** (0.16 to 20.44)	**4 more per 1.000** (from 4 fewer to 82 more)	⨁⨁◯◯ LOW	CRITICAL
**Bleeding**												
2	randomised trials	not serious	not serious	not serious	serious	none	8/221 (3.6%)	6/217 (2.8%)	**OR 1.22** (0.41 to 3.64)	**6 more per 1.000** (from 16 fewer to 66 more)	⨁⨁⨁◯ MODERATE	CRITICAL

CI: Confidence interval; **OR:** Odds ratio
