# Supplementary material for: Clinical practice guidelines of the European Association for Endoscopic Surgery (EAES) on bariatric surgery: update 2020 endorsed by IFSO-EC, EASO and ESPCOP
Source: Surg Endosc. 2020 Apr 23;34(6):2332–58. doi: 10.1007/s00464-020-07555-y (PMC7214495; doi:10.1007/s00464-020-07555-y)
Supplement: Supplementary file 16 — Supplementary file16 (PDF 116 kb) [file 464_2020_7555_MOESM16_ESM.pdf]

Question: Should inferior vena cava filter (IVCF) vs. low molecular weight heparin (LMWH) and intermittent pneumatic compression (IPC) be used for prevention of thromboembolism in patients undergoing bariatric surgery?

Certainty assessment							№ of patients		Effect		Certainty	Importance
№ of studies	Study design	Risk of bias	Inconsistency	Indirectness	Imprecision	Other considerations	inferior vena cava filter (IVCF)	low molecular weight heparine (LMWH) and intermittent pneumatic compression (IPC)	Relative (95% CI)	Absolute (95% CI)		
**DVT**												
3	observational studies	not serious	not serious	not serious	not serious	none	22/1779 (1.2%)	38/7081 (0.5%)	**OR 1.98** (1.04 to 3.75)	**5 more per 1.000** (from 0 fewer to 14 more)	⨁⨁◯◯ LOW	CRITICAL
**Pulmonary embolism**												
2	observational studies	not serious	not serious	not serious	not serious	none	10/1237 (0.8%)	10/1247 (0.8%)	**OR 1.02** (0.42 to 2.46)	**0 fewer per 1.000** (from 5 fewer to 11 more)	⨁⨁◯◯ LOW	CRITICAL
**Serious complications**												
1	randomised trials	not serious	not serious	not serious	not serious	none	26/542 (4.8%)	214/5834 (3.7%)	**OR 1.32** (0.87 to 2.01)	**11 more per 1.000** (from 5 fewer to 34 more)	⨁⨁⨁⨁ HIGH	
**Post-operative mortality (30 days)**												

3	observational studies	not serious	not serious	not serious	not serious	none	15/1779 (0.8%)	31/7081 (0.4%)	**OR 3.14** (1.49 to 6.63)	**9 more per 1.000** (from 2 more to 24 more)	⨁⨁◯◯ LOW	CRITICAL

CI: Confidence interval; **OR:** Odds ratio
