# Supplementary material for: Clinical practice guidelines of the European Association for Endoscopic Surgery (EAES) on bariatric surgery: update 2020 endorsed by IFSO-EC, EASO and ESPCOP
Source: Surg Endosc. 2020 Apr 23;34(6):2332–58. doi: 10.1007/s00464-020-07555-y (PMC7214495; doi:10.1007/s00464-020-07555-y)
Supplement: Supplementary file 17 — Supplementary file17 (PDF 120 kb) [file 464_2020_7555_MOESM17_ESM.pdf]

**Question**: Should ERAS protocol vs. standard care be used for patients undergoing bariatric surgery?

| Certainty assessment | | | | | | | № of patients | | Effect | | Certainty | Importance |
|---|---|---|---|---|---|---|---|---|---|---|---|---|
| № of studies | Study design | Risk of bias | Inconsistency | Indirectness | Imprecision | Other considerations | ERAS protocol | standard care | Relative (95% CI) | Absolute (95% CI) | | |
| **Major postoperative complications (follow up: range 14 days to 8 weeks)** | | | | | | | | | | | | |
| 5 | observational studies | serious | serious | not serious | serious | none | 163/3278 (5.0%) | 77/1578 (4.9%) | **OR 0.94** (0.58 to 1.51) | **3 fewer per 1.000** (from 20 fewer to 23 more) | ⨁◯◯◯ VERY LOW | CRITICAL |
| **Minor postoperative complications (follow up: range 14 days to 8 weeks)** | | | | | | | | | | | | |
| 5 | observational studies | serious | very serious | not serious | not serious | none | 370/3278 (11.3%) | 182/5781 (3.1%) | **OR 0.88** (0.55 to 1.41) | **4 fewer per 1.000** (from 14 fewer to 12 more) | ⨁◯◯◯ VERY LOW | IMPORTANT |
| **Length of stay** | | | | | | | | | | | | |
| 10 | observational studies | serious | very serious | not serious | serious | strong association | 2291 | 1366 | - | MD **2.4 days lower** (3.9 lower to 0.9 lower) | ⨁◯◯◯ VERY LOW | CRITICAL |
| **Readmission** | | | | | | | | | | | | |
| 9 | observational studies | serious | serious | not serious | not serious | publication bias strongly suspected strong association | 214/3330 (6.4%) | 122/1677 (7.3%) | **OR 0.86** (0.57 to 1.30) | **10 fewer per 1.000** (from 30 fewer to 20 more) | ⨁◯◯◯ VERY LOW | CRITICAL |

**Mortality**

| | | | | | | | | | | | | |
|---|---|---|---|---|---|---|---|---|---|---|---|---|
| 8 | observational studies | serious | serious | not serious | serious | strong association | 1/2006 (0.0%) | 3/1163 (0.3%) | **RR 0.4** (0.1 to 2.2) | **2 fewer per 1.000** (from 2 fewer to **3** more) | ⊕◯◯◯ VERY LOW | CRITICAL |
| **Cost** | | | | | | | | | | | | |
| 2 | observational studies | very serious | very serious | serious | very serious | publication bias strongly suspected strong association all plausible residual confounding would reduce the demonstrated effect dose response gradient | 82 | 65 | - | SMD **1.17 SD fewer** (2.83 fewer to 0.5 more) | ⊕◯◯◯ VERY LOW | IMPORTANT |

**CI:** Confidence interval; **OR:** Odds ratio; **MD:** Mean difference; **RR:** Risk ratio; **SMD:** Standardised mean difference
