# Supplementary material for: Clinical practice guidelines of the European Association for Endoscopic Surgery (EAES) on bariatric surgery: update 2020 endorsed by IFSO-EC, EASO and ESPCOP
Source: Surg Endosc. 2020 Apr 23;34(6):2332–58. doi: 10.1007/s00464-020-07555-y (PMC7214495; doi:10.1007/s00464-020-07555-y)
Supplement: Supplementary file 18 — Supplementary file18 (PDF 141 kb) [file 464_2020_7555_MOESM18_ESM.pdf]

Question: Should multimodal analgesia with minimal use of opioids vs. standard analgesia be used for pain control in patients after bariatric surgery?

Certainty assessment							№ of patients		Effect			
№ of studies	Study design	Risk of bias	Inconsistency	Indirectness	Imprecision	Other considerations	multimodal analgesia with minimal use of opioids	standard analgesia	Relative (95% CI)	Absolute (95% CI)	Certainty	Importance
**Mortality**												
1	observational studies	serious	not serious	not serious	very serious	none	1/245 (0.4%)	0/384 (0.0%)	**OR 4.72** (0.19 to 116.28)	**0 fewer per 1.000** (from 0 fewer to 0 fewer)	⨁◯◯◯ VERY LOW	CRITICAL
**Re-intubation**												
1	observational studies	serious	not serious	serious	very serious	none	2/245 (0.8%)	2/384 (0.5%)	**OR 1.57** (0.22 to 11.23)	**3 more per 1.000** (from 4 fewer to 50 more)	⨁◯◯◯ VERY LOW	CRITICAL
**Cardio-vascular complications**												
1	observational studies	serious	not serious	not serious	very serious	none	1/245 (0.4%)	3/384 (0.8%)	**OR 0.52** (0.05 to 5.03)	**4 fewer per 1.000** (from 7 fewer to 30 more)	⨁◯◯◯ VERY LOW	CRITICAL
**Pulmonary complications**												
1	observational studies	serious	not serious	serious	very serious	none	5/245 (2.0%)	1/384 (0.3%)	**OR 7.98** (0.93 to 68.71)	**18 more per 1.000** (from 0 fewer to 150 more)	⨁◯◯◯ VERY LOW	CRITICAL
**Pulmonary complications**												

3	randomised trials	not serious	not serious	not serious	not serious	none	2/252 (0.8%)	9/279 (3.2%)	**RD -0.02** (-0.05 to 0.00)	**-- per 1.000** (from to --)	⨁⨁⨁⨁ HIGH	CRITICAL
**Morphine consumption (assessed with: mg in first 24 h postoperative)**												
8	randomised trials	not serious	not serious	not serious	not serious	none	345	376	-	MD **1.66 mg lower** (2.43 lower to 0.88 higher)	⨁⨁⨁⨁ HIGH	IMPORTANT
**VAS score (30 min – 1 h) (assessed with: 1-10)**												
5	randomised trials	not serious	not serious	very serious	not serious	none	101	213	-	MD **0.64 number lower** (1.21 lower to 0.07 lower)	⨁⨁◯◯ LOW	IMPORTANT
**VAS score (6h)**												
3	randomised trials	not serious	not serious	not serious	not serious	none	126	148	-	MD **1.71 number lower** (3.49 lower to 0.06 higher)	⨁⨁⨁⨁ HIGH	IMPORTANT

VAS score (12h)

2	randomised trials	not serious	not serious	not serious	not serious	none	98	120	-	MD **0.75 number lower** (1.6 lower to 0.1 higher)	⊕⊕⊕⊕ HIGH	IMPORTANT

Length of stay in PACU (assessed with: min)

2	randomised trials	not serious	not serious	not serious	not serious	none	112	121	-	MD **0.99 min lower** (1.45 lower to 0.52 lower)	⊕⊕⊕⊕ HIGH	IMPORTANT

Nausea and Vomiting

4	randomised trials	not serious	not serious	not serious	not serious	none	36/190 (18.9%)	76/216 (35.2%)	**OR 0.40** (0.25 to 0.64)	**173 fewer per 1.000** (from 232 fewer to 94 fewer)	⊕⊕⊕⊕ HIGH	IMPORTANT

CI: Confidence interval; **OR:** Odds ratio; **MD:** Mean difference
