# Supplementary material for: Clinical practice guidelines of the European Association for Endoscopic Surgery (EAES) on bariatric surgery: update 2020 endorsed by IFSO-EC, EASO and ESPCOP
Source: Surg Endosc. 2020 Apr 23;34(6):2332–58. doi: 10.1007/s00464-020-07555-y (PMC7214495; doi:10.1007/s00464-020-07555-y)
Supplement: Supplementary file 19 — Supplementary file19 (PDF 61 kb) [file 464_2020_7555_MOESM19_ESM.pdf]

Question: Should sleeve gastrectomy vs. LAGB be used for weight loss?

Certainty assessment							№ of patients		Effect			
**№ of studies**	**Study design**	**Risk of bias**	**Inconsistency**	**Indirectness**	**Imprecision**	**Other considerations**	**sleeve gastrectomy**	**LAGB**	**Relative (95% CI)**	**Absolute (95% CI)**	**Certainty**	**Importance**
**%EWL (follow up: range 1 years to 5 years)**												
11	randomised trials	serious	not serious	not serious	serious	none	0	0	-	MD **26 % lower** (6.4 lower to 41 lower)	⨁⨁◯◯ LOW	

CI: Confidence interval; **MD:** Mean difference
