# Supplementary material for: Clinical practice guidelines of the European Association for Endoscopic Surgery (EAES) on bariatric surgery: update 2020 endorsed by IFSO-EC, EASO and ESPCOP
Source: Surg Endosc. 2020 Apr 23;34(6):2332–58. doi: 10.1007/s00464-020-07555-y (PMC7214495; doi:10.1007/s00464-020-07555-y)
Supplement: Supplementary file 20 — Supplementary file20 (PDF 141 kb) [file 464_2020_7555_MOESM20_ESM.pdf]

Question: Should sleeve gastrectomy vs. gastric plication be used for weight loss?

Certainty assessment							№ of patients		Effect		Certainty	Importance
№ of studies	Study design	Risk of bias	Inconsistency	Indirectness	Imprecision	Other considerations	sleeve gastrectomy	gastric plication	Relative (95% CI)	Absolute (95% CI)		
**EWL (follow up: range 4 months to 3 years)**												
8	observational studies	very serious [a]	very serious [b]	serious	very serious	none	233	189	-	MD **31 % higher** (10 higher to 72 higher)	⨁◯◯◯ VERY LOW	CRITICAL
**EWL for gastric plication (follow up: mean 5 years)**												
1	observational studies	serious	not serious	very serious	not serious	none	% EWL at 5y 52.6%, SD 4.9, 95% CI 51.9 to 53.3; n=244, follow up completed by 86.9%				⨁◯◯◯ VERY LOW	CRITICAL
**EWL for gastric plication (follow up: mean 10 years)**												
1	observational studies	very serious	not serious	very serious	not serious	none	% EWL at 10y 42%; n=800; follow up completed by 4.4%				⨁◯◯◯ VERY LOW	CRITICAL
**Postoperative complications**												
9	observational studies	serious	serious	not serious	serious	none	80/335 (23.9%)	39/350 (11.1%)	**OR 2.86** (1.47 to 5.88)	**153 more per 1.000** (from 44 more to 313 more)	⨁◯◯◯ VERY LOW	CRITICAL
**Readmission (follow up: 30 days)**												

2	observational studies	serious [c]	not serious	serious [d]	very serious	none	3/101 (3.0%)	3/100 (3.0%)	**OR 0.99** (0.19 to 5.05)	**0 fewer per 1.000** (from 24 fewer to 105 more)	⊕◯◯◯ VERY LOW	IMPORTANT
**Readmission (follow up: range 30 days to 1 years)**												
2	observational studies	serious [c]	not serious	serious [d]	very serious	none	1/101 (1.0%)	1/100 (1.0%)	**OR 0.99** (0.06 to 16.07)	**0 fewer per 1.000** (from 9 fewer to 130 more)	⊕◯◯◯ VERY LOW	IMPORTANT
**Reoperation**												
2	observational studies	serious [c]	not serious	serious [d]	very serious	none	2/101 (2.0%)	1/100 (1.0%)	**OR 0.66** (0.08 to 4.74)	**3 fewer per 1.000** (from 9 fewer to 36 more)	⊕◯◯◯ VERY LOW	IMPORTANT
**Reoperation (follow up: range 30 days to 1 years)**												
2	observational studies	serious [c]	not serious	serious [d]	very serious	none	1/101 (1.0%)	0/100 (0.0%)	**OR 0.32** (0.01 to 8.09)	**0 fewer per 1.000** (from 0 fewer to 0 fewer)	⊕◯◯◯ VERY LOW	IMPORTANT
**Mortality**												
1	observational studies	serious [e]	not serious	not serious	not serious	none	0/40 (0.0%)	0/40 (0.0%)	**RD 0.00** (-0.05 to 0.05)	**-- per 1.000** (from to --)	⊕◯◯◯ VERY LOW	CRITICAL
**Hypertension remission (follow up: range 4 months to 3 years)**												
8	observational studies	very serious	very serious [f]	serious [a]	very serious	none	11/233 (4.7%)	8/189 (4.2%)	**RD 0.14** (-0.57 to 0.29)	**-- per 1.000** (from to --)	⊕◯◯◯ VERY LOW	IMPORTANT
**Hypertension improvement (follow up: range 4 months to 3 years)**												

8	observational studies	very serious [a]	very serious [f]	serious [a]	very serious	none	10/233 (4.3%)	7/189 (3.7%)	**RD 0.08** (-0.47 to 0.64)	**-- per 1.000** (from to --)	⊕◯◯◯ VERY LOW	IMPORTANT
**OSAS remission (follow up: range 4 months to 3 years)**												
8	observational studies	very serious [a]	not serious	serious [a]	very serious	none	5/233 (2.1%)	2/189 (1.1%)	**RD 0.45** (-1.00 to 0.10)	**-- per 1.000** (from to --)	⊕◯◯◯ VERY LOW	IMPORTANT
**OSAS improvement (follow up: range 4 months to 3 years)**												
8	observational studies	very serious [a]	not serious	serious [a]	very serious	none	1/233 (0.4%)	3/189 (1.6%)	**RD 0.45** (0.10 to 1.00)	**-- per 1.000** (from to --)	⊕◯◯◯ VERY LOW	IMPORTANT
**T2DM remission (follow up: range 3 months to 18 months)**												
1	observational studies	very serious [g]	not serious	serious [g]	not serious	none	0/84 (0.0%)	0/43 (0.0%)	**OR 0.41** (0.16 to 1.04)	**0 fewer per 1.000** (from 0 fewer to 0 fewer)	⊕◯◯◯ VERY LOW	IMPORTANT

CI: Confidence interval; **MD:** Mean difference; **OR:** Odds ratio
