# Supplementary material for: Clinical practice guidelines of the European Association for Endoscopic Surgery (EAES) on bariatric surgery: update 2020 endorsed by IFSO-EC, EASO and ESPCOP
Source: Surg Endosc. 2020 Apr 23;34(6):2332–58. doi: 10.1007/s00464-020-07555-y (PMC7214495; doi:10.1007/s00464-020-07555-y)
Supplement: Supplementary file 21 — Supplementary file21 (PDF 115 kb) [file 464_2020_7555_MOESM21_ESM.pdf]

Question: Should staple line reinforcement vs. no staple line reinforcement be used in patients undergoing sleeve gastrectomy?

Certainty assessment							№ of patients		Effect		Certainty	Importance
**№ of studies**	**Study design**	**Risk of bias**	**Inconsistency**	**Indirectness**	**Imprecision**	**Other considerations**	**staple line reinforcement**	**no staple line reinforcement**	**Relative (95% CI)**	**Absolute (95% CI)**		
**overall complications**												
8	randomised trials	not serious	not serious	not serious	not serious	none			**RR 0.7** (0.5 to 0.9)	**1 fewer per 1.000** (from 1 fewer to 1 fewer)	⊕⊕⊕⊕ HIGH	CRITICAL
**Bleeding**												
8	randomised trials	not serious	not serious	not serious	serious	none			**OR 0.56** (0.25 to 1.27)	**1 fewer per 1.000** (from 1 fewer to 0 fewer)	⊕⊕⊕◯ MODERATE	CRITICAL
**leak**												
8	randomised trials	not serious	serious	not serious	serious	none			**RR 0.60** (0.27 to 1.50)	**1 fewer per 1.000** (from 2 fewer to 0 fewer)	⊕⊕◯◯ LOW	CRITICAL

CI: Confidence interval; **RR:** Risk ratio; **OR:** Odds ratio
