# Supplementary material for: Clinical practice guidelines of the European Association for Endoscopic Surgery (EAES) on bariatric surgery: update 2020 endorsed by IFSO-EC, EASO and ESPCOP
Source: Surg Endosc. 2020 Apr 23;34(6):2332–58. doi: 10.1007/s00464-020-07555-y (PMC7214495; doi:10.1007/s00464-020-07555-y)
Supplement: Supplementary file 22 — Supplementary file22 (PDF 112 kb) [file 464_2020_7555_MOESM22_ESM.pdf]

Question: Should sleeve gastrectomy calibrated on bougie size <=36 Fr vs. > 36 Fr be used for weight loss?

Certainty assessment							№ of patients		Effect		Certainty	Importance
**№ of studies**	**Study design**	**Risk of bias**	**Inconsistency**	**Indirectness**	**Imprecision**	**Other considerations**	**sleeve gastrectomy calibrated on bougie size <=36 Fr**	**> 36 Fr**	**Relative (95% CI)**	**Absolute (95% CI)**		
**Weight loss (follow up: range 12 months to 60 months)**												
4	observational studies	not serious	not serious	not serious	not serious	strong association			**OR 0.23** (0.14 to 0.33)	**0 fewer per 1.000** (from 0 fewer to 0 fewer)	⨁⨁⨁◯ MODERATE	CRITICAL
**Leaks (follow up: range 12 months to 60 months)**												
7	observational studies	not serious	not serious	serious	serious	none			**OR 0.91** (0.67 to 1.24)	**1 fewer per 1.000** (from 1 fewer to 1 fewer)	⨁◯◯◯ VERY LOW	CRITICAL
**GERD (follow up: range 12 months to 60 months)**												
3	observational studies	not serious	serious	serious	serious	none			**OR 0.77** (0.37 to 1.59)	**1 fewer per 1.000** (from 2 fewer to 0 fewer)	⨁◯◯◯ VERY LOW	IMPORTANT
**Overall complications (follow up: range 12 months to 60 months)**												
8	observational studies	not serious	serious	serious	serious	none			**OR 1.00** (0.73 to 1.37)	**1 fewer per 1.000** (from 1 fewer to 1 fewer)	⨁◯◯◯ VERY LOW	CRITICAL

CI: Confidence interval; **OR:** Odds ratio
