# Supplementary material for: Clinical practice guidelines of the European Association for Endoscopic Surgery (EAES) on bariatric surgery: update 2020 endorsed by IFSO-EC, EASO and ESPCOP
Source: Surg Endosc. 2020 Apr 23;34(6):2332–58. doi: 10.1007/s00464-020-07555-y (PMC7214495; doi:10.1007/s00464-020-07555-y)
Supplement: Supplementary file 23 — Supplementary file23 (PDF 71 kb) [file 464_2020_7555_MOESM23_ESM.pdf]

Question: Should sleeve gastrectomy with antral resection (2-3 cm) vs. antral preservation (>5 cm) be used for weight loss?

Certainty assessment							№ of patients		Effect		Certainty	Importance
№ of studies	Study design	Risk of bias	Inconsistency	Indirectness	Imprecision	Other considerations	sleeve gastrectomy with antral resection (2-3 cm)	antral preservation (>5 cm)	Relative (95% CI)	Absolute (95% CI)		
**Weight loss (follow up: mean 24 months)**												
4	observational studies	serious	serious	serious	serious	none	0	0	-	MD **0.95 higher** (0.32 higher to 1.58 higher)	⨁◯◯◯ VERY LOW	CRITICAL
**Bibliography: staple line leak**												
4	observational studies	serious	serious	serious	serious	none	0/0	0/0	**RR 1.87** (0.46 to 7.61)	**2 fewer per 1.000** (from 8 fewer to 0 fewer)	⨁◯◯◯ VERY LOW	CRITICAL
**Staple line bleed**												
5	observational studies	serious	serious	serious	serious	none	0/0	0/0	**RR 1.27** (0.40 to 4.01)	**1 fewer per 1.000** (from 4 fewer to 0 fewer)	⨁◯◯◯ VERY LOW	CRITICAL
**gerd**												
3	observational studies	serious	serious	serious	not serious	none	0/0	0/0	**RR 0.69** (0.26 to 1.82)	**1 fewer per 1.000** (from 2 fewer to 0 fewer)	⨁◯◯◯ VERY LOW	IMPORTANT

CI: Confidence interval; **MD:** Mean difference; **RR:** Risk ratio
