# Supplementary material for: Clinical practice guidelines of the European Association for Endoscopic Surgery (EAES) on bariatric surgery: update 2020 endorsed by IFSO-EC, EASO and ESPCOP
Source: Surg Endosc. 2020 Apr 23;34(6):2332–58. doi: 10.1007/s00464-020-07555-y (PMC7214495; doi:10.1007/s00464-020-07555-y)
Supplement: Supplementary file 24 — Supplementary file24 (PDF 67 kb) [file 464_2020_7555_MOESM24_ESM.pdf]

Question: Should RYGB vs. LAGB be used for weight loss in obese patients?

Certainty assessment							№ of patients		Effect		Certainty	Importance
**№ of studies**	**Study design**	**Risk of bias**	**Inconsistency**	**Indirectness**	**Imprecision**	**Other considerations**	**RYGB**	**LAGB**	**Relative (95% CI)**	**Absolute (95% CI)**		
**EWL (follow up: range 1 years to 5 years; assessed with: %)**												
11	randomised trials	serious	not serious	not serious	not serious a	strong association	0	0	-	MD **22 % higher** (6.5 higher to 34 higher)	⨁⨁⨁⨁ HIGH	IMPORTANT
**T2DM (follow up: range 3 months to 5 years)**												
25	randomised trials	serious	not serious	not serious	serious [b]	none	5/24 (20.8%)	2/23 (8.7%)	**RR 1.96** (0.47 to 8.33)	**83 more per 1.000** (from 46 fewer to 637 more)	⨁⨁◯◯ LOW	IMPORTANT

CI: Confidence interval; **MD:** Mean difference; **RR:** Risk ratio
