# Supplementary material for: Clinical practice guidelines of the European Association for Endoscopic Surgery (EAES) on bariatric surgery: update 2020 endorsed by IFSO-EC, EASO and ESPCOP
Source: Surg Endosc. 2020 Apr 23;34(6):2332–58. doi: 10.1007/s00464-020-07555-y (PMC7214495; doi:10.1007/s00464-020-07555-y)
Supplement: Supplementary file 25 — Supplementary file25 (PDF 63 kb) [file 464_2020_7555_MOESM25_ESM.pdf]

Question: Should RYGB vs. gastric plication be used for weight loss in obese patients?

Certainty assessment							№ of patients		Effect		Certainty	Importance
№ of studies	Study design	Risk of bias	Inconsistency	Indirectness	Imprecision	Other considerations	RYGB	gastric plication	Relative (95% CI)	Absolute (95% CI)		
**T2DM (follow up: range 3 months to 5 years)**												
25	randomised trials	serious [a]	not serious	not serious	serious	none			**RR 4.00** (1.40 to 11.11)	**4 fewer per 1.000** (from 11 fewer to 1 fewer)	⨁⨁◯◯ LOW	IMPORTANT

CI: Confidence interval; **RR:** Risk ratio
