# Supplementary material for: Clinical practice guidelines of the European Association for Endoscopic Surgery (EAES) on bariatric surgery: update 2020 endorsed by IFSO-EC, EASO and ESPCOP
Source: Surg Endosc. 2020 Apr 23;34(6):2332–58. doi: 10.1007/s00464-020-07555-y (PMC7214495; doi:10.1007/s00464-020-07555-y)
Supplement: Supplementary file 26 — Supplementary file26 (PDF 34 kb) [file 464_2020_7555_MOESM26_ESM.pdf]

Author(s): It includes GERD recommendation
Question: RYGB compared to sleeve gastrectomy for weight loss in obese patients
Setting: Question 6
Bibliography:

Certainty assessment							№ of patients		Effect		Certainty	Importance
**№ of studies**	**Study design**	**Risk of bias**	**Inconsistency**	**Indirectness**	**Imprecision**	**Other considerations**	**RYGB**	**sleeve gastrectomy**	**Relative (95% CI)**	**Absolute (95% CI)**		
**EWL (follow up: range 1 years to 5 years; assessed with: %)**												
11	randomised trials	serious	not serious	not serious	not serious	none			-	MD **4 % higher** (14 lower to 8.2 higher)	⨁⨁⨁◯ MODERATE	CRITICAL
**T2DM (follow up: range 3 months to 5 years)**												
25	randomised trials	serious	not serious	not serious	not serious	none	117/226 (51.8%)	104/223 (46.6%)	**RR 0.89** (0.74 to 1.06)	**51 fewer per 1.000** (from 121 fewer to 28 more)	⨁⨁⨁◯ MODERATE	IMPORTANT
**Long-term major complications (follow up: range 1 months to 5 years)**												
6	randomised trials	serious	serious	not serious	serious	none	6/345 (1.7%)	3/350 (0.9%)	**OR 1.56** (0.51 to 4.76)	**5 more per 1.000** (from 4 fewer to 31 more)	⨁◯◯◯ VERY LOW	CRITICAL
**Long term minor complications (follow up: range 1 months to 5 years)**												
6	randomised trials	serious	serious	not serious	not serious	none	17/205 (8.3%)	10/213 (4.7%)	**OR 1.56** (0.68 to 3.57)	**24 more per 1.000** (from 15 fewer to 103 more)	⨁⨁◯◯ LOW	IMPORTANT
**Hypertension remission (follow up: range 2 months to 5 years)**												
21	observational studies	serious	serious	serious	not serious	none	552/920 (60.0%)	323/632 (51.1%)	**OR 0.70** (0.86 to 0.87)	**89 fewer per 1.000** (from 38 fewer to 35 fewer)	⨁◯◯◯ VERY LOW	IMPORTANT
**Dyslipidemia resolution (follow up: range 2 months to 5 years)**												

14	observational studies	serious	serious	serious	not serious	none	489/689 (71.0%)	287/580 (49.5%)	**OR 0.42** (0.33 to 0.53)	**203 fewer per 1.000** (from 251 fewer to 153 fewer)	⨁◯◯◯ VERY LOW	IMPORTANT
**GERD (follow up: range 2 months to 5 years)**												
11	observational studies	serious	serious	serious	not serious	strong association	109/147 (74.1%)	26/93 (28.0%)	**OR 0.11** (0.06 to 0.21)	**239 fewer per 1.000** (from 257 fewer to 204 fewer)	⨁◯◯◯ VERY LOW	IMPORTANT
**OSAS (follow up: range 2 months to 5 years)**												
11	observational studies	serious	not serious	serious	not serious	none	205/270 (75.9%)	160/247 (64.8%)	**OR 0.44** (0.19 to 1.04)	**201 fewer per 1.000** (from 389 fewer to 9 more)	⨁◯◯◯ VERY LOW	IMPORTANT
**Minor operative morbidity (follow up: range 1 months to 5 years)**												
6	randomised trials	serious [a]	serious [b]	not serious	not serious	none	28/316 (8.9%)	19/317 (6.0%)	**OR 1.43** (0.60 to 3.23)	**24 more per 1.000** (from 23 fewer to 111 more)	⨁⨁◯◯ LOW	IMPORTANT
**Major operative morbidity (follow up: range 1 months to 5 years)**												
6	randomised trials	serious [a]	serious [b]	not serious	not serious	none	26/346 (7.5%)	12/347 (3.5%)	**OR 2.04** (1.00 to 4.16)	**34 more per 1.000** (from 0 fewer to 95 more)	⨁⨁◯◯ LOW	CRITICAL
**GERD remission in pre-existing GERD (follow up: 5 years)**												
1	randomised trials	serious	not serious	not serious	not serious	none	104	101	-	AD **0.36 % lower** (0.57 lower to 0.15 lower)	⨁⨁⨁◯ MODERATE	IMPORTANT
**De novo GERD (follow up: 5 years)**												

1	randomised trials	serious	not serious	not serious	serious	none	56	57	-	AD **0.31 % lower** (0.08 lower to 0.54 lower)	⨁⨁◯◯ LOW	IMPORTANT

CI: Confidence interval; **MD:** Mean difference; **RR:** Risk ratio; **OR:** Odds ratio

Explanations

a. Heterogenity between the studies
b. Inconsistency between the studies
