# Supplementary material for: Clinical practice guidelines of the European Association for Endoscopic Surgery (EAES) on bariatric surgery: update 2020 endorsed by IFSO-EC, EASO and ESPCOP
Source: Surg Endosc. 2020 Apr 23;34(6):2332–58. doi: 10.1007/s00464-020-07555-y (PMC7214495; doi:10.1007/s00464-020-07555-y)
Supplement: Supplementary file 27 — Supplementary file27 (PDF 68 kb) [file 464_2020_7555_MOESM27_ESM.pdf]

Question: Should BPD/DS vs. sleeve gastrectomy be used for weight loss?

Certainty assessment							№ of patients		Effect		Certainty	Importance
**№ of studies**	**Study design**	**Risk of bias**	**Inconsistency**	**Indirectness**	**Imprecision**	**Other considerations**	**BPD/DS**	**sleeve gastrectomy**	**Relative (95% CI)**	**Absolute (95% CI)**		
**30-day morbidity**												
2	observational studies	not serious	not serious	not serious	very serious [a]	none	10/267 (3.7%)	6/674 (0.9%)	**OR 5.10** (0.24 to 107.62)	**35 more per 1.000** (from 7 fewer to 483 more)	⨁◯◯◯ VERY LOW	CRITICAL
**30-day mortality**												
2	observational studies	not serious	not serious	not serious	very serious [a]	none	0/267 (0.0%)	0/674 (0.0%)	not estimable	**0 fewer per 1.000** (from 10 fewer to 10 more)	⨁◯◯◯ VERY LOW	CRITICAL
**Overall mortality**												
1	observational studies	not serious	not serious	not serious	very serious [b]	none	0/100 (0.0%)	0/100 (0.0%)	**RD 0.00** (-0.01 to 0.01)	**0 fewer per 1.000** (from 20 fewer to 20 more)	⨁◯◯◯ VERY LOW	CRITICAL

CI: Confidence interval; **OR:** Odds ratio
