# Supplementary material for: Clinical practice guidelines of the European Association for Endoscopic Surgery (EAES) on bariatric surgery: update 2020 endorsed by IFSO-EC, EASO and ESPCOP
Source: Surg Endosc. 2020 Apr 23;34(6):2332–58. doi: 10.1007/s00464-020-07555-y (PMC7214495; doi:10.1007/s00464-020-07555-y)
Supplement: Supplementary file 28 — Supplementary file28 (PDF 135 kb) [file 464_2020_7555_MOESM28_ESM.pdf]

Question: Should BPD/DS vs. RYGB be used for weight loss?

Certainty assessment							№ of patients		Effect		Certainty	Importance
№ of studies	Study design	Risk of bias	Inconsistency	Indirectness	Imprecision	Other considerations	BPD/DS	RYGB	Relative (95% CI)	Absolute (95% CI)		
**3 year weight loss**												
2	randomised trials	not serious	very serious	serious [a]	very serious [b]	none	44	43	-	MD **14.97 higher** (12.21 lower to 42.15 higher)	⊕◯◯◯ VERY LOW	CRITICAL
**30-day morbidity**												
3	randomised trials	not serious [c]	serious [a]	serious [a]	very serious [b]	all plausible residual confounding would reduce the demonstrated effect	17/66 (25.8%)	12/80 (15.0%)	**OR 2.24** (0.67 to 7.55)	**133 more per 1.000** (from 44 fewer to 421 more)	⊕◯◯◯ VERY LOW	CRITICAL
**Long term morbidity**												
2	randomised trials	not serious	not serious	serious [a]	serious [d]	none	13/49 (26.5%)	6/55 (10.9%)	**OR 3.38** (1.14 to 10.05)	**184 more per 1.000** (from 13 more to 443 more)	⊕⊕◯◯ LOW	CRITICAL
**30-day mortality**												
2	randomised trials	not serious	not serious	serious [a,b]	very serious [b]	none	1/44 (2.3%)	0/43 (0.0%)	**RR 0.02** (-0.05 to 0.10)	**0 fewer per 1.000** (from 90 fewer to 90 more)	⊕◯◯◯ VERY LOW	CRITICAL
**T2DM remission**												
2	randomised trials	not serious [a,d]	not serious [a,d]	serious [a,b]	very serious [b]	very strong association [a,d]	43/44 (97.7%)	34/43 (79.1%)	**OR 8.06** (1.35 to 48.14)	**178 more per 1.000** (from 45 more to 204 more)	⊕⊕⊕◯ MODERATE	IMPORTANT
**Hypertension remission**												

1	randomised trials	not serious	not serious	serious [a,b]	very serious [b]	none	17/20 (85.0%)	16/20 (80.0%)	**OR 1.42** (0.27 to 7.34)	**50 more per 1.000** (from 281 fewer to 167 more)	⨁◯◯◯ VERY LOW	IMPORTANT
**Dyslipidemia remission**												
1	randomised trials	not serious	not serious	serious [a]	very serious [b]	none	18/20 (90.0%)	17/20 (85.0%)	**OR 1.59** (0.24 to 10.70)	**50 more per 1.000** (from 274 fewer to 134 more)	⨁◯◯◯ VERY LOW	IMPORTANT
**Iron deficiency anemia**												
2	randomised trials	not serious	not serious	serious [a]	very serious [b]	none	4/44 (9.1%)	5/43 (11.6%)	**OR 0.76** (0.19 to 3.04)	**25 fewer per 1.000** (from 92 fewer to 169 more)	⨁◯◯◯ VERY LOW	IMPORTANT
**Hypoalbuminemia**												
2	randomised trials	not serious	not serious	serious [a]	very serious [b]	none	2/44 (4.5%)	0/43 (0.0%)	**OR 0.05** (-0.04 to 0.13)	**0 fewer per 1.000** (from 0 fewer to 0 fewer)	⨁◯◯◯ VERY LOW	IMPORTANT
**Overall Mortality**												
1	randomised trials	very serious [c]	very serious [c]	very serious [b,c]	very serious [b,c]	publication bias strongly suspected [b,c]	1/24 (4.2%)	0/23 (0.0%)	**OR 0.04** (-0.07 to 0.15)	**0 fewer per 1.000** (from 0 fewer to 0 fewer)	⨁◯◯◯ VERY LOW	CRITICAL
**Weight Regain**												

1	randomised trials	not serious [b,c]	not serious	not serious	very serious [c]	none	0/24 (0.0%)	0/23 (0.0%)	**OR 0.00** (-0.08 to 0.08)	**-- per 1.000** (from 0 fewer to 0 fewer)	⨁⨁◯◯ LOW	IMPORTANT

CI: Confidence interval; **MD:** Mean difference; **OR:** Odds ratio; **RR:** Risk ratio
