# Supplementary material for: Clinical practice guidelines of the European Association for Endoscopic Surgery (EAES) on bariatric surgery: update 2020 endorsed by IFSO-EC, EASO and ESPCOP
Source: Surg Endosc. 2020 Apr 23;34(6):2332–58. doi: 10.1007/s00464-020-07555-y (PMC7214495; doi:10.1007/s00464-020-07555-y)
Supplement: Supplementary file 29 — Supplementary file29 (PDF 101 kb) [file 464_2020_7555_MOESM29_ESM.pdf]

**Question**: Should OAGB vs. RYGB be used for weight loss?

| Certainty assessment | | | | | | | № of patients | | Effect | | Certainty | Importance |
|---|---|---|---|---|---|---|---|---|---|---|---|---|
| № of studies | Study design | Risk of bias | Inconsistency | Indirectness | Imprecision | Other considerations | OAGB | RYGB | Relative (95% CI) | Absolute (95% CI) | | |
| **EWL** | | | | | | | | | | | | |
| 2 | randomised trials | serious | very serious | not serious | serious | strong association | 220 | 224 | - | MD **13.23 higher** (2.05 lower to 28.51 higher) | ⨁◯◯◯ VERY LOW | |
