# Supplementary material for: Clinical practice guidelines of the European Association for Endoscopic Surgery (EAES) on bariatric surgery: update 2020 endorsed by IFSO-EC, EASO and ESPCOP
Source: Surg Endosc. 2020 Apr 23;34(6):2332–58. doi: 10.1007/s00464-020-07555-y (PMC7214495; doi:10.1007/s00464-020-07555-y)
Supplement: Supplementary file 31 — Supplementary file31 (PDF 69 kb) [file 464_2020_7555_MOESM31_ESM.pdf]

Question: Should OAGB vs. sleeve gastrectomy be used for weight loss?

Certainty assessment							№ of patients		Effect		Certainty	Importance
**№ of studies**	**Study design**	**Risk of bias**	**Inconsistency**	**Indirectness**	**Imprecision**	**Other considerations**	**OAGB**	**LAGB**	**Relative (95% CI)**	**Absolute (95% CI)**		
**Post-operative BMI (follow up: mean 1 years; assessed with: %)**												
4	observational studies	serious	very serious	not serious	not serious	publication bias strongly suspected	0/0	0.0%	**MD -6.58** (-9.37 to -3.79)	**-- per 1.000** (from -to --)	⨁◯◯◯ VERY LOW	
**T2DM remission (follow up: mean 1 years)**												
2	observational studies	serious	not serious	not serious	not serious	none	0/0	0.0%	**RR 1.48** (0.98 to 2.25)	**0 fewer per 1.000** (from 0 fewer to 0 fewer)	⨁◯◯◯ VERY LOW	
**Post-operative Waist Circumference (follow up: mean 1 years)**												
3	observational studies	serious	very serious	not serious	serious	strong association	0/0	0.0%	**MD -14.15** (-27.23 to -1.06)	**-- per 1.000** (from -to --)	⨁◯◯◯ VERY LOW	

CI: Confidence interval; **RR:** Risk ratio
