# Supplementary material for: Clinical practice guidelines of the European Association for Endoscopic Surgery (EAES) on bariatric surgery: update 2020 endorsed by IFSO-EC, EASO and ESPCOP
Source: Surg Endosc. 2020 Apr 23;34(6):2332–58. doi: 10.1007/s00464-020-07555-y (PMC7214495; doi:10.1007/s00464-020-07555-y)
Supplement: Supplementary file 32 — Supplementary file32 (PDF 73 kb) [file 464_2020_7555_MOESM32_ESM.pdf]

Question: Should OAGB vs. gastric plication be used for weight loss?

Certainty assessment							№ of patients		Effect		Certainty	Importance
**№ of studies**	**Study design**	**Risk of bias**	**Inconsistency**	**Indirectness**	**Imprecision**	**Other considerations**	**OAGB**	**gastric plication**	**Relative (95% CI)**	**Absolute (95% CI)**		
**EWL**												
1	randomised trials	serious	not serious	not serious	serious	none	20	20	-	MD **6.1 higher** (6.58 lower to 18.78 higher)	⨁⨁◯◯ LOW	
**Iron deficiency anemia**												
1	randomised trials	serious	not serious	serious	very serious	strong association	4/20 (20.0%)	0/20 (0.0%)	**OR 11.18** (0.56 to 222.98)	**0 fewer per 1.000** (from 0 fewer to 0 fewer)	⨁◯◯◯ VERY LOW	

CI: Confidence interval; **MD:** Mean difference; **OR:** Odds ratio
