# Supplementary material for: Clinical practice guidelines of the European Association for Endoscopic Surgery (EAES) on bariatric surgery: update 2020 endorsed by IFSO-EC, EASO and ESPCOP
Source: Surg Endosc. 2020 Apr 23;34(6):2332–58. doi: 10.1007/s00464-020-07555-y (PMC7214495; doi:10.1007/s00464-020-07555-y)
Supplement: Supplementary file 33 — Supplementary file33 (PDF 70 kb) [file 464_2020_7555_MOESM33_ESM.pdf]

Question: Should SADI-S vs. RYGB be used for weight loss?

Certainty assessment							№ of patients		Effect		Certainty	Importance
**№ of studies**	**Study design**	**Risk of bias**	**Inconsistency**	**Indirectness**	**Imprecision**	**Other considerations**	**SADI-S**	**RYGB**	**Relative (95% CI)**	**Absolute (95% CI)**		
**Weight loss (follow up: 18 months; assessed with: %)**												
1	observational studies	serious	not serious	not serious	serious	none	SADI-S: 41, 95% CI 39.3 to 42.7; RYGB: 39.6, 95% CI 36.6 to 42.6				⊕◯◯◯ VERY LOW	CRITICAL
**Morbidity (follow up: 30 days)**												
1	observational studies	serious	not serious	not serious	serious	none	9/54 (16.7%)	34/54 (63.0%)	**OR 0.12** (0.05 to 0.29)	**460 fewer per 1.000** (from 551 fewer to 299 fewer)	⊕◯◯◯ VERY LOW	CRITICAL
**Mortality (follow up: 30 days)**												
1	observational studies	serious	not serious	not serious	not serious	none	0/54 (0.0%)	0/54 (0.0%)	**RD 0.00** (-0.04 to 0.04)	**0 fewer per 1.000** (from 40 fewer to 40 more)	⊕◯◯◯ VERY LOW	CRITICAL

CI: Confidence interval; **OR:** Odds ratio
