# Supplementary material for: Clinical practice guidelines of the European Association for Endoscopic Surgery (EAES) on bariatric surgery: update 2020 endorsed by IFSO-EC, EASO and ESPCOP
Source: Surg Endosc. 2020 Apr 23;34(6):2332–58. doi: 10.1007/s00464-020-07555-y (PMC7214495; doi:10.1007/s00464-020-07555-y)
Supplement: Supplementary file 34 — Supplementary file34 (PDF 108 kb) [file 464_2020_7555_MOESM34_ESM.pdf]

Question: Should SADI-S vs. BPD/DS be used for weight loss?

Certainty assessment							№ of patients		Effect		Certainty	Importance
№ of studies	Study design	Risk of bias	Inconsistency	Indirectness	Imprecision	Other considerations	SADI-S	BPD/DS	Relative (95% CI)	Absolute (95% CI)		
**EWL (follow up: 24 months; assessed with: %)**												
1	observational studies	serious	not serious	not serious	not serious	none	SADI-S: 87.1, 95% CI 83.8 to 90.3; BPD/DS: 94.9%, 95% CI 91.6 to 98.2				⨁◯◯◯ VERY LOW	CRITICAL
**Short term morbidity (follow up: 30 days)**												
1	observational studies	serious	not serious	not serious	very serious	very strong association	13/62 (21.0%)	2/120 (1.7%)	**OR 15.65** (3.40 to 71.96)	**193 more per 1.000** (from 38 more to 533 more)	⨁◯◯◯ VERY LOW	CRITICAL
