# Supplementary material for: Clinical practice guidelines of the European Association for Endoscopic Surgery (EAES) on bariatric surgery: update 2020 endorsed by IFSO-EC, EASO and ESPCOP
Source: Surg Endosc. 2020 Apr 23;34(6):2332–58. doi: 10.1007/s00464-020-07555-y (PMC7214495; doi:10.1007/s00464-020-07555-y)
Supplement: Supplementary file 35 — Supplementary file35 (PDF 135 kb) [file 464_2020_7555_MOESM35_ESM.pdf]

Question: Should SADI-S vs. sleeve gastrectomy be used for weight loss?

Certainty assessment							№ of patients		Effect		Certainty	Importance
№ of studies	Study design	Risk of bias	Inconsistency	Indirectness	Imprecision	Other considerations	SADI-S	sleeve gastrectomy	Relative (95% CI)	Absolute (95% CI)		
**EWL (follow up: 12 months; assessed with: %)**												
1	observational studies	serious	not serious	not serious	serious	none	26	29	-	MD **19.7 higher** (8.33 higher to 31.07 higher)	⨁◯◯◯ VERY LOW	IMPORTANT
**Mortality (follow up: 30 days)**												
1	observational studies	serious	not serious	not serious	serious	none	0/89 (0.0%)	0/89 (0.0%)	**RD 0.00** (-0.02 to 0.02)	**0 fewer per 1.000** (from 20 fewer to 20 more)	⨁◯◯◯ VERY LOW	CRITICAL
**Morbidity (follow up: 30 days)**												
1	observational studies	serious	not serious	not serious	serious	none	13/89 (14.6%)	8/89 (9.0%)	**OR 1.73** (0.68 to 4.41)	**56 more per 1.000** (from 27 fewer to 214 more)	⨁◯◯◯ VERY LOW	CRITICAL
**HbA1c (follow up: 12 months; assessed with: %)**												
1	observational studies	serious	not serious	not serious	not serious	none	26	26	-	MD **0** (0.38 lower to 0.38 higher)	⨁◯◯◯ VERY LOW	IMPORTANT
**Hypertension (follow up: 12 months)**												

1	observational studies	serious	not serious	not serious	very serious	none	2/26 (7.7%)	4/29 (13.8%)	**OR 0.52** (0.09 to 3.11)	**61 fewer per 1.000** (from 124 fewer to 194 more)	⨁◯◯◯ VERY LOW	IMPORTANT
**Dyslipidemia (follow up: 12 months)**												
1	observational studies	serious	not serious	not serious	very serious	none	1/26 (3.8%)	6/29 (20.7%)	**OR 0.15** (0.02 to 1.37)	**169 fewer per 1.000** (from 202 fewer to 56 more)	⨁◯◯◯ VERY LOW	IMPORTANT
**SBP (follow up: 12 months; assessed with: mmHg)**												
1	observational studies	serious	not serious	not serious	not serious	none	26	29	-	MD **1 higher** (8.25 lower to 10.25 higher)	⨁◯◯◯ VERY LOW	IMPORTANT
**DBP (follow up: 12 months; assessed with: mmHg)**												
1	observational studies	serious	not serious	not serious	not serious	none	26	29	-	MD **2.5 higher** (4.81 lower to 9.81 higher)	⨁◯◯◯ VERY LOW	IMPORTANT
**Triglycerides (follow up: 12 months; assessed with: mg/dL)**												
1	observational studies	serious	not serious	not serious	very serious	none	26	29	-	MD **17.1 lower** (33.57 lower to 0.63 lower)	⨁◯◯◯ VERY LOW	IMPORTANT
**HDL (follow up: 12 months; assessed with: mg/dL)**												
1	observational studies	serious	not serious	not serious	not serious	none	26	29	-	MD **1.9 higher** (3.95 lower to 7.75 higher)	⨁◯◯◯ VERY LOW	IMPORTANT
**LDL (follow up: 12 months; assessed with: mg/dL)**												

1	observational studies	serious	not serious	not serious	serious	none	26	29	-	MD **14.4 lower** (30.19 lower to 1.39 higher)	⨁◯◯◯ VERY LOW	IMPORTANT
**Total cholesterol (follow up: 12 months; assessed with: mg/dL)**												
1	observational studies	serious	not serious	not serious	not serious	none	26	29	-	MD **11.1 lower** (27 lower to 4.8 higher)	⨁◯◯◯ VERY LOW	IMPORTANT
**Iron (follow up: 12 months; assessed with: ug/dL)**												
1	observational studies	serious	not serious	not serious	serious	strong association	26	29	-	MD **34.1 lower** (59 lower to 9.2 lower)	⨁◯◯◯ VERY LOW	IMPORTANT
**Hemoglobin (follow up: 12 months; assessed with: g/dL)**												
1	observational studies	serious	not serious	not serious	serious	none	26	29	-	MD **0.9 lower** (2.01 lower to 0.21 higher)	⨁◯◯◯ VERY LOW	IMPORTANT
**Albumin (follow up: 12 months; assessed with: mg/dL)**												
1	observational studies	serious	not serious	not serious	not serious	none	26	29	-	MD **0** (0.13 lower to 0.13 higher)	⨁◯◯◯ VERY LOW	IMPORTANT
**Calcium (follow up: 12 months; assessed with: mg/dL)**												
1	observational studies	serious	not serious	not serious	very serious	none	26	29	-	MD **0.2 lower** (0.39 lower to 0.01 lower)	⨁◯◯◯ VERY LOW	IMPORTANT

CI: Confidence interval; **MD:** Mean difference; **OR:** Odds ratio
