# Supplementary material for: Clinical practice guidelines of the European Association for Endoscopic Surgery (EAES) on bariatric surgery: update 2020 endorsed by IFSO-EC, EASO and ESPCOP
Source: Surg Endosc. 2020 Apr 23;34(6):2332–58. doi: 10.1007/s00464-020-07555-y (PMC7214495; doi:10.1007/s00464-020-07555-y)
Supplement: Supplementary file 36 — Supplementary file36 (DOCX 46 kb) [file 464_2020_7555_MOESM36_ESM.docx]

**Supplementary Table 29.** Nomenclature proposed by ASMBS Revision Task Force for dividing re-operative procedures according to their technical aspects.^124^

| *Conversion*: | Procedures that change from an index procedure to a different type of procedure. |
| --- | --- |
| *Corrective*: | Procedures addressing complications or incomplete treatment effect of a previous bariatric operation. |
| *Reversal*: | Procedures that restore original anatomy. |
