# Supplementary material for: Clinical practice guidelines of the European Association for Endoscopic Surgery (EAES) on bariatric surgery: update 2020 endorsed by IFSO-EC, EASO and ESPCOP
Source: Surg Endosc. 2020 Apr 23;34(6):2332–58. doi: 10.1007/s00464-020-07555-y (PMC7214495; doi:10.1007/s00464-020-07555-y)
Supplement: Supplementary file 37 — Supplementary file37 (DOCX 88 kb) [file 464_2020_7555_MOESM37_ESM.docx]

**Table 2.** Proposed standardization of the timing and diagnostic criteria for the definition of non-responder after primary bariatric/metabolic surgery.

| *Primary non-responder* | *Timing:*  Insufficient weight loss can be detected early after the index procedure in patients with weight loss clearly lower than expected. Early detection may offer the opportunity for additional interventions (intensive lifestyle modifications, behavioral programs, weight-loss medication). However, for the purposes of revisional surgery, the patients should be evaluated at the nadir of the curve of weight loss following the index procedure, not earlier than 18-24 months.  *Criteria:*  Patients could be defined as primary non-responders in the presence of at least one of the following:   1. Weight loss lower than 10% of the baseline body weight 2. Weight loss not sufficient to remove patients from the index classification as obese as an indication for primary bariatric surgery 3. Weight loss not sufficient to permit adequate control of baseline comorbidities with medical therapy, including type 2 diabetes. |
| --- | --- |
| *Secondary non-responder* | *Timing:*  Weight regain after a successful primary procedure should be evaluated after the expected time for weight stabilization, taking into account that a minimal level of weight regain after the nadir should be considered as normal, at least 24 months after the index procedure.  *Criteria:*  Patients could be defined as secondary non-responders in the presence of at least one of the following:   1. Ongoing progressive weight regain 2. Weight regain sufficient to re-classify as obese as an indication for primary bariatric surgery 3. Weight regain accompanied by inadequate control of baseline co-morbidities with medical therapy, including type 2 diabetes. |
