# Supplementary material for: Clinical practice guidelines of the European Association for Endoscopic Surgery (EAES) on bariatric surgery: update 2020 endorsed by IFSO-EC, EASO and ESPCOP
Source: Surg Endosc. 2020 Apr 23;34(6):2332–58. doi: 10.1007/s00464-020-07555-y (PMC7214495; doi:10.1007/s00464-020-07555-y)
Supplement: Supplementary file 38 — Supplementary file38 (PDF 103 kb) [file 464_2020_7555_MOESM38_ESM.pdf]

Question: Should scheduled post-operative follow up vs. no post-operative follow up be used in patients undergoing bariatric surgery?

Certainty assessment							№ of patients		Effect		Certainty	Importance
№ of studies	Study design	Risk of bias	Inconsistency	Indirectness	Imprecision	Other considerations	scheduled post-operative follow up	no post-operative follow up	Relative (95% CI)	Absolute (95% CI)		
**Weight loss/weight regain (follow up: range 6 months to 12 months; assessed with: %EWL)**												
5	randomised trials	serious	serious	not serious	not serious	strong association	163	163	-	MD **1.6 higher** (0.82 higher to 2.38 higher)	⨁⨁⨁◯ MODERATE	

CI: Confidence interval; **MD:** Mean difference
