# Supplementary material for: Clinical practice guidelines of the European Association for Endoscopic Surgery (EAES) on bariatric surgery: update 2020 endorsed by IFSO-EC, EASO and ESPCOP
Source: Surg Endosc. 2020 Apr 23;34(6):2332–58. doi: 10.1007/s00464-020-07555-y (PMC7214495; doi:10.1007/s00464-020-07555-y)
Supplement: Supplementary file 39 — Supplementary file39 (PDF 63 kb) [file 464_2020_7555_MOESM39_ESM.pdf]

Question: Should treatment with ursodeoxycolic acid vs. no treatment with ursodeoxycolic acid be used for prevention of gallstone disease after bariatri surgery?

Certainty assessment							№ of patients		Effect		Certainty	Importance
№ of studies	Study design	Risk of bias	Inconsistency	Indirectness	Imprecision	Other considerations	treatment with ursodeoxycolic acid	no treatment with ursodeoxycolic acid	Relative (95% CI)	Absolute (95% CI)		
**Gallstone formation (follow up: range 3 months to 18 months)**												
8	randomised trials	very serious	not serious	not serious	not serious	publication bias strongly suspected	62/825 (7.5%)	64/548 (11.7%)	**OR 0.20** (0.13 to 0.33)	**91 fewer per 1.000** (from 100 fewer to 75 fewer)	⨁◯◯◯ VERY LOW	

CI: Confidence interval; **OR:** Odds ratio
