# Supplementary material for: Clinical practice guidelines of the European Association for Endoscopic Surgery (EAES) on bariatric surgery: update 2020 endorsed by IFSO-EC, EASO and ESPCOP
Source: Surg Endosc. 2020 Apr 23;34(6):2332–58. doi: 10.1007/s00464-020-07555-y (PMC7214495; doi:10.1007/s00464-020-07555-y)
Supplement: Supplementary file 40 — Supplementary file40 (PDF 104 kb) [file 464_2020_7555_MOESM40_ESM.pdf]

Question: Should nutritional (micro and/or macronutrients) supplementation vs. no supplementation be used in in patients undergoing bariatric surgery?

Certainty assessment							№ of patients		Effect		Certainty	Importance
**№ of studies**	**Study design**	**Risk of bias**	**Inconsistency**	**Indirectness**	**Imprecision**	**Other considerations**	**nutritional (micro and/or macronutrients) supplementation**	**no supplementation**	**Relative (95% CI)**	**Absolute (95% CI)**		
**Vitamin D deficiency (follow up: mean 1 years; assessed with: vitamin D serum levels)**												
5	randomised trials	serious	serious	not serious	not serious	publication bias strongly suspected			**RR 3.82** (1.70 to 8.57)	**4 fewer per 1.000** (from 9 fewer to 2 fewer)	⨁◯◯◯ VERY LOW	

CI: Confidence interval; **RR:** Risk ratio
