# Supplementary material for: Clinical practice guidelines of the European Association for Endoscopic Surgery (EAES) on bariatric surgery: update 2020 endorsed by IFSO-EC, EASO and ESPCOP
Source: Surg Endosc. 2020 Apr 23;34(6):2332–58. doi: 10.1007/s00464-020-07555-y (PMC7214495; doi:10.1007/s00464-020-07555-y)
Supplement: Supplementary file 41 — Supplementary file41 (PDF 102 kb) [file 464_2020_7555_MOESM41_ESM.pdf]

Question: Should PPI treatment vs. no treatment be used in patients undergoing bariatric surgery?

Certainty assessment							№ of patients		Effect		Certainty	Importance
№ of studies	Study design	Risk of bias	Inconsistency	Indirectness	Imprecision	Other considerations	PPI treatment	no treatment	Relative (95% CI)	Absolute (95% CI)		
**Marginal ulcer formation (follow up: range 6 months to 144 months)**												
3	observational studies	serious	not serious	serious	not serious	strong association	24/315 (7.6%)	121/707 (17.1%)	**OR 0.50** (0.29 to 0.90)	**78 fewer per 1.000** (from 115 fewer to 14 fewer)	⨁⨁⨁◯ MODERATE	

CI: Confidence interval; **OR:** Odds ratio
