# Supplementary material for: Clinical practice guidelines of the European Association for Endoscopic Surgery (EAES) on bariatric surgery: update 2020 endorsed by IFSO-EC, EASO and ESPCOP
Source: Surg Endosc. 2020 Apr 23;34(6):2332–58. doi: 10.1007/s00464-020-07555-y (PMC7214495; doi:10.1007/s00464-020-07555-y)
Supplement: Supplementary file 42 — Supplementary file42 (PDF 111 kb) [file 464_2020_7555_MOESM42_ESM.pdf]

Question: Should post-operative diet vs. no post-operative diet be used in patients undergoing bariatric surgery?

Certainty assessment							№ of patients		Effect		Certainty	Importance
№ of studies	Study design	Risk of bias	Inconsistency	Indirectness	Imprecision	Other considerations	post-operative diet	no post-operative diet	Relative (95% CI)	Absolute (95% CI)		
**Weight (follow up: mean 12 months; assessed with: kg)**												
6	randomised trials	serious	serious	serious	serious	none	205	196	-	MD **4.55 higher** (13.08 higher to 3.99 higher)	⨁◯◯◯ VERY LOW	
**Percentage excess weight change (follow up: mean 12 months)**												
4	randomised trials	serious	serious	serious	not serious	none	106	105	-	MD **10.85 higher** (19.02 higher to 2.69 higher)	⨁◯◯◯ VERY LOW	
**BMI (follow up: mean 12 months)**												
3	randomised trials	serious	serious	serious	not serious	none	106	96	-	MD **2.77 lower** (0.72 lower to 6.25 lower)	⨁◯◯◯ VERY LOW	
**Weight change (follow up: mean 24 years; assessed with: kg)**												
3	randomised trials	serious	serious	serious	not serious	none	32	30	-	MD **12.96 lower** (4.26 lower to 21.66 lower)	⨁◯◯◯ VERY LOW	

CI: Confidence interval; **MD:** Mean difference
