# Supplementary material for: Clinical practice guidelines of the European Association for Endoscopic Surgery (EAES) on bariatric surgery: update 2020 endorsed by IFSO-EC, EASO and ESPCOP
Source: Surg Endosc. 2020 Apr 23;34(6):2332–58. doi: 10.1007/s00464-020-07555-y (PMC7214495; doi:10.1007/s00464-020-07555-y)
Supplement: Supplementary file 43 — Supplementary file43 (PDF 103 kb) [file 464_2020_7555_MOESM43_ESM.pdf]

Question: Should delayed pregnancy vs. early pregnancy be used for prevention of fetal complications?

Certainty assessment							№ of patients		Effect		Certainty	Importance
№ of studies	Study design	Risk of bias	Inconsistency	Indirectness	Imprecision	Other considerations	delayed pregnancy	early pregnancy	Relative (95% CI)	Absolute (95% CI)		
**Gestational diabetes**												
5	observational studies	serious	serious	serious	not serious	none	12/115 (10.4%)	19/212 (9.0%)	**OR 1.73** (0.30 to 9.81)	**56 more per 1.000** (from 61 fewer to 402 more)	⨁◯◯◯ VERY LOW	

CI: Confidence interval; **MD:** Mean difference
