# Supplementary material for: Clinical practice guidelines of the European Association for Endoscopic Surgery (EAES) on bariatric surgery: update 2020 endorsed by IFSO-EC, EASO and ESPCOP
Source: Surg Endosc. 2020 Apr 23;34(6):2332–58. doi: 10.1007/s00464-020-07555-y (PMC7214495; doi:10.1007/s00464-020-07555-y)
Supplement: Supplementary file 44 — Supplementary file44 (PDF 70 kb) [file 464_2020_7555_MOESM44_ESM.pdf]

Question: Should EndoBarrier® vs. non-surgical management be used for weight loss?

Certainty assessment							№ of patients		Effect		Certainty	Importance
№ of studies	Study design	Risk of bias	Inconsistency	Indirectness	Imprecision	Other considerations	Endobarrier	non-surgical management	Relative (95% CI)	Absolute (95% CI)		
**%EWL (follow up: mean 9.2 months)**												
4	randomised trials	not serious	not serious	not serious	serious [a]	none	301		-	MD **36.9 % more** (29.2 more to 44.6 more)	⨁⨁⨁◯ MODERATE	CRUCIAAL
**Improvement in %HbA1c (follow up: mean 8.4 months)**												
14	observational studies	serious [b]	serious [c]	not serious	not serious	publication bias strongly suspected [d]	388		-	MD **1.29 % more** (0.97 more to 1.62 more)	⨁◯◯◯ VERY LOW	BELANGRIJK
**Morbidity (follow up: mean 1 years)**												
3	randomised trials	not serious	not serious	not serious	very serious [e]	none	12/54 (22.2%)	0/47 (0.0%)	**OR 0.27** (0.13 to 0.41)	**0 fewer per 1.000** (from 0 fewer to 0 fewer)	⨁⨁◯◯ LOW	CRUCIAAL

CI: Confidence interval; **MD:** Mean difference; **OR:** Odds ratio
