# Supplementary material for: Clinical practice guidelines of the European Association for Endoscopic Surgery (EAES) on bariatric surgery: update 2020 endorsed by IFSO-EC, EASO and ESPCOP
Source: Surg Endosc. 2020 Apr 23;34(6):2332–58. doi: 10.1007/s00464-020-07555-y (PMC7214495; doi:10.1007/s00464-020-07555-y)
Supplement: Supplementary file 45 — Supplementary file45 (PDF 71 kb) [file 464_2020_7555_MOESM45_ESM.pdf]

Question: Should AspireAssist® vs. non-surgical management be used for weight loss?

Certainty assessment							№ of patients		Effect		Certainty	Importance
**№ of studies**	**Study design**	**Risk of bias**	**Inconsistency**	**Indirectness**	**Imprecision**	**Other considerations**	**Aspire Assist**	**non-surgical management**	**Relative (95% CI)**	**Absolute (95% CI)**		
**%EWL (follow up: mean 1 years)**												
1	randomised trials	not serious [a]	not serious	not serious	serious [b]	none	82	31	-	MD **21.7 % more** (13.75 more to 29.65 more)	⊕⊕⊕◯ MODERATE	CRUCIAAL
**IWQL (follow up: mean 1 years)**												
1	randomised trials	not serious	not serious	not serious	serious [c]	none	82	31	-	MD **2.9 higher** (1.66 lower to 7.46 higher)	⊕⊕⊕◯ MODERATE	BELANGRIJK
**Improvement in %HbA1c (follow up: mean 1 years)**												
1	randomised trials	not serious	not serious	not serious	not serious	none	82	31	-	MD **0.14 more** (0 to 0.28 more)	⊕⊕⊕⊕ HIGH	BELANGRIJK
**Morbidity (follow up: mean 1 years)**												
1	randomised trials	not serious	not serious	not serious	very serious [d]	none	5/82 (6.1%)	0/31 (0.0%)	**OR 4.47** (0.24 to 83.27)	**0 fewer per 1.000** (from 0 fewer to 0 fewer)	⊕⊕◯◯ LOW	CRUCIAAL

CI: Confidence interval; **MD:** Mean difference; **OR:** Odds ratio
