# Supplementary material for: Clinical practice guidelines of the European Association for Endoscopic Surgery (EAES) on bariatric surgery: update 2020 endorsed by IFSO-EC, EASO and ESPCOP
Source: Surg Endosc. 2020 Apr 23;34(6):2332–58. doi: 10.1007/s00464-020-07555-y (PMC7214495; doi:10.1007/s00464-020-07555-y)
Supplement: Supplementary file 46 — Supplementary file46 (PDF 63 kb) [file 464_2020_7555_MOESM46_ESM.pdf]

Question: Should abiliti® vs. non-surgical management be used for weight loss?

Certainty assessment							Impact	Certainty	Importance
№ of studies	Study design	Risk of bias	Inconsistency	Indirectness	Imprecision	Other considerations			
**Weight loss (%EWL) (follow up: mean 1 years)**									
3	observational studies	serious [a]	not serious	not serious	serious [b]	none	In three studies, with a total of 102 subjects receiving Abiliti, the device demonstrated significant excess weight loss after 1 year, respectively: 28.7% EWL (n=31) , 40.7 (n=45) and 49.3% (n=26). One study shows stable weight loss of 27.5% EWL after a period of 27 months (n=16).	⨁◯◯◯ VERY LOW	CRUCIAAL
**Morbidity**									
3	observational studies	not serious	not serious	not serious	not serious		Procedure is well-tolerated with self-limiting adverse events, mainly abdominal pain. Reported SAE's across studies were severe recurrent seroma (n=1) and postsurgical superficial wound infection (n=1)	-	CRUCIAAL

CI: Confidence interval; **MD:** Mean difference
