# Supplementary material for: Clinical practice guidelines of the European Association for Endoscopic Surgery (EAES) on bariatric surgery: update 2020 endorsed by IFSO-EC, EASO and ESPCOP
Source: Surg Endosc. 2020 Apr 23;34(6):2332–58. doi: 10.1007/s00464-020-07555-y (PMC7214495; doi:10.1007/s00464-020-07555-y)
Supplement: Supplementary file 47 — Supplementary file47 (PDF 70 kb) [file 464_2020_7555_MOESM47_ESM.pdf]

Question: Should VBloc vs. non-surgical management be used for weight loss?

Certainty assessment							№ of patients		Effect		Certainty	Importance
**№ of studies**	**Study design**	**Risk of bias**	**Inconsistency**	**Indirectness**	**Imprecision**	**Other considerations**	**VBloc**	**non-surgical management**	**Relative (95% CI)**	**Absolute (95% CI)**		
**%EWL (follow up: mean 1 years)**												
2	randomised trials	serious [a]	not serious	not serious	not serious	none	One randomized double blind multicenter trial (n=192) and one randomized sham controlled trial (n=162) demonstrate excess weight loss of respectively 17% and 24.4% after 1 year of Vbloc-therapy. Unblinded follow up of VBloc-arm (n=123) of the sham controlled trial suggests sustained weight loss up till 24 months.				⨁⨁⨁◯ MODERATE	CRUCIAAL
**IWQL (follow up: mean 1 years)**												
1	randomised trials	not serious	not serious	not serious	not serious	none	192	102	-	MD **2 more** (1.52 more to 2.48 more)	⨁⨁⨁⨁ HIGH	BELANGRIJK
**Morbidity (follow up: mean 1 years)**												
2	randomised trials	not serious	not serious	not serious	very serious [b]	none	38/354 (10.7%)	12/179 (6.7%)	**OR 3.05** (0.16 to 56.73)	**113 more per 1.000** (from 56 fewer to 736 more)	⨁⨁◯◯ LOW	CRUCIAAL

CI: Confidence interval; **MD:** Mean difference; **OR:** Odds ratio
