# Supplementary material for: Clinical practice guidelines of the European Association for Endoscopic Surgery (EAES) on bariatric surgery: update 2020 endorsed by IFSO-EC, EASO and ESPCOP
Source: Surg Endosc. 2020 Apr 23;34(6):2332–58. doi: 10.1007/s00464-020-07555-y (PMC7214495; doi:10.1007/s00464-020-07555-y)
Supplement: Supplementary file 48 — Supplementary file48 (PDF 104 kb) [file 464_2020_7555_MOESM48_ESM.pdf]

Question: Should Duodenal Mucosal Resurfacing (DMR) vs. non-surgical management be used for weight loss?

Certainty assessment							Impact	Certainty	Importance
№ of studies	Study design	Risk of bias	Inconsistency	Indirectness	Imprecision	Other considerations			
**Weight loss (follow up: mean 1 years)**									
1	observational studies	serious [a]	not serious	not serious	not serious	none	One cohortstudy (n=46) reports weight loss of 3.1 kg and 2.1kg after respectively 6 and 12 months.	⨁◯◯◯ VERY LOW	CRUCIAAL
**Hba1C% improvement (follow up: mean 1 years)**									
1	observational studies	serious [a]	not serious	not serious	not serious	none	One cohortstudy (n=46) reports slight improvement in HbA1C levels of 1.0% after 12 months.	⨁◯◯◯ VERY LOW	BELANGRIJK

CI: Confidence interval
