# Supplementary material for: Clinical practice guidelines of the European Association for Endoscopic Surgery (EAES) on bariatric surgery: update 2020 endorsed by IFSO-EC, EASO and ESPCOP
Source: Surg Endosc. 2020 Apr 23;34(6):2332–58. doi: 10.1007/s00464-020-07555-y (PMC7214495; doi:10.1007/s00464-020-07555-y)
Supplement: Supplementary file 49 — Supplementary file49 (PDF 107 kb) [file 464_2020_7555_MOESM49_ESM.pdf]

Question: Should Pose® vs. non-surgical management be used for weight loss?

Certainty assessment							Impact	Certainty	Importance
№ of studies	Study design	Risk of bias	Inconsistency	Indirectness	Imprecision	Other considerations			
**Weight loss (follow up: mean 1 years)**									
3	randomised trials	serious	not serious	not serious	not serious	none	Total of 359 patients receiving POSE. Two studies ((nonblinded RCT and prospective cohort) showed mean excess weight loss of 45% after 1 year in respectively 30 and 116 patients. Another study (sham-controlled RCT) showed excess weight loss of 16% after 1 year in 213 patients.	⊕⊕⊕◯ MODERATE	CRUCIAAL
**Resolution of diabetes (follow up: mean 1 years)**									
1	randomised trials	serious	not serious	not serious	not serious	none	One sham-controlled trial (n=213) reports resolution in 9/16 diabetic subjects (56,3%) after 1 year	⊕⊕⊕◯ MODERATE	BELANGRIJK
**Quality of life (follow up: mean 1 years)**									
1	randomised trials	serious	not serious	not serious	not serious	none	One sham-controlled trial (n=213) reports significant improvement from severe to a borderline mild impact on quality of life after 1 year when compared to sham subjects.	⊕⊕⊕◯ MODERATE	CRUCIAAL
**Morbidity (follow up: mean 1 years)**									
1	randomised trials	serious	not serious	not serious	not serious	none	1% reported SAE's: extragastric bleed (n=1) and hepatic abcess (n=1)	⊕⊕⊕◯ MODERATE	CRUCIAAL

CI: Confidence interval
