# Supplementary material for: Clinical practice guidelines of the European Association for Endoscopic Surgery (EAES) on bariatric surgery: update 2020 endorsed by IFSO-EC, EASO and ESPCOP
Source: Surg Endosc. 2020 Apr 23;34(6):2332–58. doi: 10.1007/s00464-020-07555-y (PMC7214495; doi:10.1007/s00464-020-07555-y)
Supplement: Supplementary file 50 — Supplementary file50 (PDF 106 kb) [file 464_2020_7555_MOESM50_ESM.pdf]

Question: Should Overstitch (endoscopic sleeve gastroplasty) vs. non-surgical management be used for weight loss ?

Certainty assessment									
№ of studies	Study design	Risk of bias	Inconsistency	Indirectness	Imprecision	Other considerations	Impact	Certainty	Importance
**Weight loss (%EWL) (follow up: range 1 years to 2 years)**									
5	observational studies	serious [a]	not serious	not serious	not serious	none	In 209 patients the procedure resulted in a mean excess weight loss of 50%, with durability of plications. Three studies report progressive weight loss up till 24 months after procedure, respectively: 20.9% TBWL (n=8), 18.6% TBWL (n=57) , 60.4% EWL (n=28).	⨁◯◯◯ VERY LOW	CRUCIAAL
**Improvement in HbA1c% (follow up: mean 2 years)**									
1	observational studies	serious [a]	not serious	not serious	not serious	none	One study (n=91) demonstrated resolution of diabetes in 2 subjects (15.4%) 2 years after procedure.	⨁◯◯◯ VERY LOW	BELANGRIJK
**Morbidity (follow up: range 1 years to 2 years)**									
5	observational studies	serious [a]	not serious	not serious	not serious	none	Adverse events were mainly nausea and post-discharge abdominal pain and were self-limiting. Reported serious adverse events among 209 subjects ;perigastric inflammatory fluid collections (n=3), extra gastric hemorrhage requiring blood transfusion (n=1), pulmonary embolism (n=1) pneumoperitoneum and pneumothorax requiring chest tube placement (n=1)	⨁◯◯◯ VERY LOW	CRUCIAAL

CI: Confidence interval
